# Flavylium Salts: A Blooming Core for Bioinspired Ionic Liquid Crystals

**DOI:** 10.1002/chem.201901975

**Published:** 2019-09-18

**Authors:** Robert Forschner, Jakob Knelles, Korinna Bader, Carsten Müller, Wolfgang Frey, Andreas Köhn, Yann Molard, Frank Giesselmann, Sabine Laschat

**Affiliations:** ^1^ Institut für Organische Chemie, Universität Stuttgart Pfaffenwalding 55 70569 Stuttgart Germany; ^2^ Institut für Physikalische Chemie Universität Stuttgart Pfaffenwalding 55 70569 Stuttgart Germany; ^3^ Institut für Theoretische Chemie Universität Stuttgart Pfaffenwaldring 55 70569 Stuttgart Germany; ^4^ CNRS, ISCR-UMR6226, ScanMAT-UMS 2001 University Rennes 35000 Rennes France

**Keywords:** fluorescence, ionic liquid crystals, self-assembly, UV/Vis spectroscopy, X-ray diffraction

## Abstract

Thermotropic ionic liquid crystals based on the flavylium scaffold have been synthesized and studied for their structure‐properties relationship for the first time. The mesogens were probed by differential scanning calorimetry (DSC), polarizing optical microscopy (POM), and X‐ray diffraction (XRD). Low numbers of alkoxy side chains resulted in smectic (SmA) and lamello‐columnar (Lam_Col_) phases, whereas higher substituted flavylium salts showed Col_ro_ as well as ordered and disordered columnar (Col_ho_, Col_hd_) mesophases. Mesophase width ranged from 13 K to 220 K, giving access to room temperature liquid crystals. The optical properties of the synthesized compounds were probed towards absorption and emission properties. Strong absorption with maxima between 444 and 507 nm was observed, and some chromophores were highly emissive with quantum yields up to 99 %. Ultimately, mesogenic and dye properties were examined by temperature‐dependent emissive experiments in the solid state.

## Graphical abstract

**Flower power**: In the search for new mesogenic cores for ionic liquid crystals, the authors were inspired by anthocyanidins, which are responsible for the bright red to blue colors in many plants. The new flavylium triflates were examined for their liquid crystalline and dye properties.

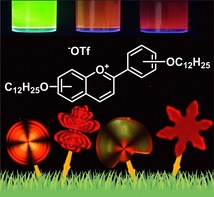

## Introduction

Ionic liquid crystals (ILCs) are an emerging class of soft matter materials which combine the best of two worlds, that is, the fluidity and adjustable polarity of ionic liquids with the anisotropic properties of liquid crystals.^1^, ^2^, ^3^, ^4^, ^5^, ^6^ The vast majority of ILCs consist of nitrogen‐containing cations, such as ammonium, pyridinium, imidazolium or guanidinium salts and analogues thereof, whereas cations with other heteroatoms (O, S, P, …) are less commonly employed.

Although oxonium ions usually only occur as short‐lived intermediates in reactions, their high reactive character can be tamed by embedding the positively charged oxygen in an aromatic system. Such pyrylium derivatives, for example, 2,4,6‐triphenylpyrylium salts, show strong fluorescence and anion–π interactions.^7^, ^8^ They have been successfully utilized for electron‐transfer reactions,^9^ (photo)organocatalysis,^10^, ^11^, ^12^ white‐light fluorophores^13^ and laser dyes.^14^

**Scheme 1:**
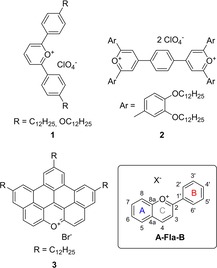
Examples of liquid crystalline oxonium salts reported in the literature and the basic structure of the flavylium salts **A‐Fla‐B**.

Liquid crystalline oxonium salts reported in the literature (Scheme 1) are solely based on di‐ or triphenyl pyrylium cations **1**,^15^ 1,4‐disubstituted benzenes functionalized with two pyrylium units **2** 16 and condensed xanthylium derivative BNAX **3**.^17^ We found it quite surprising that ILCs based on the probably most prominent organic oxonium salt, the flavylium cation **A‐Fla‐B** never has been reported to the best of our knowledge. Those salts are an important scaffold in natural and synthetic dyes. For example, anthocyanins, that is, hydroxylated and O‐glycosylated flavylium salts provide the largest family of water‐soluble plant dyes, which protect plants against photooxidation, serve as food colorants^18^ and attractant for insects.^19^ Key features of these natural dyes are their pH‐dependent colored species ranging from red to blue, their ability for complexation of metals and formation of different types of aggregates.^20^, ^21^ Synthetic flavylium derivatives have been successfully utilized for organic–inorganic hybrid pigments,^22^ or photosensitizer for dye‐sensitized solar cells.^23^, ^24^, ^25^, ^26^

Because of their nearly planar structure and the strong tendency for aggregation into dimers and higher aggregates,^27^ they are particularly attractive candidates for ILCs. Additionally, flavylium salts possess unique structural features, that is, the ionic moiety is located in the center of the mesogenic core rather than as peripheral headgroup and the molecular shape is unsymmetrical. We anticipated that the substitution pattern of the rigid A and flexible B ring should enable tailoring of both liquid crystalline self‐assembly as well as linear optical properties of flavylium salts. Here, we report the first flavylium ILCs showing a rich polymorphism and promising absorption and emission characteristics. The results are discussed below.

## Results and Discussion

### Synthesis

**Scheme 2:**
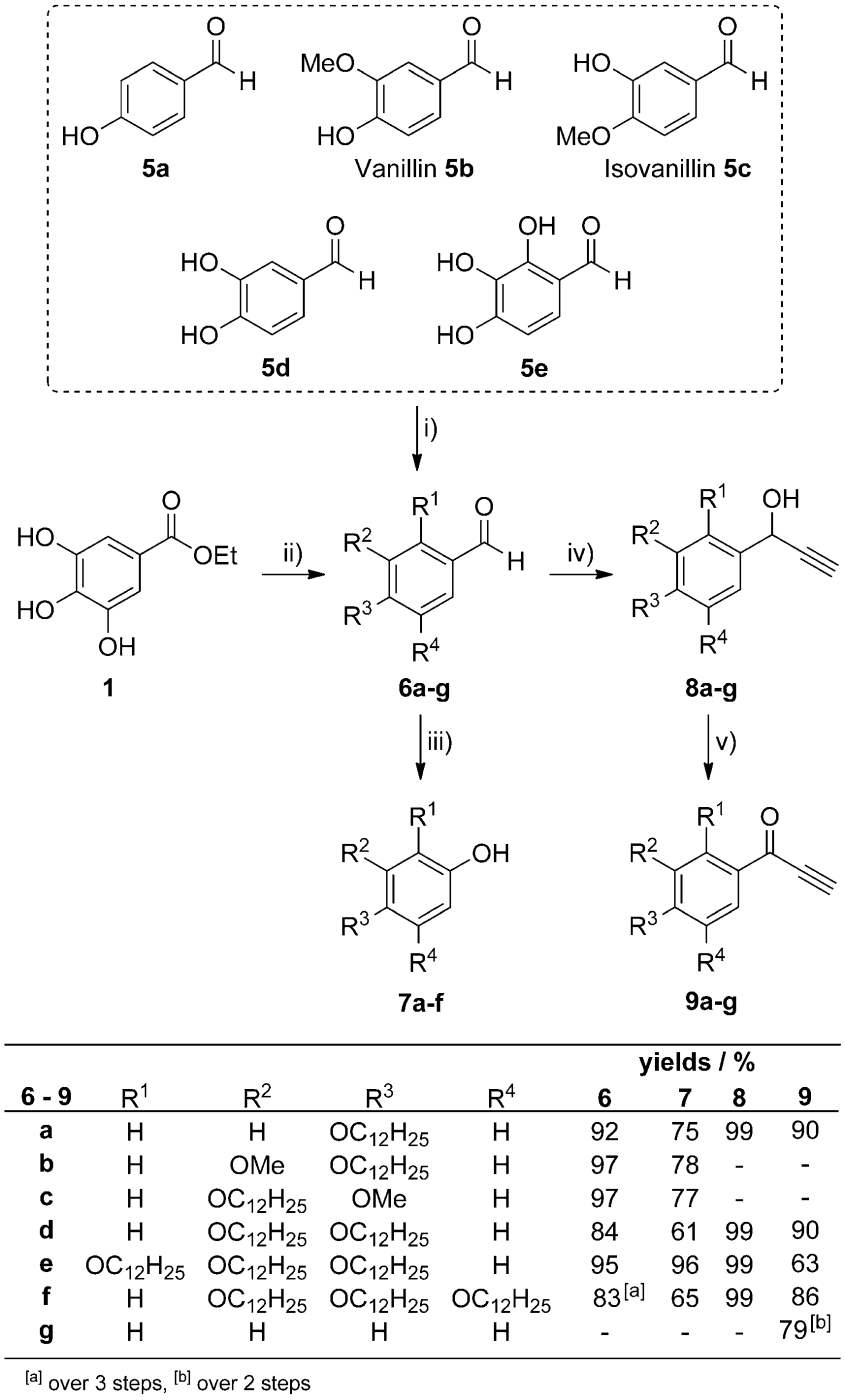
i) C_12_H_25_Br, K_2_CO_3_, DMF, 80 °C, 3 h; ii) 1. C_12_H_25_Br, K_2_CO_3_, NaI, CH_3_CN, 105 °C, 2 d; 2. LiAlH_4_, Et_2_O, RT, 1 h; 3. DDQ, 1,4‐dioxane, RT, 1 h; iii) H_2_O_2_, H_2_SO_4_, CHCl_3_, CH_3_OH, RT, 18 h; iv) ethynylmagnesium bromide, THF, RT, 3 h; v) IBX, EtOAc, 80 °C, 18 h.

For the syntheses of the desired flavylium salts a protocol by Chassaing^28^ was applied were the A ring of the flavylium salt is derived from a phenol and the B and C rings are generated from an ethynyl ketone. As shown in Scheme 2, a series of hydroxy substituted arylaldehydes **5 a**–**e** was converted into the corresponding alkoxy‐substituted arylaldehydes **6 a**–**e** through Williamson etherification in 84–97 % yields, except for the 3,4,5‐trisdodecyloxyphenylcarbaldehyde **6 f**, which was obtained in three steps from ethyl gallate **1** in 83 % overall yield.^29^ Aldehydes **6 a**–**f** and benzaldehyde **6 g** were then treated with ethynyl magnesium bromide to give the propargylic alcohols **8 a**–**g** in almost quantitative yield after aqueous workup, followed by 2‐iodoxybenzoic acid (IBX) oxidation to the corresponding ethynyl ketones **9 a**–**g**. Dakin oxidation with H_2_O_2_ and H_2_SO_4_ of aldehydes **6 a**–**f** yielded in the phenol derivatives **7 a**–**f** in 61–96 %.^30^

**Scheme 3:**
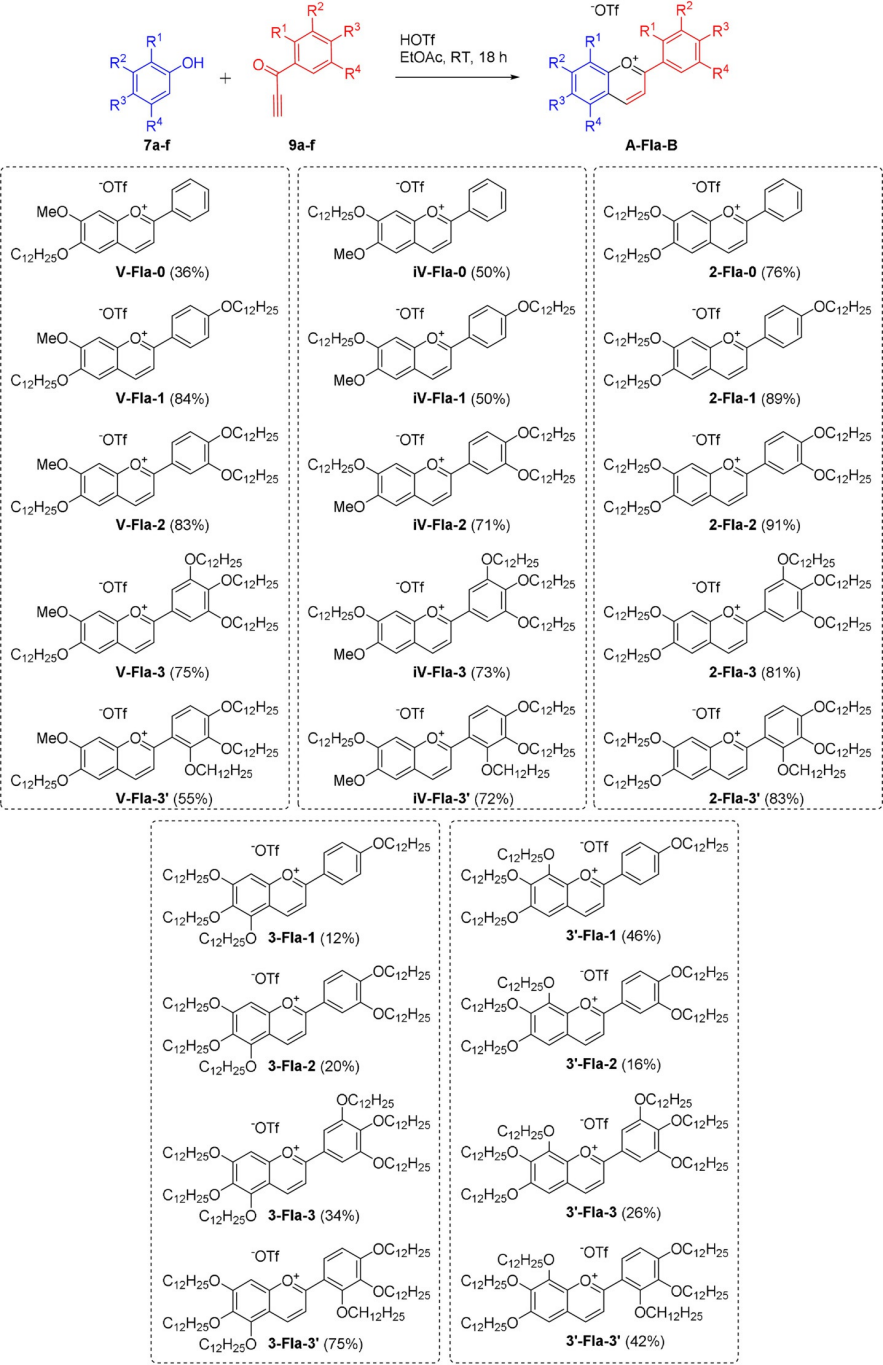
Molecular structures of the flavylium salts **A‐Fla‐B** prepared in this work.

Treatment of an equimolar solution of a phenol **7 a**–**f** and an ethynyl ketone **9 a**–**g** in EtOAc with an excess of trifluoromethanesulfonic acid resulted in the formation of the desired flavylium salt **A‐Fla‐B** (Scheme 3). The variation in the yields of the flavylium salt **A‐Fla‐B** was mostly due to the differences in solubility. Some derivatives, for example, **V‐Fla‐1** and **2‐Fla‐2**, show low solubility in EtOAc and precipitated directly from the reaction solution at room temperature. Others, like **3‐Fla‐1** and **3′‐Fal‐3′** display good solubility at room temperature and, therefore, were recrystallized at low temperatures. The crude products were purified by recrystallization from the reaction solution. With **7 a** no formation of the product was observed, therefore the alkoxy substitution in *meta*‐position is crucial for the reaction. This is the reason why phenols **7 b** and **7 c** with an additional methoxy substituent were used to obtain the derivatives with one alkoxy sidechain attached to the A ring. The flavyliums salt **3‐Fla‐0** could not be isolated and **3′‐Fla‐0** could not be obtained in satisfactory purity. It should be emphasized that the solid flavylium salts were bench stable and solutions did not show any color change or loss of color upon storage over more than six months.

### Solid‐state properties

**Figure 1:**
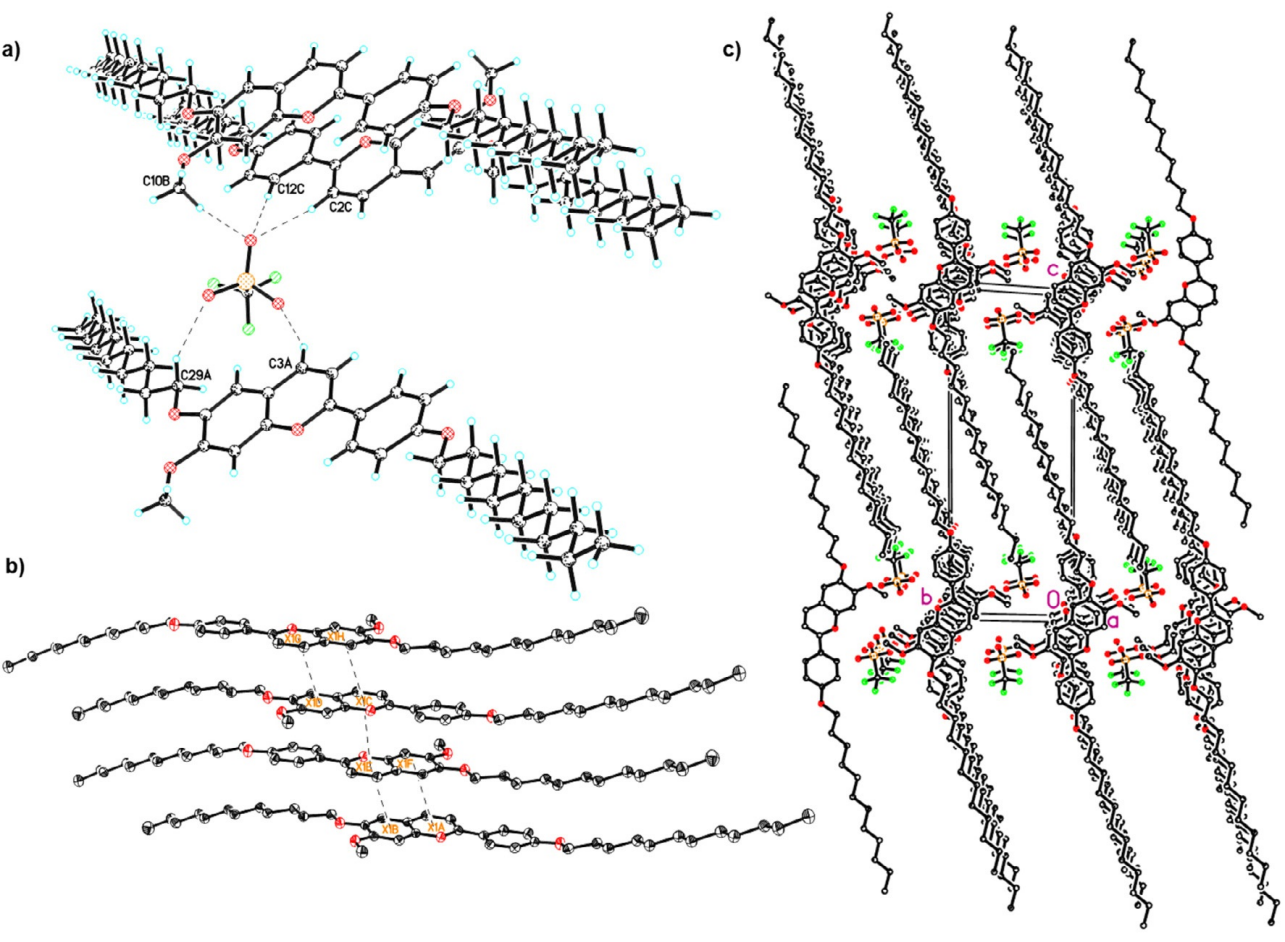
Single‐crystal X**‐**ray structure representations of **V‐Fla‐1** in the solid state^31^ (H=light blue, C=white, O=red, S=yellow, F=green, in b) and c) hydrogens are omitted for clarity). a) Hydrogen‐bond interactions of the triflate anion given by dashed lines. b) Stacking interaction of the flavylium cation. c) *bc* view along the *a* axis showing the interdigitated layer structure.

Recrystallization of **V‐Fla‐1** from EtOAc provided suitable crystals for single‐crystal analysis. The compound crystallizes with one ion pair in the asymmetric unit of the centrosymmetric space group *P* $\bar{1}$ . The flavylium cation is almost planar with a torsion angle of 5° between the chromenylium and the phenyl moiety. The oxygen atoms of the triflate anion works as acceptors for a couple of hydrogen bond interactions (Figure 1 a). Firstly, there are π(C−H) donors of the chromenylium and the phenyl moieties. The (H⋅⋅⋅O) interval of the relevant distances is 2.34– 2.39 Å. Secondly, a weaker interaction is evident with the methyl C−H function of the methoxy group. The (H⋅⋅⋅O) distance range is 2.54–2.70 Å. And finally, there is a weak interaction between a C−H donor of the alkyl chain and the O6 of the triflate anion with a (C29−H29⋅⋅⋅O6) distance of 2.48 Å. The cation built up a layer type stacking interaction with a pairwise 180° rotated orientation of the molecules forced by a slight π–π stacking interaction of the chromenylium cores (Figure 1 b). In detail, the pyrylium core interacts with the benzene part of the chromenylium and vice versa. The distance of the centroids is in both cases 3.66 Å. Additional each pair is also stabilized by a slight stronger π–π stacking generated only between the pyrylium cores with a distance of 3.56 Å. Remarkably, the phenyl groups of the flavylium moieties are not involved in π–π stacking interactions. This is most likely due to the strong ionic and dipolar interaction of the benzopyrylium moiety, surpassing the possible contribution of the π–π interaction from the phenyl group. This packing motive seems to be universal for flavylium cations and will be important for the following discussion of the liquid crystalline properties. In the *bc* view of the packing diagram there is a layer‐type orientation of the molecules along the *c*‐axis evident (Figure 1 c). The central part of the cation and the triflate anions form a polar layer which alternates with the nonpolar layer consisting of the aliphatic interdigitated chains.

### Liquid crystalline properties

**Figure 2:**
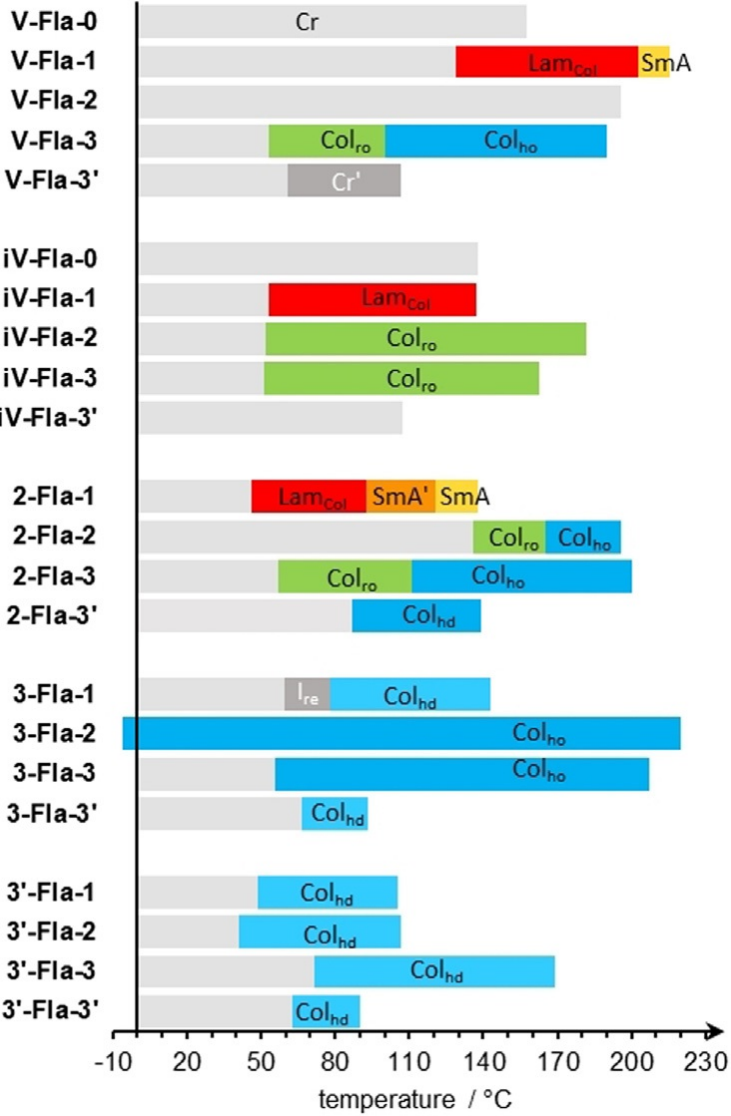
Overview of the observed mesophase of the flavylium salts **A‐Fla‐B** (a detailed version of this diagram with the mesophase width can be found in Figure S1, Supporting Information).

The thermotropic behavior of the synthesized flavylium salts **A‐Fla‐B** were examined by polarizing optical microscopy (POM), differential scanning calorimetry (DSC) as well as wide‐ and small‐angle X‐ray scattering (WAXS and SAXS). The results of the DSC experiments are summarized in Table S1 (Supporting Information) and an overview of the observed phases is presented in Figure 2.

Some compounds show decomposition of the material in the DSC. However, the TGA measurements of the series **iV‐Fla‐B** showed that the compounds are stable to 200 °C, except for **iV‐Fla‐3**, which decomposes at about 150 °C (Figure S7, Supporting Information). Flavylium salts with no side chain on the B ring, that is, **V‐Fla‐0**, **iV‐Fla‐0**, **2‐Fla‐0** and **3′‐Fla‐0** were non‐mesomorphic irrespective of the number and position of the side chain on the A ring and showed only Cr–Cr transitions. In addition, **V‐Fla‐2** with one side chain at the A ring and two side chains at the B ring as well as **V‐Fla‐3′** and **iV‐Fla‐3′** with sterically crowded B ring were non‐mesomorphic. For clarity, the following discussion is organized according to the type of mesophase (lamellar, columnar rectangular and/or hexagonal) formed by the flavylium salt.

### Flavylium salts with lamellar mesophases

**Figure 3:**
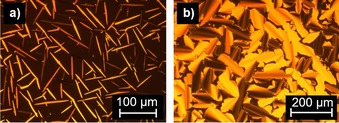
POM micrographs of a) **V‐Fla‐1** at 205 °C (magnification 200×) and b) at 188 °C (magnification 100×). All pictures were taken between crossed polarizers upon cooling from the isotropic phase with a cooling rate of 5 K min^−1^.

Flavylium salts with one or two side chains on the A ring and one side chain on the B ring resulted in lamellar mesophases. For example, vanillin‐derived flavylium salt **V‐Fla‐1** with one dodecyloxy chain on both A and B ring displayed two liquid crystalline phases, that is, a melting transition at 129 °C followed by a mesomorphic transition at 201 °C and a clearing point at 214 °C upon first heating in the DSC (Figure S2 b, Supporting Information). Under the POM the lower‐temperature phase showed uncharacteristic textures, whereas Maltese cross textures and a strong tendency for homeotropic alignment were observed upon heating into the high‐temperature mesophase. Upon cooling from the isotropic liquid, the compound displayed *bâtonnets* textures (Figure 3 a), indicating a SmA mesophase. Upon further cooling into the low temperature phase fan shaped textures were visible (Figure 3 b) which can be observed in smectic and columnar mesophases, but also has been reported for lamello‐columnar Lam_Col_ mesophases.^32^

**Figure 4:**
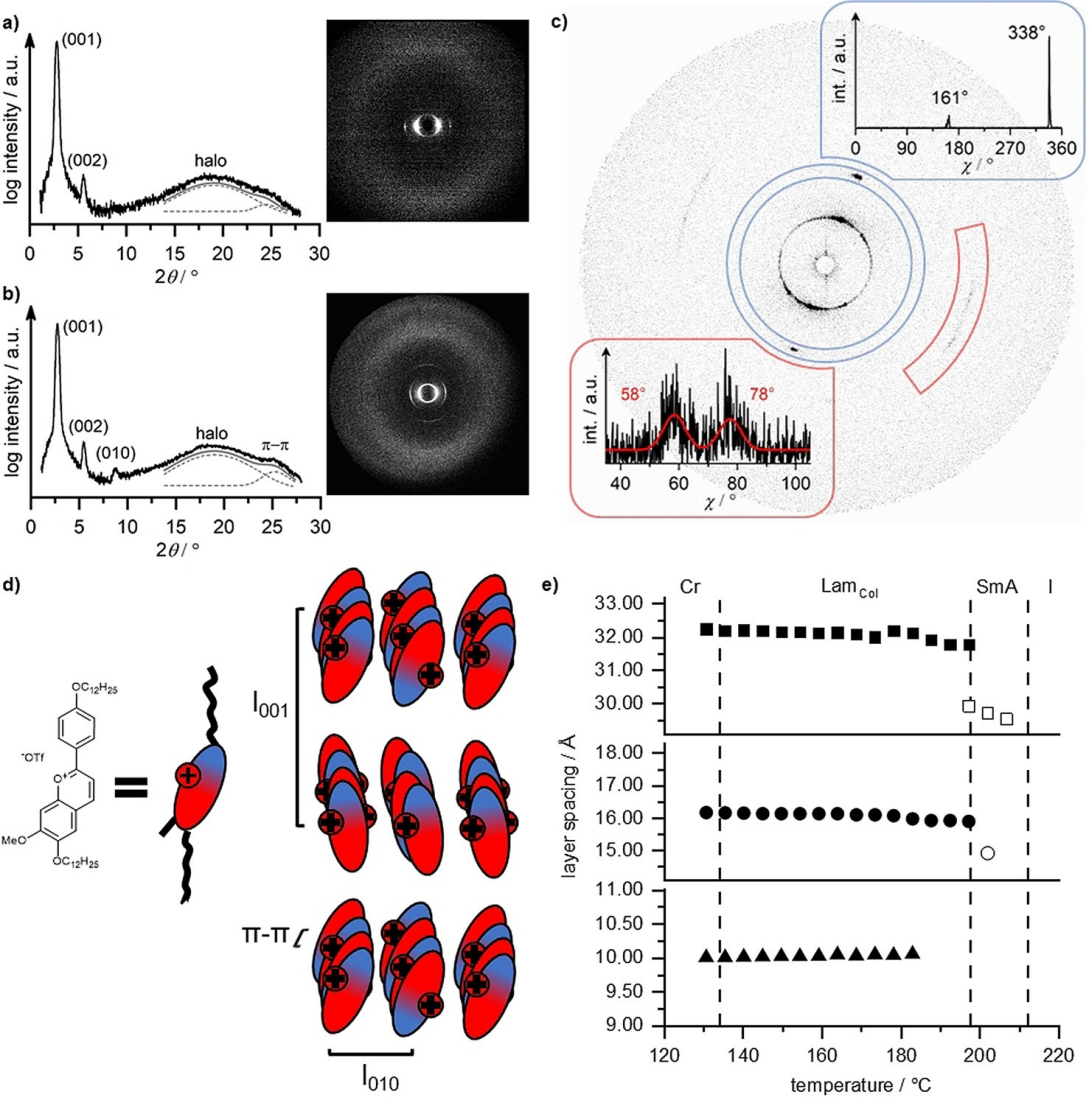
Diffractogram and the diffraction pattern of the oriented sample of **V‐Fla‐1** in the a) SmA phase at 210 °C and b) Lam_Col_ phase at 170 °C after cooling from the isotropic state (cooling rate: 5 K min^−1^). c) 2D SAXS pattern of the Lam_Col_ mesophase and the χ‐scan of the diffraction peaks obtained by slow cooling (0.2 K min^−1^) from the SmA phase into the Lam_Col_ phase. d) Proposed packing of the molecules in the Lam_Col_ mesophase. e) Temperature dependent layer spacing of the (001) reflex (▪), the (002) reflex (•) and the (010) reflex (▴) in the SmA (hollow symbols) and the Lam_Col_ phase (filled symbols) of compound **V‐Fla‐1**. The measurement was performed via the second heating (rate: 2 K min^−1^).

X‐ray diffraction of **V‐Fla‐1** showed upon cooling from the isotropic phase the typical diffraction pattern of an oriented SmA mesophase consisting of a sharp layer reflex (001) and the higher order reflex (002) in the small angle region (Figure S9 a, Supporting Information). The wide‐angle region displayed two broad halos at 4.66 and 3.64 Å resulting from the molten alkyl chains and presumably short aggregates of the mesogens, respectively (Figure 4 a). Both reflexes are oriented perpendicular to the layer reflexes. The experimental layer spacing *d=*29.7 Å from the SAXS measurement is significantly smaller than the molecular length obtained from single crystal structure analysis (*L=*41.7 Å). Usually, the layer spacing of a SmA phase is 5–10 % smaller than the molecular length, due to the axial disorder of the mesogens defined by the order parameter.^33^ In the case of **V‐Fla‐1**, the observed *d*/*L* ratio of 0.71 is unusual and cannot solely be explained by a low order parameter. However, the difference between flavylium ILCs and conventional mesogens with smectic phases is that their charge is located in the center of the molecule. The close proximity to the counter ion leads to an expansion of the effective cross section of the core. The resulting free space between the alkyl chains is then filled by interdigitation of the neighboring hydrophobic layers, forming an SmA_1_ phase. This packing model is further supported by analogy with the crystal structure of **V‐Fla‐1**, which reveals interdigitation of alkyl chains and a lattice parameter of *c=*26 Å similar to the experimentally determined layer spacing.

Cooling of the sample into the lower‐temperature phase at 170 °C, resulted in a more pronounced wide‐angle reflex at 3.54 Å as a result of the growing intracolumnar stacks, which were already weakly present in the SmA phase (Figure 4 b). Additionally, a sharp reflex with a layer spacing of 10.04 Å is observed. The perpendicular orientation towards the layer reflex, as well as the similarity of the length to the b‐axis (10.27 Å) of the single crystal structure, led to the assumption that this reflex originates from the lateral distance of the short columnar stacks. The scan over *χ* of a slowly cooled sample revealed that this reflex is split into two reflexes with ±10° with respect to the center (Figure 4 c).

Therefore, we assume that the lower‐temperature phase is a lamello‐columnar phase Lam_Col_, in which the layers are build up by short stacks of flavylium cations (Figure 4 d). Within these stacks the molecules are organized in an antiparallel manner as observed in the crystal structure, in order to enable π–π interactions and reduce charge repulsion. The splitting of the lateral intercolumnar distance can be explained by an alternating tilt of these mesogenic stacks with ±10° in respect to the layer normal from one layer to another, comparable to the anticlinic SmC phase. Due to the low number of observed reflexes and the sliding of the layers, further differentiation regarding the symmetry was not reliable. Further evidence for this hypothesis can be given by calculating the volume of the elemental cell using the length of the 001 and the 010 reflex, as well as the intracolumnar distance. With the volume in hand the number of molecules per elemental cell *Z* can be calculated according to Lehmann,^29^ which in the case of the lower‐temperature phase of **V‐Fla‐1** resulted in *Z=*1.07 assuming a density of 1 g mL^−1^.

The occurrence of the Lam_Col_ phase can be rationalized by the strong tendency for the formation of vertical aggregates of the flavylium cation and a weak layer coupling.^34^ At high temperatures the stacking is unfavorable, therefore the calamitic shaped molecules form a SmA phase as expected. However, the presence of the wide‐angle reflex corresponding to the π–π reflex indicates, that aggregation already occurs in the SmA phase. Upon decrease of the temperature the molecular stacks are growing until the phase transition into the Lam_Col_ phase occurs.

Further information of the mesophase was obtained by the temperature‐dependent SAXS measurements (Figure 4 e). In the Lam_Col_ phase, the layer spacing decreased only very slightly with increasing temperature. At the Lam_Col_–SmA phase transition the layer spacing dropped significantly by approximately 2 Å and upon further increase of temperature a negative thermal expansion of the (001) reflex was observed, which is typical for the SmA phase.

The DSC of the isovanillin‐derived flavylium salt **iV‐Fla‐1** with one dodecyloxy side chain on both A and B ring showed only one mesophase between 47 and 131 °C (Figure S3 a, Supporting Information). Under the POM an uncharacteristic grainy texture was observed. The XRD pattern showed an intense (001) reflex and the higher order reflexes (002) and (004) (Figure S10 a,b, Supporting Information). In addition, one further reflex at 10.4 Å was observed as well as a diffuse halo centered around 4.84 Å and a π–π reflex at 3.63 Å. For this compound no oriented sample could be obtained, but due to the similarity of the diffraction pattern compared to **V‐Fla‐1** we surmised that this phase is also a Lam_Col_ phase. However, it should be noted that the layer spacing of **iV‐Fla‐1** shows a stronger dependency on the temperature than **V‐Fla‐1** in the Lam_Col_ phase (Figure S10 c, Supporting Information).

**Figure 5:**
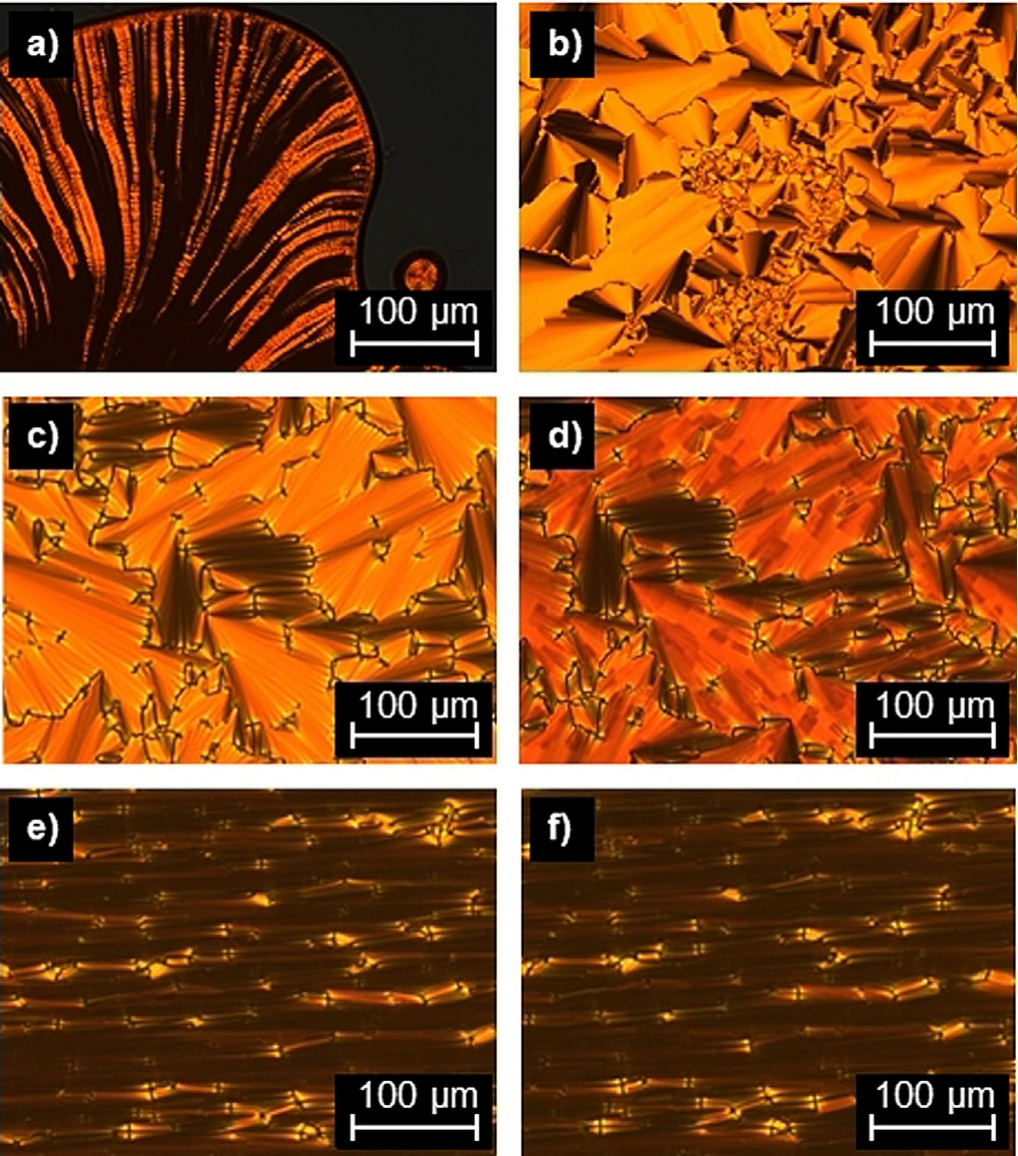
Polarized optical micrographs of a) **2‐Fla‐1** at 135 °C (magnification 100x) upon heating, b) **2‐Fla‐1** at 115 °C (magnification 100x) upon cooling from the isotropic liquid, c) fan‐shaped textures at 135 °C and at d) 110 °C. **2‐Fla‐1** in a rubbed polyimide cell (homogeneous alignment, cell gap: 3 μm) upon cooling. **2‐Fla‐1** in a single side rubbed nylon cell, cell gap: 1.6 μm) at e) 135 °C and f) 110 °C.

Flavylium salt **2‐Fla‐1** with two dodecyloxy chains at the A ring and one chain at the B ring showed three transitions upon heating in the DSC. An endothermal melting transition at 46 and 100 °C followed by a first order transition at 126 °C with a small transition (less than 0.7 kJ mol^−1^) and a clearing transition at 145 °C was detected (Table S1, Figure S4 a, Supporting Information). Under the POM **2‐Fla‐1** showed filament‐like textures in the highest mesophase upon heating (Figure 5 a). Cooling from the isotropic liquid resulted in a pronounced homeotropic alignment, interrupted by occasionally occurring Maltesian crosses. Upon entering the lower‐temperature mesophase fan‐shaped textures were observed (Figure 5 b). In a polyimide coated cell, already the higher‐temperature phase showed fan‐shaped textures, characteristic for the SmA phase (Figure 5 c). The lower‐temperature phase showed broken fan‐shaped textures characteristic for the SmC phase (Figure 5 d). Additionally, the birefringence increases drastically. However, attempts to determine optical tilt angles in both polyamide and nylon test cells failed despite good alignment, because the lower‐temperature phase appears to be uniaxial, disproving a SmC phase. Cooling into the lowest temperature phase resulted in grainy and darkened textures.

**Figure 6:**
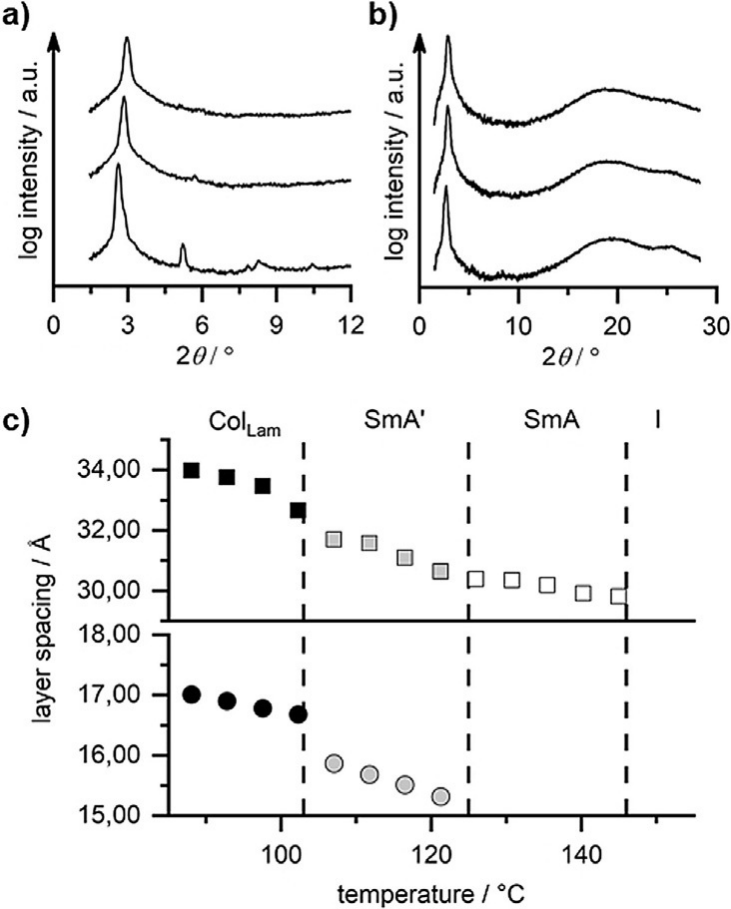
a) SAXS and b) WAXS patterns of **2‐Fla‐1** at 130 °C, 120 °C and 90 °C (from top to bottom). b) Temperature dependent layer spacing of the 001 and 002 reflex on cooling from the isotropic state. Transition temperatures are given by the dashed lines. c) Temperature‐dependent layer spacing of the (001) reflex (▪) and the (002) reflex (•) in the Lam_Col_ phase (filled symbols), the SmA′ phase (gray symbols) and the SmA phase (hollow symbols).

To gain further insight into the observed phases, **2‐Fla‐1** was examined by temperature‐dependent XRD measurements. In the small‐angle region at 130 °C, a sharp reflex indexed as (001) of the SmA phase is observed (Figure 6 a, Figure S11, Supporting Information). The layer spacing of 31.1 Å is smaller than the molecular length due to interdigitation of the alkoxy side chains, as also seen in the SmA phase of **V‐Fla‐1**. The wide‐angle region displayed a diffuse halo at 4.75 Å oriented perpendicular to the layer reflex (Figure 6 b). Therefore, this phase has been identified as a partially interdigitated SmA phase.

The mesophase at 120 °C showed besides the (001) reflex the higher ordered reflex (002). The *χ* scan of the wide‐angle region of the oriented sample showed that the reflexes oriented perpendicular to the layer reflexes and no significant difference towards the SmA phase could be observed. However, the reflex corresponding the intramolecular distance is more prominent in this phase indicating a longer correlation length of the lateral intermolecular distance. The temperature dependent SAXS measurement revealed, that the layer spacing decreased continuously with increasing temperature from 102 to 145 °C (Figure 6 c). Considering the results from the POM and the X‐ray analysis, we assume that this phase is a SmA′ phase, with the difference that the layers are built up by flavylium dimers.

The lowest temperature mesophase at 90 °C showed the layer reflexes (001), (002), (003) and (004) along the meridian, as well as the intercolumnar reflex (010) oriented perpendicular to the layer reflexes. Thus, the overall appearance of the XRD pattern showed similarities to the Lam_Col_ phase of **V‐Fla‐1**. The layer spacing of 34.0 Å is larger as compared to **V‐Fla‐1**, showing that interdigitation is less pronounced in **2‐Fla‐1**, due to the additional side chain. The intercolumnar distance of the columns is 10.6 Å and no splitting of this reflex could be detected. The WAXS showed a diffuse halo at 4.60 Å and the intracolumnar reflex at 3.50 Å along the equator.

### Flavylium salts with columnar rectangular phases

**Figure 7:**
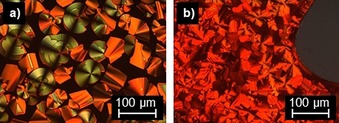
Polarized optical micrographs of a) iV‐Fla‐2 at 196 °C (magnification 200×) and b) **iV‐Fla‐3** at 120 °C (magnification 200×). All pictures were taken by cooling from the isotropic phase with a cooling rate of 5 K min^−1^.

In contrast to the non‐mesomorphic vanillin‐derived flavylium salt **V‐Fla‐2** (melting point: 195 °C), the corresponding isovanillin‐derived flavylium salt **iV‐Fla‐2** and **iV‐Fla‐3** showed Col_ro_ mesophases between 52 and 199 °C and between 50 and 156 °C in the DSC, respectively (Figure S3 c, Supporting Information). Under the POM both compounds displayed spherulite‐like textures and line defects, characteristic for columnar mesophases (Figure 7).

**Figure 8:**
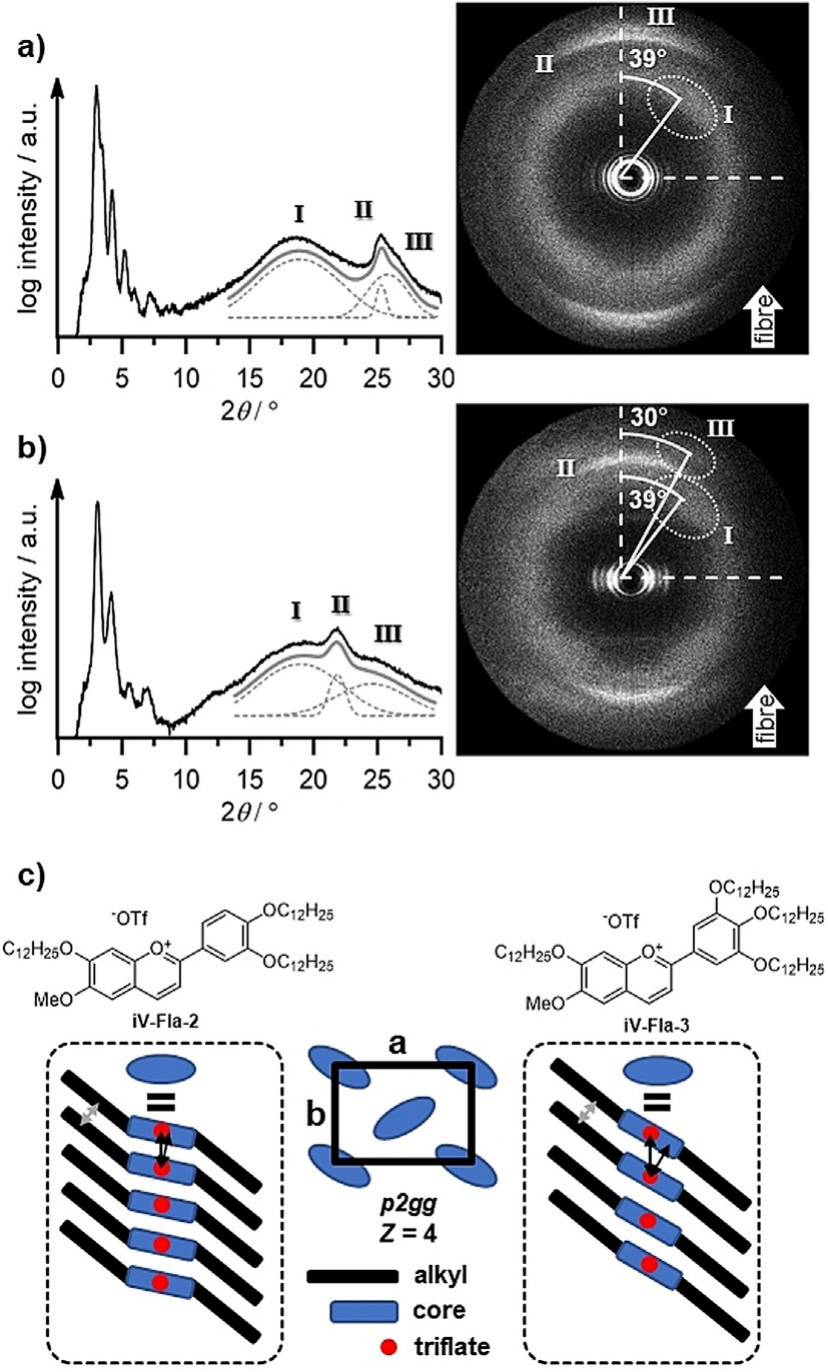
X‐ray diffractograms of a fiber sample of a) **iV‐Fla‐2** at 110 °C and b) **iV‐Fla‐3** at 120 °C with the corresponding 2D diffraction pattern with the azimuthal angles of the diffuse wide‐angle reflexes. The fitting of the wide‐angle area is given in grey with the sum (bold line) of the distinct Gauss peaks (dashed lines). White arrows indicate the direction of the fiber. c) Proposed packing model of the Col_ro_ phase and the stacking within the column (the representation of the discoid has been simplified, for a more detailed discussion see Figure 10 and the corresponding text).

The diffraction pattern of an oriented fiber of **iV‐Fla‐2** consisted of sharp reflexes in the small‐angle region (Figure S12 a, Supporting Information). The reflexes could be assigned as (11), (02), (12), (22), (23) and (31) of a columnar rectangular ordered mesophase Col_ro_ with *p*2*gg* symmetry. The lattice parameters of the elemental cell are *a=*35.9 Å, *b=*50.2 Å and a *Z* value of 4, indicating that one disk is formed by two molecules (it must be noted that a *p*2*mg* symmetry, where one disc consists of one molecule, cannot be completely ruled out). The wide‐angle region of a fiber sample showed a diffuse halo of the molten alkyl chains centered around 4.71 Å. This reflex is split into two reflexes with an azimuthal angle of 39° with respect to the meridian (Figure 8 a). Furthermore, a relatively sharp reflex at 3.52 Å as well as an additional diffuse reflex at 3.44 Å was observed. The diffraction pattern of **iV‐Fla‐3** (Figure S12 b, Supporting Information) showed fewer and less intense higher‐order reflexes compared to **iV‐Fla‐2**, but the same phase geometry of *p2gg* with slightly larger lattice parameters (*a=*36.3 Å, *b=*51.0 Å, *Z=*4) was observed. The wide‐angle region showed also three reflexes: a diffuse halo at 4.63 Å with a tilt of 39°, a relative sharp reflex at 4.06 Å with no tilt as well as an additional diffuse reflex at 3.39 Å with a tilt of 30° with respect to the meridian (Figure 8 b). Furthermore, a reflex is observed at 7.46 Å.

The occurrence of a relative sharp reflex in the wide‐angle region in discotic phases is usually referred to the periodic arrangement of the aromatic cores, but for the flavylium salts with a Col_ro_ mesophase the situation seems to be different, considering that the intracolumnar distance of **iV‐Fla‐3** would be too large for typical π–π stacking. To rationalize the wide angle reflexes I–III, and their different azimuthal angles in Figure 8, the mesogens can be divided into three parts: the alkoxy side chains I, the triflate anion II and the aromatic core III. From the diffraction pattern of the **iV‐Fla‐2** fiber sample the tilt of the mesogens with respect to the column axis is mainly governed by the alkyl chains. The observed tilt angle of 39° can be obtained directly from the azimuthal angle. Presumably the almost spherical anions form the linear, non‐tilted backbone of the columns as depicted in Figure 8 c. The small difference in the distances of the anions (3.52 Å) and cations (3.44 Å) can be explained by the tilt between the aromatic cores of 12° calculated by using the formula *α*=cos^−1^ (d_III/_ d_II_). This angle is quite small and, therefore, could not be determined directly form the diffraction pattern due to overlapping of both reflexes.

For **iV‐Fla‐3**, the tilt of the alkyl chains is similar to **iV‐Fla‐2** but the tilt between the aromatic cores is much higher. Firstly, this becomes noticeable by the splitting of the corresponding reflex III with an azimuthal angle of 30° in the diffraction pattern and, secondly, by the increased anion–anion distance of 4.06 Å as a result of the aromatic tilt whereas the distance between the aromatic cores remains almost identical. By using the above mentioned formula, the calculated tilt of the flavylium cation is 33° and, therefore, in good agreement with the experimental value and supports the assumed packing of the molecules within the column. Further evidence can be found by comparing the width of the peaks in the wide‐angle area. The aromatic reflex in the less tilted **iV‐Fla‐2** is sharper compared to the more tilted **iV‐Fla‐3**. A smaller tilt results in a longer correlation length and therefore in a sharper reflex. The results provide a useful tool to estimate the tilt of the columns in the Col_r_ phases in ILCs for which no planar aligned sample can be obtained. Such detailed information about the intracolumnar stacking can be important, for example, for ionic conductivity.^35^ An comprehensive packing model for the columnar phases of the flavylium salts will be discussed below by using **2‐Fla‐3** as an example.

### Flavylium salts with rectangular and hexagonal phases

**Figure 9:**
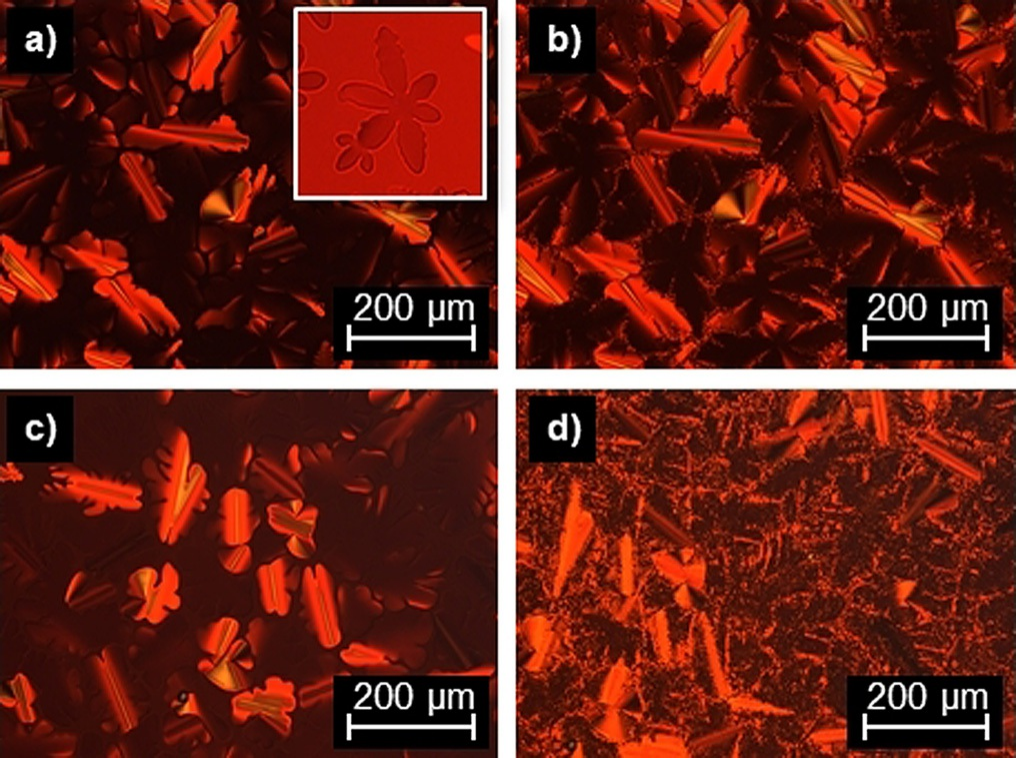
Polarized optical micrographs of **V‐Fla‐3** at a) 125 °C (inset: dendritic germ observed below the clearing point, picture taken with slightly uncrossed polarizers) and b) 75 °C between crossed polarizers. Textures of **2‐Fla‐3** at c) 185 °C and d) 75 °C. All pictures were taken by cooling from the isotropic phase with a cooling rate of 5 K min^−1^ and a magnification of 200×.

The vanillin‐derived flavylium salt **V‐Fla‐3** as well as **2‐Fla‐2** and **2‐Fla‐3** showed a lower temperature Col_ro_ mesophase and a higher temperature Col_h_ mesophase. Flavylium salt **V‐Fla‐3** showed upon heating in the DSC a melting transition at 53 °C, a mesomorphic transition at 100 °C and clearing into the isotropic phase at 190 °C (Figure S2 d, Supporting Information). Under the POM uncharacteristic grainy textures were observed for the low‐temperature phase upon heating, whereas the high‐temperature phase showed line defects. Cooling from the isotropic phase into the high‐temperature mesophase resulted in dendritic growth (Figure 9 a). The hexagonal shape of the liquid crystalline germs indicates a columnar hexagonal mesophase in agreement with Bouligand.^36^ Upon cooling into the low‐temperature phase, the texture became grainy especially in previously homeotropic aligned areas (Figure 9 b).

XRD experiments of **V‐Fla‐3** (Figure S13) in the higher‐temperature mesophase at 140 °C revealed the reflexes (10), (11), (20) and (21) of the Col_h_ mesophase (*p6mm* symmetry) with a lattice parameter of *a=*25.8 Å (*Z=*1). The WAXS region showed a diffuse halo at 4.51 Å and a reflex at 3.39 Å originating from the intracolumnar order. As compared to the Col_r_ phase, the distances between the anions and those between the aromatic cores are identical and therefore are represented by a single reflex at 3.39 Å. Furthermore, a reflex at 6.71 Å was observed, which is twice the layer spacing of the intracolumnar distance. For the further discussion this reflex will be noted as π–π′.

In the lower temperature phase of **V‐Fla‐3** at 75 °C the SAXS region showed the reflexes (01), (10), (12), (20), (03), (13), (22), (04), (30), (24) and (15) which could be assigned to a Col_r_ phase with *p*2*mm* symmetry and lattice parameters of *a=*57.7 Å and *b=*41.9 Å with a *Z* value of 4, indicating that one discoid is formed by 4 flavylium salts. Presumably, the decrease of the alkoxy chain length at 7 position to a methoxy group in **V‐Fla‐3** resulted in an different volume requirement as compared to **iV‐Fla‐2**, **iV‐Fla‐3** and **2‐Fla‐3** carrying a dodecyloxy chain at this position. The WAXS region showed a similar behavior as discussed above for **iV‐Fla‐3**. The diffuse halo centered around 4.52 Å showed a splitting into two reflexes with an angle of 51° with respect to the meridian corresponding to the average distance and tilt of the alkyl chains. The sharp reflex at 3.96 Å showed no tilt, as it originates from the non‐tilted anions as seen above. The reflexes corresponding to the distances of the aromatic cores at 6.74 and 3.64 Å showed both a tilt of 23° respectively and are in agreement with the calculated tilt *α*=cos^−1^ (3.64 Å/3.96 Å)=23°.

The DSC of flavylium salt **2‐Fla‐2** with two dodecyloxy side chains at the A and B ring showed a transition at 119 and 131 °C into the mesophase as well as a mesomorphic transition at 165 °C with low enthalpy (−0.8 kJ mol^−1^) followed by clearing into the isotropic liquid phase at 180 °C (Figure S4 b, Supporting Information). Under the POM the compound displayed spherulite‐like textures and dendritic growth typical for columnar phases. The small angle diffractogram of compound **2‐Fla‐2** at 170 °C showed a single (10) reflex of a hexagonal mesophase with a lattice parameter of *a=*35.5 Å (*Z=*2, Figure S14, Supporting Information). The WAXS pattern showed a diffuse halo at 4.68 Å and an additional intracolumnar reflex at 3.49 Å. In contrast to the hexagonal phases of **V‐Fla‐3**, **2‐Fla‐3**, **3‐Fla‐2** and **3‐Fla‐3** the π–π′ reflex could not be observed. As seen for the previous flavylium compounds **iV‐Fla‐2** and **iV‐Fla‐3**, the lower temperature Col_r_ phase shows *p*2*gg* symmetry with a lattice parameter of *a=*61.0 Å and *b=*34.5 Å, preserving the pseudohexagonal lattice of the higher temperature hexagonal phase as indicated by the ratio *a*/*b=*3^1/2^.

For the compound **2‐Fla‐3** upon heating a glass transition at 59 °C, a mesophase‐to‐mesophase transition at 102 °C and a clearing into the isotropic liquid at 200 °C was observed. In analogy to **V‐Fla‐3**, the higher‐temperature mesophase showed typical columnar textures, which became grainy upon entrance into the lower‐temperature mesophase (Figure 9 c,d).

From the XRD result for **2‐Fla‐3** a slightly larger lattice parameter of *a=*27.4 Å in the higher‐temperature Col_ho_ phase and a halo at 4.52 Å as well as the intracolumnar reflexes at 3.44 Å and 6.75 Å were observed (Figure S15 a,b, Supporting Information). The small angle peaks of the lower‐temperature mesophase could be perfectly indexed as hexagonal phase with *a=*35.4 Å (*Z=*2), but since the wide‐angle region indicates a tilt similar to **V‐Fla‐3**, we assume a Col_r_ phase.

Proper indexation could be achieved for the *hk* sets: 20/02 and 11/31 both with *p*2*gg* symmetry and a lattice parameter of *a=*61.3 Å and *b=*35.3 Å, as well as the rather unlikely case of 10/01 with *p*2*mm* symmetry and a lattice parameter of *a*=30.1 Å and *b*=17.7 Å. The wide‐angle region displayed a halo with a layer spacing of 4.52 Å and a tilt angle of 51°, resulting from the alkyl chains. The layer spacing of the anions is with a value of 3.96 Å larger than the π–π distance of the aromatic cores (3.55 Å). The tilt of these cores was determined to be of 23° by fitting of the *χ* scan (calculated: 25°).

**Figure 10:**
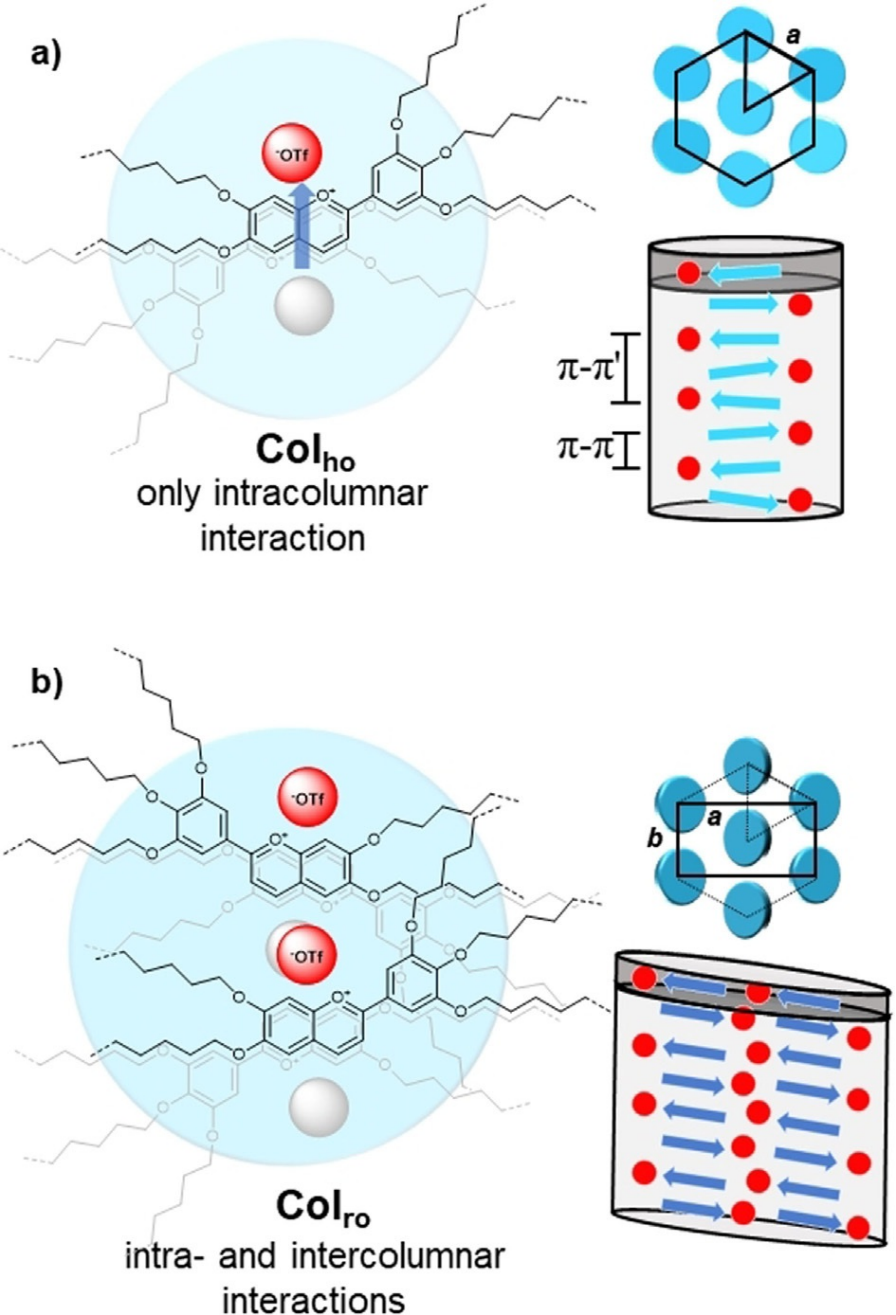
Proposed packing model of the flavylium salts in the a) Col_ho_ and the b) Col_ro_ mesophase viewed from the top (left) and in an side view (right).The flavylium cations are displayed as blue arrows (pointing towards the oxonium cation) and the triflate anions as red dots.

To construct general packing models for columnar phases, usually nanophase segregation and π–π interactions are decisive. However, in the case of flavyliums salt the additional repulsive charge interaction located in the center of the aromatic core plays the major role. Therefore, most likely flavylium salts are stacked into columns in an antiparallel arrangement (Figure 10 a) similar to the crystal structure of **V‐Fla‐1** (Figure 1). Further evidence for this model can be found in the observed π–π′ reflex, which corresponds to either the distance between the aromatic core with the same direction or the distance between the anions. This model can be applied to all compounds with a Col_ho_ phase with one molecule per disc (e.g., **V‐Fla‐3**, **2‐Fla‐3**, **3‐Fla‐2**, **3‐Fla‐3**).

In the Col_ro_ phases, the XRD results revealed that two flavylium salts form one discoid. We assume that in addition to the intracolumnar interaction in the lower temperature Col_ro_ also intercolumnar anion–H bonds occur. In other words, the Col_ro_ phase can be considered as two antiparallel packed columns bond together, as depicted in Figure 10 b. Within one discoid the flavylium salts can be arranged either in a face‐to‐face manner or pointing into same direction. In both cases, the flexible B ring has to rotate out of plane to avoid steric repulsion. We assume that additional intercolumnar anion–H interactions stabilize the lower‐temperature Col_ro_ phase, whereas the higher‐temperature Col_ho_ phase features only intramolecular interactions. **2‐Fla‐2** can be as an intermediate case which forms a Col_ho_ but contains two molecules per disc.

### Flavylium salts with hexagonal phases

Flavylium salt **3‐Fla‐2** possessing three dodecyloxy chains at the A ring and two at the B ring showed a single broad mesophase between −6 °C and 215 °C in the DSC (Figure S5 c, Supporting Information) and characteristic columnar textures under the POM. The XRD (Figure S16 a,b, Supporting Information) experiments revealed a Col_ho_ mesophase with a slightly larger *a* value of 28.6 Å (*Z=*1) as compared to the analogue **2‐Fla‐3** with an inverted substitution pattern. The wide‐angle region displayed the diffuse halo, as well as the π–π and the π–π′ reflex. Compound **3‐Fla‐3** showed also a Col_ho_ phase, ranging from 56 and 209 °C with similar textures and XRD result (for details see Table S2 and Figure S16 c,d, Supporting Information). No Col_ro_ phase was observed for the substitution pattern **3‐Fla‐B**, presumably due to steric overcrowding in the above mentioned packing models shown in Figure 10. Additionally, the overall number of alkoxy side chains can be sufficient to stabilize the Col_ho_ phase over a wide temperature range.

In contrast to the normal thermotropic behavior of **3‐Fla‐2** and **3‐Fla‐3**, the flavylium salt **3‐Fla‐1** lacked a stable mesophase. Upon heating from the solid phase, clearing into the isotropic phase was detected at 54 °C by DSC and under the POM (Figure S5 a, Supporting Information). Upon further heating no other peak could be observed. The cooling curve showed a broad endothermic peak at 20 °C and a sharp peak at 11 °C. These two transitions were enantiotropic and are also observed in the second and third heating. Surprisingly, under the POM (Figure S17, Supporting Information) the formation of columnar textures could be observed upon heating the substance to 78 °C. These textures were only observable in thin sample areas between glass or in a polyimide‐coated test cell of 4.6 μm thickness, whereas thick parts of the sample remain isotropic. At 143 °C these textures cleared into a second isotropic state. Upon cooling again typical columnar textures was observed clearing into a fluidic phase at about 78 °C, revealing the enantiotropic nature of this phase. This shows that under bulk conditions the compound **3‐Fla‐1** has no stable mesophase, but under planar anchoring liquid crystalline properties can be observed. Reentrant phases have already been reported for compounds in which two SmA phases were separated by a nematic phase, but the sequence I_re_‐Col‐I is quite rare^37^, ^38^, ^39^, ^40^ and, to the best of our knowledge, an I_re_ phase has never been reported for thermotropic ionic liquid crystals.

**Figure 11:**
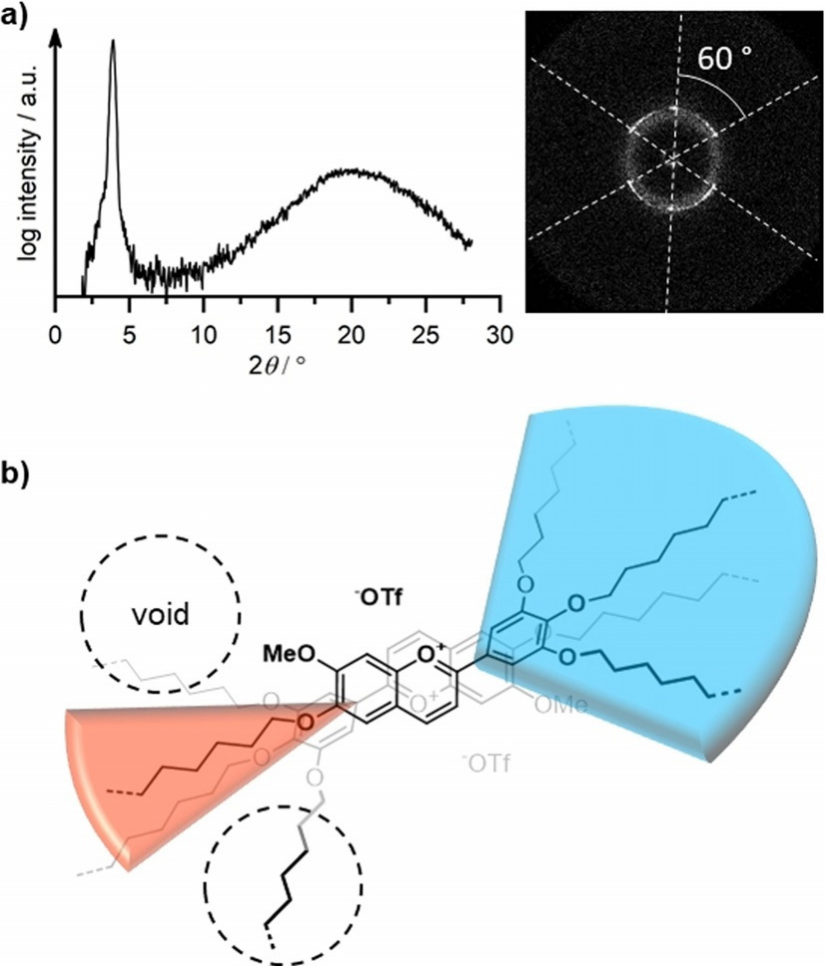
a) WAXS diffractogram of **3‐Fla‐1** at 85 °C. The inset displays the SAXS pattern of a planar aligned domain obtained by slow heating into the Col_h_ phase at 80 °C (the additional diffuse reflex is most likely due passing of the beam through another domain without mesophase). b) Proposed stacking of two flavylium cations **V‐Fla‐3** in the liquid crystalline state.

XRD experiments showed a (10) and (11) reflex in the small angle region and a diffuse halo in the WAXS measurement (Figure 11 a). Considering that no π–π reflex is observed, the intracolumnar order is quite low in contrast to the previously described columnar mesophase. Fortunately, a partially oriented SAXS pattern of **3‐Fla‐1** could be obtained by slowly heating the sample into the columnar phase from the isotropic re‐entrant phase. The diffraction pattern consisted of a diffuse part and sharp reflexes orientated in a hexagonal manner (Figure 11 b).

We assume that the unusual phase sequence can be explained by a complex relationship of intermolecular forces, surface interactions, and space filling. The high‐temperature isotropic phase probably consists of monomeric flavylium salts, whereas the surface is covered by molecules due to polar anchoring. Cooling the sample causes more and more molecules to assemble on the surface aligned molecules by intramolecular interactions, leading to the formation of columns which ultimately results in the observed Col_h_ phase. With decreasing temperature, the attractive π–π and ionic interactions increase. At some point these interactions become so strong that stable dimers of **3‐Fla‐1** are formed, which no longer display any liquid crystalline behavior, resulting in the phase transition into the lower‐temperature I_re_ phase.

This behavior can be explained by comparing **3‐Fla‐1** to **V‐Fla‐3**, with the inverted substitution pattern. In **V‐Fla‐3**, the higher number of side chains is attached to the flexible B ring. Torsion of this phenyl moiety causes the side chains in *m*‐position to rotate out of the aromatic plane and extend into the upper and lower mesogen (Figure 11 b). This allows the mesogen to effectively fill the void left by the mono‐substituted side. Additionally, the rotation of the crowded B ring disfavors the formation of a dimeric species by steric repulsion. In contrast, the higher substituted side in **3‐Fla‐1** is rigid, therefore space filling is only possible by strong coiling of the alkyl chains, leading to a rather spherical appearance, disfavoring liquid crystalline properties.

### Flavylium salts with 3′‐substitution pattern

Considering that several of the above discussed flavylium salts showed quite high clearing points, we wanted to push the clearing temperature towards lower temperatures. A lot of research has been done with respect to reduce the clearing point by using thioether side chains,^41^ branched^42^ or swallow‐tailed^43^ side chains rather than linear alkoxy side chains. In contrast, our aim was to reduce the clearing point by varying the substitution pattern at the flavylium A and B rings. Therefore the 3′‐substitution pattern, derived from 2,3,4‐dodecylalcoxybenzaldehyde **5 e** at the A and/or B ring was tested.

The clearing points of the 3′‐mesogens, that is, **A‐Fla‐3′** and **3′‐Fla‐B** could indeed be reduced compared to their **A‐Fla‐3** and **3‐Fla‐B** counterparts. This effect is more pronounced for derivatives with the 3′‐substituent attached on flexible B ring. For example, **3‐Fla‐3** shows a clearing point at 210 °C and strong decomposition in the DSC, **3′‐Fla‐3** enters the isotropic phase at 169 °C and **3‐Fla‐3′** already at 93 °C. **3′‐Fla‐3** showed only minor differences compared to **3‐Fla‐3′**.

All compounds of this series **A‐Fla‐3′** and **3′‐Fla‐B**, except the non‐mesomorphic **V‐Fla‐3′** and **iV‐Fla‐3′** derivatives exhibited a columnar mesophase as indicated by the typical columnar textures observed under the POM (Figure S18, Supporting Information). In contrast to the **A‐Fla‐3** and **3‐Fla‐B** flavylium salts, the columnar phases shows almost no intracolumnar order. Similar lattice parameters were found for compound **2‐Fla‐3′** (*a=*27.2 Å), **3‐Fla‐3′** (*a=*27.7 Å) and **3′‐Fla‐3′** (*a=*29.4 Å) and the calculated *Z*‐value of 1 indicating one molecule per discoid (Figure S19, Supporting Information). The intercolumnar reflex of **3′‐Fla‐3′** is broad, almost comparable to the isotropic liquid. This behavior can be explained by either a very small correlation length of the intercolumnar reflex, or that the mesophase is only stable under planar anchoring conditions, similar to **3‐Fla‐1**. This would also explain the missing clearing point in the DSCs. In contrast, the derivatives **3′‐Fla‐1** and **3′‐Fla‐2** showed larger *a* values with 29.7 Å and 31.4 Å respectively and a *Z* value of 2 indicating two molecules per disk. **3′‐Fla‐3** again shows a *Z*‐value of 1 and a lattice parameter of 27.7 Å. The packing model shown in Figure 10 cannot easily be transferred to these compounds, because the columnar phases are disordered and the alkoxy substituent in position 8 causes a steric hindrance.

### Dye properties

**Figure 12:**
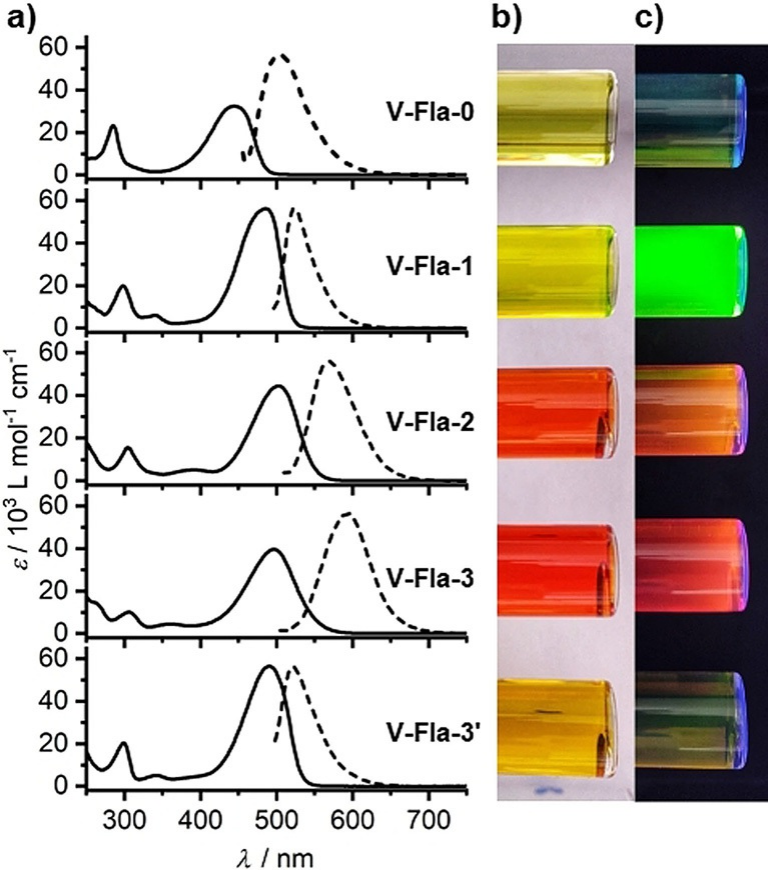
a) Absorption spectra (bold line, represented by its extinction coefficient determined by linear regression of a concentration series ranging from 0.2×10^−5^ m to 7×10^−5^ m) and normalized emission spectra (dashed line) of vanillin‐derived flavylium salts **V‐Fla‐B** in CHCl_3_. Solutions of the corresponding flavylium salt under b) daylight and c) UV‐radiation (366 nm).

To obtain insight into the optical properties of the flavylium salts **A‐Fla‐B** were examined by UV/Vis absorption and emission spectroscopy. Chloroform was chosen as the solvent, because all compounds showed good solubility in halogenated solvents. The results are summarized in Table S3 (Supporting Information). All compounds showed absorption maxima between 444 and 507 nm. The spectra of the vanillin‐derived series **V‐Fla‐B** are shown in Figure 12. The substitution pattern on the A ring had only a minor influence (<4 nm) on the absorption maximum, whereas some variations of the extinction coefficients were observed. However, the number and position of substituents at the B ring resulted in a red‐shift of the absorption maximum, which increased in the series **V‐Fla‐0**<**V‐Fla‐1**<**V‐Fla‐3′**<**V‐Fla‐3**<**V‐Fla‐2**.

Fluorescence spectra were also measured in CHCl_3_ by exciting the molecules in correspondence of their absorption maxima. The flavylium salts showed emission maxima between 498–595 nm. The Stokes shift increased in the series **V‐Fla‐1** (1467 cm^−1^)<**V‐Fla‐2** (2416 cm^−1^)<**V‐Fla‐0** (2721 cm^−1^) and **V‐Fla‐3** (3298 cm^−1^). Interestingly, the **V‐Fla‐3′** derivative showed a comparable small Stokes shift (1467 cm^−1^) as observed for **V‐Fla‐1** but the fluorescence intensity was low. The number of alkoxy substituents on the flavylium salt had a strong impact on the absolute fluorescence quantum yields. Although **V‐Fla‐0** with no substituent on the B ring was only weakly emissive (*Φ*_F_=4 %), the corresponding analogue **V‐Fla‐1** with one substituent on the B ring displayed a very strong green emission with a quantum yield of 97 %. Similar high values have already been reported by Haucke for 7,4′‐dimethoxy substituted flavylium salts.^44^ Upon further increasing the number of side chains in the B ring, the quantum yield decreased considerably (**V‐Fla‐2**: 5 %). Flavylium salts with three alkoxy side chains were only weakly emissive (**V‐Fla‐3**:<1 %, **V‐Fla‐3′**:<1 %). With increasing the number of alkoxy side chains at the A ring the quantum yields also decreased. Although vanillin‐derived flavylium salt **V‐Fla‐1** and isovanillin‐derived flavylium salt **iV‐Fla‐1** showed almost quantitative fluorescence (97 and 99 %, respectively), already **2‐Fla‐1** showed a reduced value of 92 %. With three alkoxy side chains in **3‐Fla‐1** the quantum yields drastically dropped below 1 %, as seen for **3‐Fla‐1** and **3′‐Fla‐1**. Possible fluorescence quenching resulting from H aggregates can be clearly ruled out due to the low concentration and the fact that no blueshift of absorption and emission within the series **V‐Fla‐1**, **iV‐Fla‐1**, **2‐Fla‐1**, **3‐Fla‐1** and **3′‐Fla‐1** was observed.

The fluorescence quantum yields seem to strongly depend on the number of alkoxy chains at the B ring. The high emission of **V‐Fla‐1**, **iV‐Fla‐1** and **2‐Fla‐1**, which bear only one alkoxy chain at the B ring as compared to the only weakly fluorescent flavylium salts with either no substituent on the B ring (i.e. **V‐Fla‐0**, **iV‐Fla‐0**, **2‐Fla‐0**) or two alkoxy chains on the B ring (i.e. **V‐Fla‐2**, **iV‐Fla‐2**, **2‐Fla‐2**), might be rationalized by considering the canonical Lewis structures. As exemplified for flavylium salts **V‐Fla‐0**, **V‐Fla‐1** and **V‐Fla‐2** possible Lewis structures are shown in Figure S24 (Supporting Information). According to a previous TD‐DFT study by Woodford^45^ in the parent flavylium salt **V‐Fla‐0 (M0)** carrying an unsubstituted phenyl B ring two Lewis structures **V‐Fla‐0 (M1)** and **V‐Fla‐0 (M2)** with the positive charge located at C‐2 or C‐4 are favored over Lewis structure **V‐Fla‐0 (M0)** with the positive charge at O‐1. Thus, the phenyl ring possesses a high degree of rotational freedom resulting in decreased fluorescence quantum yields. In contrast, the presence of a *para*‐alkoxy group attached to the B ring in **V‐Fla‐1** stabilizes the positive charge at C‐2 (or C‐4) through conjugation, resulting in Lewis structure **V‐Fla‐1 (M3)**. This Lewis structure seems to be preferred due to the extended π system. Thus, conjugation of the B ring leads to planarization and rigidification resulting in strong fluorescence emission. Further evidence for the rigidification can be found in the asymmetric peak shape of **V‐Fla‐1**, as a result of a similar geometric of the ground and excited state.

When a second alkoxy group is present in the B ring as in **V‐Fla‐2**, the contribution of the Lewis structure **V‐Fla‐2 (M3)** to the overall electronic structure is diminished as compared to **V‐Fla‐1 (M3)** because the electron‐donating +M effect of the *para*‐alkoxy group is partially counterbalanced by the electron‐withdrawing −I effect of the *meta*‐alkoxy group. This increases the single bond character of the C2‐C1′ bond, resulting in an increased rotational mobility and thus decreased fluorescence quantum yield. This effect becomes even more pronounced in **V‐Fla‐3**, which shows a fluorescence quantum yield below 1 %. These hypotheses were further supported by preliminary DFT calculations (for details see Supporting Information) with simplified flavylium salts (alkoxy groups were replaced by methoxy), which indicated an elongation of the C2−C1′ bond by 6 pm for **V‐Fla‐2** upon transition from the ground state to the excited state, whereas the C2−C1′ bond lengths remained almost constant for **V‐Fla‐0** and **V‐Fla‐1**. Furthermore, the calculated oscillator strengths of **V‐Fla‐1** was twice as large as compared to **V‐Fla‐0**, **V‐Fla‐2**. A previous computational study by Quina has shown that calculations on hydroxylated flavylium salts are challenging.^46^ Therefore, our results should be treated with care. However, the above proposed model serves as a useful rationale for the experimental results.

The lifetime of the excited state in solution was examined by TRSPC (time resolved single photon counting). The monosubstitution on the B ring, that is, **V‐Fla‐1**, **iV‐Fla‐1**, and **2‐Fla‐1**, resulted in monoexponential emission decays with lifetimes around 3.3 ns (Table S3, Figure S20, Supporting Information). For the unsymmetrical substitution with two alkoxy side chains on the B ring, the emission decay profiles could be fitted with two components with shorter lifetimes, revealing that two emitting species are involved in the fluorescence process. A potential reason for the two decay times might be the formation of hemiketals and chalcones, which is known for flavylium salts.^20^ The two decay times may also be caused by the presence of rotamers with intact π system.

**Figure 13:**
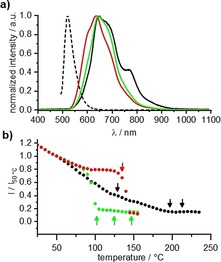
a) Emission spectra of **V‐Fla‐1** (black), **iV‐Fla‐1** (red) and **2‐Fla‐1** (green) in the solid state at 50 °C under irradiation with UV light (350–380 nm, solution spectra of **V‐Fla‐1** is given in dashed lines for comparison) and b) temperature‐dependent emission intensity at 665 nm upon cooling the sample from the isotropic liquid (cooling rate of 10 K min^−1^). For the orientation transition temperatures of the DSC are given as arrows.

In the solid state only compounds **V‐Fla‐1**, **iV‐Fla‐1**, and **2‐Fla‐1** showed enough fluorescence intensity to obtain reliable data. The emission spectrum of **V‐Fla‐1** was broad and ranged from about 550–850 nm at room temperature with the main contribution at 645 nm (Figure 13, Figure S21, Supporting Information). Additionally, the spectrum of **V‐Fla‐1** was the only spectrum which showed a contribution at about 770 nm, close to the NIR regime. The compound **iV‐Fla‐1** and **2‐Fla‐1** showed similar emission spectra but with different contributions of the single bands. In the solid state, the emission spectra were red shifted compared to the emission in solution (*λ*_max_=484 nm). Temperature‐dependent measurements were performed upon cooling the sample from the isotropic phase. With decreasing temperature, the emission intensity increased, and a red shift was detected. For the compound **V‐Fla‐1** the emission intensity is lowest in the isotropic state and remains low in the SmA phase. Upon entering the Lam_Col_ the intensity increased with a slow slope which further increased in the solid state. For **iV‐Fla‐1** a similar behavior has been observed, with the difference that the intensity increased abruptly upon entering the Lam_Col_ mesophase, in which the luminescence intensity remained almost constant over the whole phase. In the solid state, the intensity increased as expected. Compound **2‐Fla‐1** showed low intensity in the isotropic state and in the smectic phases. Upon entering the Lam_Col_ phase, the intensity increased in the same manner as **iV‐Fla‐1** and **iV‐Fla‐1** in the crystalline state. Hence, emission intensity is strongly dependent on the emitter supramolecular organization. Although the liquid phase and mesophases with pronounced fluidity, that is, the SmA and SmA′ phase, show only weak luminescence, the higher ordered phases, that is, Lam_Col_ and solid phase, show higher fluorescence.

**Figure 14:**
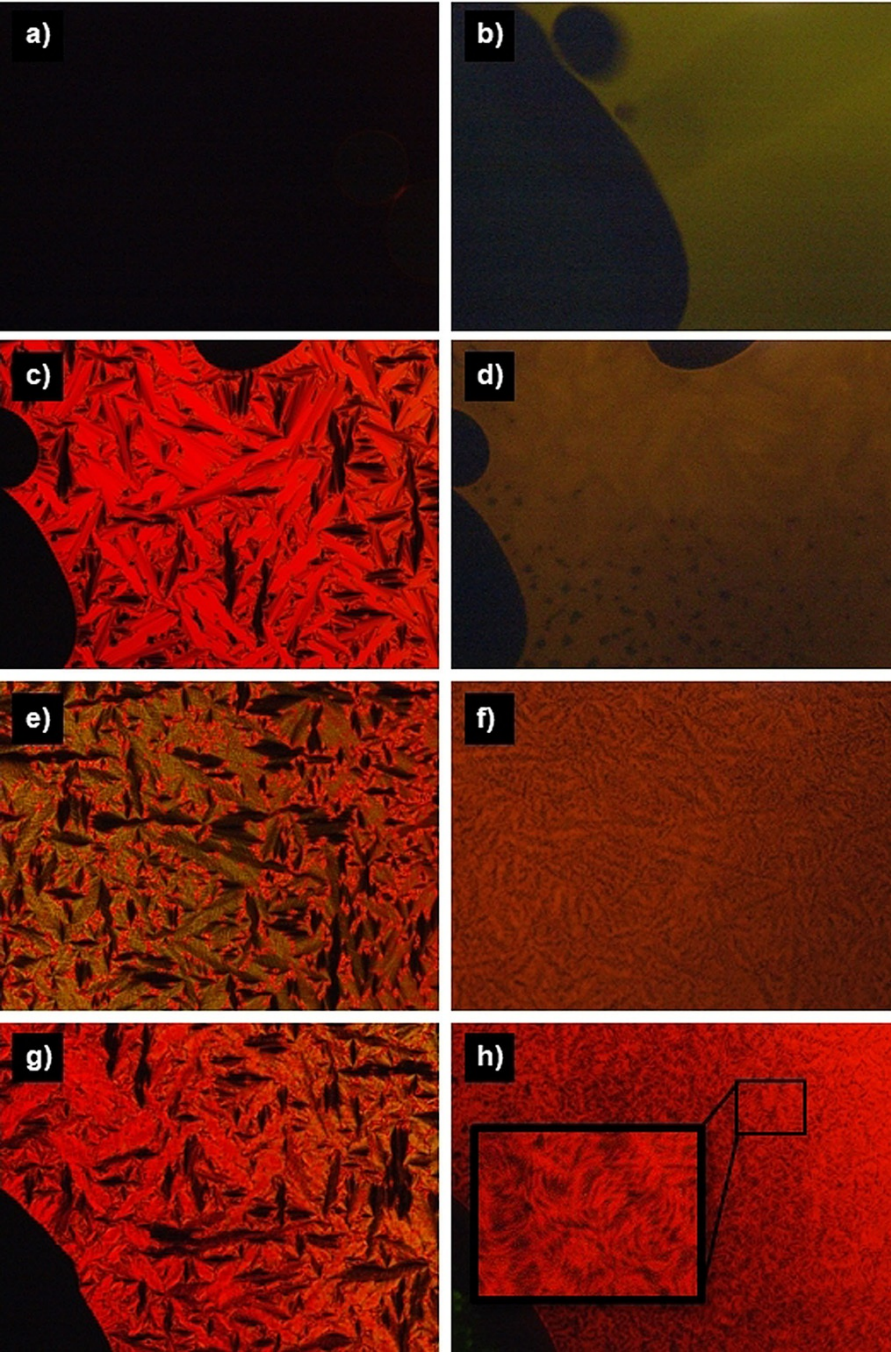
Optical micrographs of **V‐Fla‐1** observed between crossed polarizers (left column) and under UV radiation (right column, without polarizer, exposure time: 8 seconds, the brightness of b) and d) has been increased by 40 % to ensure visibility) in the a–b) isotropic phase at 230 °C, c‐d) the SmA phase at 200 °C, e–f) the Lam_Col_ phase at 170 °C and g–h) the crystalline phase at 30 °C. The images were obtained by cooling of the isotropic liquid with a cooling rate of 10 K min^−1^.

When optical micrographs of **V‐Fla‐1** were examined under UV light, different behaviors were observed depending on the temperature. In the isotropic phase, the intensity of the emitted light appears evenly distributed over the sample area (Figure 14), whereas, already in the SmA phase, this intensity was inhomogeneous. Upon decreasing the temperature, the contrast between bright and dark areas increased. Considering that fluorescence is anisotropic, the emission intensity depends on the orientation of the chromophore.^47^ For compounds **iV‐Fla‐1** and **2‐Fla‐1**, a similar behavior was observed (Figures S22 and 23, Supporting Information).

## Conclusions

Mesogens based on nitrogen cations are dominating the world of ionic liquid crystals and are therefore well explored. In this work, we have shown that the flavylium backbone provides a new functional mesogenic core for ILCs with interesting mesomorphic properties and outstanding emissive behavior.

The flavylium salts have been synthesized according to a modular principle by the condensation of a phenol derivative and an ethynyl ketone building block. Depending on the substitution pattern, various types of mesophases formed. The calamitic shaped molecules **V‐Fla‐1**, **iV‐Fla‐1**, and **2‐Fla‐1** formed lamellar phases (SmA, SmA′, Lam_Col_), the higher substituted flavylium salts displayed discotic mesophases (Col_ho_, Col_hd_, and Col_ro_). The mesophase widths varied within ranges from 13 K (SmA phase of **V‐Fla‐1**) up to 220 K for the Col_ho_ phase of **3‐Fla‐2**, which also displays liquid crystallinity at room temperature. We found that the observed mesophases are governed by the strong π–π and ionic interactions resulting in an antiparallel stacking of the flavylium cation within the columns. A general feature of these flavylium salts is the strong tendency for alignment, allowing the preparation of fiber samples, granting detailed insight into the mesophase by X‐ray diffraction. Special attention was given to the wide‐angle region, providing detailed information of the intracolumnar stacking, which is of interest for future applications of ILCs. In the rectangular phases, the triflate anion provided the linear backbone of the column, whereas the tilt of the aromatic core ranges from 12° **iV‐Fla‐2** to 33° in **iV‐Fla‐3**. A comprehensive packing model for the discotic phase have been proposed, to enable the design of novel flavylium salts with tailored mesomorphic properties. Furthermore, we were able to report the first I_re_ phase in ionic liquid crystals under polar anchoring conditions for **3‐Fla‐1**.

The flavylium salts show strong absorption in the visible area and the derivatives **A‐Fla‐1** (**A**=**V**, **iV**, **2**) show strong emission with almost quantitative absolute quantum yields as a unique feature of this substitution pattern. According to preliminary calculations for **V‐Fla‐1**, both the ground and excited state seem to remain rather rigid, explaining the spectral shape and the enhanced fluorescence. All other flavylium salts are either non‐rigid in the ground state (like **V‐Fla‐0**) or become nonrigid in the excited state (like **V‐Fla‐2**), which likely leads to additional non‐radiative pathways. These compounds also show weak emissive behavior in the solid state. The loss of luminescence intensity with increasing temperature could be reduced in the Lam_Col_ phase of **iV‐Fla‐1** making it an interesting functional liquid crystal. Further work should demonstrate, how the mesophase behavior can be tailored by variation of the anion.

## Conflict of interest

The authors declare no conflict of interest.

## Supporting information

As a service to our authors and readers, this journal provides supporting information supplied by the authors. Such materials are peer reviewed and may be re‐organized for online delivery, but are not copy‐edited or typeset. Technical support issues arising from supporting information (other than missing files) should be addressed to the authors.

Supplementary
