# Supplementary material for: Flavylium Salts: A Blooming Core for Bioinspired Ionic Liquid Crystals
Source: Chemistry. 2019 Sep 18;25(56):12966–80. doi: 10.1002/chem.201901975 (PMC6856849; doi:10.1002/chem.201901975)
Supplement: Supplementary file 1 — Supplementary [file CHEM-25-12966-s001.pdf]

CHEMISTRY
A European Journal

Supporting Information

Flavylium Salts: A Blooming Core for Bioinspired Ionic Liquid Crystals

Robert Forschner,[a] Jakob Knelles,[a] Korinna Bader,[a] Carsten Müller,[b] Wolfgang Frey,[a] Andreas Köhn,[c] Yann Molard,[d] Frank Giesselmann,*[b] and Sabine Laschat*[a]

chem_201901975_sm_miscellaneous_information.pdf

Materials and Equipment

General Methods

All chemicals were, unless otherwise stated, provided by Sigma Aldrich and used without further purification. Trifluormethanesulfonic acid (HOTf) was used in the *ReagentPlus*® (≥99%) grade from Sigma Aldrich and the acid was only handled in glass ware to avoid contamination by metals. Dry THF was obtained by refluxing the solvent over potassium. The eluents for chromatography petroleum ether (PE, low-boiling) and ethyl acetate (EtOAc) were distilled prior to use.

^{1}H NMR spectra were measured using the Bruker Avance 400, Bruker Avance 500, and Bruker Avance 700 spectrometers at 400 MHz, 500 MHz, and 700 MHz as well as ^{13}C NMR spectra at 76 MHz, 126 MHz, and 176 MHz, respectively. Deuterated chloroform ($CDCl_3$) was used as solvent. The chemical shift δ in ppm (parts per million) refers to tetramethylsilane (TMS). Atom numbering can be different from the IUPAC nomenclature to facilitate better comparability. To assign the signals of the ^{1}H and ^{13}C NMR spectra, COSY, HSQC, HMBC, and NOESY measurements were carried out. FT-IR spectra were measured on a Bruker Vektor 22 with a MKII Golden Gate Single Reflection Diamond ATR. Absorption bands were rounded to integer wavenumbers / cm^{-1} and the absorption intensities were classified as follows: w (weak), m (medium), s (strong), vs (very strong). Mass spectra (MS) and high-resolution mass spectra (HRMS) were measured by electrospray ionisation (ESI) with a Bruker MicrOTOF-Q spectrometer. CHN Analysis was performed using an Elemental Analyzer Model 1106 from Carlo Erba Strumentazione. For thin layer chromatography, silica gel 60 F254 glass plates (layer thickness of 0.25 mm) on aluminium (pore size 60 Å) from Merck were used. Column chromatography was performed using silica gel (particle diameter of 40 – 60 µm) from Fluka.

A polarising optical microscope Olympus BX 50, equipped with a Linkam LTS heating stage, was used. Temperature regulation was carried out with the control units TP93 and LNP from Linkam ($\Delta T = \pm 1$ K). Photographs were saved with a digital camera ColorView from Soft Imaging System using the software analySIS.[1] For differential scanning calorimetry, a DSC822e from the company Mettler Toledo was employed. The substances were analysed in 40 µL sealed aluminium pans. Heating and cooling rates of 10 K min^{-1} were employed. Phase transition temperatures and enthalpies were determined by onset values using the software STARe 7.01.[2]

Measurements of the temperature-dependent X-ray diffraction were performed using a Bruker AXS Nanostar C with a ceramic tube generator (1500 W) having cross-coupled Goebel mirrors providing monochromatic Cu K_{α} radiation (1.5405 Å). Diffraction patterns were recorded with Bruker HI-STAR or VÅNTEC 500 detectors. Calibration was carried out using the diffraction pattern of silver behenate at room temperature. The compounds were examined in sealed glass capillaries from Hilgenberg GmbH (external diameter of 0.7 mm, wall thickness 0.01 mm). Fibre samples were obtained by extrusion of the material at room temperature. Measured values were analysed with the software SAXS from Bruker.[3] The diffraction patterns were further processed using the software Datasqueeze [4], Origin [6] and LCDiXray [7].

For UV/Vis and fluorescence spectroscopy solvents were obtained in spectroscopic grade from the supplier and were not further purified. Solutions of flavylium salts were freshly prepared in order to avoid undesired reactions towards hemiketals and chalcones. For absorption spectra, a Perkin Elmer Lambda 35 spectrometer was employed. Emission spectra were recorded on a Perkin Elmer LS 55 spectrometer using slit lengths of 10 nm. Quantum yield were measured with a Hamamatsu C9920-03 system, equipped with a 150 W xenon lamp, monochromator and PMA-12 detector. Lifetime measurements were realized using a picosecond laser diode (HORIBA Jobin Yvon deltadiode, 375 nm) and a Hamamatsu C10910-25 streak camera mounted with a slow single sweep unit. Signals were integrated on a 30 nm bandwidth. Fits were obtained using origin software and the goodness of fit judged by the reduced χ^2 value and residual plot shape. Polarized Optical Microscopy and temperature dependent emission experiments were performed with a Nikon 80i polarized microscope equipped with a Linkam LTS420 hot stage, a Nikon Intensilight C-HGFI (UV 1 filter, 350 nm $< \lambda_{exc} <$ 380 nm) irradiation source, a Nikon DS-FI2 digital camera and an ocean optics QE65000 photodetector connected by optical fibre.

1 Syntheses

General procedure for the syntheses of alkyloxy-substituted benzaldehydes (6a-e) by Williamson etherification (GP 1)

The arylaldehyde **5a-e** (32.8 mmol for **6a**) and 1-bromododecane (39.3 mmol for **6a**) were dissolved in dimethylformamide (DMF, 100 mL) and K_2CO_3 (98.3 mmol for **6a**) was added. After the suspension was stirred at 80 °C for 3 h water was added, and the aqueous phase was extracted with EtOAc (3 x 100 mL). The combined organic phases were dried over $MgSO_4$. The solvent was removed under reduced pressure and the crude product was coevaporated twice with toluene. The products **6a-e** were used without further purification after drying under vacuum.

General procedure for the syntheses of phenols (7a-f) by acid-catalyzed Dakin oxidation (GP 2)

The arylaldehyde **6a-f** (10.3 mmol for **7a**) was dissolved in a mixture of $CHCl_3$ (50 mL) and MeOH (20 mL). After the addition of H_2O_2 (35% in H_2O, 25.8 mmol for **7a**) and concentrated H_2SO_4 (4.13 for **7a**) the reaction was stirred at room temperature. After 18 h water was added until the formation of two phases. The organic phase was separated, and the aqueous phase was extracted with $CHCl_3$ (2 x 80 mL). The combined organic phases were dried over $MgSO_4$ and the solvent removed under reduced pressure. The residue was purified by column chromatography (PE/EtOAc = 10 : 1).[8]

General procedure for the syntheses of ethynylalcohols (8a-f) by Grignard reaction (GP 3)

In a dried Schlenk flask the arylaldehyde **6a-f** (6.89 mmol) was dissolved in abs. THF (30 mL) and cooled to 0 °C. To the solution was added ethynylmagnesium bromide solution (0.5 M in THF, 8.95 mmol) slowly and the reaction was stirred for additional 3 h at room temperature. After the addition of saturated NH_4Cl solution THF was removed under reduced pressure and the aqueous phase was extracted with EtOAc (3 x 50 mL). After drying over $MgSO_4$ and the solvent was removed under reduced pressure and the product was used without further purification.[9]

General procedure for the syntheses of ethynylketones (9a-f) by oxidation of ethylnylalcohols (8a-f) (GP 4)

The ethynylalcohol **8a-f** (6.32 mmol for **8a**) was dissolved in EtOAc (20 mL) and IBX [10] (12.6 mmol) was added at room temperature. The suspension was stirred at 80 °C for 18 h.

After cooling to room temperature, the mixture was filtrated over Celite® and the filtration cake was washed with two additional portions of EtOAc. After evaporation of the solvent the crude product was purified by column chromatography (PE/EE = 20 : 1).[9]

General procedure for the syntheses of flavylium salts (A-Fla-B) (GP 5)

To a solution of phenol **7a-f** (162 µmol for **V-Fla-1**) and ethynylketone **9a-f** (162 µmol for **V-Fla-1**) in EtOAc (5 mL) an excess of triflic acid (600 µmol) was added and stirred for 18 h at room temperature. The product precipitated and was recrystallized directly from the reaction solution. For products with high solubility at room temperature the reaction mixture was stored at 4 °C or -18 °C until the product precipitated. Then 2 mL of absolute EtOH (*p.a.* grade) was added, the mixture was filtered and the obtained solid was washed with EtOH. The obtained product was dryed in a desiccator over phosphorous pentoxide for 3 days.[9]

1.1 Syntheses of arylaldehydes (6a-f)

4-(Dodecyloxy)benzaldehyde (6a)

Synthesis according to GP 1: 4-hydroxybenzaldehyde (4.00 g, 32.8 mmol), K_2CO_3 (13.6 g, 98.3 mmol), 1-bromododecane (9.80 g, 39.3 mmol), yield: 8.71 g, 30.0 mmol, 92%, colourless solid.

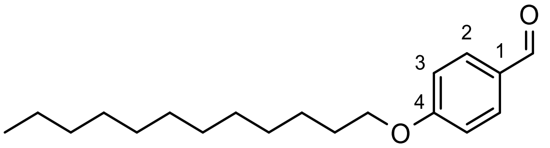

6a
$C_{19}H_{30}O_2$
(290.45)

^{1}H NMR (300 MHz, $CDCl_3$): δ = 0.84–0.93 (m, 3H, CH_3), 1.20–1.55 (m, 18H, CH_2), 1.75-1.87 (m, 2H, OCH_2CH_2), 4.04 (t, J = 6.6 Hz, 2H, OCH_2CH_2), 6.99 (d, J = 8.8 Hz, 2H, 3-H), 7.82 (d, J = 8.8 Hz, 2H, 2-H), 9.88 (s, 1H, CHO) ppm.

^{13}C NMR (75 MHz, $CDCl_3$) δ = 14.13 (CH_3), 22.70, 25.96, 29.06, 29.35, 29.55, 29.59, 29.64, 29.66, 31.92 (CH_2), 68.44 (OCH_2), 114.75 (C-3), 129.74 (C-1), 131.99 (C-2), 164.28 (C-4), 190.82 (CHO) ppm. The spectroscopic data were in accordance with those in the literature. [11]

4-(Dodecyloxy)-3-methoxybenzaldehyde (6b)

Synthesis according to GP 1: 4-hydroxy-3-methoxybenzaldehyde (5.00 g, 32.3 mmol), K_2CO_3 (9.10 g, 65.7 mmol), 1-bromododecane (9.01 g, 36.15 mmol), yield: 10.2 g, 31.8 mmol, 97%, colourless solid.

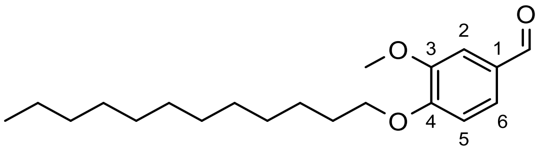

6b
$C_{20}H_{32}O_3$
(320.47)

^{1}H NMR (400 MHz, $CDCl_3$): δ = 0.84–0.92 (m, 3H, CH_2CH_3), 1.19–1.52 (m, 18H, CH_2), 1.83–1.93 (m, 2H, OCH_2CH_2), 3.93 (s, 3H, OCH_3), 4.10 (t, J = 6.9 Hz, 2H, OCH_2CH_2), 6.96 (d, J = 8.1 Hz, 1H, 5-H), 7.41 (d, J = 1.9 Hz, 1H, 2-H), 7.43 (dd, J = 8.1 Hz, 1.9 Hz, 1H, 6-H), 9.85 (s, 1H) ppm. ^{13}C NMR (101 MHz, $CDCl_3$) δ = 14.12 (CH_2CH_3), 22.69, 25.90, 28.92, 29.35, 29.53, 29.58, 29.64, 29.66, 31.92 (CH_2), 56.04 (OCH_3), 69.21 (OCH_2), 109.26, 111.37 (C-2, C-5), 126.80 (C-6), 129.87 (C-1), 149.86, 154.21 (C-3, C-4), 190.89 (CHO) ppm. The spectroscopic data were in accordance with those in the literature. [11]

3-(Dodecyloxy)-4-methoxybenzaldehyde (FOR_274)

Synthesis according to GP 1: 3-hydroxy-4-methoxybenzaldehyde (5.00 g, 32.3 mmol), K_2CO_3 (9.10 g, 65.7 mmol), 1-bromododecane (9.01 g, 34.5 mmol), DMF (100 mL), yield: 10.2 g, 31.8 mmol, 97%, colourless solid.

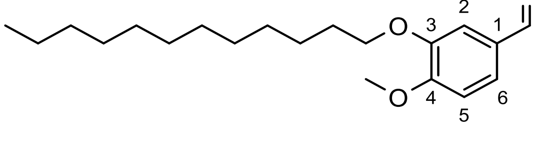

6c
$C_{20}H_{32}O_3$
(320.25)

^{1}H NMR (400 MHz, $CDCl_3$): δ = 0.84–0.91 (m, 3H, CH_3), 1.18–1.51 (m, 18H, CH_2), 1.81–1.92 (m, 2H, OCH_2CH_2), 3.95 (s, 3H, OCH_3), 4.07 (t, J = 6.9 Hz, 2H, OCH_2CH_2), 6.97 (d, J = 8.2 Hz, 1H, 5-H), 7.40 (d, J = 1.9 Hz, 1H, 2-H), 7.44 (dd, J = 8.2 Hz, 1.9 Hz, 1H, 6-H), 9.85 (s, 1H, CHO) ppm. ^{13}C NMR (126 MHz, $CDCl_3$) δ = 14.13 (CH_3), 22.70, 25.93, 28.99, 29.35, 29.37, 29.55, 29.60, 29.64, 29.66, 31.93 (CH_2), 56.17 (OCH_3), 69.08 (OCH_2), 110.22, 110.56 (C-2, C-5), 126.59 (C-6), 130.08 (C-1), 149.16, 154.82 (C-3, C-4), 190.97 (CHO) ppm.

3,4-Bis(dodecyloxy)benzaldehyde (6d)

Synthesis according to GP 1: 3,4-dihydroxybenzaldehyde (1.50 g, 10.86 mmol), K_2CO_3 (10.51 g, 76.02 mmol), 1-bromododecane (8.12 g, 32.6 mmol), yield: 4.33 g, 9.12 mmol, 84%, colourless solid.

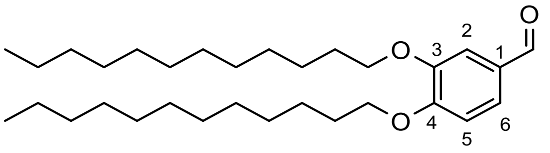

6d
$C_{31}H_{54}O_3$
(474.77)

^{1}H NMR (400 MHz, $CDCl_3$): δ = 0.85–0.92 (m, 6H, CH_3), 1.21–1.53 (m, 36H, CH_2), 1.79–1.91 (m, 4H, OCH_2CH_2), 4.01–4.12 (m, 4H, OCH_2CH_2), 6.95 (d, J = 8.1 Hz, 1H, 5-H), 7.37–7.45 (m, 2H, 2-H, 6-H), 9.83 (s, 1H, CHO) ppm. ^{13}C NMR (101 MHz, $CDCl_3$) δ = 14.14 (CH_3), 22.71, 25.96, 26.00, 28.98, 29.07, 29.38, 29.40, 29.61, 29.64, 29.68, 29.71, 31.94 (CH_2), 69.10 (OCH_2), 110.84, 111.68 (2-H, 5-H), 126.63, 129.83 (1-H, 6-H), 149.41, 154.65 (3-H, 4-H), 191.01 (CHO) ppm. The spectroscopic data were in accordance with those in the literature. [12]

2,3,4-Tris(dodecyloxy)benzaldehyde (6e)

Synthesis according to GP 1: 2,3,4-trihydroxybenzaldehyde (2.00 g, 12.98 mmol), K_2CO_3 (10.76 g, 77.86 mmol), 1-bromododecane (9.70 g, 38.9 mmol), DMF (100 mL, degassed), purification: column chromatography (PE/EE = 20 : 1), yield: 8.16 g, 12.38 mmol, 95%, colourless needles.

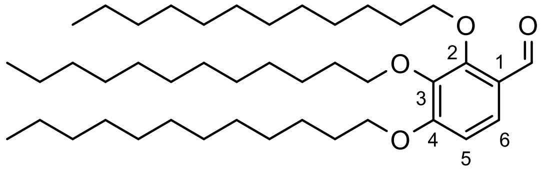

6e
$C_{43}H_{78}O_4$
(659.09)

^{1}H NMR (400 MHz, $CDCl_3$): δ = 0.83–0.94 (m, 9H, CH_3), 1.19–1.54 (m, 54H, CH_2), 1.72–1.89 (m, 6H, OCH_2CH_2), 3.97 (t, J = 6.6 Hz, 2H, OCH_2), 4.04 (t, J = 6.5 Hz, 2H, OCH_2), 4.17 (t, J = 6.7 Hz, 2H, OCH_2), 6.71 (d, J = 8.8 Hz, 1H, 5-H), 7.57 (d, J = 8.8 Hz, 1H, 6-H), 10.26 (s, 1H, CHO) ppm. ^{13}C NMR (101 MHz, $CDCl_3$) δ = 14.12 (CH_3), 22.71, 26.04, 26.07, 26.14, 29.19, 29.35, 29.38, 29.40, 29.49, 29.57, 29.63, 29.64, 29.67, 29.70, 29.72, 29.75, 30.19, 30.32, 31.94, 31.96 (CH_2), 68.95, 73.78, 75.36 (OCH_2), 108.09 (C-5), 123.50, 123.72 (C-1, C-6),

141.07, 156.72, 159.17 (C-2, C-3, C-4), 189.14 (CHO) ppm. The spectroscopic data were in accordance with those in the literature. [13]

Ethyl-3,4,5-tris(dodecyloxy)benzoate (2)

In MeCN (100 mL) gallic acid ethylester (2.00 g, 10.1 mmol), K_2CO_3 (9.76 g, 70.7 mmol), NaI (151 mg, 1.10 mmol) and 1-bromododecan (17.8 mg, 60.6 mmol) were suspended and heated under reflux for 2 d. After cooling to room temperature and evaporation of the solvent the residue was diluted in CH_2Cl_2 (50 mL) and filtrated over Celite®. The crude product was purified by column chromatography (PE/EE = 30 : 1). The product was obtained as colourless solid (yield: 6.73 g. 9.75 mmol. 95 %).

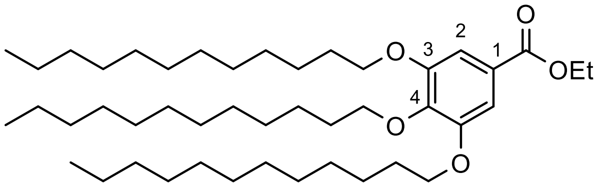

2
$C_{45}H_{82}O_5$
(703.15)

^{1}H NMR (300 MHz, $CDCl_3$): δ = 0.84–0.93 (m, 9H, CH_3), 1.18–1.54 (m, 54H, CH_2, OCH_2CH_3), 1.67–1.88 (m, 6H, OCH_2CH_2), 4.01 (t, J = 6.5 Hz, 6H, OCH_2CH_2), 4.35 (q, J = 7.1 Hz, 2H, OCH_2CH_3), 7.25 (s, 2H, CH) ppm. ^{13}C NMR (75 MHz, $CDCl_3$) δ = 14.12 (CH_3), 14.42 (OCH_2CH_3), 22.71, 26.08, 26.11, 29.34, 29.38, 29.42, 29.59, 29.65, 29.68, 29.72, 29.75, 29.77, 30.35, 31.94, 31.96 (CH_2), 60.95 (OCH_2CH_3), 69.20 (3-OCH_2), 73.49 (4-OCH_2), 108.02 (C-2), 125.05 (C-1), 142.35 (C-4), 152.81 (C-3), 166.47 (CO) ppm. The spectroscopic data were in accordance with those in the literature. [14]

(3,4,5-Tris(dodecyloxy)phenyl)methanol (3)

$LiAlH_4$ (1.36 g, 35.75 mmol) was suspended in abs. Et_2O (40 mL) and ester **2** (12.57 g, 17.88 mmol, dissolved in 50 mL Et_2O) was added dropwise to the suspension at room temperature. After 30 min the reaction mixture was cooled to 0 °C and water was added dropwise. After neutralizing with 2M H_2SO_4 the aqueous phase was extracted with Et_2O (3 x 100 mL) and dried over $MgSO_4$. After evaporation of solvent the product was obtained as a colourless solid and was used without further purification (11.56 g, 17.5 mmol, 98 %).

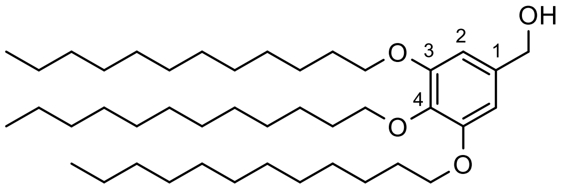

3
$C_{43}H_{80}O_4$
(661.11)

^{1}H NMR (400 MHz, $CDCl_3$): δ = 0.83–0.94 (m, 9H, CH_3), 1.19–1.52 (m, 54H, CH_2), 1.69–1.84 (m, 6H, OCH_2CH_2), 3.89–4.01 (m, 6H, OCH_2CH_2), 4.58 (d, J = 4.0 Hz, 2H, CH_2OH), 6.55 (s, 2H, 2-H) ppm. ^{13}C NMR (101 MHz, $CDCl_3$) δ = 14.12 (CH_3), 22.71, 26.13, 26.16, 29.38, 29.41, 29.44, 29.45, 29.64, 29.66, 29.68, 29.72, 29.76, 29.77, 30.36, 31.94, 31.96 (CH_2), 65.69 (CH_2OH), 69.15 (3-OCH_2), 73.45 (4-OCH_2), 105.42 (C-2), 136.04, 137.67 (C-1, C-4), 153.30 (C-3) ppm. The spectroscopic data were in accordance with those in the literature. [15]

3,4,5-Tris(dodecyloxy)benzaldehyde (6f)

Benzyl alcohol **3** (11.2 g, 15.1 mmol) was dissolved in dioxane (200 mL) and DDQ (2,3-dichloro-5,6-dicyano-1,4-benzoquinone, 3.42 g, 15.1 mmol) was added. After stirring for 1 h at room temperature the solvent was removed under reduced pressure. The residue was dissolved in CH_2Cl_2 and filtrated over Celite®. The crude product was purified by column chromatography (PE/EE = 20 : 1). The product was obtained as a slightly yellow solid (9.90 g, 13.3 mmol, 89 %).

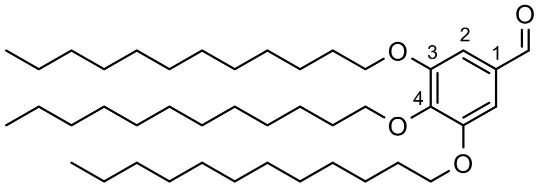

6f
$C_{43}H_{78}O_4$
(658.59)

^{1}H NMR (400 MHz, $CDCl_3$): δ = 0.83–0.92 (m, 9H, CH_3), 1.20–1.54 (m, 54H, CH_2), 1.70–1.90 (m, 6H, OCH_2CH_2), 3.99–4.09 (m, 6H, OCH_2CH_2), 7.08 (s, 2H, 2-H), 9.83 (s, 1H, CHO) ppm.

^{13}C NMR (101 MHz, $CDCl_3$) δ = 14.15 (CH_3), 22.71, 26.05, 26.09, 29.29, 29.38, 29.40, 29.56, 29.65, 29.68, 29.72, 29.74, 29.76, 30.37, 31.95 (CH_2), 69.22 (3-OCH_2), 73.64 (4-OCH_2), 107.81 (C-2), 131.43 (C-1), 143.81 (C-4), 153.52 (C-3), 191.33 (CHO) ppm. The spectroscopic data were in accordance with those in the literature. [16]

1.2 Syntheses of phenols (7a-f)

4-(Dodecyloxy)phenol (7a)

Synthesis according to GP 2: **6a** (3.00 g, 10.3 mmol), H_2O_2 (35 wt% in H_2O, 2.50 mL, 25.8 mmol), H_2SO_4 (0.22 mL, 4.13 mmol), yield: 2.15 g, 7.72 mmol, 75%, colourless solid.

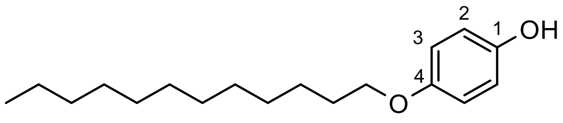

7a
$C_{18}H_{30}O_2$
(278.44)

^{1}H NMR (400 MHz, $CDCl_3$): δ = 0.83–0.92 (m, 3H, CH_3), 1.19–1.49 (m, 18H), 1.70–1.80 (m, 2H, OCH_2CH_2), 3.89 (t, J = 6.6 Hz, 2H, OCH_2CH_2), 4.52 (s, 1H, OH), 6.72–6.81 (m, 4H, 2-H, 3-H) ppm. ^{13}C NMR (101 MHz, $CDCl_3$) δ = 14.14 (CH_3), 22.70, 26.05, 29.36, 29.42, 29.59, 29.61, 29.64, 29.67, 31.92 (CH_2), 68.74 (OCH_3), 115.59, 115.97 (C-2, C-3), 149.26, 153.30 (C-1, C-4) ppm. The spectroscopic data were in accordance with those in the literature. [17]

4-(Dodecyloxy)-3-methoxyphenol (7b)

Synthesis according to GP 2: **6b** (4.00 g, 12.5 mmol), H_2O_2 (35 wt% in H_2O, 3.03 mL, 31.2 mmol), H_2SO_4 (0.27 mL, 5.00 mmol), yield: 2.99 mg, 9.68 mmol, 78%, rose coloured solid.

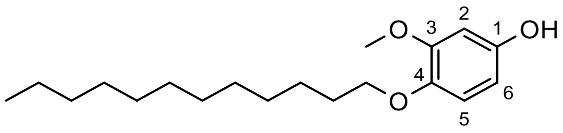

7b
$C_{19}H_{32}O_3$
(308.46)

^{1}H NMR (400 MHz, $CDCl_3$): δ = 0.83–0.93 (m, 3H, CH_3), 1.18–1.48 (m, 18H), 1.73–1.84 (m, 2H, OCH_2CH_2), 3.80 (s, 3H, OCH_3), 3.93 (t, J = 6.9 Hz, 2H, OCH_2CH_2), 4.88 (s, 1H, OH), 6.31 (dd, J = 8.6 Hz, 2.8 Hz, 1H, 6-H), 6.45 (d, J = 2.8 Hz, 1H, 2-H), 6.74 (d, J = 8.6 Hz, 1H, 5-H) ppm. ^{13}C NMR (101 MHz, $CDCl_3$) δ = 14.12 (CH_3), 22.70, 25.98, 29.36, 29.44, 29.59, 29.61, 29.65, 29.67, 31.93 (CH_2), 55.87 (OCH_3), 70.26 (OCH_2), 100.83 (C-2), 105.97 (C-6), 114.94 (C-5), 142.60 (C-4), 150.26, 150.60 (C-1, C-3) ppm. FT-IR (ATR): $\tilde{\nu}$ = 3455 (m), 2920 (vs), 2851 (s), 1611 (m), 1508 (vs), 1468 (m), 1433 (m), 1389 (w), 1350 (w), 1290 (s), 1216 (vs), 1154 (m), 1130 (m), 1069 (w), 1030 (m), 952 (s), 836 (m), 795 (m), 721 (w), 622 (w), 548

(w), 500 (w) cm^{-1}. MS (ESI): m/z = 331.22 $[M+Na]^+$. HRMS (ESI) for $[C_{19}H_{32}O_3Na]^+$: calc.: 331.2244, found: 331.2234.

3-(Dodecyloxy)-4-methoxyphenol (7c)

Synthesis according to GP 2: **6c** (4.00 g, 12.5 mmol), H_2O_2 (35 wt% in H_2O, 3.03 mL, 31.2 mmol), H_2SO_4 (0.27 mL, 5.00 mmol), yield: 2.96 mg, 9.60 mmol, 77%, grey solid.

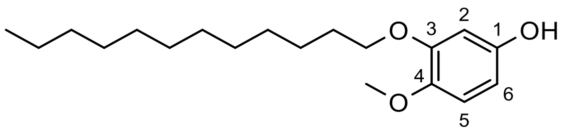

7c
$C_{19}H_{32}O_3$
(308.46)

^1H NMR (400 MHz, $CDCl_3$): δ = 0.80–0.95 (m, 3H, CH_3), 1.17–1.49 (m, 18H, CH_2), 1.74–1.88 (m, 2H, OCH_2CH_2), 3.80 (s, 3H, OCH_3), 3.88–4.04 (m, 2H, OCH_2CH_2), 6.32 (dd, J = 8.6 Hz, 2.8 Hz, 1H, 6-H), 6.46 (d, J = 2.8 Hz, 1H, 2-H), 6.73 (d, J = 8.6 Hz, 1H, 5-H) ppm. ^{13}C NMR (101 MHz, $CDCl_3$) δ = 14.12 (CH_3), 22.70, 25.97, 29.08, 29.36, 29.41, 29.43, 29.57, 29.61, 29.65, 29.67, 31.93 (CH_2), 56.89 (OCH_3), 68.97 (OCH_2), 101.89 (C-2), 105.83 (C-6), 113.23 (C-5), 143.51 (C-4), 149.65, 150.20(C-1, C-3) ppm.

3,4-Bis(dodecyloxy)phenol (7d)

Synthesis according to GP 2: **7d** (1.00 g, 2.11 mmol), H_2O_2 (35 wt% in H_2O, 0.5 mL, 5.27 mmol), H_2SO_4 (0.04 mL, 0.84 mmol), yield: 594 mg, 2.11 mmol, 61%, colourless solid.

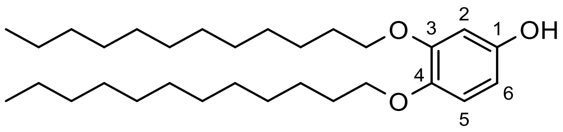

7d
$C_{30}H_{54}O_3$
(462.76)

^1H NMR (400 MHz, $CDCl_3$): δ = 0.83–0.92 (m, 6H, CH_3), 1.19–1.50 (m, 36H, CH_2), 1.70–1.85 (m, 4H, OCH_2CH_2), 3.87–3.97 (m, 4H, OCH_2CH_2), 4.65 (s, 1H, OH), 6.29 (dd, J = 8.6 Hz, 2.8 Hz, 1H, 6-H), 6.44 (d, J = 2.8 Hz, 1H, 2-H), 6.75 (d, J = 8.6 Hz, 1H, 5-H) ppm. ^{13}C NMR (101 MHz, $CDCl_3$) δ = 14.13 (CH_3), 22.71, 26.05, 29.19, 29.38, 29.43, 29.48, 29.52, 29.65, 29.67, 29.71, 31.94 (CH_2), 68.96, 70.77 (OCH_2), 102.19 (C-2), 106.05 (C-6), 116.30

(C-5), 142.97 (C-4), 150.37, 150.52 (C-1, C-3) ppm. The spectroscopic data were in accordance with those in the literature. [8]

2,3,4-Tris(dodecyloxy)phenol (7e)

Synthesis according to GP 2: **6e** (2.00 g, 3.03 mmol), H_2O_2 (35 wt%, 0.86 mL, 7.60 mmol), H_2SO_4 (0.06 mL, 1.21 mmol), yield: 1.89 mg, 2.91 mmol, 96%, colourless solid.

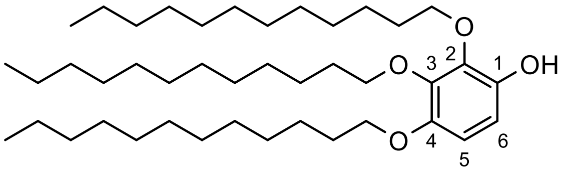

7e
$C_{42}H_{78}O_4$
(647.08)

^{1}H NMR (500 MHz, $CDCl_3$): δ = 0.82–0.92 (m, 9H, CH_3), 1.18–1.50 (m, 54H, CH_2), 1.68–1.82 (m, 6H, OCH_2CH_2), 3.89 (t, J = 6.5 Hz, 2H, 4-OCH_2), 3.99 (t, J = 6.7 Hz, 2H, 3-OCH_2), 4.11 (t, J = 6.8 Hz, 2H, 2-OCH_2), 5.37 (s, 1H, OH), 6.53 (d, J = 9.0 Hz, 1H, 5-H), 6.58 (d, J = 9.0 Hz, 1H, 6-H) ppm. ^{13}C NMR (126 MHz, $CDCl_3$) δ = 14.13 (CH_3), 22.71, 26.02, 26.17, 29.38, 29.40, 29.47, 29.57, 29.62, 29.66, 29.68, 29.71, 29.73, 29.75, 30.31, 31.94, 31.95 (CH_2), 69.59 (4-OCH_2), 73.64 (3-OCH_2), 74.05 (2-OCH_2), 108.09 (C-6), 108.90 (C-5), 139.78 (C-2), 141.92 (C-3), 143.59 (C-1), 146.63 (C-4) ppm. FT-IR (ATR): $\tilde{\nu}$ = 3540 (w), 2921 (vs), 2852 (vs), 1490 (s), 1467 (s), 1380 (m), 1354 (m), 157 (m), 1189 (m), 1163 (m), 1086 (s), 1058 (m), 967 (w), 790 (m), 722 (w), 458 (w) cm^{-1}.MS (ESI): m/z = 669.58 $[M+Na]^+$, 647.60 $[M+H]^+$. HRMS (ESI) for $[C_{42}H_{78}O_4Na]^+$: calc.: 647.5973, found.: 647.5955.

3,4,5-Tris(dodecyloxy)phenol (7f)

Synthesis according to GP 2: **6f** (1.50 g, 2.28 mmol), H_2O_2 (35wt%, 0.55 mL, 5.69 mmol), H_2SO_4 (0.05 mL, 0.91 mmol), yield: 963 mg, 1.49 mmol, 65%, colourless solid.

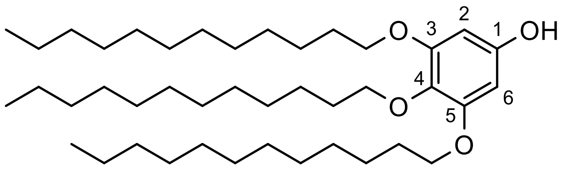

7f
$C_{42}H_{78}O_4$
(647.08)

^{1}H NMR (400 MHz, $CDCl_3$): δ = 0.81–0.92 (m, 9H, CH_3), 1.17–1.51 (m, 54H, CH_2), 1.66–1.81 (m, 6H, OCH_2CH_2), 3.83–3.92 (m, 6H, OCH_2CH_2), 4.79 (s, 1H, OH), 6.03 (s, 2H, CH) ppm. ^{13}C NMR (101 MHz, $CDCl_3$) δ = 14.12, (CH_3), 22.71, 26.10, 26.17, 29.35, 29.39, 29.41, 29.44, 29.67, 29.68, 29.72, 29.76, 29.78, 30.28, 31.95, 31.96 (CH_2), 69.00 (3-OCH_2), 73.65 (4-OCH_2), 94.26 (C-2), 132.05 (C-4), 151.73 (C-3), 153.57 (C-1) ppm. The spectroscopic data were in accordance with those in the literature. [18]

1.3 Syntheses of ethynylalkohols (8a-g)

1-(4-(Dodecyloxy)phenyl)prop-2-yn-1-ol (8a)

Synthesis according to GP 3: **8a** (2.00 g, 6.89 mmol), ethynylmagnesium bromide (0.5 M in THF, 17.9 mL, 8.95 mmol), yield: 2.16 g, 6.82 mmol, 99%, yellow solid.

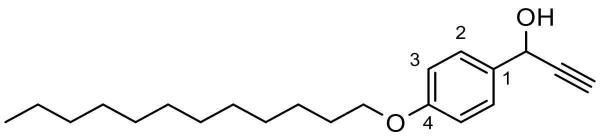

8a
$C_{21}H_{32}O_2$
(316.49)

^{1}H NMR (500 MHz, $CDCl_3$): δ = 0.88 (t, J = 6.9 Hz, 3H, CH_3), 1.19–1.49 (m, 18H, CH_2), 1.74–1.81 (m, 2H, OCH_2CH_2), 2.67 (d, J = 2.2 Hz, 1H, CCH), 3.95 (t, J = 6.6 Hz, 2H, OCH_2OCH_2), 5.41 (d, J = 2.2 Hz, 1H, C*H*OH), 6.86–6.92 (m, 2H, 3-H), 7.43–7.48 (m, 2H, 2-H) ppm. ^{13}C NMR (126 MHz, $CDCl_3$) δ = 14.17 (CH_3), 22.70, 25.99, 29.17, 29.36, 29.39, 29.40, 29.43, 29.58, 29.60, 29.63, 29.66, 29.68, 29.71, 31.91 (CH_2), 64.03 (CHOH), 68.00 (OCH_2), 74.64 (C*C*H), 83.57 (*C*CH), 114.47 (C-3), 128.03 (C-2), 131.94 (C-1), 159.29 (C-4) ppm. FFT-IR (ATR): $\tilde{\nu}$ = 3306 (w), 2919 (w), 2851 (w), 2252 (w), 1686 (w), 1610 (w), 1510 (m), 1468 (w), 1392 (w), 1304 (w), 1250 (m), 1173 (w), 1111 (w), 1018 (w), 950 (w), 905 (vs), 833 (w), 728 (vs), 649 (m), 575 (w) cm^{-1}. MS (ESI): m/z = 339 $[M+Na]^+$, 299 $[M\ -OH]^+$, 131 $[M\ -OC_{12}H_{25}]^+$. HRMS (ESI) for $[C_{21}H_{32}O_2Na]^+$: calc.: 339.2295, found.: 339.2276.

1-(3,4-Bis(dodecyloxy)phenyl)prop-2-yn-1-ol (8d)

Synthesis according to GP 3: **6d** (2.00 g, 4.21 mmol), ethynylmagnesium bromide (0.5 M in THF, 10.95 mL, 5.47 mmol), yield: 2.10 g, 4.21 mmol, 99 %, yellow solid.

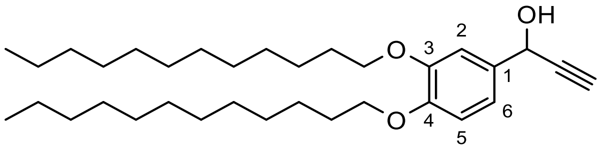

8d
$C_{33}H_{56}O_3$
(500.81)

^{1}H NMR (500 MHz, $CDCl_3$): δ = 0.82–0.93 (m, 6H, CH_3), 1.20–1.51 (m, 36H), 1.75–1.88 (m, 4H, OCH_2CH_2), 2.17 (s, 1H, OH), 2.66 (d, *J* = 2.2 Hz, 1H, CCH), 3.96–4.03 (m, 4H, OCH_2CH_2), 5.36–5.41 (m, 1H, C*H*OH), 6.86 (d, *J* = 8.2 Hz, 1H, 5-H), 7.05 (dd, *J* = 8.2 Hz, 2.2 Hz, 1H, 6-H), 7.08 (d, *J* = 2.2 Hz, 1H, 2-H) ppm. ^{13}C NMR (126 MHz, $CDCl_3$) δ = 14.13 (CH_3), 22.70, 26.03, 26.05, 29.26, 29.29, 29.38, 29.43, 29.45, 29.65, 29.67, 29.71, 31.94 (CH_2), 64.36 (CHOH), 69.28, 69.35 (OCH_2), 74.59 (C*C*H), 83.66 (*C*CH), 112.31 (C-2), 113.57 (C-5), 119.17 (C-6), 132.73 (C-1), 149.31, 149.44 (C-3, C-4) ppm. FT-IR (ATR): $\tilde{\nu}$ = 3313 (w), 3293 (w), 2955 (m), 2917 (vs), 2849 (vs), 1592 (w), 1517 (m), 1467 (m), 1427 (m), 1390 (m), 1350 (w), 1257 (s), 1234 (s), 1160 (w), 1137 (s), 1070 (w), 1026 (m), 1003 (m), 956 (w), 912 (w), 853 (w), 806 (w), 776 (w), 762 (w), 722 (m), 649 (m), 621 (m) cm^{-1}. MS (ESI): *m*/*z* = 1024 $[2M+Na]^+$, 523.41 $[M+Na]^+$. HRMS (ESI) for $[C_{33}H_{56}O_3Na]^+$: calc.: 523.4122, found.: 523.4126.

1-(2,3,4-Tris(dodecyloxy)phenyl)prop-2-yn-1-ol (8e)

Synthesis according to GP 3: **6e** (2.00 g, 3.03 mmol), ethynylmagnesium bromide (0.5 M in THF, 7.9 mL, 3.99 mmol), yield: 2.66 g, 3.01 mmol, 99%, yellow solid.

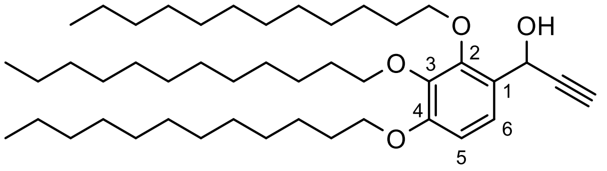

8e
$C_{45}H_{80}O_4$
(685.13)

^{1}H NMR (700 MHz, $CDCl_3$): δ = 0.88 (t, *J* = 7.1 Hz, 9H, CH_3), 1.22–1.51 (m, 54H, CH_2), 1.72–1.84 (m, 6H, OCH_2CH_2), 2.60 (d, *J* = 2.3 Hz, 1H, CCH), 3.02 (d, *J* = 6.7 Hz, 1H, OH), 3.93–3.98 (m, 4H, 3-OCH_2, 4-OCH_2), 4.18 (t, *J* = 6.8 Hz, 2H, OCH_2, 2-OCH_2), 5.56 (dd, *J* = 6.7 Hz, 2.3 Hz, 1H, C*H*OH), 6.63 (d, *J* = 8.5 Hz, 1H, 5-H), 7.17 (d, *J* = 8.5 Hz, 1H, 6-H) ppm. ^{13}C NMR (176 MHz, $CDCl_3$) δ = 14.13 (CH_3), 22.71, 26.05, 26.13, 26.16, 29.32, 29.38, 29.40, 29.42, 29.54, 29.60, 29.65, 29.67, 29.71, 29.74, 29.76, 30.34, 30.37, 31.94, 31.95 (CH_2), 61.20 (CHOH), 68.72, 73.61, 73.90 (3-OCH_2, 4-OCH_2, C*C*H), 74.17(2-OCH_2), 83.93 (*C*CH), 107.89 (C-5), 121.93 (C-6), 126.27 (C-1), 141.60 (C-3), 150.92 (C-2), 153.96 (C-

4) ppm. FT-IR (ATR): $\tilde{\nu}$ = 3311 (w), 2921 (vs), 2851 (vs), 1599 (w), 1493 (m), 1454 (m), 1379 (m), 1281 (m), 1176 (w), 1093 (s), 1018 (m), 919 (w), 796 (w), 721 (w), 650 (w), 626 (m), 563 (w) cm^{-1}. MS (ESI): *m*/*z* = 707.59 [M+Na]$^+$, 667.6 [M-OH]$^+$. HRMS (ESI) for [$C_{45}H_{80}O_4Na$]$^+$: calc.: 707.5949, found.: 707.5985.

1-(3,4,5-Tris(dodecyloxy)phenyl)prop-2-yn-1-ol (8f)

Synthesis according to GP 3: **6f** (1.50 g, 2.28 mmol), ethynylmagnesium bromide (0.5 M in THF, 6.00 mL, 3.00 mmol), yield: 1.55 g, 2.26 mmol, 99%, yellow solid.

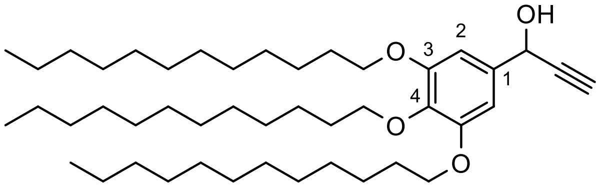

8f
$C_{45}H_{80}O_4$
(685.13)

^{1}H NMR (500 MHz, $CDCl_3$): δ = 0.88 (t, *J* = 6.9 Hz, 9H, CH_3), 1.20–1.50 (m, 54H, CH_2), 1.69–1.84 (m, 6H, OCH_2), 2.18 (d, *J* = 6.0 Hz, 1H, OH), 2.66 (d, *J* = 2.2 Hz, 1H, CCH), 3.94 (t, *J* = 6.6 Hz, 2H, 4-H), 3.98 (t, *J* = 6.5 Hz, 4H, 3-H), 5.37 (dd, *J* = 6.0 Hz, 2.2 Hz, 1H, CHOH), 6.74 (s, 2H, 2-H) ppm. ^{13}C NMR (126 MHz, $CDCl_3$) δ = 14.12 (CH_3), 22.71, 26.12, 26.14, 29.38, 29.40, 29.43, 29.63, 29.66, 29.67, 29.72, 29.75, 29.77, 30.33, 31.94, 31.96 (CH_2), 64.66 (CHOH), 69.14 (3-OCH_2), 73.44 (4-OCH_2), 74.70 (C*C*H), 83.51 (*C*CH), 105.05 (C-2), 134.99 (C-1), 138.31 (C-4), 153.26 (C-3) ppm. FT-IR (ATR): $\tilde{\nu}$ = 3311 (w), 2920 (vs), 2852 (s), 1593 (m), 1503 (m), 1466 (m), 1347 (m), 1380 (m), 1331 (m), 1231 (m), 1115 (vs), 1011 (m), 905 (w), 838 (w), 721 (m), 651 (m) cm^{-1}. MS (ESI): *m*/*z* = 707.59 [M+Na]$^+$. HRMS (ESI) for [$C_{45}H_{80}O_4Na$]$^+$: calc.: 707.5949, found.: 707.5974.

1-Phenylprop-2-yn-1-ol (8g)

Synthesis according to GP 3: benzaldehyde (1.00 g, 9.42 mmol), ethynylmagnesium bromide (0.5 M in THF, 20.7 mL, 10.4 mmol), yield: brown oil, the crude product was directly submitted into the next reaction without complete removal of solvent.

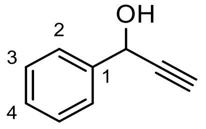

8g
C_9H_8O
(132.16)

^{1}H NMR (300 MHz, $CDCl_3$): δ = 2.31 (s, 1H, OH), 2.67 (d, *J* = 2.3 Hz, 1H, CCH), 5.47 (s, 1H, CHOH), 7.30–7.44 (m, 3H, Ar), 7.53–7.59 (m, 2H, Ar) ppm. The spectroscopic data were in accordance with those in the literature. [9]

1.4 Syntheses of ethynylketones (9a-g)

1-(4-(Dodecyloxy)phenyl)prop-2-yn-1-one (9a)

Synthesis according to GP 4: **8a** (2.00 g, 6.32 mmol), IBX (3.54 g, 12.6 mmol), yield: 1.78 g, 5.67 mmol, 90%, yellow solid.

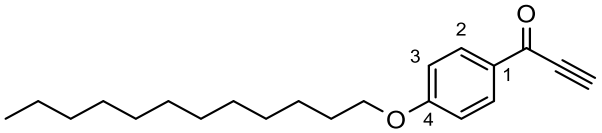

9a
$C_{21}H_{30}O_2$
(314.47)

^{1}H NMR (500 MHz, $CDCl_3$): δ = 0.88 (t, *J* = 6.9 Hz, 3H, CH_3), 1.22–1.50 (m, 18H, CH_2), 1.77–1.85 (m, 2H, OCH_2CH_2), 3.36 (s, 1H, CCH), 4.04 (t, *J* = 6.6 Hz, 2H, OCH_2CH_2), 6.91–6.97 (m, 2H, 2-H), 8.08–8.15 (m, 2H, 3-H) ppm. ^{13}C NMR (126 MHz, $CDCl_3$) δ = 14.13 (CH_3), 22.69, 25.95, 29.04, 29.34, 29.35, 29.54, 29.58, 29.63, 29.65, 31.92 (CH_2), 68.47 (OCH_2), 79.87 (C*C*H), 80.46 (*C*CH), 114.38 (C-3), 129.37 (C-1), 132.15 (C-2), 164.46 (C-4), 175.92 (CO) ppm. FT-IR (ATR): $\tilde{\nu}$ = 3242 (w), 2922 (s), 2852 (m), 2095 (m), 1642 (s), 1595 (vs), 1572 (m), 1508 (m), 1467 (m), 1423 (w) ,1392 (w), 1314 (m); 1249 (vs), 1163 (vs), 1117 (w), 1015 (m), 991 (m), 844 (m), 759 (s), 721 (w), 691 (m), 646 (w), 633 (w), 617 (m), 510 (w) cm^{-1}. MS (ESI): *m/z* = 315.23 $[M+H]^+$. HRMS (ESI) for $[C_{21}H_{31}O_2]^+$: calc.: 315.2319, found.: 315.2306.

1-(3,4-Bis(dodecyloxy)phenyl)prop-2-in-1-one (9c)

Synthesis according to GP 4: **8c** (1.87 g, 3.73 mmol), IBX (2.09 g, 7.46 mmol), yield1.67 g, 3.36 mmol, 90%, yellow solid.

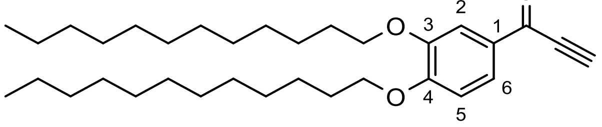

9c
$C_{33}H_{54}O_3$
(498.79)

^{1}H NMR (500 MHz, $CDCl_3$): δ = 0.84–0.92 (m, 6H), 1.20–1.53 (m, 36H, CH_2), 1.78–1.91 (m, 4H, (OCH_2CH_2), 3.35 (s, 1H, CCH), 4.02–4.11 (m, 4H, OCH_2CH_2), 6.90 (d, *J* = 8.5 Hz, 1H, 5-

H), 7.60 (d, *J* = 2.0 Hz, 1H, 2-H), 7.82 (dd, *J* = 8.4 Hz, 2.0 Hz, 1H; 6-H) ppm. ^{13}C NMR (126 MHz, $CDCl_3$) δ = 14.12 (CH_3), 22.70, 25.95, 26.00, 28.97, 29.08, 29.36, 29.38, 29.40, 29.61, 29.63, 29.67, 29.70, 29.71, 31.93 (CH_2), 69.13, 69.21 (OCH_2), 79.72 (C*C*H, 80.48 (*C*CH), 111.47 (C-5), 112.18 (C-2), 125.93 (C-6), 129.46 (C-1), 148.91 (C-3), 154.95 (C-4), 176.03 (CO) ppm. FT-IR (ATR): $\tilde{\nu}$ = 3234 (w), 2915 (s), 2848 (s), 2090 (w), 1642 (s) ,1591 (m), 1581 (s), 1514 (s), 1466 (m), 1431 (s), 1389 (w), 1354 (w), 1289 (m), 1270 (vs), 1247 (s), 1209 (s), 1140 (s), 1070 (m), 1042 (w), 1031 (w), 994 (m), 958 (w), 924 (w), 885 (w), 876 (m), 826 (w), 808 (w), 794 (w), 752 (s), 723 (m), 706 (m), 622 (m), 579 (w) cm^{-1}. MS (ESI): *m*/*z* = 1519 [3M+Na$^+$], 1020 [2M+Na$^+$], 521 [M+Na$^+$]. HRMS (ESI) for $[C_{33}H_{54}O_3Na]^+$: calc.: 521.3965, found.: 521.3937.

1-(2,3,4-Tris(dodecyloxy)phenyl)prop-2-in-1-on (9e)

Synthesis according to GP 4: **8c** (2.06 g, 3.01 mmol), IBX (1.68 g, 6.02 mmol), yield: 1.29 g, 1.88 mmol, 63%, yellow solid.

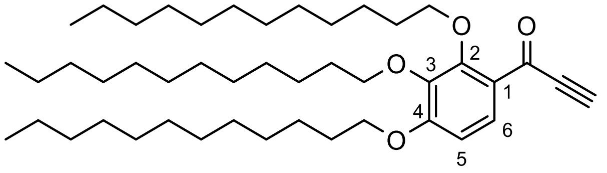

FOR_380

$C_{45}H_{78}O_4$
(683.12)

^{1}H NMR (400 MHz, $CDCl_3$): δ = 0.83–0.92 (m, 9H, CH_3), 1.19–1.54 (m, 54H, CH_2), 1.71–1.89 (m, 6H, OCH_2CH_2), 3.30 (s, 1H, CCH), 3.96 (t, *J* = 6.6 Hz, OCH_2), 4.04 (t, *J* = 6.5 Hz, 2H, OCH_2), 4.09 (t, *J* = 6.9 Hz, 2H, OCH_2), 6.69 (d, *J* = 9.0 Hz, 1H, 5-H), 7.78 (d, *J* = 9.0 Hz, 1H, 6-H) ppm. ^{13}C NMR (101 MHz, $CDCl_3$) δ = 14.12 (CH_3), 22.71, 25.99, 26.06, 26.14, 29.15, 29.38, 29.40, 29.58, 29.61, 29.63, 29.67, 29.68, 29.70, 29.72, 29.74, 29.76, 30.10, 30.34, 31.94, 31.95 (CH_2), 68.93 (4-OCH_2), 73.89, 75.00, (2-OCH_2, 3-OCH_2), 78.57 (C*C*H), 82.42 (*C*CH), 107.47 (C-5), 124.34, 128.43 (C-1, C-6), 142.10, 154.78, 158.70 (C-2, C-3, C-4), 174.75 (CO) ppm. FT-IR (ATR): $\tilde{\nu}$ = 3303 (w), 3240 (w), 2921 (vs), 2852 (s), 2092 (w), 1650 (m), 1586 (s), 1563 (w), 1492 (w), 1466 (m), 1444 (w), 1378 (w), 1286 (s), 1261 (s), 1195 (m), 1124 (m), 1101 (m), 1079 (m), 1001 (w), 795 (w), 732 (w), 681 (w), 643 (w) cm^{-1}. MS (ESI): *m*/*z* = 705.57 [M+Na]$^+$, 683.59 [M+H]$^+$. HRMS (ESI) for $[C_{45}H_{78}O_4H]^+$: calc.: 683.5973, found.: 683.5961.

1-(3,4,5-Tris(dodecyloxy)phenyl)prop-2-yn-1-one (9f)

Synthesis according to GP 4: **8f** (1.50 g, 2.19 mmol), IBX (1.23 g, 4.38 mmol), yield: 1.29 g, 1.89 mmol, 86%, yellow solid.

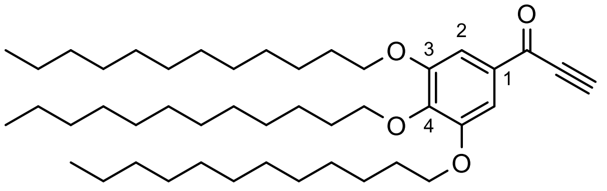

9f
$C_{45}H_{78}O_4$
(683.12)

^{1}H NMR (500 MHz, $CDCl_3$): δ = 0.85–0.91 (m, 9H, CH_3), 1.20–1.52 (m, 54H), 1.70–1.87 (m, 6H, OCH_2CH_2), 3.39 (s, 1H, CCH), 4.01–4.09 (m, 6H, OCH_2CH_2), 7.39 (s, 2H, C-2) ppm. ^{13}C NMR (126 MHz, $CDCl_3$) δ = 14.14 (CH_3), 22.72, 26.04, 26.10, 29.27, 29.39, 29.41, 29.56, 29.65, 29.69, 29.72, 29.75, 29.77, 30.36, 31.95, 31.97 (CH_2), 69.26 (3-OCH_2), 73.69 (4-OCH_2), 80.18, 80.39 (CCH), 108.15 (C-2), 131.06 (C-1), 144.25 (C-4), 153.00 (C-3), 176.35 (CO) ppm. FT-IR (ATR): $\tilde{\nu}$ = 3247 (w), 2955 (m), 2918 (vs), 2872 (m), 2848 (s), 2090 (m), 1643 (s), 1580 (s), 1501 (m), 1468 (s), 1428 (s), 1387 (m), 1334 (vs), 1231 (m), 1190 (vs), 1121 (vs), 1070 (m), 989 (m), 886 (m), 856 (m), 745 (s), 720 (m), 695 (m), 679 (m), 649 (w), 607 (w), 689 (w) cm^{-1}. MS (ESI): *m/z* = 705.6 $[M+Na]^+$, 684.6 $[M+H]^+$. HRMS (ESI) for $[C_{21}H_{31}O_2]^+$: calc.: 705.5792, found.: 705.5781.

1.5 Syntheses of flavylium salts (A-Fla-B)

6-(Dodecyloxy)-2-phenyl-7-methoxychromenium triflate (V-Fla-0)

Synthesis according to GP 5: phenol **7b** (100 mg, 324 µmol), ethynylketone **9g** (42 mg, 324 µmol), EtOAc (5 mL), yield: 66 mg, 115 µmol, 36 %, yellow needles.

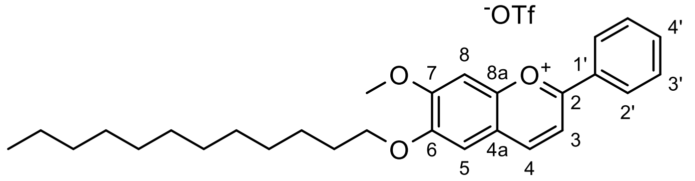

$C_{29}H_{37}F_3O_6S$
(570.66)

FOR_459

^{1}H NMR (700 MHz, $CDCl_3$): δ = 0.88 (d, *J* = 7.2 Hz, 3H, CH_3), 1.22–1.53 (m, 18H, CH_2), 1.87–1.94 (m, 2H, OCH_2CH_2), 4.17 (t, *J* = 6.6 Hz, 2H, OCH_2OCH_2), 4.25 (s, 3H, OCH_3), 7.51 (s, 1H, 5-H), 7.58–7.64 (m, 2H, 3'-H), 7.65–7.70 (m, 1H, 4'-H), 7.82 (s, 1H, 8-H), 8.31 (d,

J = 7.5 Hz, 2H, 2'-H), 8.36 (d, J = 8.5 Hz, 1H, 3-H), 9.36 (d, J = 8.5 Hz, 1H, 4-H) ppm. ^{13}C NMR (176 MHz, $CDCl_3$) δ = 14.13 (CH_3), 22.70, 25.89, 28.69, 29.36, 29.55, 29.62, 29.66, 29.69, 31.93 (CH_2), 58.36 (OCH_3), 70.49 (OCH_2), 100.28 (C-8), 107.48 (C-5), 114.10 (C-3), 120.91 (q, J = 320.4 Hz, CF_3), 121.71 (C-4a), 128.45 (C-1'), 128.79 (C-2'), 130.20 (C-3'), 135.72 (C-4'), 151.89 (C-6), 153.65 (C-4), 155.90 (C-8a), 162.51 (C-7), 168.92 (C-2) ppm. FT-IR (ATR): $\tilde{\nu}$ = 3066 (w, br.), 2922 (s), 2853 (m), 1625 (m), 1598 (w), 1560 (w), 1510 (s), 1466 (m), 1416 (m), 1397 (s), 1341 (s), 1320 (m), 1263 (vs), 1212 (s), 1155 (vs), 1128 (m),1031 (vs), 997 (m), 909 (m), 874 (m), 776 (w), 726 (m), 680 (m), 637 (s), 600 (w), 573 (w), 517 (m), 485 (w) cm^{-1}. MS (ESI): m/z = 421.272 $[M]^+$. HRMS (ESI) for $[C_{28}H_{37}O_3]^+$: calc.: 421.2737, found.: 421.2718. CHNS analysis: calc.: C 61.04, H 6.54, S 5.62; found.: C 61.00, H 6.41, S 5.32.

6-(Dodecyloxy)-2-[4-(dodecyloxy)phenyl]-7-methoxychromenium triflate (V-Fla-1)

Synthesis according to GP 5: phenol **7b** (50 mg, 162 µmol), ethynylketone **9a** (51 mg, 162 µmol), EtOAc (5 mL), yield: 103 mg, 136 µmol, 84 %, orange needles.

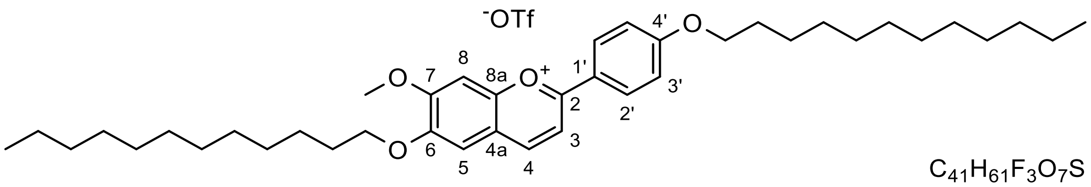

FOR_334

$C_{41}H_{61}F_3O_7S$
(754.99)

^{1}H NMR (700 MHz, $CDCl_3$): δ = 0.84–0.91 (m, 6H, CH_3), 1.20–1.54 (m, 36H, CH_2), 1.79–1.92 (m, 4H, OCH_2CH_2), 4.05 (t, J = 6.6 Hz, 2H, 4'-OCH_2), 4.11 (t, J = 6.7 Hz, 2H, 6-OCH_2), 4.21 (s, 3H, OCH_3)), 7.01 (d, J = 9.0 Hz, 2H, 3'-H), 7.30 (s, 1H, 5-H), 7.80 (s, 1H, 8-H), 8.19 (d, J = 8.7 Hz, 1H, 3-H), 8.28 (d, J = 9.0 Hz, 2H, 2'-H), 9.03 (d, J = 8.7 Hz, 1H, 4-H) ppm. ^{13}C NMR (176 MHz, $CDCl_3$) δ = 14.13 (CH_3), 22.70, 25.92, 28.78, 28.99, 29.37, 29.39, 29.41, 29.58, 29.62, 29.64, 29.66, 29.67, 29.69, 31.93 (CH_2), 58.14 (OCH_3), 69.18, 70.20 (OCH_2), 100.58 (C-8), 107.33 (C-5), 113.39 (C-3), 116.34 (C-3'), 119.91 (C-4a), 120.29 (C-1'), 120.97 (q, J = 320.5 Hz, CF_3), 131.89 (C-2'), 151.11 (C-6), 151.89 (C-4), 154.45 (C-8a), 160.96 (C-7), 166.27 (C-4'), 169.20 (C-2) ppm. FT-IR (ATR): $\tilde{\nu}$ = 2922 (vs), 2853 (s), 1625 (m), 1604 (m), 1556 (m), 1534 (m), 1515 (s), 1467 (m), 1398 (m), 1353 (m), 1323 (m), 1307 (m), 1268 (vs), 1210 (m), 1190 (m), 1158 (s), 1126 (m), 1031 (s), 999 (w), 871 (w), 843 (w), 747 (w), 719 (w), 638 (m), 574 (w), 518 (w) cm^{-1}. MS (ESI): m/z = **605.46** $[M]^+$, 437.27 $[M+H -C_{12}H_{25}]^+$, 269.08

$[M+H_2 -C_{24}H_{50}]^+$. HRMS (ESI) for $[C_{40}H_{61}O_4]^+$: calc.: 605.4564, found.: 605.4574. CHNS analysis: calc.: C 65.23, H 8.14, S 4.25; found.: C 65.46, H 8.14, S 3.98.

6-(Dodecyloxy)-2-[3,4-bis(dodecyloxy)phenyl]-7-methoxychromenium triflate (V-Fla-2)

Synthesis according to GP 5: phenol **7b** (50 mg, 162 µmol), ethynylketone **9d** (81 mg, 162 µmol), EtOAc (5 mL), yield: 127 mg, 135 µmol, 83 %, red powder.

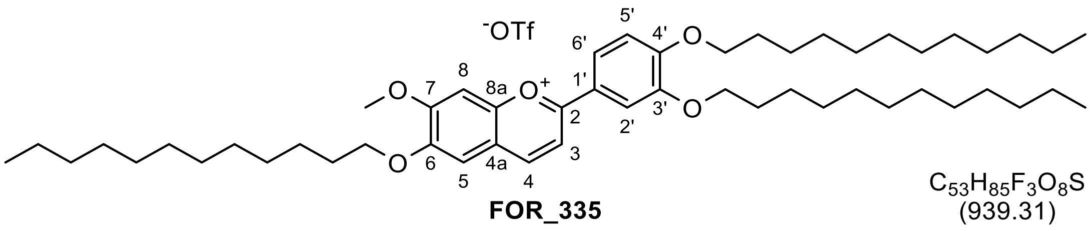

FOR_335

^{1}H NMR (700 MHz, $CDCl_3$): δ = 0.85–0.92 (m, 9H, CH_3), 1.21–1.55 (m, 54H, CH_2), 1.82–1.90 (m, 6H, OCH_2CH_2), 4.02–4.08 (m, 4H, 6-OCH_2, 4'-OCH_2), 4.13 (t, J = 6.3 Hz, 2H, 3'-OCH_2), 4.25 (s, 3H, OCH_3), 6.89 (d, J = 8.7 Hz, 1H, 5'-H), 7.12 (s, 1H, 5-H), 7.59 (d, J = 2.3 Hz, 1H, 2'-H), 7.86 (s, 1H, 8-H), 7.96 (dd, J = 8.7 Hz, 2.3 Hz, 1H, 6'-H), 8.20 (d, J = 8.8 Hz, 1H, 3-H), 8.85 (d, J = 8.8 Hz, 1H, 4-H) ppm. ^{13}C NMR (176 MHz, $CDCl_3$) δ = 14.13 (CH_3), 22.71, 25.94, 25.95, 26.12, 28.84, 29.00, 29.17, 29.39, 29.40, 29.46, 29.58, 29.61, 29.67, 29.69, 29.71, 29.72, 29.75, 29.79, 31.95 (CH_2), 58.17 (OCH_3), 69.53, 69.68, 70.07 (OCH_2), 100.95 (C-8), 106.96 (C-5), 111.28 (C-2'), 112.92 (C-5'), 113.75 (C-3), 119.58 (C-4a), 120.27 (C-1'), 120.96 (q, J = 320.4 Hz, CF_3), 125.50 (C-6'), 150.04 (C-3'), 150.94, 151.02 (C-4', C-6), 154.24 (C-8a), 156.90 (C-4), 160.65 (C-7), 169.05 (C-2) ppm. FT-IR (ATR): $\tilde{\nu}$ = 2920 (s), 2852 (s), 1625 (m), 1594 (m), 1556 (m), 1509 (vs), 1468 (m), 1438 (m), 1396 (s), 1348 (s), 1261 (vs), 1242 (vs), 1224 (vs), 1152 (s), 1127 (s), 1067 (w), 1030 (vs), 1002 (m), 920 (w), 862 (m), 815 (w), 742 (w); 722 (w), 638 (s), 574 (w), 518 (w) cm^{-1}. MS (ESI): m/z = 789.64 $[M]^+$, 621.45 $[M+H - C_{12}H_{25}]^+$. HRMS (ESI) for $[C_{52}H_{85}O_5]^+$: calc.: 789.6392, found.: 789.6392. CHNS analysis: calc.: C 67.77, H 9.12, S 3.41; found.: C 68.00, H 9.26, S 3.11.

6-(Dodecyloxy)-2-[3,4,5-tris(dodecyloxy)phenyl]-7-methoxychromenium triflate (V-Fla-3)

Synthesis according to GP 5: phenol **7b** (50 mg, 162 µmol), ethynylketone **9f** (111 mg, 162 µmol), EtOAc (5 mL), yield: 137 mg, 121 µmol, 75 %, red-brown solid.

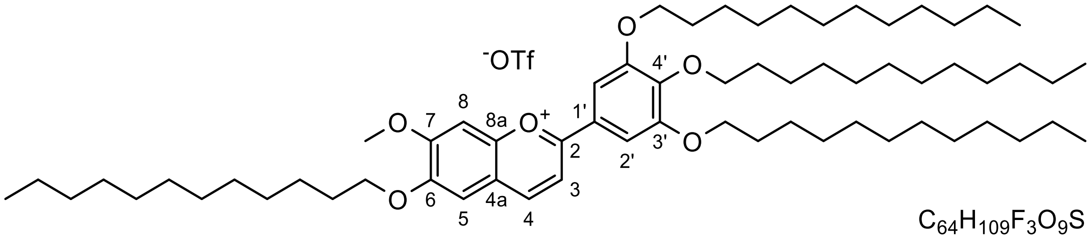

FOR_336

$C_{64}H_{109}F_3O_9S$
(1122.77)

^{1}H NMR (700 MHz, $CDCl_3$): δ = 0.86–0.91 (m, 12H, CH_3), 1.22–1.56 (m, 72H, CH_2), 1.70–1.94 (m, 6H, OCH_2CH_2), 4.05–4.13 (m, 8H, OCH_2CH_2), 4.30 (s, 3H, OCH_3), 6.91 (s, 1H, 5-H), 7.34 (s, 2H, 2'-H), 8.01 (s, 1H, 8-H), 8.34 (d, *J* = 8.7 Hz, 1H, 3-H), 8.64 (d, *J* = 8.7 Hz, 1H, 4-H) ppm. ^{13}C NMR (176 MHz, $CDCl_3$) δ = 14.12 (CH_3), 22.71, 26.05, 26.18, 29.23, 29.41, 29.43, 29.58, 29.60, 29.66, 29.68, 29.72, 29.73, 29.75, 29.77, 29.79, 29.81, 31.96 (CH_2), 58.20 (OCH_3), 69.67, 70.03, 73.98 (OCH_2), 101.25 (C-8), 106.39 (C-5), 106.66 (C-2'), 114.07(C-3), 120.01 (C-4a), 120.91 (q, *J* = 320.2 Hz, CF_3), 122.43 (C-1'), 145.43 (C-4'), 150.95 (C-6), 151.35 (C-4), 153.89 (C-3'), 154.82 (C-8a), 161.22 (C-7), 168.23 (C-2) ppm. FT-IR (ATR): $\tilde{\nu}$ = 2917 (vs), 2850 (vs), 1623 (m), 1581 (m), 1556 (w), 1513 (s), 1491 (s), 1468 (s), 1439 (m), 1413 (m), 1389 (m), 1356 (vs), 1314 (s), 1240 (vs), 1210 (m), 1159 (s), 1128 (vs), 1031 (s), 989 (m), 861 (m), 742 (w), 721 (w), 677 (w), 639 (s), 574 (w), 518 (w) cm^{-1}.MS (ESI): *m*/*z* = 973.82 $[M]^+$, 805.63 $[M+H -C_{12}H_{25}]^+$. HRMS (ESI) for $[C_{64}H_{109}O_6]^+$: calc.: 973.8219, found.: 973.8209. CHNS analysis: calc.: C 69.48, H 9.78, S 2.85; found.: C 69.31, H 9.95, S 2.37.

6-(Dodecyloxy)-2-[2,3,4-tris(dodecyloxy)phenyl]-7-methoxychromeniumtriflate (V-Fla-3')

Synthesis according to GP 5: phenole **7c** (50 mg, 162 µmol), ethynylketone **9e** (111 mg, 162 µmol), EtOAc (5 mL), yield: 100 mg, 89 µmol, 55 %, orange solid.

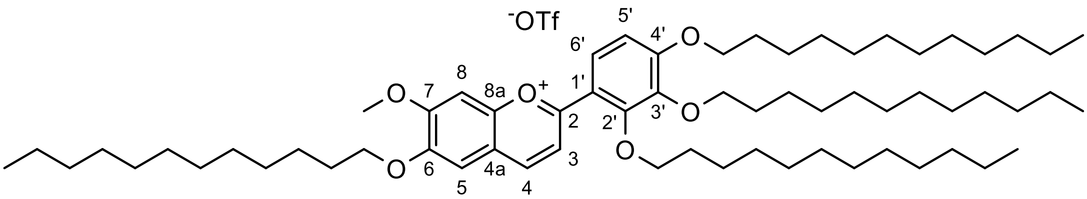

FOR_384

$C_{65}H_{109}F_3O_9S$
(1123.6)

^{1}H NMR (700 MHz, $CDCl_3$): δ = 0.85–0.91 (m, 12H, CH_3), 1.20–1.54 (m, 72H, CH_2), 1.76–1.91 (m, 8H, OCH_2CH_2), 3.99 (t, *J* = 6.6 Hz, 2H, OCH_2, 4'-OCH_2), 4.12–4.16 (m, 4H, 6-OCH_2,

4'-OCH_2), 4.21 (s, 3H, OCH_3), 4.23 (t, *J* = 7.0 Hz, 2H, 2'-OCH_2), 7.00 (d, *J* = 9.3 Hz, 1H, 5'-H), 7.44 (s, 1H, 5-H), 7.87 (s, 1H, 8-H), 8.35 (d, *J* = 9.2 Hz, 1H, 6'-H), 8.50 (d, *J* = 8.8 Hz, 1H, 3-H), 9.00 (d, *J* = 8.8 Hz, 1H, 4-H) ppm. ^{13}C NMR (176 MHz, $CDCl_3$) δ = 14.13 (CH_3), 22.71, 25.86, 25.93, 26.05, 26.16, 28.74, 29.10, 29.37, 29.38, 29.40, 29.56, 29.61, 29.63, 29.65, 29.67, 29.68, 29.70, 29.72, 29.74, 29.75, 29.77, 30.31, 30.35, 31.93, 31.94, 31.95 (CH_2), 58.23 (OCH_3), 69.65, 70.26 (6-OCH_2, 4'-OCH_2), 74.07 (3'-OCH_2), 75.27 (2'-OCH_2), 100.63 (C-8), 107.26 (C-5), 109.99 (C-5'), 115.41 (C-1'), 116.69 (C-3), 120.11 (C-4a), 128.29 (C-6'), 141.80 (C-3'), 151.19, 151.22 (C-4, C-6), 155.12 (C-2'), 155.25 (C-8a), 160.93 (C-4'), 161.21 (C-7), 168.15 (C-2) ppm. FT-IR (ATR): $\tilde{\nu}$ = 2956 (m), 2918 (vs), 2850 (vs), 1622 (m), 1590 (m), 1561 (m), 1514 (vs), 1487 (s), 1465 (s), 1412 (w), 1396 (w), 1338 (vs), 1297 (vs), 1237 (vs), 1210 (vs), 1154 (s), 1129 (m), 1112 (vs), 1079 (s), 1029 (vs), 996 (m), 877 (w), 813 (w), 721 (w), 636 (s), 599 (w), 573 (w), 517 (w) cm^{-1}. MS (ESI): *m*/*z* = 973.82 [M]$^+$. HRMS (ESI) for $[C_{64}H_{109}O_6]^+$: calc.: 973.8219, found.: 973.8226. CHNS analysis: calc.: C 69.48, H 9.78, S 2.85 found.: C 68.84, H 9.76, S 2.75.

7-(Dodecyloxy)-2-phenyl-6-methoxychromenium triflate (iV-Fla-0)

Synthesis according to GP 5: phenol **7c** (100 mg, 324 µmol), ethynylketone **9g** (42 mg, 324 µmol), EtOAc (5 mL), yield: 70 mg, 123 µmol, 38 %, yellow powder.

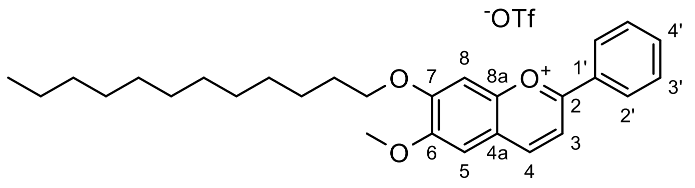

$C_{29}H_{37}F_3O_6S$
(570.66)

FOR_460

^{1}H NMR (700 MHz, $CDCl_3$): δ = 0.85–0.92 (m, 3H, CH_3), 1.22–1.57 (m, 18H, CH_2), 1.94–2.01 (m, 2H, OCH_2CH_2), 4.08 (s, 3H, OCH_3), 4.38 (t, *J* = 6.6 Hz, 2H, OCH_2CH_2), 7.61–7.66 (m, 3H, 5-H, 3'-H), 7.68–7.72 (m, 1H, 4'-H), 7.72 (s, 1H, 8-H), 8.31 (d, *J* = 7.8 Hz, 2H, 2'-H), 8.37 (d, *J* = 8.5 Hz, 1H, 3-H), 9.43 (d, *J* = 8.5 Hz, 1H, 4-H) ppm. ^{13}C NMR (176 MHz, $CDCl_3$) δ = 14.13 (CH_3), 22.69, 25.77, 28.53, 29.28, 29.35, 29.49, 29.59, 29.64, 29.66, 31.92 (CH_2), 57.26 (OCH_3), 71.88 (OCH_2), 100.36 (C-8), 107.34 (C-5), 114.01 (3-H), 120.89 (q, *J* = 320.4 Hz, CF_3), 121.66, 128.54 (C-1'), 128.71 (C-2'), 130.21 (C-3'), 135.68 (C-4'), 152.49 (C-6), 153.85 (C-4), 156.13, 161.97 (C-7), 168.88 ppm. FT-IR (ATR): $\tilde{\nu}$ = 3068 (w), 2923 (m), 2853 (m), 1626 (m), 1599 (w), 1559 (w), 1508 (vs), 1469 (m), 1456 (m), 1409 (m), 1391 (m), 1340 (s),

1318 (m), 1260 (vs), 1240 (vs), 1207 (s), 1182 (m), 1155(s), 1128 (m), 1058 (w), 1030 (vs), 1012 (m), 999 (m), 977 (m), 910 (w), 869 (m), 832 (w), 776 (w), 754 (w), 736 (m), 679 (m), 659 (w), 638 (s), 600 (w), 573 (w), 517 (w), 498 (m) cm^{-1}. MS (ESI): *m*/*z* = 421.27 $[M]^+$, 253.08 $[M+H\ -OC_{12}H_{25}]^+$. HRMS (ESI) for $[C_{28}H_{37}O_3]^+$: calc.: 421.2737, found.: 421.2712. CHNS analysis: calc.: C 61.04, H 6.54, S 5.62; found.: C 61.00, H 6.47, S 5.37.

7-(Dodecyloxy)-2-[4-(dodecyloxy)phenyl]-6-methoxychromenium triflate (iV-Fla-1)

Synthesis according to GP 5: phenol **7c** (50 mg, 162 µmol), ethynylketone **9a** (51 mg, 162 µmol), EtOAc (5 mL), yield: 61 mg, 81 µmol, 50 %, orange solid.

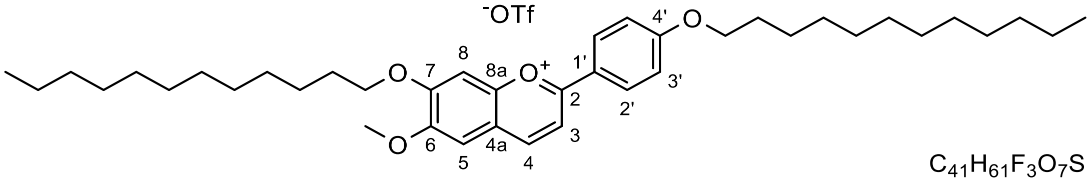

FOR_340

$C_{41}H_{61}F_3O_7S$
(754.99)

^{1}H NMR (500 MHz, $CDCl_3$): δ = 0.84–0.93 (m, 6H, CH_3), 1.19–1.57 (m, 36H, CH_2), 1.78–1.87 (m, 2H, 4'-OCH_2CH_2), 1.89–1.98 (m, 2H, 7-OCH_2CH_2), 4.02 (s, 3H, CH_3), 4.05 (t, *J* = 6.6 Hz, 2H, 4'-OCH_2), 4.34 (t, *J* = 6.7 Hz, 2H, 7-OCH_2), 7.01 (d, *J* = 9.1 Hz, 2H, 3'-H), 7.37 (s, 1H, 5-H), 7.74 (s, 1H, 8-H), 8.17 (d, *J* = 8.8 Hz, 1H, 3-H), 8.26 (d, *J* = 9.1 Hz, 2H, 2'-H), 9.06 (d, *J* = 8.8 Hz, 1H, 4-H) ppm. ^{13}C NMR (126 MHz, $CDCl_3$) δ = 14.12 (CH_3), 22.70, 25.79, 25.92, 28.64, 29.00, 29.37, 29.39, 29.54, 29.58, 29.62, 29.66, 29.69, 31.93 (CH_2), 56.93 (CH_3), 69.15 (4'-OCH_2), 71.60 (7-OCH_2), 100.88 (C-8), 106.97 (C-5), 113.19 (C-3), 116.31 (C-3'), 119.77 (C-4a), 120.30 (C-1'), 122.23 (q, *J* = 320.7 Hz, CF_3) 131.82 (C-2'), 151.67 (C-6), 151.95 (C-4), 154.67 (C-8a), 160.40 (C-7), 166.19 (C-4'), 169.10 (C-2) ppm. FT-IR (ATR): $\tilde{\nu}$ = 2916 (s), 2849 (s), 1626 (m), 1602 (s), 1571 (m), 1557 (w), 1511 (s), 1468 (m), 1443 (m), 1405 (m), 1389 (m), 1351 (s), 1304 (s), 1269 (vs), 1230 (vs), 1208 (s), 1179 (vs), 1157 (vs), 1124 (s), 1067 (m), 1030 (vs), 1014 (m), 988 (m), 952 (m), 914 (w), 879 (m), 834 (m), 747 (w), 719 (w), 638 (s), 598 (w), 574 (w), 517 (w) cm^{-1}. MS (ESI): *m*/*z* = 605.46 $[M]^+$, 437.27 $[M+H\ -C_{12}H_{25}]^+$. HRMS (ESI) for $[C_{40}H_{61}O_4]^+$: calc.: 605.4564, found.: 605.4543. CHNS analysis: calc.: C 65.23, H 8.27, S 3.83; found.: C 65.39, H 8.27, S 3.83.

7-(Dodecyloxy)-2-[3,4-bis(dodecyloxy)phenyl]-6-methoxychromenium triflate (iV-Fla-2)

Synthesis according to GP 5: phenol **7c** (50 mg, 162 µmol), ethynylketone **9d** (81 mg, 162 µmol), EtOAc (5 mL), yield: 108 mg, 115 µmol, 71 %, red solid.

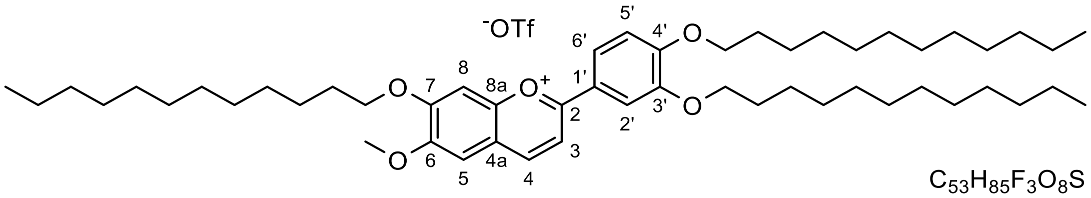

FOR_341

$C_{53}H_{85}F_3O_8S$
(939.31)

^{1}H NMR (500 MHz, $CDCl_3$): δ = 0.85–0.92 (m, 9H CH_3), 1.21–1.58 (m, 54H, CH_2), 1.81–1.91 (m, 4H, 3'-OCH_2CH_2, 4'-OCH_2CH_2), 1.91–1.99 (m, 2H, 7-OCH_2CH_2), 3.99 (s, 3H, CH_3), 4.06 (t, J = 6.6 Hz, 2H, 4'-OCH_2), 4.13 (t, J = 6.3 Hz, 2H, 3'-OCH_2), 4.39 (t, J = 6.6 Hz, 2H, 7-OCH_2), 6.89 (d, J = 8.7 Hz, 1H, 5'-H), 7.17 (s, 1H, 5-H), 7.57 (d, J = 2.3 Hz, 1H, 2'-H), 7.79 (s, 1H, 8-H), 7.95 (dd, J = 8.7 Hz, 2.3 Hz, 1H, 6'-H), 8.22 (d, J = 8.8 Hz, 1H, 3-H), 8.91 (d, J = 8.8 Hz, 1H, 4-H) ppm. ^{13}C NMR (126 MHz, $CDCl_3$) δ = 14.12 (CH_3), 22.71, 25.84, 25.95, 26.12, 28.75, 28.99, 29.19, 29.38, 29.41, 29.44, 29.57, 29.64, 29.67, 29.70, 29.72, 29.74, 29.78, 31.94 (CH_2), 56.76 (OCH_3), 69.50 (4'-OCH_3), 69.69 (3'-OCH_3), 71.60 (7-OCH_2), 101.24 (C-8), 106.54 (C-5), 111.31 (2'-C), 112.92 (5'-C), 113.67 (C-3), 119.46 (C-4a), 120.27 (C-1'), 120.96 (q, J = 320.4 Hz, CF_3), 125.45 (C-6'), 149.97 (C-3'), 151.22 (C-4), 151.51 (C-6), 154.50 (C-8a), 156.83 (C-4'), 160.14 (C-7), 169.00 (C-2) ppm. FT-IR (ATR): $\tilde{\nu}$ = 2917 (s), 2850 (s), 1625 (m), 1594 (w), 1553 (w), 1509 (vs), 1467 (m), 1449 (m), 1421 (w), 1392 (m), 1355 (s), 1309 (m), 1264 (vs), 1238 (vs), 1207 (m), 1179 (m), 1151 (s), 1128 (s), 1066 (w), 1030 (s), 952 (m), 911 (w), 866 (m), 814 (w), 744 (w), 720 (w), 637 (s), 574 (w), 516 (m) cm^{-1}. MS (ESI): m/z = 789.64 $[M]^+$, 621.45 $[M+H -C_{12}H_{25}]^+$. HRMS (ESI) for $[C_{52}H_{85}O_5]^+$: calc.: 789.6392, found.: 789.6402. CHNS analysis: calc.: C 67.77, H 9.12, S 3.41; found.: C 67.42, H 9.22, S 2.98.

7-(Dodecyloxy)-2-[3,4,5-tris(dodecyloxy)phenyl]-6-methoxychromenium triflate (iV-Fla-3)

Synthesis according to GP 5: phenol **7c** (50 mg, 162 µmol), ethynylketone **9f** (111 mg, 162 µmol), EtOAc (5 mL), yield: 131 mg, 116 µmol, 72 %, red-brown solid.

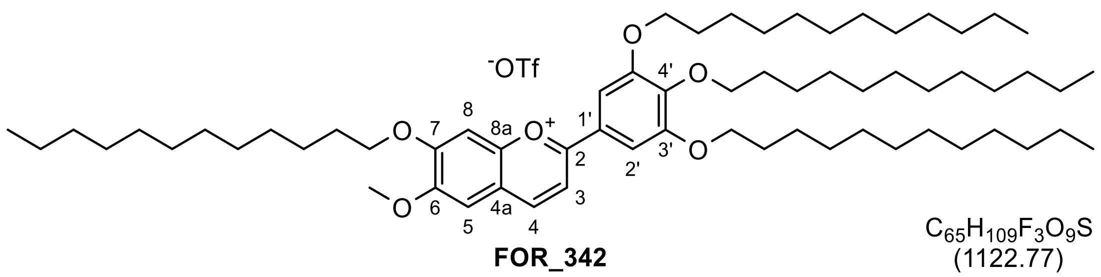

^{1}H NMR (500 MHz, $CDCl_3$): δ = 0.85–0.92 (m, 12H, CH_3), 1.21–1.58 (m, 72H, CH_2), 1.72–2.00 (m, 8H, OCH_2CH_2), 4.02 (s, 3H, OCH_3), 4.07–4.14 (m, 6H, 3'-OCH_2, 4'-OCH_2), 4.44 (t, J = 6.6 Hz, 2H, 7-OCH_2), 7.11 (s, 1H, 5-H), 7.36 (s, 2H, 2'-H), 7.85 (s, 1H, 8-H), 8.34 (d, J = 8.7 Hz, 1H, 3-H), 8.80 (d, J = 8.7 Hz, 1H, 4-H) ppm. ^{13}C NMR (126 MHz, $CDCl_3$) δ = 14.12 (CH_3), 22.71, 25.84, 26.03, 26.19, 28.78, 29.29, 29.38, 29.41, 29.56, 29.58, 29.60, 29.64, 29.67, 29.72, 29.78, 30.50, 31.94, 31.96 (CH_2), 56.81 (OCH_3) (OCH_3), 69.73 (3'-OCH_2), 71.64 (7-OCH_2), 74.00 (4'-OCH_2), 101.21 (C-8), 106.40 (C-5), 106.86 (C-2'), 114.12 (C-3), 120.04 (C-4a), 122.54 (C-1'), 145.59 (C-4'), 151.37 (C-4), 151.88 (C-6), 153.89 (C-3'), 155.06 (C-8a), 160.74 (C-7), 168.50 (C-2) ppm. FT-IR (ATR): $\tilde{\nu}$ = 2916 (s), 2850 (s), 1624 (w), 1580 (w), 1556 (w), 1514 (s), 1489 (s), 1468 (m), 1468 (m), 1447 (m), 1410 (m), 1388 (m), 1355 (vs), 1311 (s), 1239 (vs), 1207 (s), 1179 (m), 1159 (s), 1128 (vs), 1031 (s), 1015 (m), 966 (m), 861 (m), 757 (w), 742 (w), 720 (w), 678 (w), 638 (s), 601 (w), 574 (w), 516 (m) cm^{-1}. MS (ESI): m/z = 973.82 $[M]^+$. HRMS (ESI) for $[C_{64}H_{109}O_6]^+$: calc.: 973.8219, found.: 973.8202. CHNS analysis: calc.: C 96.48, H 9.78, S 2.85; found.: C 96.03, H 9.85, S 2.54.

7-(Dodecyloxy)-2-[2,3,4-tris(dodecyloxy)phenyl]-6-methoxychromeniumtriflate (iV-Fla-3')

Synthesis according to GP 5: phenol **7c** (50 mg, 162 µmol), ethynylketone **9e** (111 mg, 162 µmol), EtOAc (5 mL), yield: 134 mg, 119 µmol, 74 %, orange solid.

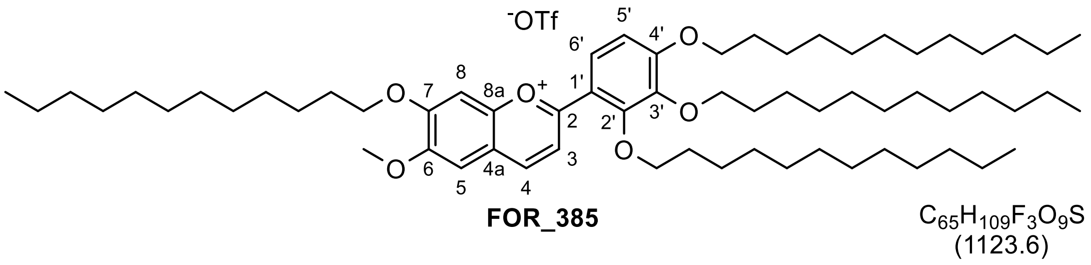

^{1}H NMR (500 MHz, $CDCl_3$): δ = 0.83–0.92 (m, 12H, CH_3), 1.19–1.55 (m, 72H, CH_2), 1.75–1.95 (m, 10H, $OCH_2C\mathit{H}_2$), 4.00 (t, *J* = 6.6 Hz, 2H, 3'-OCH_2), 4.06 (s, 3H, OCH_3), 4.14 (t, *J* = 6.5 Hz, 2H, 4'-OCH_2), 4.23 (t, *J* = 7.0 Hz, 2H, 2'-OCH_2), 4.32 (t, *J* = 6.7 Hz, 2H, 7-OCH_2), 6.99 (d, *J* = 9.2 Hz, 1H, 5'-H), 7.59 (s, 1H, 5-H), 7.76 (s, 1H, 8-H), 8.29 (d, *J* = 9.2 Hz, 1H, 6'-H), 8.50 (d, *J* = 8.8 Hz, 1H, 3-H), 9.10 (d, *J* = 8.9 Hz, 1H, 4-H) ppm. ^{13}C NMR (126 MHz, $CDCl_3$) δ = 14.12 (CH_3), 22.70, 25.75, 25.93, 26.05, 26.15, 28.59, 29.10, 29.35, 29.37, 29.39, 29.41, 29.52, 29.60, 29.63, 29.65, 29.68, 29.71, 29.74, 29.77, 30.31, 30.36, 31.93, 31.95 (CH_2), 57.15 (OCH_3), 69.63 (4'-OCH_2), 71.60 (7-OCH_2), 74.10 (3'-OCH_2), 75.29 (2'-OCH_2), 100.70 (C-8), 107.13 (C-5), 109.92 (C-5'), 115.48 (C-1'), 116.60 (C-3), 120.09 (C-4a), 128.03 (C-6'), 141.88 (C-3'), 151.55 (C-4), 151.84 (C-6), 155.10 (C-2'), 155.44 (C-8a), 160.63 (C-7), 160.84 (C-4'), 168.05 (C-2) ppm. FT-IR (ATR): $\tilde{\nu}$ = 2956 (m), 2919 (vs), 2851 (vs), 1624 (m), 1590 (m), 1563 (m), 1516 (vs), 1487 (s), 1466 (s), 1405 (m), 1390 (m), 1339 (vs), 1298 (vs), 1262 (vs), 1239 (s), 1208 (s), 1176 (m), 1157 (m), 1113 (s), 1079 (m), 1029 (m), 875 (w), 814 (w), 721 (w), 637 (s), 598 (w), 573 (w), 517 (w) cm^{-1}. MS (ESI): *m*/*z* = 1027.83 $[M+OCH_3+Na]^+$, 973.82 $[M]^+$. HRMS (ESI) for $[C_{64}H_{109}O_6]^+$: calc.: 973.8219, found.: 973.8217. CHNS analysis: calc.: C 69.48, H 9.78, S 2.85; found.: C 69.47, H 9.84, S 2.18.

6,7-Bis(dodecyloxy)-2-phenyl-6-methoxychromenium triflate (2-Fla-0)

Synthesis according to GP 5: phenol **7d** (100 mg, 216 µmol), ethynylketone **9g** (28 mg, 216 µmol), EtOAc (5 mL), yield: 119 mg, 164 µmol, 76 %, yellow powder.

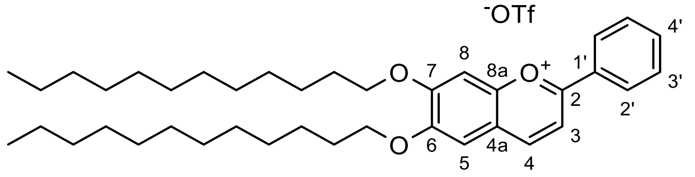

FOR_461

$C_{40}H_{59}F_3O_6S$
(724.96)

^{1}H NMR (700 MHz, $CDCl_3$): δ = 0.88 (t, *J* = 7.0 Hz, 6H, CH_3), 1.22–1.58 (m, 36H, CH_2), 1.87–1.99 (m, 4H, OCH_2CH_2), 4.18 (t, *J* = 6.4 Hz, 2H, X-OCH_2), 4.38 (t, *J* = 6.4 Hz, 2H, X-OCH_2), 7.50 (s, 1H, 5-H), 7.61 (t, *J* = 7.7 Hz, 2H, 3'-H), 7.65–7.70 (m, 1H, 4'-H), 7.71 (s, 1H, 8-H), 8.28–8.31 (m, 2H, 2'-H), 8.37 (d, *J* = 8.5 Hz, 1H, 3-H), 9.37 (d, *J* = 8.5 Hz, 1H, 4-H) ppm. ^{13}C NMR (176 MHz, $CDCl_3$) δ = 14.13 (CH_3), 22.70, 25.84, 25.96, 28.54, 28.70, 29.30, 29.37, 29.39, 29.58, 29.63, 29.64, 29.68, 29.69, 29.71, 29.73, 31.94 (CH_2), 70.28, 71.67 (OCH_2),

100.31 (C-8), 107.47 (C-5), 113.97 (C-3), 120.91 (q, *J* = 320.7 Hz, CF_3), 121.70 (C-4a), 128.58 (C-1'), 128.65 (C-1'), 130.17 (C-3'), 135.52 (C-4'), 152.07 (C-6), 153.53 (C-4), 156.05 (C-8a), 162.25 (C-7), 168.58 (C-2) ppm. FT-IR (ATR): $\tilde{\nu}$ = 3065 (w), 2957 (m), 2916 (vs), 2848 (s), 1626 (m), 1560 (w), 1523 (s), 1467 (m), 1410 (m); 1393 (m), 1342 (s), 1319 (m), 1268 (vs), 1246 (vs), 1196 (s), 1151 (s), 1068 (w), 1030 (vs), 986 (w), 947 (w), 907 (w), 870 (m), 841 (w), 807 (w), 775 (w), 754 (w), 738 (w), 724 (m), 677 (m), 637 (s), 599 (w), 572 (w), 554 (w), 517 (m) cm^{-1}. MS (ESI): *m/z* = 575.44 $[M]^+$. HRMS (ESI) for $[C_{39}H_{59}O_3]^+$: calc.: 575.4459, found.: 575.4421. CHNS analysis: calc.: C 66.27, H 8.20, S 4.42; found.: C 66.13, H 8.04, S 3.89.

6,7-Bis(dodecyloxy)-2-[4-(dodecyloxy)phenyl]chromenium triflate (2-Fla-1)

Synthesis according to GP 5: phenol **7d** (74 mg, 160 µmol), ethynylketone **9a** (51 mg, 160 µmol), EtOAc (5 mL), yield: 129 mg, 142 µmol, 89 %, orange solid.

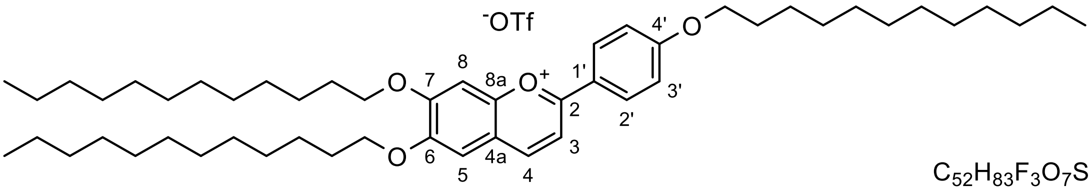

FOR_337

$C_{52}H_{83}F_3O_7S$
(909.28)

^{1}H NMR (700 MHz, $CDCl_3$): δ = 0.85–0.91 (m, 9H, CH_3), 1.21–1.57 (m, 54H, CH_2), 1.79–1.97 (m, 6H, OCH_2CH_2), 4.04 (t, *J* = 6.6 Hz, 2H, 4'-OCH_2), 4.11 (t, *J* = 6.5 Hz, 2H, 6-OCH_2), 4.33 (t, *J* = 6.5 Hz, 2H, 7-OCH_2), 7.01 (d, *J* = 8.9 Hz, 2H, 3'-H), 7.29 (s, 1H, 5-H), 7.68 (s, 1H, 8-H), 8.20 (d, *J* = 8.7 Hz, 1H, 3-H), 8.26 (d, *J* = 8.9 Hz, 2H, 2'-H), 9.02 (d, *J* = 8.7 Hz, 4-H) ppm. ^{13}C NMR (176 MHz, $CDCl_3$) δ = 14.13 (CH_3), 22.71, 25.86, 25.93, 25.97, 28.63, 28.80, 29.01, 29.36, 29.37, 29.40, 29.42, 29.59, 29.62, 29.63, 29.65, 29.67, 29.70, 29.74, 29.75, 31.93, 31.94 (CH_2), 69.11 (4'-OCH_2), 69.98 (6-OCH_2), 71.33 (7-OCH_2), 100.64 (C-8), 107.32 (C-5), 113.24 (C-3), 116.28 (C-3'), 119.88 (C-4a), 120.39 (C-1'), 120.89 (q, *J* = 319.1 Hz, CF_3), 131.70 (C-2'), 151.29 (C-6), 151.80 (C-4), 154.60 (C-8a), 160.70 (C-7), 166.07 (C-4'), 168.92 (C-2) ppm. FT-IR (ATR): $\tilde{\nu}$ = 3500 (w, br.), 3057 (w, br.), 2955 (m), 2919 (vs), 2851 (s), 1625 (m), 1604 (m), 1556 (m), 1531 (m), 1512 (vs), 1467 (m), 1441 (w), 1406 (s), 1352 (s), 1323 (m), 1306 (m), 1269 (vs), 1242 (vs), 1190 (s), 1158 (s), 1127 (m), 1030 (s), 1005 (w), 956 (w), 912 (w), 872 (w), 844 (w), 747 (w), 638 (s), 599 (w), 574 (w), 553 (w), 518 (w) cm^{-1}. MS (ESI): *m/z* = 813.62 $[M+OCH_3+Na]^+$, 759.62 $[M]^+$. HRMS (ESI) for $[C_{51}H_{83}O_4]^+$: calc.: 759.6286, found.: 759.6279. CHNS analysis: calc.: C 68.69, H 9.20, S 3.53; found.: C 68.41, H 9.14, S 3.23.

6,7-Bis(dodecyloxy)-2-[3,4-bis(dodecyloxy)phenyl]chromenium triflate (2-Fla-2)

Synthesis according to GP 5: phenol **7d** (50 mg, 108 µmol), ethynylketone **9d** (54 mg, 108 µmol), EtOAc (5 mL), yield: 107 mg, 98 µmol, 91 %, red powder.

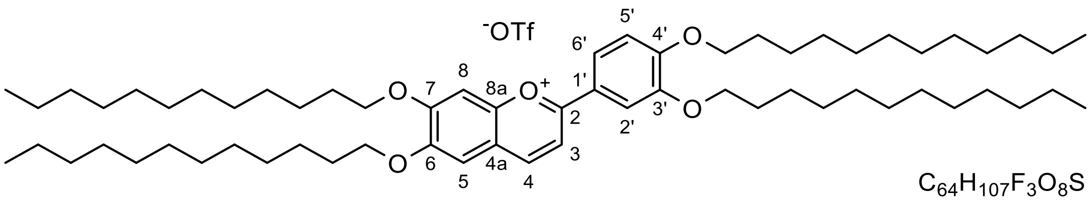

FOR_324

$C_{64}H_{107}F_3O_8S$
(1093.61)

^{1}H NMR (700 MHz, $CDCl_3$): δ = 0.85–0.91 (m, 12H, CH_3), 1.21–1.44 (m, 72H, CH_2), 1.46–1.58 (m, 8H, $OCH_2C\mathit{H}_2$), 1.81–1.98 (m, 8H, $OC\mathit{H}_2CH_2$), 4.04–4.12 (m, 4H, OCH_2, 6-OCH_2, 4'-OCH_2), 4.13 (t, *J* = 6.3 Hz, 2H, 3'-OCH_2), 4.37 (t, *J* = 6.5 Hz, 2H, 7-OCH_2), 6.93 (d, *J* = 8.6 Hz, 1H, 5'-H), 7.19 (s, 1H, 5-H), 7.60 (d, *J* = 2.3 Hz, 1H, 2'-H), 7.69 (s, 1H, 8-H), 7.97 (dd, *J* = 8.6 Hz, 2.3 Hz, 1H, 6'-H), 8.27 (d, *J* = 8.7 Hz, 1H, 3-H), 8.95 (d, *J* = 8.7 Hz, 1H, 4-H) ppm. ^{13}C NMR (176 MHz, $CDCl_3$) δ = 14.13 (CH_3), 22.71, 25.90, 25.96, 26.00, 26.12, 28.70, 28.83, 28.99, 29.20, 29.39, 29.40, 29.42, 29.45, 29.57, 29.64, 29.66, 29.68, 29.69, 29.71, 29.72, 29.75, 29.76, 29.79, 31.95 (CH_2), 69.50, 69.72, 69.92 (7-OCH_2, 3'-OCH_2, 4'-OCH_2), 71.28 (7-OCH_2), 100.80 (C-8), 107.17 (C-5), 111.44 (C-2'), 113.01 (C-5'), 113.79 (C-3), 119.73 (C-4a), 120.48 (C-1'), 120.66 (d, *J* = 320.8 Hz, CF_3), 125.28 (C-6'), 150.04, 151.22 (C-3', C-4), 151.33 (C-6), 154.49 (C-8a), 156.80 (C-4'), 160.49 (C-7), 168.99 (C-2) ppm. FT-IR (ATR): $\tilde{\nu}$ = 2955 (w), 2916 (s), 2848 (s), 1622 (w), 1594 (w), 1556 (w), 1511 (vs), 1469 (m), 1446 (m), 1418 (w), 1400 (m), 1359 (s), 1318 (m), 1300 (s), 1274 (s), 1257 (vs), 1200 (m), 1162 (s), 1149 (s), 1136 (s), 1067 (m), 1029 (m), 991 (w), 949 (w), 909 (w), 875 (w), 856 (w), 817 (w), 720 (w), 638 (m), 604 (w), 574 (w), 518 (w) cm^{-1}. MS (ESI): *m*/*z* = 943.81 $[M]^+$. HRMS (ESI) for $[C_{63}H_{107}O_5]^+$: calc.: 943.8113, found.: 943.8116. CHNS analysis: calc.: C 70.29, H 9.86, S 2.93; found.: C 69.83, H 9.86, S 2.79.

6,7-Bis(dodecyloxy)-2-[3,4,5-bis(dodecyloxy)phenyl]chromenium triflate (2-Fla-3)

Synthesis according to GP 5: phenol **7d** (50 mg, 108 µmol), ethynylketone **7f** (74 mg, 108 µmol), EtOAc (5 mL), yield: 112 mg, 88 µmol, 81 %, red solid.

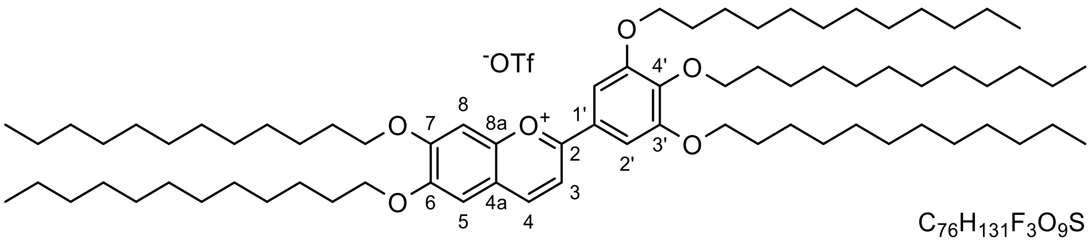

FOR_339

$C_{76}H_{131}F_3O_9S$
(1277.93)

^{1}H NMR (700 MHz, $CDCl_3$): δ = 0.85–0.92 (m, 15H, CH_3), 1.22–1.59 (m, 90H, CH_2), 1.71–1.97 (m, 10H, OCH_2CH_2), 4.03–4.13 (m, 8H, OCH_2CH_2, 6-OCH_2, 3'-OCH_2, 4'-OCH_2), 4.45 (t, *J* = 6.4 Hz, 2H, 7-OCH_2), 6.93 (s, 1H, 5-H), 7.32 (s, 2H, 2'-H), 7.90 (s, 1H, 8-H), 8.30 (d, *J* = 8.7 Hz, 1H, 3-H), 8.68 (d, *J* = 8.7 Hz, 1H, 4-H) ppm. ^{13}C NMR (176 MHz, $CDCl_3$) δ = 14.13 (CH_3), 22.72, 25.93, 26.06, 26.10, 26.23, 28.79, 28.93, 29.29, 29.42, 29.44, 29.46, 29.58, 29.63, 29.66, 29.69, 29.72, 29.74, 29.76, 29.77, 29.80, 29.82, 30.55, 31.96 (CH_2), 69.63 (3'-OCH_2), 69.79 (6-OCH_2), 71.41 (7-OCH_2), 73.94 (4'-OCH_2), 101.32 (C-8), 106.42 (C-5), 106.55 (C-2'), 113.85 (C-3), 120.02 (C-4a), 122.59 (C-1'), 145.24 (C-4'), 150.92 (C-4), 151.47 (C-6), 153.89 (C-3'), 154.99 (C-8a), 161.00 (C-7), 168.00 (C-2) ppm. FT-IR (ATR): $\tilde{\nu}$ = 2910 (vs), 2850 (vs), 1623 (w), 1581 (w), 1556 (w), 1513 (s), 1491 (s), 1469 (m), 1446 (m), 1410 (m), 1388 (m), 1357 (vs), 1315 (m), 1242 (vs), 1197 (m), 1160 (m), 1131 (s), 1032 (m), 973 (w), 860 (w), 721 (w), 639 (m), 574 (w), 518 (w) cm^{-1}. MS (ESI): *m*/*z* = 1127.97 $[M]^+$. HRMS (ESI) for $[C_{75}H_{131}O_6]^+$: calc.: 1127.9940, found.: 1127.9917. CHNS analysis: calc.: C 71.43, H 10.33, S 2.51; found.: C 71.22, H 10.20, S 2.28.

6,7-Bis(dodecyloxy)-2-[2,3,4-tris(dodecyloxy)phenyl]chromenium triflate (2-Fla-3')

Synthesis according to GP 5: phenol **7d** (68 mg, 146 µmol), ethynylketone **9e** (100 mg, 146 µmol), EtOAc (5 mL), yield: 155 mg, 121 µmol, 83 %, orange solid.

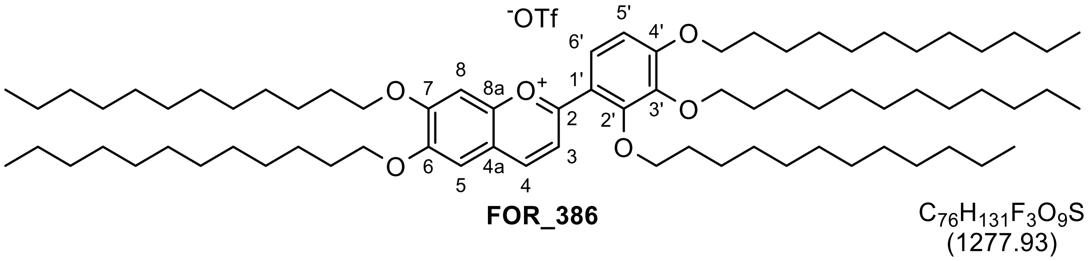

FOR_386

$C_{76}H_{131}F_3O_9S$
(1277.93)

^{1}H NMR (700 MHz, $CDCl_3$): δ = 0.84–0.91 (m, 15H, CH_3), 1.19–1.56 (m, 90H, CH_2), 1.75–1.96 (m, 10H, OCH_2CH_2), 3.99 (t, *J* = 6.6 Hz, 2H, 3'-OCH_2), 4.14 (t, *J* = 6.5 Hz, 2H, 4'-OCH_2),

4.17 (t, J = 6.4 Hz, 2H, 6-OCH_2), 4.22 (t, J = 7.0 Hz, 2H, 2'-OCH_2), 4.34 (t, J = 6.5 Hz, 2H, 7-OCH_2), 6.99 (d, J = 9.2 Hz, 1H, 5'-H), 7.45 (s, 1H, 5-H), 7.74 (s, 1H, 8-H), 8.25 (d, J = 9.2 Hz, 1H, 6'-H), 8.50 (d, J = 8.8 Hz, 1H, 3-H), 9.01 (d, J = 8.8 Hz, 1H, 4-H) ppm. ^{13}C NMR (176 MHz, $CDCl_3$) δ = 14.13 (CH_3), 22.70, 25.84, 25.94, 25.95, 26.05, 26.15, 28.61, 28.77, 29.10, 29.34, 29.37, 29.39, 29.41, 29.60, 29.63, 29.65, 29.67, 29.69, 29.71, 29.73, 29.77, 30.31, 30.35, 31.93, 31.94 (CH_2), 69.59 (4'-OCH_2), 70.09 (6-OCH_2), 71.37 (7-OCH_2), 74.07 (3'-OCH_2), 75.25 (2'-OCH_2), 100.60 (C-8), 107.30 (C-5), 109.89 (5'-H), 115.60 (1'-H), 116.62 (3-H), 120.19 (C-4a), 127.98 (6'-H), 141.86 (3'-H), 151.14 (4-H), 151.53 (6-H), 154.94 (2'-H), 155.50 (C-8a), 160.69 (4'-H), 161.06 (7-H), 167.97 (C-2) ppm. FT-IR (ATR): $\tilde{\nu}$ = 2956 (w), 2916 (vs), 289 (vs), 1622 (m), 1590 (m), 1563 (m), 1515 (vs), 1487 (s), 1467 (s), 1406 (s), 1390 (m), 1337 (vs), 1298 (vs), 1274 (vs), 1240 (vs), 1195 (s), 1156 (m), 1114 (s), 1079 (m), 1029 (s), 951 (w), 910 (w), 874 (w), 814 (w), 721 (w), 637 (s), 600 (w), 573 (w), 554 (w), 518 (w) cm^{-1}. MS (ESI): m/z = 1182.00 $[M+OCH_3+Na]^+$, 1127.99 $[M]^+$. HRMS (ESI) for $[C_{75}H_{131}O_6]^+$: calc.: 1127.9940, found.: 1127.9945. CHNS analysis: calc.: C 71.43, H 10.33, S 2.51; found.: C 70.09, H 10.32, S 2.11.

5,6,7-Tris(dodecyloxy)-2-[4- (dodecyloxy)phenyl]chromenium triflat (3-Fla-1)

Synthesis according to GP 5: phenole **7f** (200 mg, 310 µmol), ethynylketone **7a** (97 mg, 310 µmol), EtOAc (5 mL), yield: 71 mg, 64.9 µmol, 21 %, yellow solid.

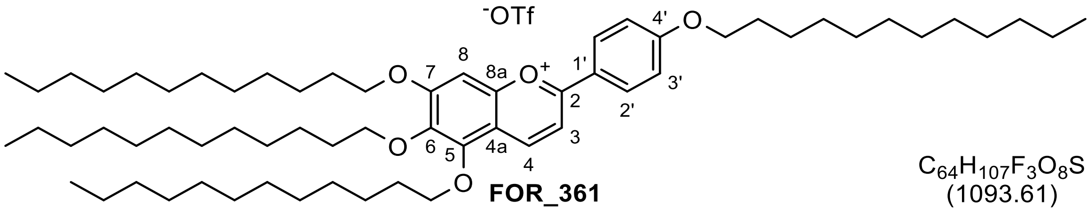

$C_{64}H_{107}F_3O_8S$
(1093.61)

^{1}H NMR (700 MHz, $CDCl_3$): δ = 0.84–0.92 (m, 12H, CH_3), 1.22–1.59 (m, 72H, CH_2), 1.77–2.00 (m, 8H, OCH_2OCH_2), 4.03–4.07 (m, 4H, 6-OCH_2, 4'-OCH_2), 4.36 (t, J = 6.8 Hz, 2H, 5-OCH_2), 4.40 (t, J = 6.4 Hz, 2H, 7-OCH_2), 7.00 (d, J = 8.7 Hz, 2H, 3'-H), 7.71 (s, 1H, 8-H), 8.28 (d, J = 8.8 Hz, 1H, 2'-H), 8.42 (d, J = 8.8 Hz, 2H, 3-H), 8.95 (d, J = 8.8 Hz, 1H, 4-H) ppm. ^{13}C NMR (176 MHz, $CDCl_3$) δ = 14.13 (CH_3), 22.71, 25.89, 25.92, 26.03, 26.05, 28.88, 28.97, 29.39, 29.41, 29.43, 29.55, 29.60, 29.62, 29.64, 29.66, 29.67, 29.69, 29.70, 29.72, 29.73, 29.76, 30.20, 30.23, 31.94, 31.95 (CH_2), 69.24 (4'-OCH_2), 71.67 (7-OCH_2), 74.66 (6-OCH_2), 75.46 (5-OCH_2), 97.36 (C-8), 112.35 (C-3), 115.78 (C-4a), 116.40 (C-3'), 120.26 (C-1'), 132.78 (C-

2'), 140.94 (C-6), 148.07 (C-5), 148.37 (C-4), 154.40 (C-8a), 164.94 (C-7), 166.71 (C-4'), 170.84 (C-2) ppm. FT-IR (ATR): $\tilde{\nu}$ = 2922 (vs), 2853 (s), 1627 (m), 1602 (m), 1566 (w), 1549 (m), 1530 (m), 1502 (s), 1468 (m), 1439 (w), 1382 (m), 1349 (s), 1316 (m), 1276 (s), 1237 (vs), 1181 (s), 1158 (m), 1099 (w), 1030 (s), 838 (w), 721 (w), 639 (m), 518 (w) cm^{-1}. MS (ESI): *m/z* = 997.81 $[M+OCH_3+Na]^+$, 943.81 $[M]^+$. HRMS (ESI) for $[C_{63}H_{107}O_5]^+$: calc.: 943.8113, found.: 943.8111. CHNS analysis: calc.: C 70.29, H 70.10, S 2.19; found.: C 70.10, H 9.93, S 2.19.

5,6,7-Tris(dodecyloxy)-2-[3,4-bis(dodecyloxy)phenyl]chromenium triflate (3-Fla-2)

Synthesis according to GP 5: phenol **7f** (50 mg, 77 µmol), ethynylketone **9d** (39 mg, 77 µmol), EtOAc (5 mL), yield: 20 mg, 15.6 µmol, 20 %, red waxy solid.

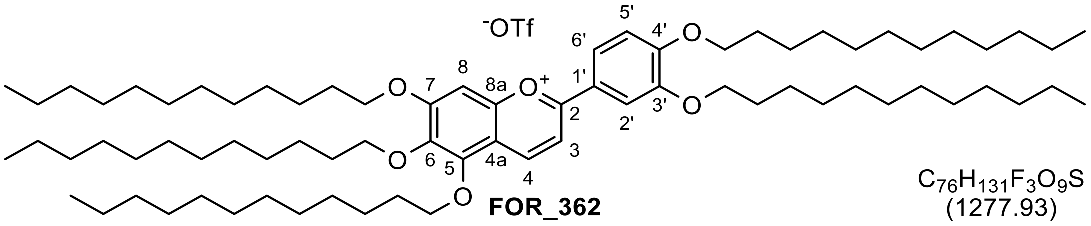

^1H NMR (700 MHz, $CDCl_3$): δ = 0.86–0.91 (m, 15H, CH_3), 1.19–1.60 (m, 90H, CH_2), 1.76–1.99 (m, 10H, $OCH_2C\mathit{H}_2$), 4.00 (t, *J* = 6.6 Hz, 2H, 4'-OCH_2), 4.06–4.12 (m, 4H, 3'-OCH_2, 6-OCH_2), 4.32 (t, *J* = 6.8 Hz, 2H, 5-OCH_2), 4.43 (t, *J* = 6.3 Hz, 2H, 7-OCH_2), 6.87 (d, *J* = 8.7 Hz, 1H, 5'-H), 7.60 (d, *J* = 2.2 Hz, 1H, 2'-H), 7.75 (s, 1H, 8-H), 8.16 (dd, *J* = 8.7 Hz, 2.2 Hz, 1H, 6'-H), 8.38 (d, *J* = 8.8 Hz, 1H, 3-H), 8.77 (d, *J* = 8.8 Hz, 1H, 4-H) ppm. ^{13}C NMR (176 MHz, $CDCl_3$) δ = 14.13 (CH_3), 22.71, 25.91, 25.95, 26.08, 26.11, 26.13, 28.97, 29.00, 29.18, 29.37, 29.40, 29.42, 29.47, 29.51, 29.53, 29.64, 29.65, 29.67, 29.73, 29.74, 29.76, 29.78, 29.80, 29.81, 30.25, 30.34, 31.95, 31.96 (CH_2), 69.45 (4'-OCH_2), 69.65 (3'-OCH_2), 71.62 (7-OCH_2), 74.45 (6-OCH_2), 75.32 (5-OCH_2), 97.61 (C-8), 111.40 (C-2'), 112.83 (C-5'), 112.88 (C-3), 115.48 (C-4a), 120.03 (C-1'), 126.79 (C-6'), 140.96 (C-6), 147.23 (C-4), 148.07 (C-5), 149.64 (C-3'), 154.07 (C-8a), 157.03 (C-4'), 164.53 (C-7), 170.22 (C-2) ppm. FT-IR (ATR): $\tilde{\nu}$ = 2922 (vs), 2853 (s), 1625 (m), 1592 (w), 1571 (w), 1548 (m), 1502 (vs), 1467 (m), 1443 (m), 1383 (m), 1339 (m), 1295 (s), 1256 (s), 1240 (vs), 1225 (s), 1155 (s), 1067 (m), 1029 (vs), 852 (w), 804 (m), 756 (w), 722 (w), 638 (s), 517 (w) cm^{-1}. MS (ESI): *m/z* = 1182.00 $[M+OCH_3+Na]^+$, 1167.99 $[M+OH+Na]^+$, 1149.97 $[M –H +Na]^+$, 1127.99 $[M]^+$. HRMS (ESI) for $[C_{75}H_{131}O_6]^+$: calc.: 1127.9940, found.:1127.9941. CHNS analysis: calc.: C 71.43, H 10.33, S 2.51; found.: C 71.15, H 10.43, S 2.22.

5,6,7-Tris(dodecyloxy)-2-[3,4,5-tris(dodecyloxy)phenyl]chromenium triflate (3-Fla-3)

Synthesis according to GP 5: phenol **7e** (50 mg, 77 µmol), thynylketon **7e** (53 mg, 77 µmol), EtOAc (5 mL), yield: 38 mg, 26.0 µmol, 34 %, red needles.

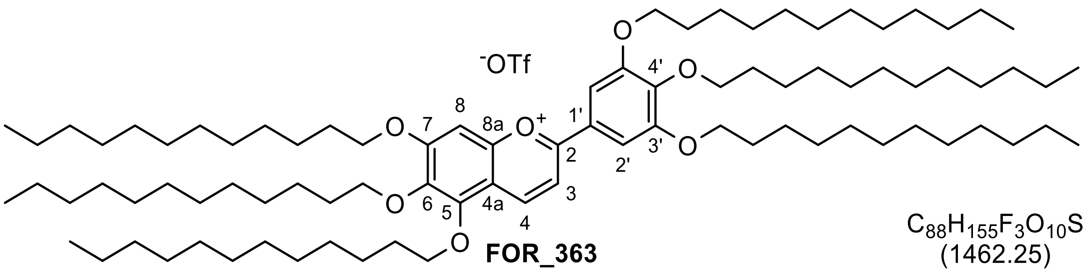

^{1}H-NMR (700 MHz, $CDCl_3$): δ = 0.85–0.92 (m, 18H, CH_3), 1.21–1.60 (m, 108H, CH_2), 1.70–1.99 (m, 12H, OCH_2OCH_2), 4.08–4.17 (m, 8H, 3'-OCH_2, 4'-OCH_2, 5'-OCH_2, 6-OCH_2), 4.39 (t, *J* = 6.9 Hz, 2H, 5-OCH_2), 4.43 (t, *J* = 6.3 Hz, 2H, 7-OCH_2), 7.51 (s, 2H), 7.66 (s, 1H), 8.47 (d, *J* = 8.8 Hz, 1H), 8.95 (d, *J* = 8.8 Hz, 1H) ppm.

^{13}C-NMR (176 MHz, $CDCl_3$) δ = 14.13 (CH_3), 22.71, 25.88, 26.03, 26.08, 26.09, 26.23, 28.96, 29.35, 29.40, 29.41, 29.43, 29.45, 29.49, 29.59, 29.62, 29.64, 29.67, 29.70, 29.71, 29.73, 29.74, 29.76, 29.78, 29.80, 29.80, 30.18, 30.26, 30.51, 31.95, 31.96 (CH_2), 69.93 (3'-OCH_2), 71.67 (7-OCH_2), 74.05 (4'-OCH_2), 74.79 (6-OCH_2), 75.22 (5-OCH_2), 96.91 (C-8), 107.80 (C-2'), 113.31 (C-3), 116.25 (C-4a), 122.38 (C-1'), 140.65 (C-6), 146.40 (C-4'), 148.25 (C-5), 148.43 (C-4), 153.82 (C-3'), 154.79 (C-8), 165.45 (C-7), 170.31(C-2) ppm. FT-IR (ATR): $\tilde{\nu}$ = 2921 (vs), 2852 (s), 1626 (m), 1578 (m), 1545 (w), 1528 (w), 1493 (s), 1468 (m), 1442 (m), 1410 (w), 1347 (vs), 1298 (m), 1257 (vs), 1237 (vs), 1161 (m), 1141 (m), 1077 (w), 1029 (s), 849 (w), 721 (w), 638 (m), 573 (w), 518 (w) cm^{-1}. MS (ESI): *m*/*z* = 1367.17 $[M+OCH_3+Na]^+$, 1312.18 $[M^+]$. HRMS (ESI) for $[C_{87}H_{155}O_7]^+$: calc.: 1312.1767, found.: 1312.1761. CHNS analysis: calc.: C 72.28, H 10.68, S 2.19; found.: C 72.71, H 10.93, S 1.72.

5,6,7-Tris(dodecyloxy)-2-[2,3,4-tris(dodecyloxy)phenyl]chromenium triflate (3-Fla-i3)

Synthesis according to GP 5: phenol **7f** (38 mg, 56 µmol), ethynylketone **9e** (40 mg, 56 µmol), EtOAc (5 mL), yield: 64 mg, 43.8 µmol, 75 %, red solid.

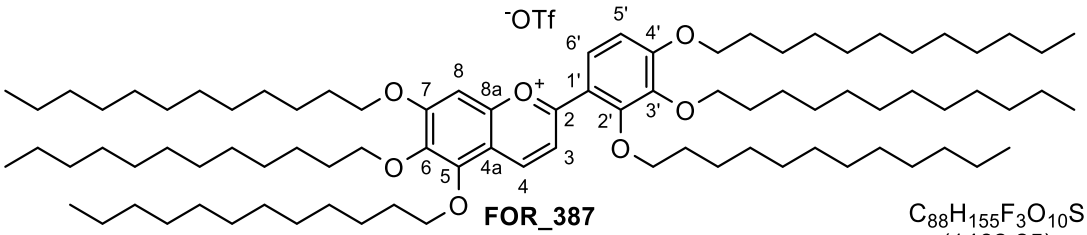

$C_{88}H_{155}F_3O_{10}S$
(1462.25)

^{1}H NMR (700 MHz, $CDCl_3$): δ = 0.82–0.93 (m, 18H, CH_3), 1.16–1.57 (m, 108H, CH_2), 1.74–1.97 (m, 12H, OCH_2CH_2), 3.99 (t, *J* = 6.6 Hz, 2H, 3'-OCH_2), 4.05 (t, *J* = 6.6 Hz, 2H, 6-OCH_2), 4.17 (t, *J* = 6.4 Hz, 2H, 4'-OCH_2), 4.23 (t, *J* = 6.9 Hz, 2H, 2'-OCH_2), 4.39 (t, *J* = 6.7 Hz, 2H, 5-OCH_2), 4.43 (t, *J* = 6.3 Hz, 2H, 7-OCH_2), 7.09 (d, *J* = 9.3 Hz, 1H, 5'-H), 7.93 (s, 1H, 8-H), 8.49 (d, *J* = 9.0 Hz, 1H, 3-H), 8.57 (d, *J* = 9.3 Hz, 1H, 6'-H), 8.88 (d, *J* = 9.0 Hz, 1H, 4-H) ppm. ^{13}C NMR (176 MHz, $CDCl_3$) δ = 14.13 (CH_3), 22.71, 25.94, 25.99, 26.01, 26.13, 28.86, 29.05, 29.37, 29.39, 29.40, 29.42, 29.51, 29.57, 29.60, 29.62, 29.64, 29.65, 29.67, 29.69, 29.72, 29.74, 29.76, 30.18, 30.22, 30.28, 30.43, 31.93, 31.95 (CH_2), 69.76 (4'-OCH_2), 71.90 (7-OCH_2), 74.06 (3'-OCH_2), 74.67 (6-OCH_2), 75.22 (5-OCH_2), 75.27 (2'-OCH_2), 97.94 (C-8), 110.59 (C-5'), 115.11 (C-3), 115.25 (C-1'), 115.81 (C-4a), 129.70 (C-6'), 140.94 (C-6), 141.58 (C-3'), 146.78 (C-4), 147.85 (C-5), 155.26 (C-8a), 155.36 (C-2'), 161.64 (C-4'), 165.38 (C-7), 169.83 (C-2) ppm. FT-IR (ATR): $\tilde{\nu}$ = 2920 (vs), 2852 (s), 1737 (w), 1625 (m), 1589 (w), 1549 (m), 1517 (m), 1492 (s), 1467 (s), 1369 (m), 1336 (s), 1291 (vs), 1225 (s), 1203 (s), 1158 (m), 1133 (s), 1074 (m), 1030 (s), 812 (w), 721 (w), 638 (s), 572 (w), 517 (w) cm^{-1}. MS (ESI): *m/z* = 1366.18 $[M+OCH_3+Na]^+$, 1312.18 $[M]^+$, 1143.99 $[M+H -C_{12}H_{25}]^+$. HRMS (ESI) for $[C_{87}H_{155}O_7]^+$: calc.: 1312.1767, found.: 1312.1763. CHNS analysis: calc.: C 72.28, H 10.68, S 2.19; found.: C 72.29, H 10.77, S 1.45.

6,7,8-Tris(dodecyloxy)-2-[4-(dodecyloxy)phenyl]chromenium triflate (3'-Fla-1)

Synthesis according to GP 5: phenol **7e** (100 mg, 155 µmol), ethynylketone **9a** (49 mg, 155 µmol), EtOAc (5 mL), yield: 102 mg, 93µmol, 60 %, orange solid.

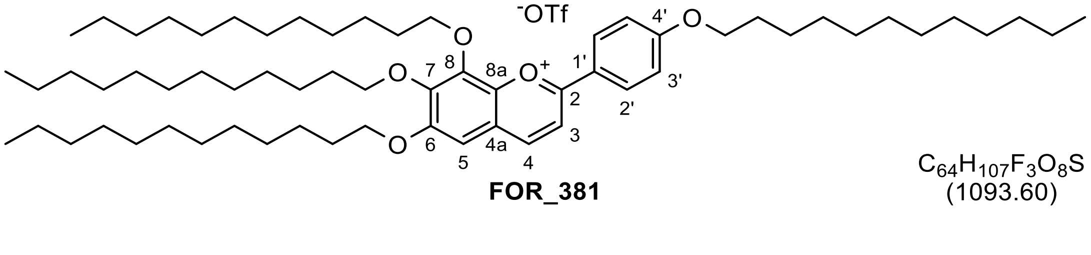

FOR_381

$C_{64}H_{107}F_3O_8S$
(1093.60)

^{1}H NMR (700 MHz, $CDCl_3$): δ = 0.84–0.93 (m, 12H, CH_3), 1.19–1.62 (m, 72H, CH_2), 1.80–1.94 (m, 6H, OCH_2OCH_2), 4.14 (t, *J* = 6.5 Hz, 2H, 4'-OCH_2), 4.20 (t, *J* = 6.3 Hz, 2H, 6-OCH_2), 4.29 (t, *J* = 6.4 Hz, 2H, 8-OCH_2), 4.39 (t, *J* = 6.5 Hz, 2H, 7-OCH_2), 7.13–7.17 (m, 2H, 3'-H), 7.44 (d, *J* = 1.3 Hz, 1H, 5-H), 8.37–8.40 (m, 2H, 2'-H), 8.62 (dd, *J* = 9.0 Hz, 1.4 Hz, 1H, 3-H), 9.42 (dd, *J* = 9.0 Hz, 1.3 Hz, 1H, 4-H) ppm. ^{13}C NMR (176 MHz, $CDCl_3$) δ = 14.12 (CH_3), 22.70, 25.85, 25.90, 26.11, 26.16, 28.92, 29.32, 29.36, 29.38, 29.40, 29.49, 29.54, 29.59, 29.60, 29.62, 29.64, 29.67, 29.69, 29.71, 29.73, 30.40, 30.42, 31.92, 31.94 (CH_2), 69.29 (4'-OCH_2), 70.22 (6-OCH_2), 75.39 (7-OCH_2), 75.57 (8-OCH_2), 104.63 (C-5), 115.66 (C-3), 116.66 (C-3'), 120.94 (C-1'), 121.32 (C-4a), 132.26 (C-2'), 140.43 (C-8), 146.82 (C-8a), 152.64 (C-7), 154.45 (C-4), 154.63 (C-6), 166.85 (C-4'), 170.64 (C-2) ppm. FT-IR (ATR): $\tilde{\nu}$ = 2922 (vs), 2853 (vs), 1603 (m), 1553 (m), 1535 (m), 1501 (m), 1460 (m), 1426 (m), 1377 (m), 1349 (m), 1274 (s), 1257 (s), 1184 (s), 1140 (m), 1074 (w), 1031 (m), 843 (w), 721 (w), 638 (m), 517 (w) cm^{-1}. MS (ESI): *m/z* = 997.82 $[M+OCH_3+Na]^+$, 943.81 $[M]^+$. HRMS (ESI) for $[C_{63}H_{107}O_5]^+$: calc.: 943.8113, found.: 943.8101. CHNS analysis: calc.: C 70.29, H 9.86, S 2.93; found.: C 69.77, H 9.73, S 2.08.

6,7,8-Tris(dodecyloxy)-2-[3,4-bis(dodecyloxy)phenyl]chromenium triflate (3'-Fla-2)

Synthesis according to GP 5: phenol **7e** (100 mg, 155 µmol), ethynylketone **9d** (49 mg, 155 µmol), EtOAc (5 mL), yield: 102 mg, 93µmol, 60 %, red solid.

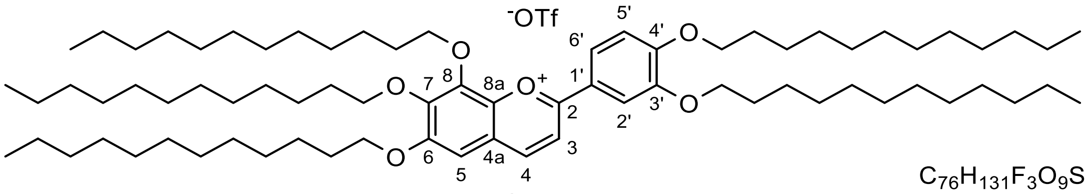

FOR_382

$C_{76}H_{131}F_3O_9S$
(1277.93)

^{1}H NMR (700 MHz, $CDCl_3$): δ = 0.82–0.92 (m, 15H, CH_3), 1.17–1.59 (m, 90H, CH_2), 1.80–1.95 (m, 10H, OCH_2CH_2), 4.13–4.22 (m, 6H, 5-OCH_2, 3'-OCH_2, 4'-OCH_2), 4.29 (t, *J* = 6.7 Hz, 2H, 8-OCH_2), 4.38 (t, *J* = 6.6 Hz, 4H, 7-OCH_2), 7.10 (d, *J* = 8.8 Hz, 1H, 5'-H), 7.41 (s, 1H, 5-H), 7.79 (d, *J* = 2.3 Hz, 1H, 2'-H), 8.13 (dd, *J* = 8.8 Hz, 2.3 Hz, 1H, 6'-H), 8.66 (d, *J* = 9.0 Hz, 1H, 3-H), 9.36 (d, *J* = 9.0 Hz, 1H, 4-H) ppm. ^{13}C NMR (176 MHz, $CDCl_3$) δ = 14.13 (CH_3), 22.71, 25.87, 25.91, 26.05, 26.07, 26.13, 28.86, 28.94, 29.14, 29.35, 29.37, 29.39, 29.40, 29.41, 29.43, 29.45, 29.47, 29.54, 29.61, 29.62, 29.63, 29.65, 29.68, 29.71, 29.72, 29.74, 29.74, 29.76, 29.80, 30.42, 30.57, 31.94, 31.95 (CH_2), 69.56, 69.76 (3'-OCH_2, 4'-OCH_2) 70.20 (6-OCH_2),

75.40 (7-OCH_2), 75.52 (8-OCH_2), 104.58 (C-5), 111.63 (C-2'), 113.27 (C-5'), 116.08 (C-3), 120.98 (C-1'), 121.23 (C-4a), 126.35 (C-6'), 140.51 (C-8), 146.59 (C-8a), 150.35 (C-3'), 152.30 (C-7), 153.87 (C-6), 154.59 (C-4), 157.80 (C-4'), 170.67 (C-2) ppm. FT-IR (ATR): $\tilde{\nu}$ = 2955 (m), 2918 (vs), 2850 (s), 1716 (w), 1612 (w), 1596 (m), 1548 (m), 1499 (s), 1465 (s), 1452 (s), 1429 (m), 1377 (m), 1354 (s), 1286 (vs), 1250 (vs), 1194 (s), 1137 (vs), 1069 (m), 1028 (vs), 990 (m), 953 (m), 852 (w), 812 (w), 721 (m), 573 (w), 516 (w) cm^{-1}. MS (ESI): *m/z* = 1127.99 [M^+]. HRMS (ESI) for $[C_{75}H_{131}O_6]^+$: calc.: 1172.9940, found.: 1127.9913. CHNS analysis: calc.: C 71.43, H 10.33, S 2.51; found.: C 71.74, H 10.44, S 1.34.

6,7,8-Tris(dodecyloxy)-2-[3,4,5-tris(dodecyloxy)phenyl]chromenium triflate (3'-Fla-3)

Synthesis according to GP 5: phenol **7e** (100 mg, 155 µmol), ethynylketone **9f** (106 mg, 155 µmol), EtOAc (5 mL), yield: 59 mg, 40.3 µmol, 26 %, dark red waxy solid.

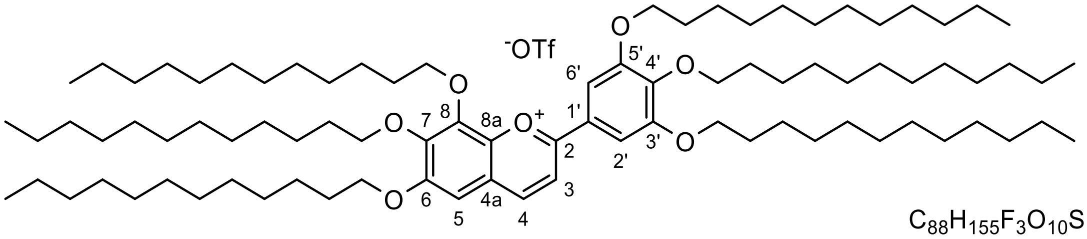

FOR_383

$C_{88}H_{155}F_3O_{10}S$
(1462.2)

^{1}H NMR (700 MHz, $CDCl_3$): δ = 0.83–0.91 (m, 18H, CH_3), 1.16–1.55 (m, 108H), 1.74–1.95 (m, 12H, OCH_2CH_2), 4.14 (t, *J* = 6.4 Hz, 4H, 3'-OCH_2), 4.20 (t, *J* = 6.4 Hz, 2H, 6-OCH_2), 4.23 (t, *J* = 6.5 Hz, 2H, 4-OCH_2), 4.29 (t, *J* = 6.8 Hz, 2H, 8-OCH_2), 4.41 (t, *J* = 6.6 Hz, 2H, 7-OCH_2), 7.42 (s, 1H, 5-H), 7.60 (s, 2H, 3'-H), 8.76 (d, *J* = 8.9 Hz, 1H, 3-H), 9.46 (d, *J* = 8.9 Hz, 1H, 4-H) ppm. ^{13}C NMR (176 MHz, $CDCl_3$) δ = 14.12 (CH_3), 22.70, 25.86, 25.95, 25.97, 26.12, 26.13, 28.90, 29.28, 29.35, 29.38, 29.39, 29.41, 29.46, 29.51, 29.55, 29.60, 29.62, 29.67, 29.69, 29.71, 29.73, 29.74, 29.76, 30.42, 30.45, 30.62, 31.94 (CH_2), 69.72, 70.27, 74.33, 75.52, 75.54 (OCH_2), 104.45 (C-5), 107.70 (C-3'), 116.56 (C-3), 121.59 (C-4a), 122.80 (C-1), 140.38 (C-8), 147.03, 147.04 (C-4, C-8a), 152.78 (C-7), 153.99 (C-3'), 154.57 (C-4), 154.74 (C-6), 170.47 (C-2) ppm. FT-IR (ATR): $\tilde{\nu}$ = 2920 (vs), 2852 (vs), 1615 (w), 1579 (w), 1552 (m), 1530 (w), 1492 (m), 1465 (s), 1423 (w), 1377 (m), 1347 (vs), 1252 (vs), 1225 (m), 1194 (m), 1121 (s), 1031 (s), 872 (w), 805 (w), 722 (w), 638 (s), 573 (w), 517 (w) cm^{-1}. MS (ESI): *m/z* = 1312.17 $[M]^+$. HRMS (ESI) for $[C_{87}H_{155}O_7]^+$: calc.: 1312.1767, found.: 1312.1749. CHNS analysis: calc.: C 71.34, H 10.64, S 2.12; found.: C 71.32, H 10.85, S 1.11.

6,7,8-Tris(dodecyloxy)-2-[2,3,4-tris(dodecyloxy)phenyl]chromenium triflate (3'-Fla-3')

Synthesis according to GP 5: phenol **7f** (100 mg, 154 µmol), ethynylketone **9f** (106 mg, 154 µmol), EtOAc (5 mL), yield: 96 mg, 65.7 µmol, 42 %, orange-red solid.

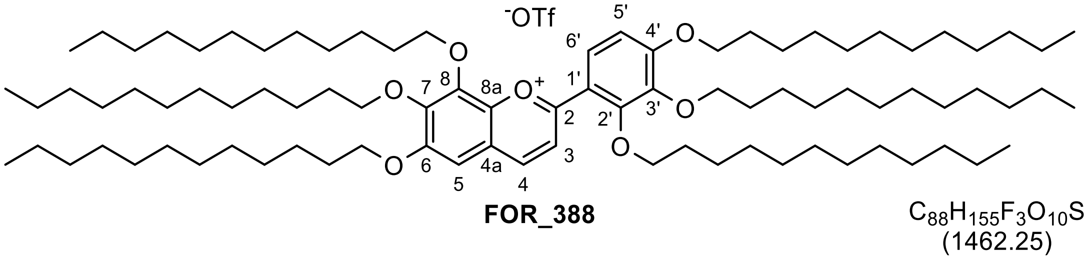

^{1}H NMR (700 MHz, $CDCl_3$): δ = 0.85–0.91 (m, 18H, CH_3), 1.20–1.59 (m, 108H, CH_2), 1.78–1.93 (m, 12H, OCH_2CH_2), 4.01 (t, *J* = 6.6 Hz, 2H,), 4.14 (t, *J* = 6.5 Hz, 2H), 4.27 (td, *J* = 6.6 Hz, 2.1 Hz, 7H), 4.40 (t, *J* = 6.5 Hz, 2H), 6.89 (d, *J* = 9.2 Hz, 1H), 7.76 (s, 1H), 7.98 (d, *J* = 9.1 Hz, 1H), 8.68 (d, *J* = 9.0 Hz, 1H), 9.57 (d, *J* = 9.1 Hz, 1H) ppm. ^{13}C NMR (176 MHz, $CDCl_3$) δ = 14.12 (CH_3), 22.70, 25.82, 25.85, 26.04, 26.12, 26.15, 26.17, 28.98, 29.08, 29.33, 29.37, 29.39, 29.40, 29.42, 29.46, 29.54, 29.58, 29.61, 29.64, 29.67, 29.70, 29.72, 29.76, 29.79, 30.21, 30.26, 30.41, 30.42, 31.93, 31.95 (CH_2), 69.61 (4'-OCH_2), 70.47 (6-OCH_2), 74.24 (3'-OCH_2), 75.38 (7-OCH_2), 75.48, 75.61 (8-OCH_2, 2'-OCH_2), 105.34 (C-5), 109.37 (C-5'), 116.14 (C-2'), 118.53 (C-2), 121.79 (C-4a), 126.92 (C-6'), 139.99 (C-8), 142.19 (C-3'), 147.55 (C-8a), 153.03 (C-7), 154.73 (C-6), 154.92 (C-4), 155.77 (C-2'), 160.99 (C-4'), 168.93 (C-2) ppm. FT-IR (ATR): $\tilde{\nu}$ = 2956 (m), 2918 (vs), 2850 (s), 1591 (w), 1556 (w), 1518 (w), 1489 (m), 1466 (m), 1381 (m), 1340 (m), 1302 (s), 1278 (m), 1132 (m), 1081 (w), 1030 (m), 721 (w), 637 (w), 518 (w) cm^{-1}. MS (ESI): *m*/*z* = 1366.17 $[M+OCH_3+Na]^+$, 1312.17 $[M]^+$. HRMS (ESI) for $[C_{87}H_{155}O_7]^+$: calc.: 1312.18, found.: 1312.1671. CHNS analysis: calc.: C 72.28, H 10.68, S 2.19; found.: C 72.04, H 10.80, S 1.47.

Appendix

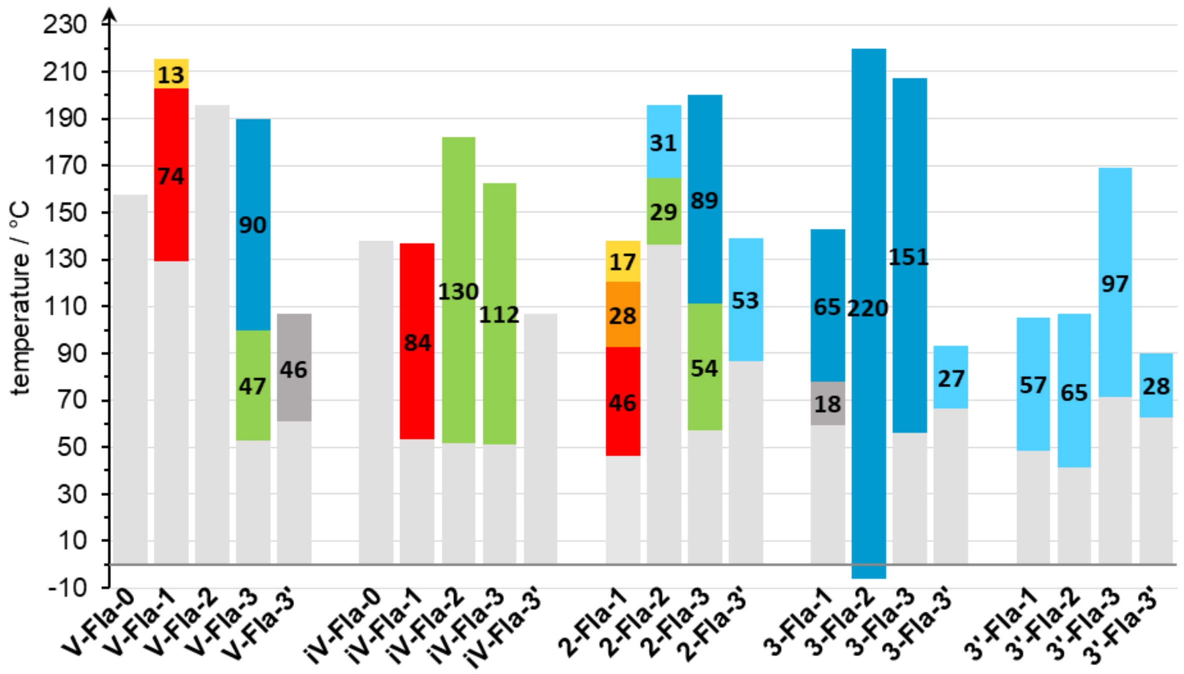

Figure S1 Overview of the observed mesophase of the flavylium salts **A-Fla-B**. Mesophase widths are given as numbers in the corresponding bars (solid phase: light and dark grey, yellow: SmA, orange: SmA', red: Lam_{Col}, green: Col_r, blue: Col_h, black: I_{re}).

Table S1 Phase transitions T / °C and their enthalpies ΔH / kJ mol^{-1} of the flavylium salts **A-Fla-B** obtained by DSC during the first and second heating/cooling cycle with heating and cooling rates of 10 K min^{-1}.

		Phase [a]	T_1 (ΔH)	Phase [a]	T_2 (ΔH)	Phase [a]	T_{Cl} (ΔH)	Phase [a]
**V-Fla-0**	1st H	Cr_1	84 (-30.0)	Cr_2			157 (-23.2)	I
	1st C		67 (17.1)				146 (20.2)	
	2nd H		68 (-16.6)				154 (-20.5)	
	2nd C		66 (18.24)				146 (18.8)	
**V-Fla-1**	1st H	Cr	129 (-55.1)	Lam_{Col}	201 (-5.2)	SmA	214 (-7.1)	I
	1st C				197 (3.0)		213 (6.5)	
	2nd H				190 (-4.1)		209 (-9.5)	
	2nd C				189 (2.2)		209 (8.0)	
**V-Fla-2**	1st H	Cr					195 (-59.5)	I
	1st C						181 (47.1)	
	2nd H						188 (-46.1)	
	2nd C						174 (34.4)	
**V-Fla-3**	1st H	Cr_1	53 (-10.3)	Col_{ro}	100 (-23.9)	Col_{ho}	190 (-6.6)	I
	1st C				85 (9.6)		183 (2.99)	
	2nd H				94 (-22.7)		174 (-5.7)	
	2nd C				90 (6.2)		–	

**V-Fla-3'**	1st H	Cr$_1$	54 (-42.9)	Cr$_2$			102 (-32.0)	I
	1st C						79 (46.5)	
	2nd H						103 (-29.5)	
	2nd C						85 (47.5)	
**iV-Fla-0**	1st H	Cr$_1$	33 (-12.0)	Cr$_2$			136 (-26.3)	I
	1st C						92 (14.6)	
	2nd H		80 (10.1) [b]				136 (-25.1)	
	2nd C						92 (14.2)	
**iV-Fla-1**	1st H	Cr	47 (40.8)	Lam$_{Col}$			131 (-7.7)	I
	1st C						135 (4.8)	
	2nd H						130 (-7.9)	
	2nd C						134 (4.30)	
**iV-Fla-2**	1st H	Cr	52 (-51.0)	Col$_{ro}$			199 (-21.5)	I
	1st C							
	2nd H							
	2nd C							
**iV-Fla-3**	1st H	Cr	50 (-6.2)	Col$_{ro}$			156 (-12.4)	I
	1st C	Gl	52 [c]				162 (4.7)	
	2nd H	Gl	55 [c]				152 (-6.1)	
	2nd C	Gl	46 [c]				159 (4.4)	
**iV-Fla-3'**	1st H	Cr					106 (-66.1)	I
	1st C						88 (10.6)	
	2nd H						104 (-63.7)	
	2nd C						88 (7.84)	
**2-Fla-1**	1st H	Lam$_{Col}$[d]	100 (-2.1)	SmA'	126 (-0.7)	SmA	145 (-3.2)	I
	1st C		102 (2.5)		125 (0.4)		149 (3.4)	
	2nd H		96 (-1.7)		126 (0.4)		147 (-3.1)	
	2nd C		103 (2.9)		125 (0.4)		148 (3.1)	
**2-Fla-2**	1st H	Cr$_1$	119 (-6.8) Cr$_2$ 131 (-17.7)	Col$_{ro}$	164 (-0.8)	Col$_{ho}$	180 (-9.7)	I
	1st C		-4 (12.1)				179 (14.1)	
	2nd H		-6 (-13.8)				177 (-11.2)	
	2nd C		-1 (12.1)				176 (14.6)	
**2-Fla-3** [g]	1st C	Gl	59	Col$_{ho}$	102 (26.9)			
	2nd H		65		101 (-26.2)			
	2nd C		58		101 (26.0)			
	3rd H		58		116 (-25.0)		200 (-5.9)	I
**2-Fla-3'**	1st H	Cr	80 (91.0)	Col$_h$			134 (-0.3)	I
	1st C		68 (82.7)				137 (0.9)	
	2nd H		77 (-55.6)				132 (-0.5)	
	2nd C		66 (78.5)				137 (0.7)	
**3-Fla-1**	1st H	Cr	54 (-90.9)	I$_{re}$	78 [e]	Col$_h$	143 [e]	I
	1st C		0 [f]	I$_{re}$	78 [e]	Col$_h$	119 [e]	
	2nd H				79 [e]		144 [e]	
	2nd C				76 [e]		118 [e]	
**3-Fla-2**	1st H	Cr	-6 (-20.0)	Col$_{ho}$			215 (-7.4)	I
	1st C							
	2nd H							
	2nd C							
**3-Fla-3** [g]	1st H	Cr	56 (-74.8)	Col$_{ho}$				
	1st C		5 (20.2)					
	2nd H		-1 (-21.8)					
	2nd C		5 (21.4)					
	3rd H		-1 (-22.5)				209 (-10.5)	I
	3rd C		1 (20.0)				208 (4.3)	

**3-Fla-3'**	1st H	Cr_1	–	Cr_2	63 (-138.6)	Col_h	90 (-5.7)	I
	1st C		6 (14.8)				88 (3.4)	
	2nd H		8 (-19.5)		52 (-104.7)		85 (-4.6)	
	2nd C		6 (14.5)				85 (2.9)	
**3'-Fla-1**	1st H	Cr_1	–	Cr_2	41 (-91.4)	Col_h	101 (-1.3)	I
	1st C		-2 (14.4)				91 (0.6)	
	2nd H		0 (-14.5		24 (-22.0)			
	2nd C		-2 (15.5)					
**3'-Fla-2**	1st H	Cr	38 (-54.1)	Col_h			147 (-1.6)	I
	1st C		7 (18.9)				147 (0.3)	
	2nd H		6 (-17.4)					
	2nd C		7 (19.3)					
**3'-Fla-3**	1st H	Cr	11 (-26.0)	Col_h			169 [d]	I
	1st C		6 (15.6)					
	2nd H		7 (-14.1)					
	2nd C		-1 (4.8)					
**3'-Fla-3'**	1st H	Cr	58 (-85.35)	Col_h	90 [d]		169 [d]	I
	1st C							
	2nd H							
	2nd C							

[a] The following phases were observed: Cr_x (crystalline), Gl (glass), SmA (smectic A), SmA' (smectic A'), Lam_{Col} (lamello-columnar), Col_{ro} (columnar rectangular ordered), Col_{ho} (columnar rectangular ordered), Col_h (columnar hexagonal), I (isotropic), I_{re} (isotropic re-entrant), [b] cold crystallisation, [c] glass transition, [d] In the 1st heating a transition from the crystalline state into the Lam_{Col} phase is observed at 46 °C (-39.9 kJ mol^{-1}), [e] Transition observed under the POM, [f] determined by sheering of the sample under the POM, [g] due to strong decomposition of the sample the first to heating/cooling cycles was performed from -30 °C to 150 °C and the 3rd from-30 to 220 °C.

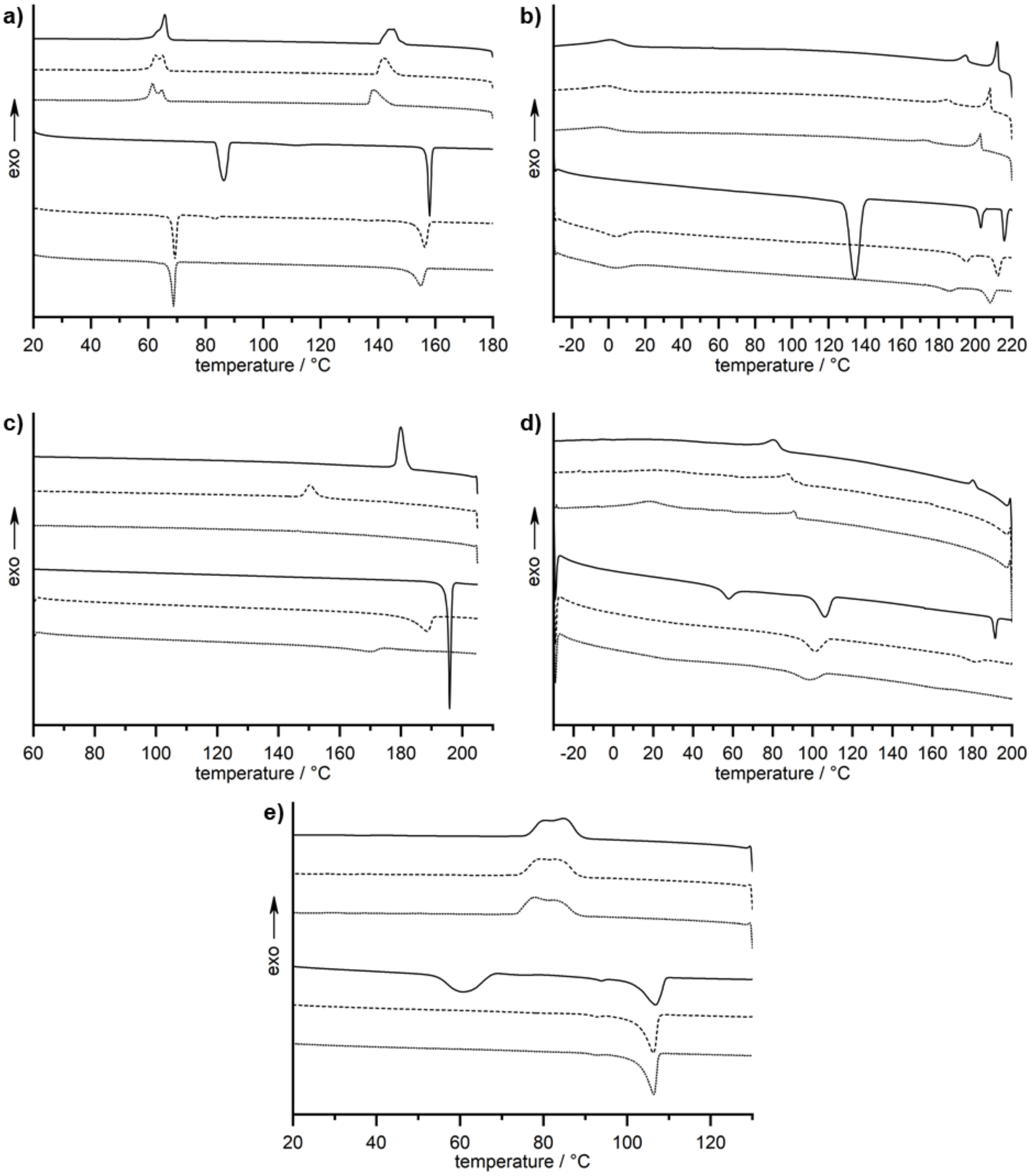

Figure S2 DSC traces of a) **V-Fla-0**, b) **V-Fla-1**, c) **V-Fla-2**, d) **V-Fla-3** and e) **V-Fla-3'**. In the 1st, 2nd, 3rd cooling and 1st, 2nd, 3rd heating (from top to bottom) with a rate of 10 K min^{-1}.

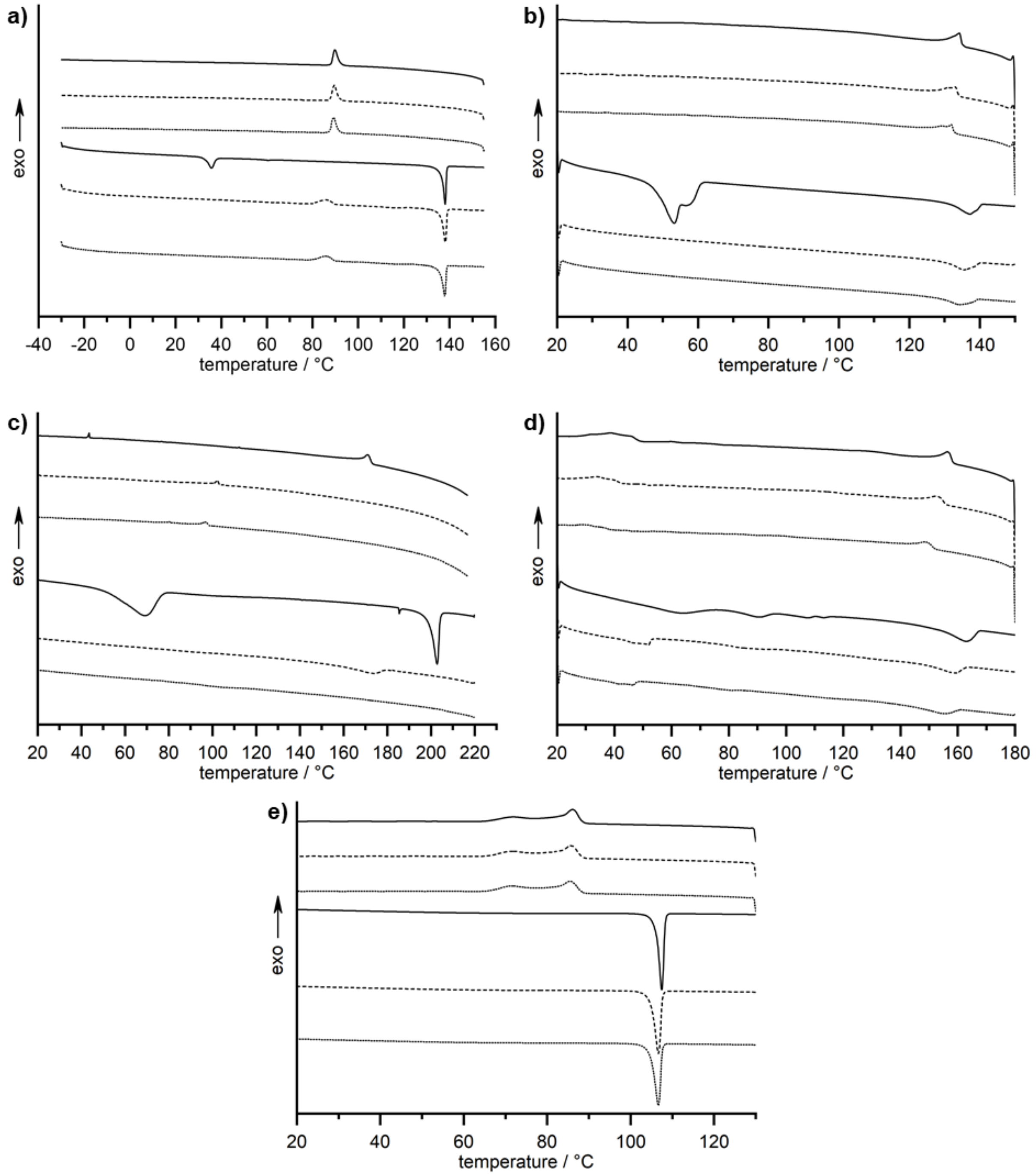

Figure S3 DSC traces of a) **iV-Fla-0**, b) **iV-Fla-1**, c) **iV-Fla-2**, d) **iV-Fla-3** and e) **iV-Fla-3'** in the 1st, 2nd, 3rd cooling and 1st, 2nd, 3rd heating (from top to bottom) with a rate of 10 K min^{-1}.

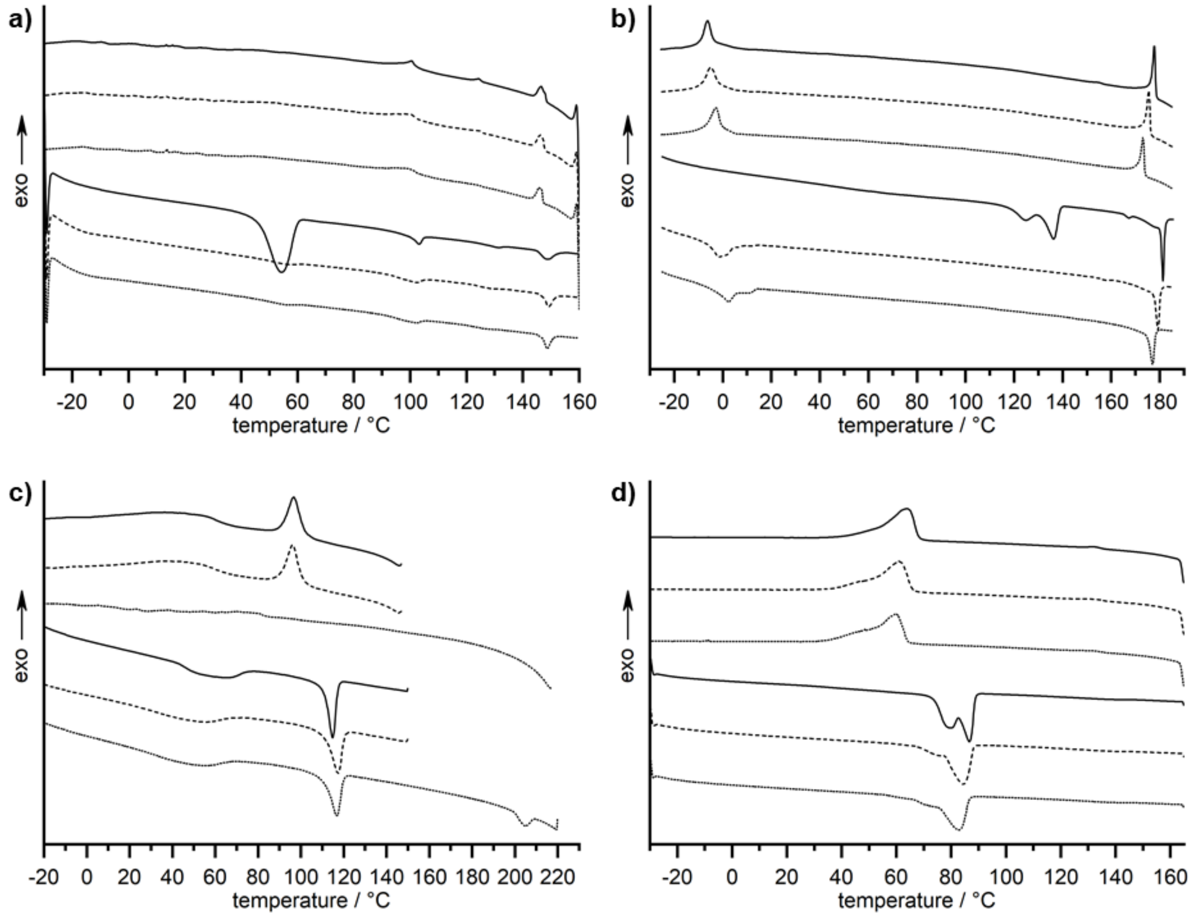

Figure S4 DSC traces of a) **2-Fla-1**, b) **2-Fla-2**, c) **2-Fla-3**, d) **2-Fla-3'** In the 1st, 2nd, 3rd cooling and 1st, 2nd, 3rd heating (from top to bottom) with a rate of 10 K min^{-1}.

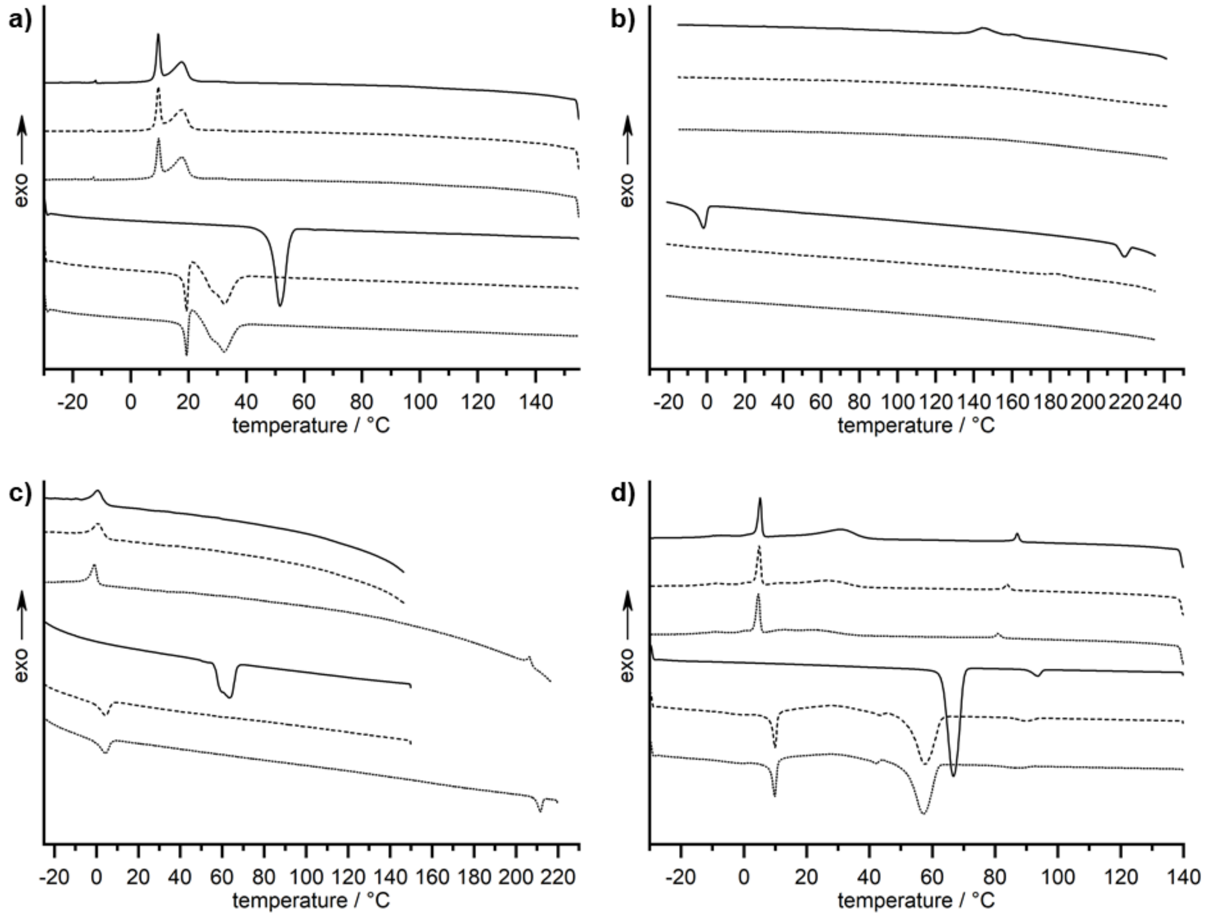

Figure S5 DSC traces of a) **3-Fla-1**, b) **3-Fla-2**, c) **3-Fla-3**, d) **3-Fla-3'** in the 1st, 2nd, 3rd cooling and 1st, 2nd, 3rd heating (from top to bottom) with a rate of 10 K min^{-1}.

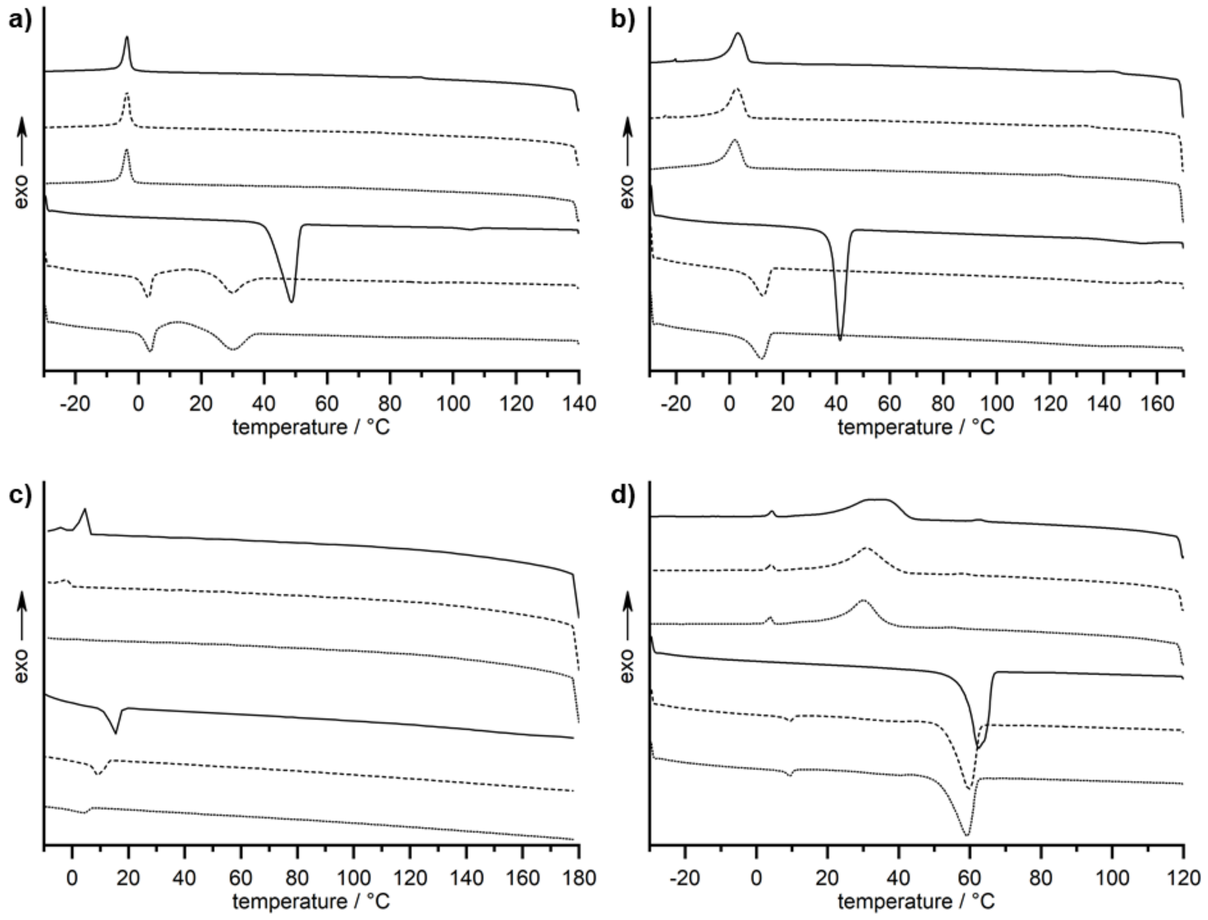

Figure S6 DSC traces of a) **3'-Fla-1**, b) **3'-Fla-2**, c) **3'-Fla-3**, d) **3'-Fla-3'** in the 1st, 2nd, 3rd cooling and 1st, 2nd, 3rd heating (from top to bottom) with a rate of 10 K min^{-1}.

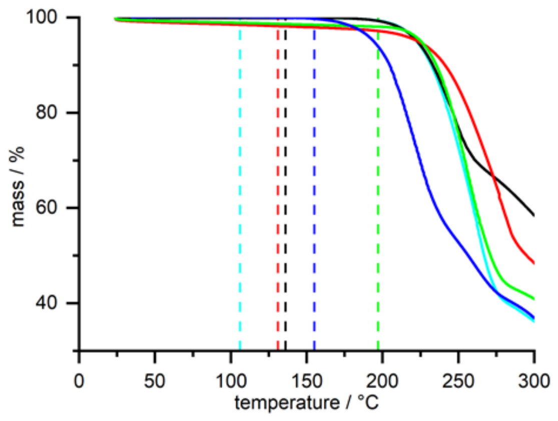

Figure S7 TGA trace of **iV-Fla-0** (black), **iV-Fla-1** (red) and **iV-Fla-2** (green), **iV-Fla-3** (blue) and **iV-Fla-3'** (light blue) obtained under a constant flow of synthetic air and a heating rate of 5 K min^{-1}. The clearing temperatures are given as dashed lines.

Table S2 Detailed table of the XRD data.

Compound	phase	Lattice parameter / Å	*d* spacing / Å [a]	Miller index
**V-Fla-1**	SmA at 210 °C		29.71	(001)
			14.91 (14.86)	(002)
			4.66	(halo)
			3.64	(anions)
	Lam_{Col} at 170 °C	Z = 1	32.03	(001)
			15.96 (16.02)	(002)
			10.04	(010)
			4.67	(halo)
			3.52	(π-π)
**iV-Fla-1**	Lam_{Col} at 100 °C	Z = 1	31.25	(001)
			15.61 (15.63)	(002)
			11.42	(010)
			10.21 (10.41)	(003)
			4.84	(halo)
			3.63	(π-π)
**2-Fla-1**	SmA at 130 °C		31.08	(001)
			4.75	(halo)
			3.77	(π-π)
	SmA' at 120 °C		31.58	(001)
			15.68 (15.78)	(002)
			4.66	(halo)
			3.48	(π-π)
	Lam_{Col} at 85 °C	Z = 1	34.00	(001)
			17.01 (17.00)	(002)
			11.35 (11.33)	(003)
			10.61	(010)
			8.52 (8.50)	(004)
			4.60	(halo)
			3.50	(π-π)
**iV-Fla-2**	Col_{ro} at 110 °C	*a* = 35.9	29.18	(110)
	p2gg	*b* = 50.0	25.01	(020)
		Z = 4	20.46 (20.53)	(120)
			16.91 (16.91)	(210)
			14.59 (14.59)	(220)
			12.22 (12.22)	(230)
			11.65 (11.65)	(310)
			4.71	(halo)
			3.52	(anions)
			3.44	(π-π)
**iV-Fla-3**	Col_{ro} at 120 °C	*a* = 36.3	29.55	(110)
	p2gg	*b* = 51.0	25.51	(020)
		Z = 4	20.77 (20.86)	(120)
			17.32 (17.08)	(210)
			7.50 (8.12, 6.78)	(π-π2)
			4.63	(halo)
			4.06	(anions)
			3.39	(π-π)
**2-Fla-2**	Col_{ho} at 170 °C	*a* = 35.5	30.81	(100)
	p6mm	*Z* = 2	15.40 (15.40)	(200)
			11.53 (11.65)	(210)
			4.68	(halo)
			3.49	(π-π)
	Col_{ro} at 140 °C	*a* = 61.0	30.02	(11)
	p2gg	*b* = 34.5	22.84	(21)
			17.27 (17.25)	(02)

			14.94 (15.01)	(22)
			13.42 (13.15)	(32)
			11.25 (11.30)	(13)
			4.50	(halo)
			3.46	(anions)
			3.42	(π-π)
**2-Fla-3**	Col_{ho} at 140 °C	*a* = 27.4	24.04	(100)
	p6mm	*Z* = 1	13.86 (13.88)	(110)
			12.01 (12.02)	(200)
			6.75 (6.88)	(π-π')
			4.52	(halo)
			3.44	(π-π)
	Col_{ro} at 80 °C	*a* = 61.3	30.67	(20), (11)[b]
	p2gg or *p2mm*	*b* = 35.3	17.69	(02), (31)
		Z = 4	15.54 (15.34)	(40), (22)
			11.67 (11.59)	(51/42), (13)
		or	10.32 (10.22)	(60/33), (33)
			8.77 (8.75)	(14), (14)
		a = 30.1	8.48 (8.50)	(24/27), (71/53/24)
		b = 17.7	4.52	(halo)
		Z = 2	3.96	(anions)
			3.55	(π-π)
**3-Fla-2**	Col_{ho} at 100 °C	*a* = 28.6	23.55	(100)
	p6mm	*Z* = 1	13.65 (13.60)	(110)
			11.80 (11.77)	(200)
			8.96 (8.90)	(210)
			6.83 (6.88)	(π-π1)
			4.18	(halo)
			3.44	(π-π2)
	Col_{ho} at 021 °C		6.64 (6.74)	(π-π1)
			4.02	(halo)
			3.37	(π-π2)
**3-Fla-3**	Col_{ho} at 100 °C	*a* = 28.4	24.86	(100)
		Z = 1	14.26 (14.35)	(110)
			12.36 (12.43)	(200)
			6.88 (6.82)	(π-π')
			4.26	(halo)
			3.41	(π-π)
**V-Fla-3**	Col_{ho} at 120 °C	*a* = 25.8	22.33	(100)
	p6mm	*Z* = 1	12.95 (12.89)	(110)
			11.23 (11.16)	(200)
			8.47 (8.44)	(210)
			6.71 (6.78)	(π-π')
			4.51	(halo)
			3.39	(π-π)
	Col_{ro} at 75 °C	*a* = 41.9	57.66	(01)
	p2mm	*b* = 57.7	41.92	(10)
		Z = 4	28.87 (28.83)	(12)
			24.29 (23.75)	(20)
			18.95 (19.22)	(03)
			17.77 (17.47)	(13)
			16.52 (16.95)	(22)
			14.41 (14.42)	(04)
			13.85 (13.97)	(30)
			11.76 (11.88)	(24)
			10.87 (11.12)	(15)
			7.12 (7.28)	(π-π')
			4.43	(halo)
			3.96	(anions)

			3.64	(π-π)
**3-Fla-1**	Col_h at 80 °C<br>*p6mm*	*a* = 25.5<br>*Z* = 1	22.07<br>12.83 (12.74)<br>4.37	(100)<br>(110)<br>(halo)
**2-Fla-3'**	Col_h at 110 °C<br>*p6mm*	*a* = 27.2<br>*Z* = 1	23.51<br>13.61 (13.57)<br>11.81 (11.75)<br>4.62	(100)<br>(110)<br>(200)<br>(halo)
**3-Fla-3'**	Col_h at 80 °C<br>*p6mm*	*a* = 27.7<br>*Z* = 1	24.00<br>13.88 (13.86)<br>4.50	(100)<br>(110)<br>(halo)
**3'-Fla-1**	Col_h at 80 °C<br>*p6mm*	*a* = 29.7<br>*Z* = 2	25.72<br>4.38	(100)<br>(halo)
**3'-Fla-2**	Col_h at 70 °C<br>*p6mm*	*a* = 31.3<br>*Z* = 2	27.08<br>15.72 (15.64)<br>4.33	(100)<br>(110)<br>(halo)
**3'-Fla-3**	Col_h at 30 °C<br>*p6mm*	*a* = 30.12<br>*Z* = 1	26.08<br>15.11 (15.06)<br>4.38	(100)<br>(110)<br>(halo)
**3'-Fla-3'**	Col_h at 30 °C<br>*p6mm*	*a* = 29.04 [c]<br>*Z* = 1	25.15<br>4.46	(100)<br>(halo)

[a] Calculated values are given in parenthesis, [b] indexation was not given due to several possibilities., [c] broad reflex.

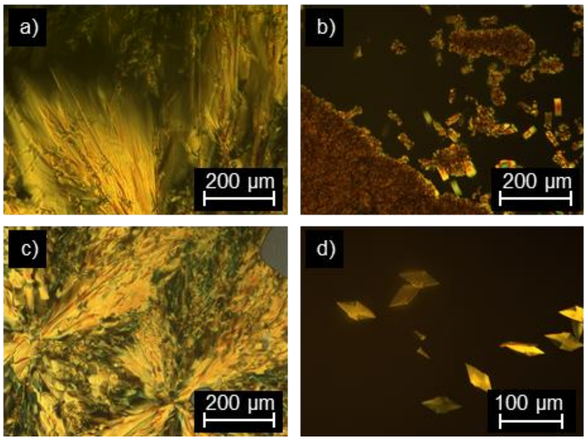

Figure S8 a) **V-Fla-0** at 140 °C (100x magn.) b) **iV-Fla-0** at 90 °C (100x magn.) c) **2-Fla-0** at 125 °C (100x magn.) d) **3'-Fla-0** at 80 °C (200x magn.). All pictures were taken by cooling from the isotropic phase with a cooling rate of 5 K min^{-1}.

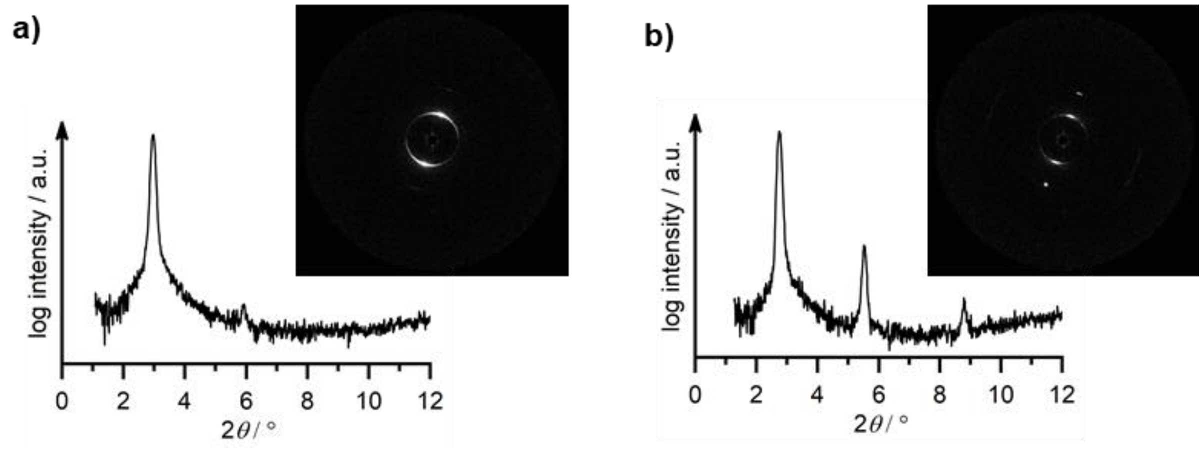

Figure S9 SAXS diffractogram and diffraction pattern of **V-Fla-1** at a) 210 °C and b) 170 °C upon cooling from the isotropic phase.

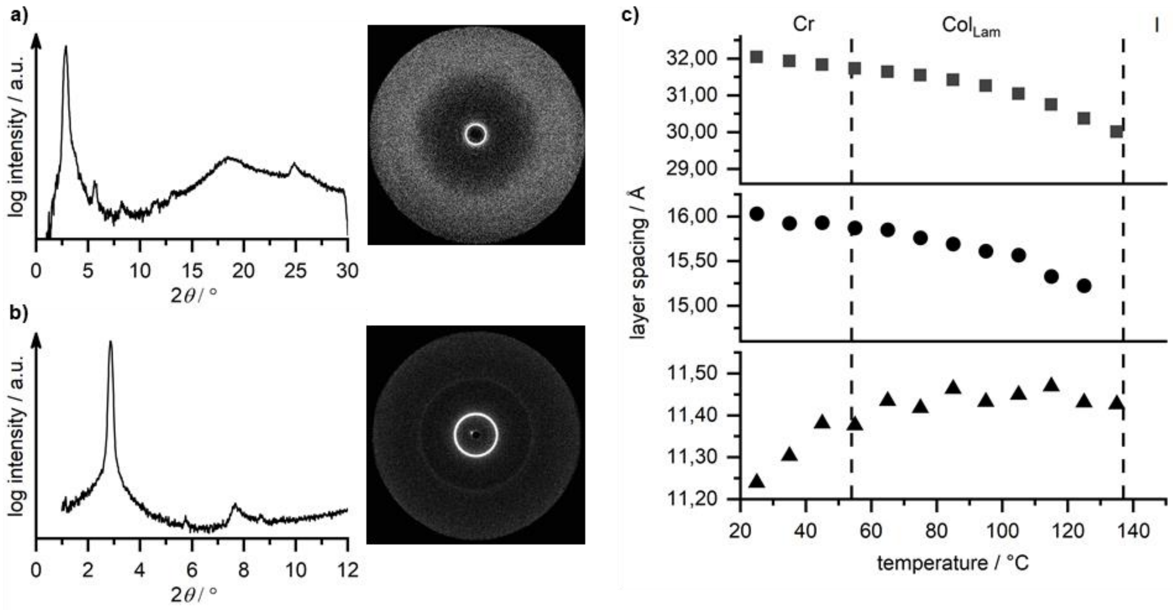

Figure S10 a) WAXS-diffractogram and b) SAXS-diffractogram at 100 °C with diffraction pattern as inset of **iV-Fla-1**. c) Temperature dependent layer spacing of the (001) reflex (square), (002) reflex (circle) and (010) reflex (triangle) of **iV-Fla-1** upon cooling from the isotropic liquid.

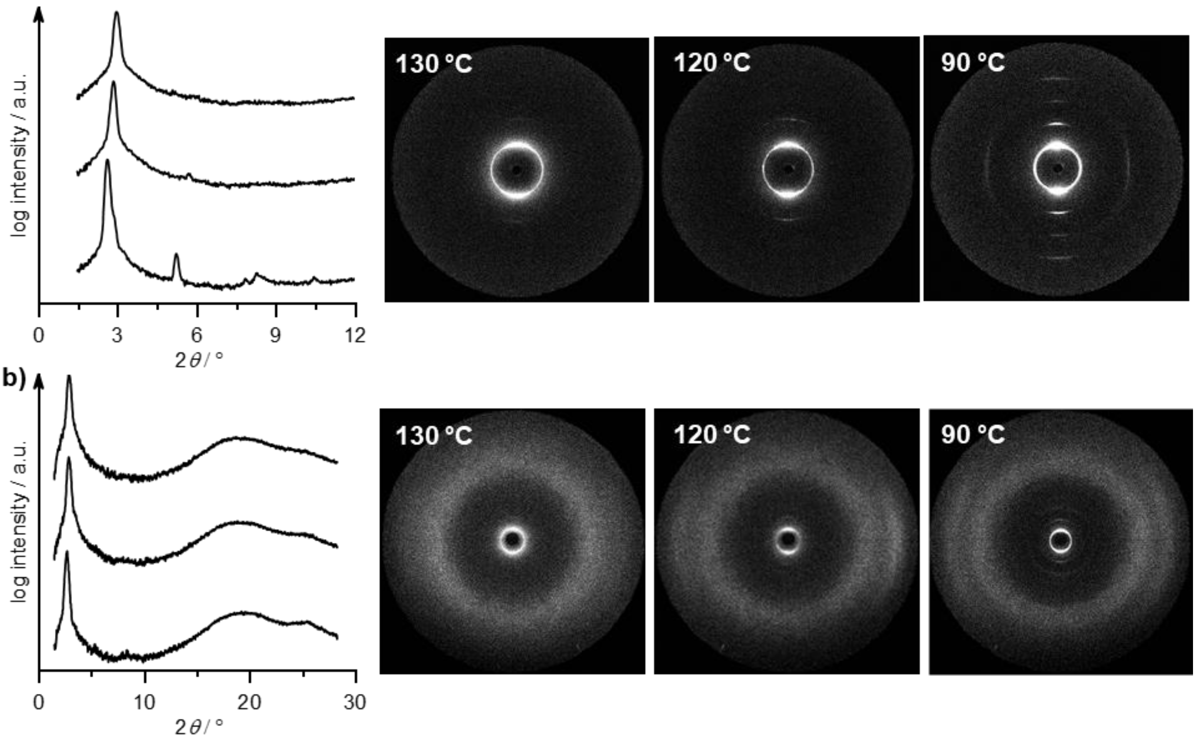

Figure S11 a) SAXS and b) WAXS diffractograms and patterns of **2-Fla-1** at 130 °C, 120 °C and 90 °C (from top to bottom).

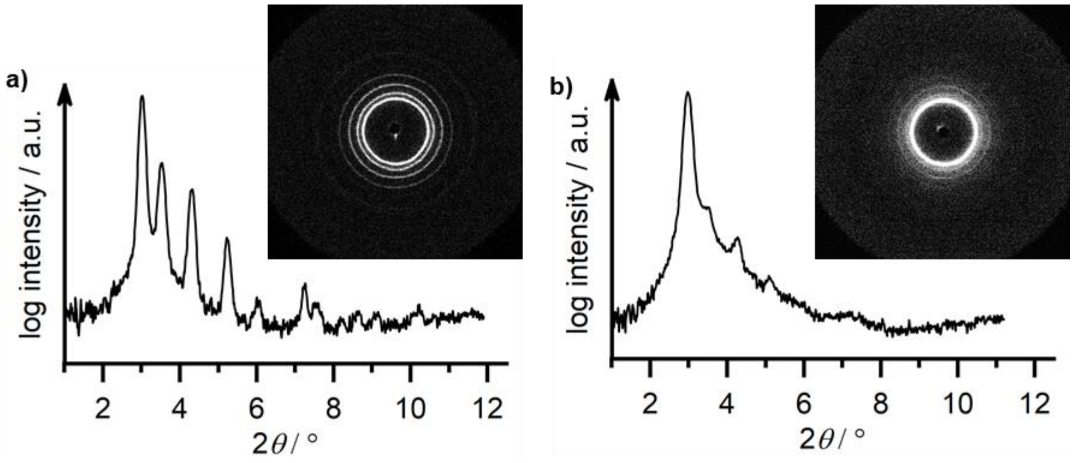

Figure S12 X-ray diffractograms of a powder sample of a) **iV-Fla-2** at 110 °C and b) **iV-Fla-3** at 120 °C with the corresponding diffraction pattern (inset) obtained by cooling from the isotropic liquid.

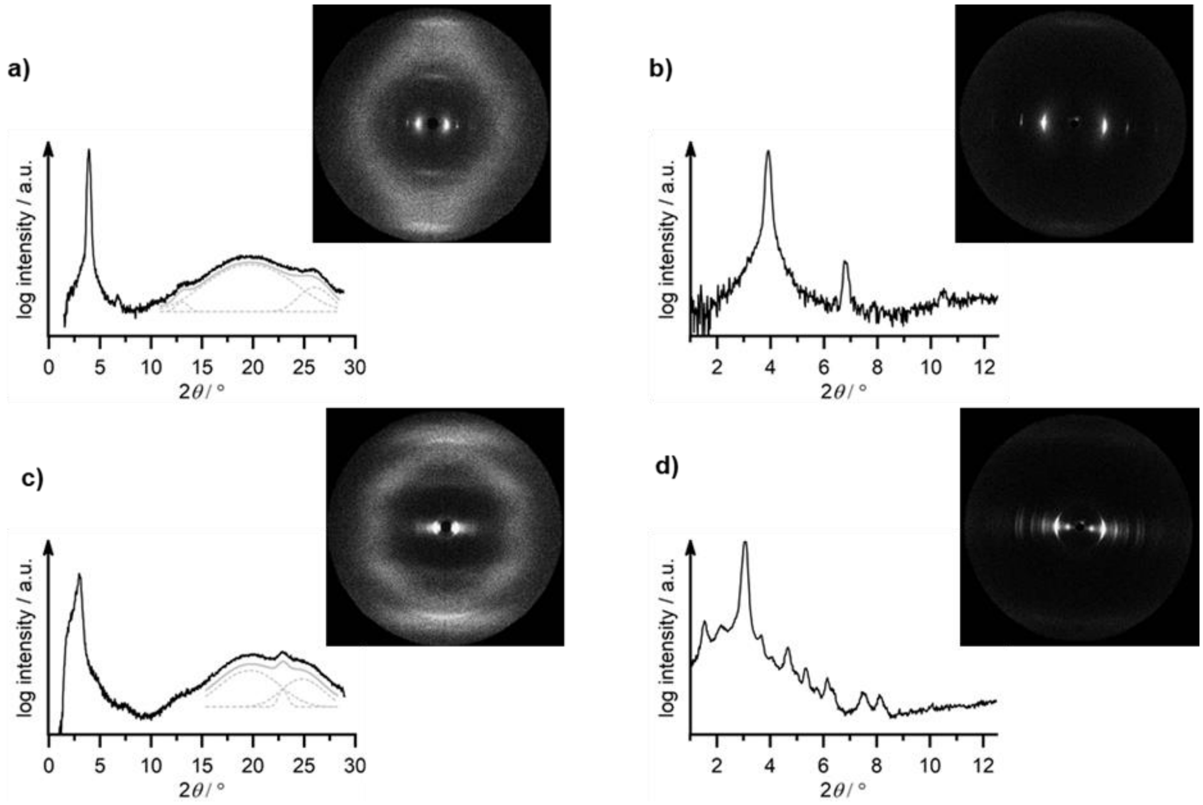

Figure S13 X-ray diffractograms of an oriented fibre of **V-Fla-3** in the Col_{ho} mesophase at 140 °C (a) WAXS b) SAXS) and in the Col_{ro} mesophase at 75 °C (c) WAXS d) SAXS) with the corresponding diffraction pattern (inset) obtained by cooling from 150 °C. The grey line corresponds to the sum of the fitted curves (dashed lines).

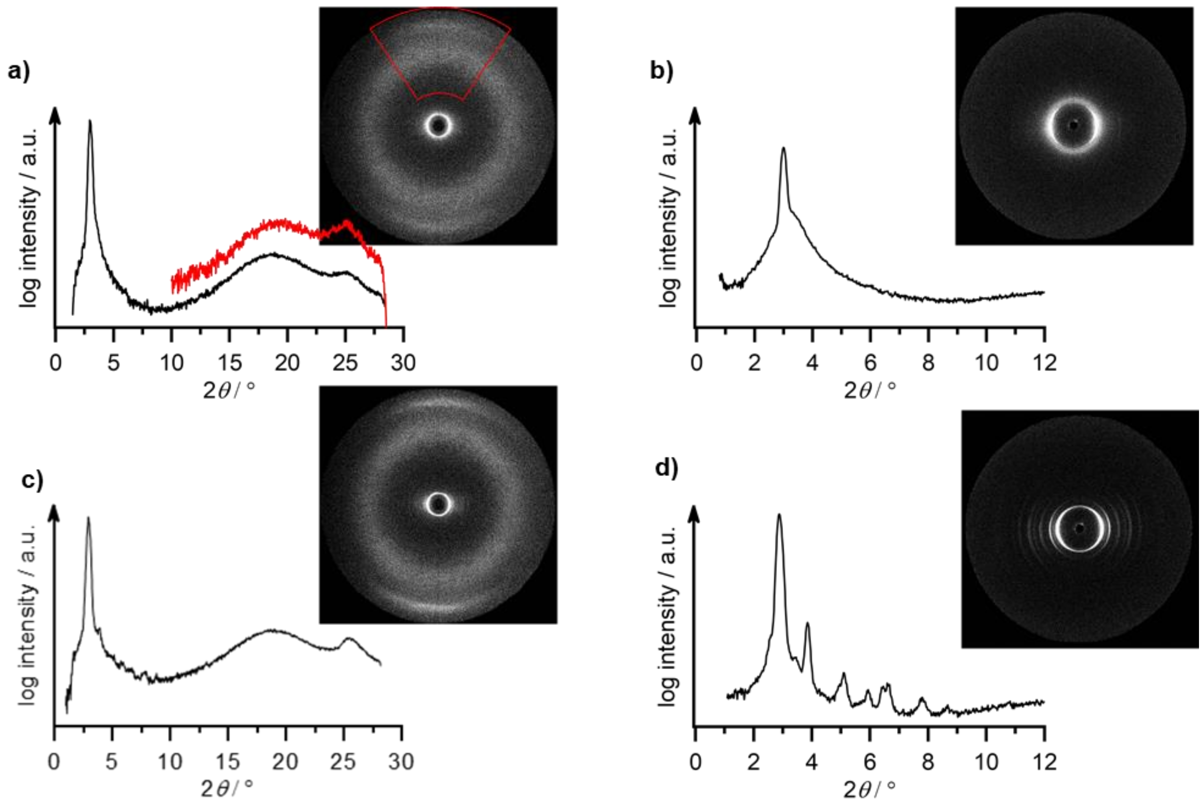

Figure S 14 X-ray diffractograms of an oriented fibre of **2-Fla-2** in the Col_{ho} mesophase at 170 °C (a) WAXS b) SAXS) and in the Col_{ro} mesophase at 75 °C (c) WAXS d) SAXS) with the corresponding diffraction pattern (inset) obtained by cooling from 140 °C.

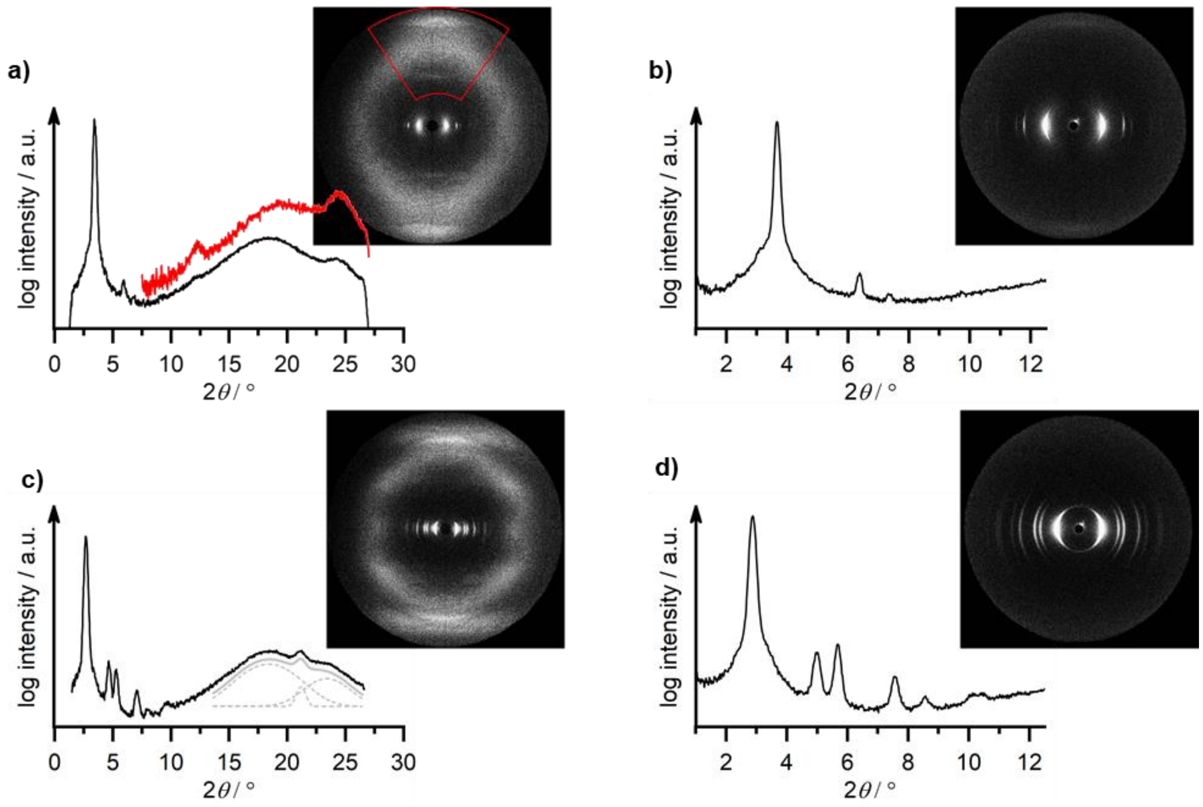

Figure S15 X-ray diffractograms of an oriented fibre of **2-Fla-3** in the Col_{ho} mesophase at 150 °C (a) WAXS b) SAXS) and in the Col_{ho} mesophase at 80 °C (c) WAXS d) SAXS) with the corresponding diffraction pattern (inset) obtained by cooling from 150 °C. The red curve corresponds to the integration in the marked area.

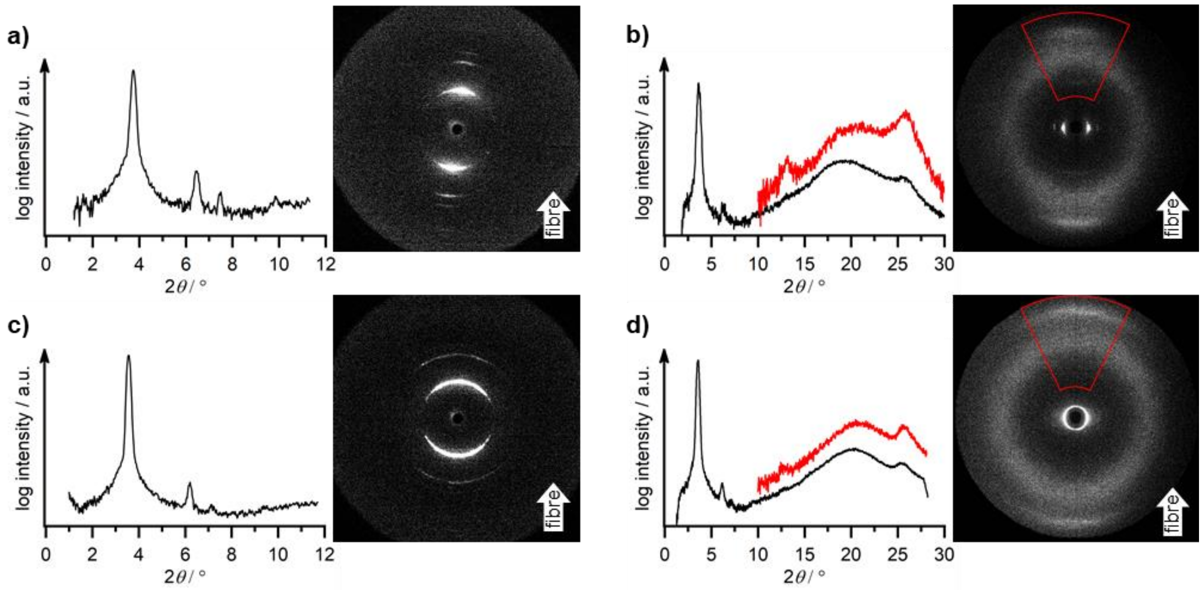

Figure S16 WAXS (a, c) and SAXS (c, d) diffractograms of an oriented fibre of **3-Fla-2** (a-b) and **3-Fla-3** (c-d) in the Col_{ho} mesophase at 100 °C and with the corresponding diffraction pattern (inset). The red curve is the integration of the marked section.

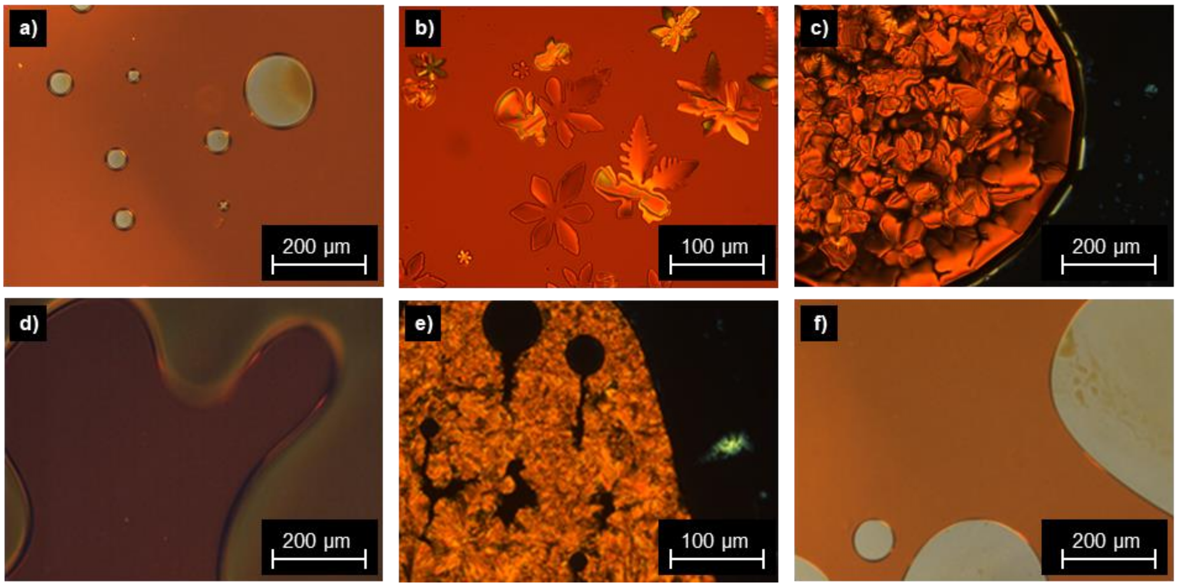

Figure S17 POM images of **3-Fla-1** upon the first cooling from the isotopic phase at a) 147 °C, b) 140 °C (with slightly uncrossed polarizers), c) 130 °C, d) 55 °C (blurred, due to fluidity of the phase). POM images o the second heating at e)

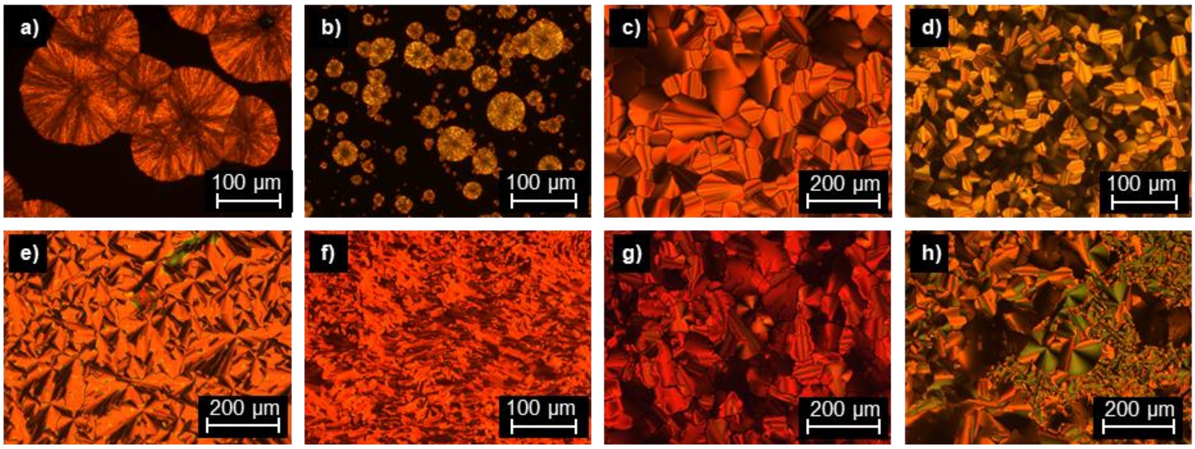

Figure S18 POM images of a) **V-Fla-** at 92 °C (magnification 100x) and b) **iV-Fla-** at -70 °C (magnification 200x) during crystallization. Textures of c) **2-Fla-3'** at 123 °C (magnification 200x), d) **3-Fla-3'** at 120 °C (magnification 100x), e) **3'-Fla-1** at 102 °C (magnification 200x), f) **3'-Fla-2** at 130 °C (magnification 100x), g) **3'-Fla-3** at 30 °C (magnification 200x) and h) **3'-Fla-3'** at 50 °C (magnification 200x). All pictures were taken by cooling from the isotropic phase with a cooling rate of 5 K min^{-1}.

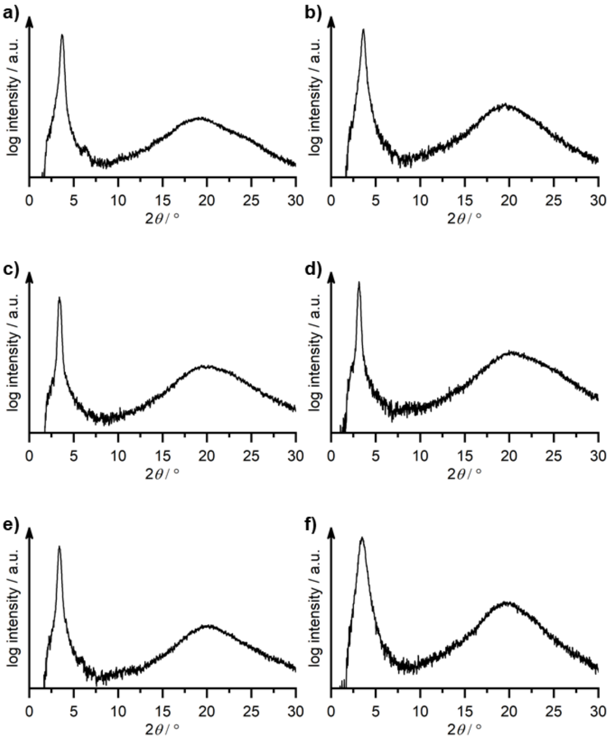

Figure S19 WAXS diffractograms of a) **2-Fla-3'** at 90 °C, b) **3-Fla-3**' at 72 °C, c) **3'-Fla-1** at 85 °C, d) **3'-Fla-2** at 50 °C, e) **3'-Fla-3** at 22 °C and f) **3'-Fla-3'** at 70 °C.

Table S3 Absorption maximum λ_{max} and the extinction coefficient ε of the flavylium salts **A-Fla-B** in $CHCl_3$ at room temperature (concentration ranging from $0.2 \cdot 10^{-5}$ M to $7 \cdot 10^{-5}$ M), as well as the emission maximum $\lambda_{Em,max}$ and the Stokes shift, absolute quantum yield Φ_F and the fluorescence lifetimes τ_1 and τ_2 obtained by TCSPC. Solutions of flavylium salts were freshly prepared from spectroscopically grade commercial solvents in order to avoid undesired reactions towards hemiketals and chalcones.

compound	λ_{max} / nm	ε / L $mol^{-1}cm^{-1}$	$\lambda_{Em,max}$ / nm	Stokes shift / cm^{-1}	Φ_F	τ_1 /ns	τ_2 /ns
**V-Fla-0**	444	44543	505	2721	0.04		
**V-Fla-1**	484	56243	521	1467	0.97	3.29	
**V-Fla-2**	501	44448	570	2416	0.05	0.36 (89 %)	0.80 (11 %)
**V-Fla-3**	496	39671	593	3298	< 0.01		
**V-Fla-3'**	490	35437	525	1361	< 0.01		
**iV-Fla-0**	444	24307	498	2442			
**iV-Fla-1**	484	46700	521	1467	0.99	3.38	
**iV-Fla-2**	501	44267	566	2292	0.06	0.41 (95 %)	1.03 (5 %)
**iV-Fla-3**	495	40353	588	3195	< 0.01		
**iV-Fla-3'**	488	63600	523	1371			
**2-Fla-0**	447	23439	503	2491			
**2-Fla-1**	485	46027	522	1472	0.92	3.16	
**2-Fla-2**	502	44286	564	2180	0.08	0.52 (91 %)	1.44 (9 %)
**2-Fla-3**	496	40656	588	3166			
**2-Fla-3'**	490	44861	525	1361			
**3-Fla-1**	485	55086	516	1239	< 0.01		
**3-Fla-2**	503	46708	567	2244			
**3-Fla-3**	498	39567	592	3188			
**3-Fla-3'**	489	43773	533	1688			
**3'-Fla-1**	487	41044	524	1450	< 0.01		
**3'-Fla-2**	507	38566	566	2056			
**3'-Fla-3**	500	26714	595	3193			
**3'-Fla-3'**	489	41719	533	1688			

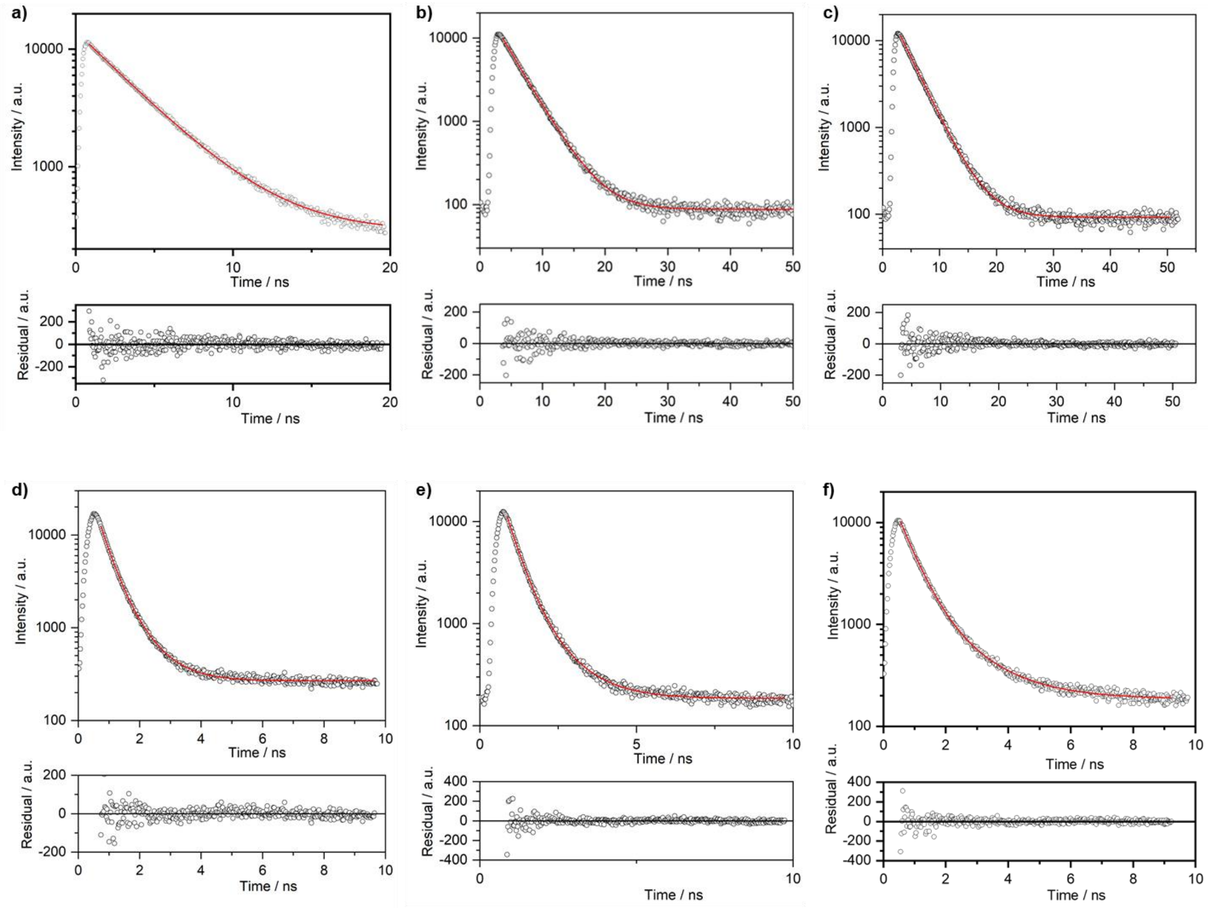

Figure S20 Fluorescence decay of a) **V-Fla-1**, b) **iV-Fla-1**, c) **2-Fla-1**, d) **V-Fla-2**, e) **iV-Fla-2** and f) **2-Fla-2** in solution.

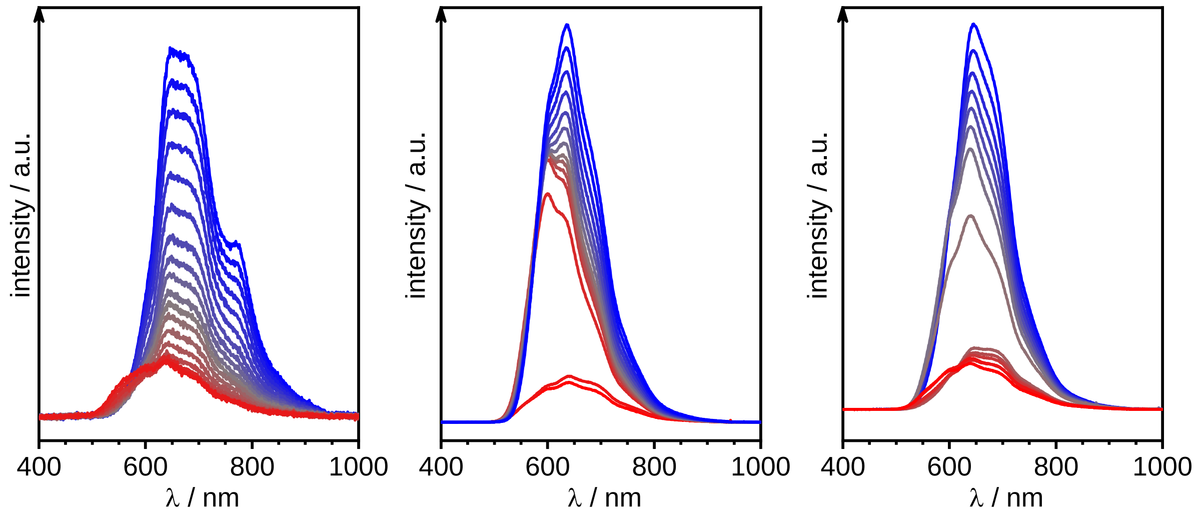

Figure S21 Temperature dependent solid-state luminescence of **V-Fla-1**, **iV-Fla-1** and **2-Fla-1**. The temperature decreases from red to blue.

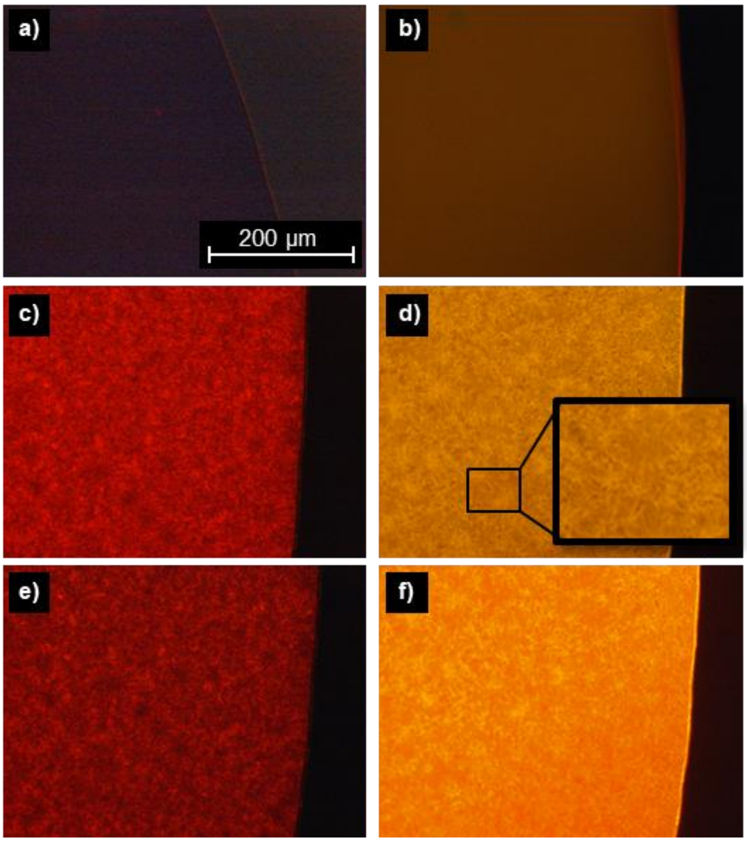

Figure S22 Microscope images of **iV-Fla-1** under white light with crossed polarizers (left column) and under irradiation with UV light (right column, exposure time: 4 seconds) at a-b) 150 °C, c-d) 130 °C and e-f) 30 °C.

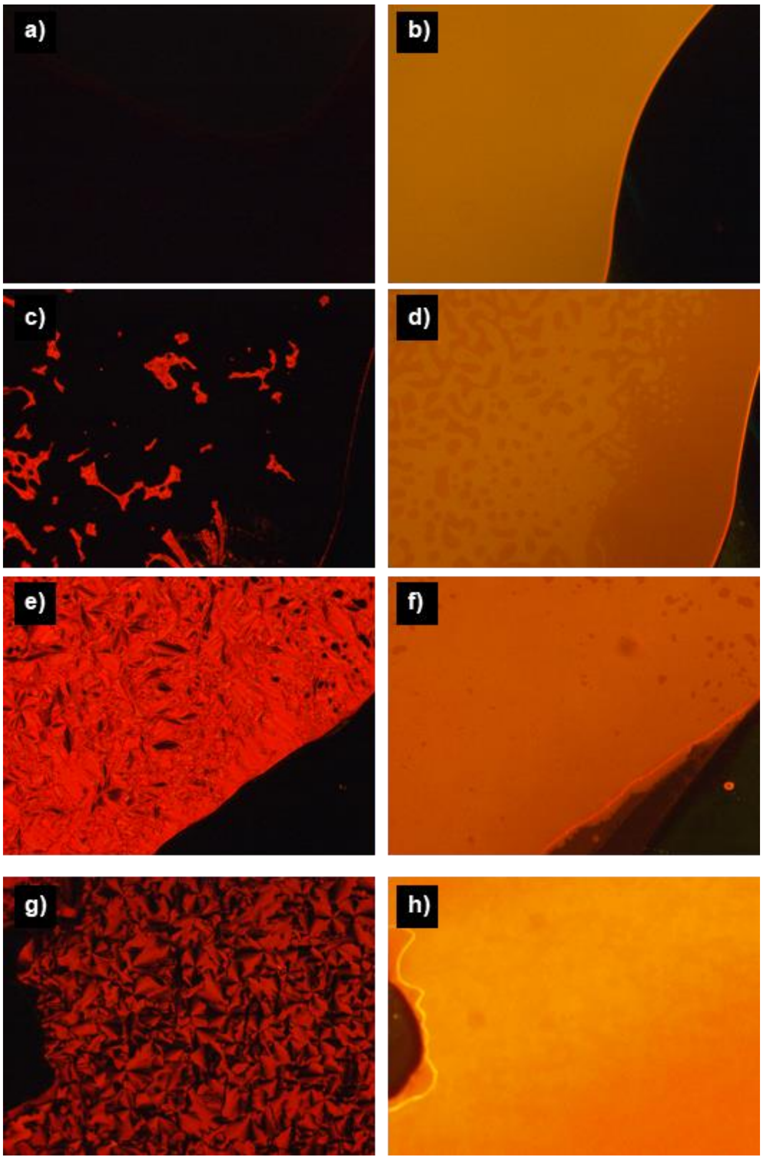

Figure S23 Microscope images of **2-Fla-1** under white light with crossed polarizers (left column) and under irradiation with UV light (right column, exposure time: 6 seconds) at a-b) 155 °C, c-d) 140 °C, e-f) 110 °C, and g-h) 70 °C.

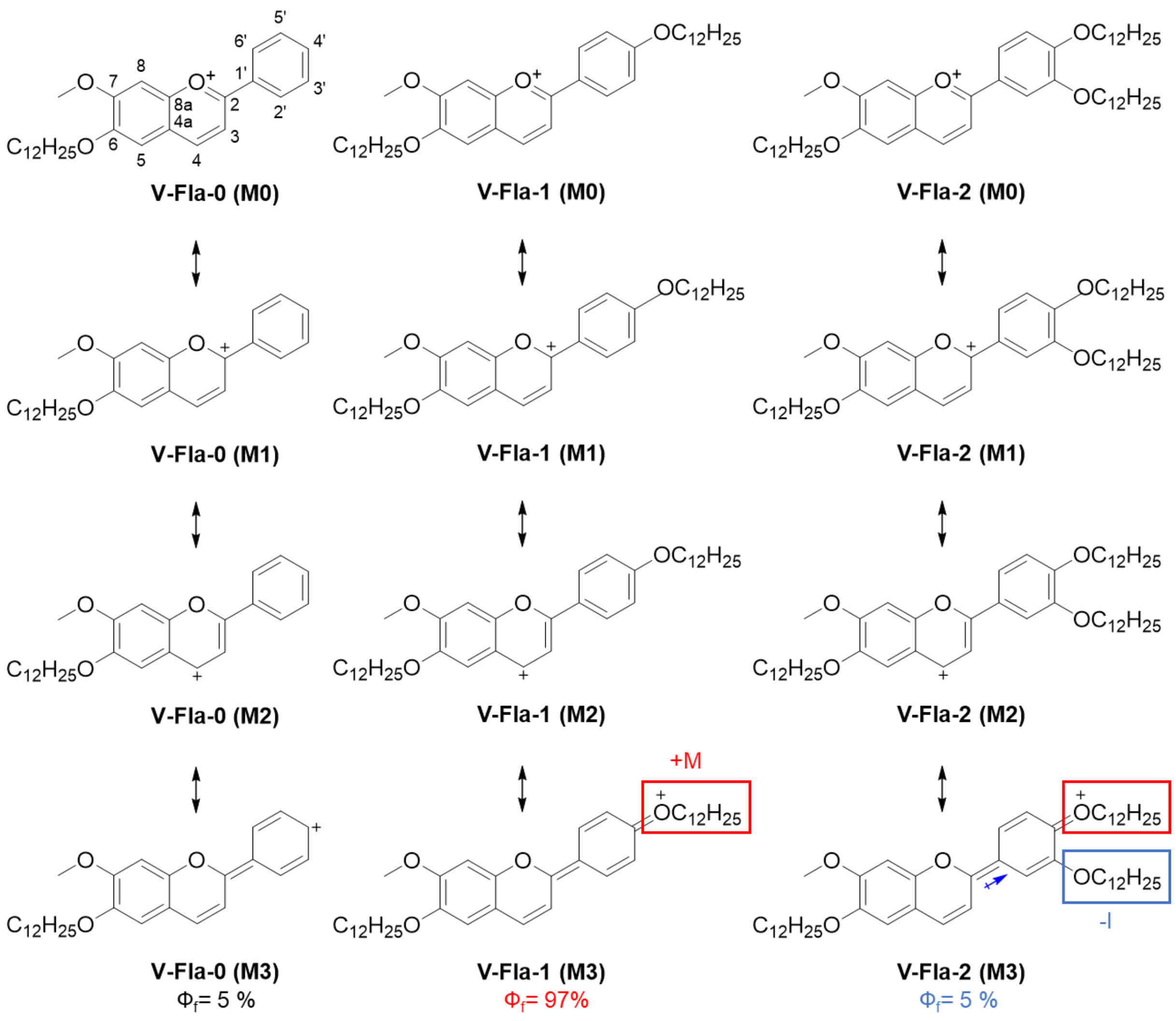

Figure S 24 Possible Lewis structures of flavylium salts **V-Fla-0**, **V-Fla-1** and **V-Fla-2**.

A quick quantum chemical survey

We have carried out a preliminary investigation of three selected Flavylium ions **V-Fla-0**, **V-Fla-1** and **V-Fla-2**, replacing the long alkyl chains by methyl groups. This replacement reduces the conformational complexity, while it is expected to be of minor influence on the photophysical properties of the chromophore. All investigations were carried out with the Turbomole program package[19] and are based on density functional theory (DFT), while excited state energies and oscillator strength (velocity gauge) rely on the time-dependent DFT (TDDFT) formalism. The B3LYP functional,[20] the D3 dispersion correction with Becke-Johnson damping,[21] and the def2-TZVPP basis set[22] were used. Visualizations of the structures and orbitals were carried out with Tmolex.[23] Only the conformations as shown in Figure S25 along with the highest occupied molecular orbitals (HOMO) and lowest unoccupied molecular orbitals (LUMO) were considered here. For convenience, we started with planar

structures (C_s point group symmetry). Interestingly, this assumption is confirmed by a normal mode analysis for **V-Fla-1** and **V-Fla-2**, while for **V-Fla-0** the ground state exhibits a small imaginary frequency for the torsion of the phenyl group. Reoptimization of **V-Fla-0** in C_1 indicates a very shallow torsional potential and an equilibrium torsion angle of around 7°, in agreement with previous computational studies.[24] Excitation energies were computed for the C_s constrained structures and geometry optimizations were also carried out for the excited state (again under C_s constraint). The estimates for the excitation energies are listed in Table S4. We find a very good correlation of the vertical excitation energies (computed for the ground state equilibrium structure) of **V-Fla-0** and **V-Fla-2** with the experimental absorption maxima, and of the vertical emission energies (computed for the excited state equilibrium structures) with the experimental emission maxima. For **V-Fla-1**, some deviations are found, in line with the different line shape of these spectra: While **V-Fla-0** and **V-Fla-2** show broad spectra indicative of many weak degrees of freedom contributing to the Franck-Condon profile, the spectrum of **V-Fla-1** is more reminiscent of the spectra of more rigid molecules with large π-system, which are often dominated by the 0-0 transition. The adiabatic excitation energies (minimum to minimum energy differences of two electronic states) given in Table S4 can be used an approximation to the 0-0 transition and in fact the adiabatic energy of **V-Fla-1** is in viable agreement with the experimentally observed absorption maximum. Another interesting observation is the oscillator strength of **V-Fla-1**, which is stronger than those of **V-Fla-0** and **V-Fla-2**, in particular in the excited state geometry (most relevant for emission).

We note, however, that our comparison is hampered by not considering solvent effects and conformational averaging, nonetheless the comparison reveals that the chosen approach (B3LYP/def2-TZVPP) gives reasonable energetics.

Table S5 shows the computed structure changes (ground state equilibrium bond distances compared to excited state equilibrium bond distances), while keeping the C_s symmetry constraint. Most evident is the strong increase of the 2-1' interring bond distance by more than 6 pm for **V-Fla-2**, which is not observed for **V-Fla-0** and **V-Fla-1**. This indicates a much reduced π-interaction between the rings in the excited state, possibly followed by a partial ring twist (not yet investigated). In any way, the computations show that there is a very subtle balance of substituent effects that enhance or diminish the conjugation between the two ring systems. For **V-Fla-1**, both ground and excited state seem to remain rather rigid, explaining the spectral shape and the enhanced fluorescence. All other flavylium salts are either non-rigid in

the ground state (like **V-Fla-0**) or become non-rigid in the excited state, which likely leads to additional non-radiative pathways.

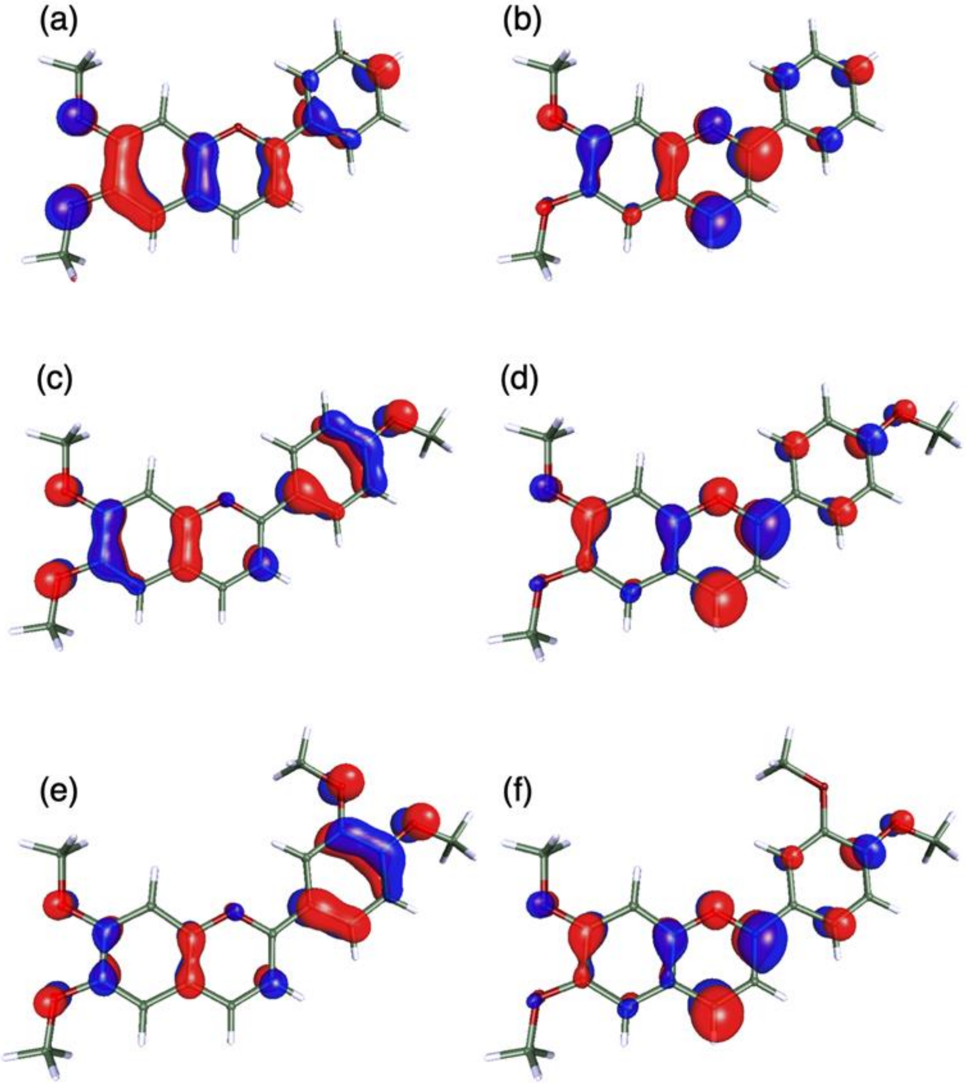

Figure S25 HOMO (a, c, e) and LUMO (b, d, f) of **V-Fla-0** (a, b), **V-Fla-1** (c, d) and **V-Fla-2** (e, f) obtained by DFT calculations (B3LYP, def2-TZVPP, alkoxy groups were replaced by methoxy).

Table S4 Photophysical properties computed at the DFT/TDDFT level (functional B3LYP, basis def2-TZVPP). Vertical absorption means the vertical energy difference between the S0 and S1 surfaces at the ground state equilibrium geometry, vertical emission is the vertical energy difference at the excited state equilibrium geometry. The adiabatic excitation is the minimum to minimum energy difference. In all cases only one conformation is considered.

	**V-Fla-0**	**V-Fla-1**	**V-Fla-2**
Calc. vert. Abs. (eV)	2.87	2.77	2.54
Calc. vert. Abs. (nm)	432	448	488
Calc. osc.Str.	0.48	0.74	0.59
Exp. abs. max (eV)	2.59	2.56	2.47
Exp. abs. max (nm)	444	485	501
Calc. vert. Em. (eV)	2.52	2.55	2.12
Calc. vert. Em. (nm)	486	491	585
Osc.Str.	0.30	0.61	0.31
Exp. em. max (eV)	2.46	2.38	2.18
Exp. em. max (nm)	505	521	570
Adiabatic Exc. (eV)	2.71	2.65	2.36
Adiabatic Exc. (nm)	457	467	526

Table S5 Structure changes of the bond lengths r (in pm) of the ground state S0 upon excitation into the excited state S1, computed at the DFT/TDFT level (functional B3LYP, basis def2-TZVPP).

	**V-Fla-0**		**V-Fla-1**		**V-Fla-2**	
Bond	r(S0)	Δr(S1)	r(S0)	Δr(S1)	r(S0)	Δr(S1)
2-1'	144.90	-1.65	143.60	0.65	143.42	6.10
1-2	133.77	4.83	134.01	3.74	134.11	2.09
2-3	138.99	-0.04	139.63	-1.59	139.80	-4.57
3-4	138.08	-0.33	137.58	1.31	137.40	4.48
4-4a	140.05	3.10	140.50	1.86	140.68	-1.17
4a-5	141.45	-1.05	141.36	-1.42	141.30	0.38
5-6	137.26	4.02	137.36	2.21	137.40	0.10
6-7	144.60	-0.86	144.26	-0.11	144.11	-0.42
7-8	138.72	-1.05	138.71	-0.43	138.71	0.26
8-8a	138.59	1.80	138.63	0.56	138.68	-0.34
8a-1	135.32	1.62	135.65	0.80	135.80	0.75
8a-4a	141.03	-2.07	140.65	0.35	140.50	1.48
1'-2'	140.51	0.92	141.26	0.32	141.42	-3.26
2'-3'	138.29	-0.05	137.06	0.24	137.70	4.14
3'-4'	139.22	-0.04	140.66	-0.02	142.74	0.08
4'-5'	139.18	0.61	140.31	0.64	139.52	-1.07
5'-6'	138.30	-0.50	137.86	-0.26	138.18	1.06
6'-1'	140.51	0.86	140.60	0.51	140.12	-0.03
7-O	132.15	1.31	132.46	0.68	132.62	0.70
6-O	133.23	-1.38	133.49	-0.92	133.58	0.25
4'-O			133.27	-0.02	132.85	0.68
3'-O					134.25	-2.02

2 References

[1] Soft Imaging System, AnalySIS 3.2, **2002**.

[2] STARe 7.01, **2006**.

[3] Bruker, SAXS 4.1.26, **2006**.

[4] P. Heiney, Datasqueeze 2.2, **2011**.

[5] I. Vasilief, QtiPlot 0.9.8.9, **2011**.

[6] OriginLab Cooperation, OriginPro 9.1.0 Sr2 B271, **2011**.

[7] N. Godbert, A. Crispini, M. Ghedini, M. Carini, F. Chiaravalloti, A. Ferrise, *J. Appl. Crystallogr.* **2014**, *47*, 668–679.

[8] R. J. Bergeron, J. Wiegand, J. S. McManis, N. Bharti, S. Singh, *J. Med. Chem.* **2008**, *51*, 5993–6004.

[9] S. Chassaing, M. Kueny-Stotz, G. Isorez, R. Brouillard, *Eur. J. Org. Chem.* **2007**, *2007*, 2438–2448.

[10] M. Frigerio, M. Santagostino, S. Sputore, *J. Org. Chem.* **1999**, *64*, 4537–4538.

[11] A. D. Bromby, R. P. Jansonius, T. C. Sutherland, *J. Org. Chem.* **2013**, *78*, 1612–1620.

[12] Q. Zhang, H. Peng, G. Zhang, Q. Lu, J. Chang, Y. Dong, X. Shi, J. Wei, *J. Am. Chem. Soc.* **2014**, *136*, 5057–5064.

[13] K. Praefcke, D. Singer, B. Gündogan, *Mol. Cryst. Liq. Cryst. Sci. Technol. Sect. A. Mol. Cryst. Liq. Cryst.* **1992**, *223*, 181–195.

[14] R. Judele, S. Laschat, A. Baro, M. Nimtz, *Tetrahedron* **2006**, *62*, 9681–9687.

[15] M. Lehmann, I. Fischbach, H. W. Spiess, H. Meier, *J. Am. Chem. Soc.* **2004**, *126*, 772–784.

[16] H. D. Becker, A. Bjoerk, E. Adler, *J. Org. Chem.* **1980**, *45*, 1596–1600.

[17] E. Westphal, I. H. Bechtold, H. Gallardo, *Macromolecules* **2010**, *43*, 1319–1328.

[18] E. Terazzi, L. Guénée, P.-Y. Morgantini, G. Bernardinelli, B. Donnio, D. Guillon, C. Piguet, *Chem. - A Eur. J.* **2007**, *13*, 1674–1691.

[19] Turbomole V7.1 2017, a development of University of Karlsruhe and Forschungszentrum Karlsruhe GmbH, 1989-2007, TURBOMOLE GmbH, since 2007; available from http://www.turbomole.com. (b) F. Furche, R. Ahlrichs, C. Hättig, W. Klopper, M. Sierka, and F. Weigend, *WIREs Comput. Mol. Sci.* **2013**, *4*, 91-100.

[20] a) A. D. Becke, *J. Chem. Phys.* **1993**, *98*, 5648-5652; b) C. Lee, W. Yang, R. G. Parr, *Phys. Rev. B* **1988**, *37*, 785-789; c) B. Miehlich, A. Savin, H. Stoll, H. Preuss, *Chem. Phys. Lett.* **1989**, *157*, 200-206.

[21] a) S. Grimme, J. Antony, S. Ehrlich, H. Krieg, *J. Chem. Phys* .**2010**, *132*, 154104-1-154104-19; b) S. Grimme, S. Ehrlich, L. Goerigk, *J. Comput. Chem.* **2011**, *32*, 1456-1465.

[22] F. Weigend, R. Ahlrichs, *Phys. Chem. Chem. Phys.* **2005**, *7*, 3297-3305

[23] C. Steffen, K. Thomas, U. Huniar, A. Hellweg, O. Rubner, and A. Schroer, *J. Comput. Chem.* **2010**, *31*, 2967-2970 (2010).

[24] J. N. Woodford, *Chem. Phys. Lett.* **2005**, *410*, 182–187.

3 ^{1}H and ^{13}C NMR spectra

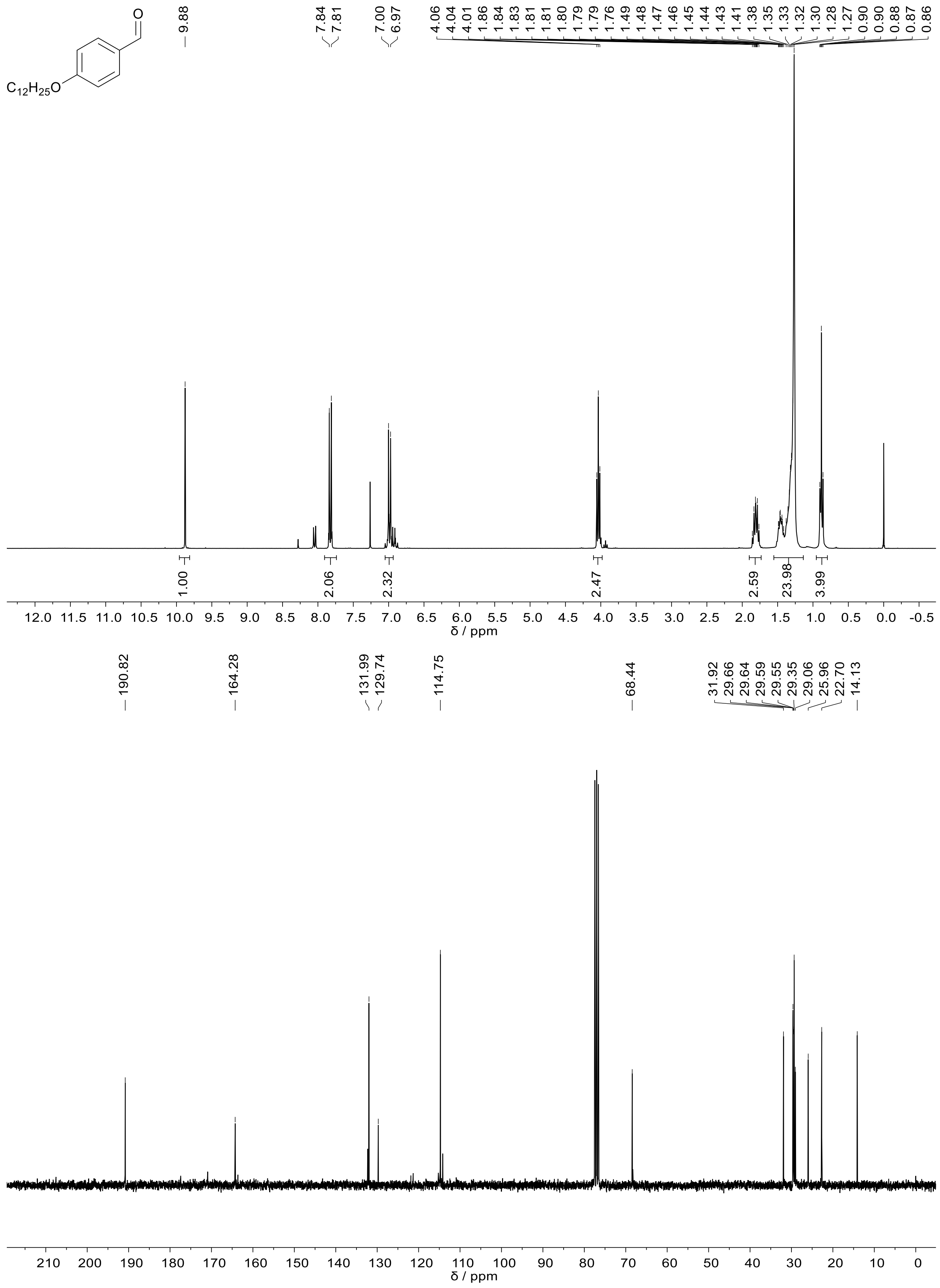

Figure S 26: ^{1}H (top**, at 300 MHz**) and ^{13}C NMR (bottom**, at 75 MHz**) of 6a.

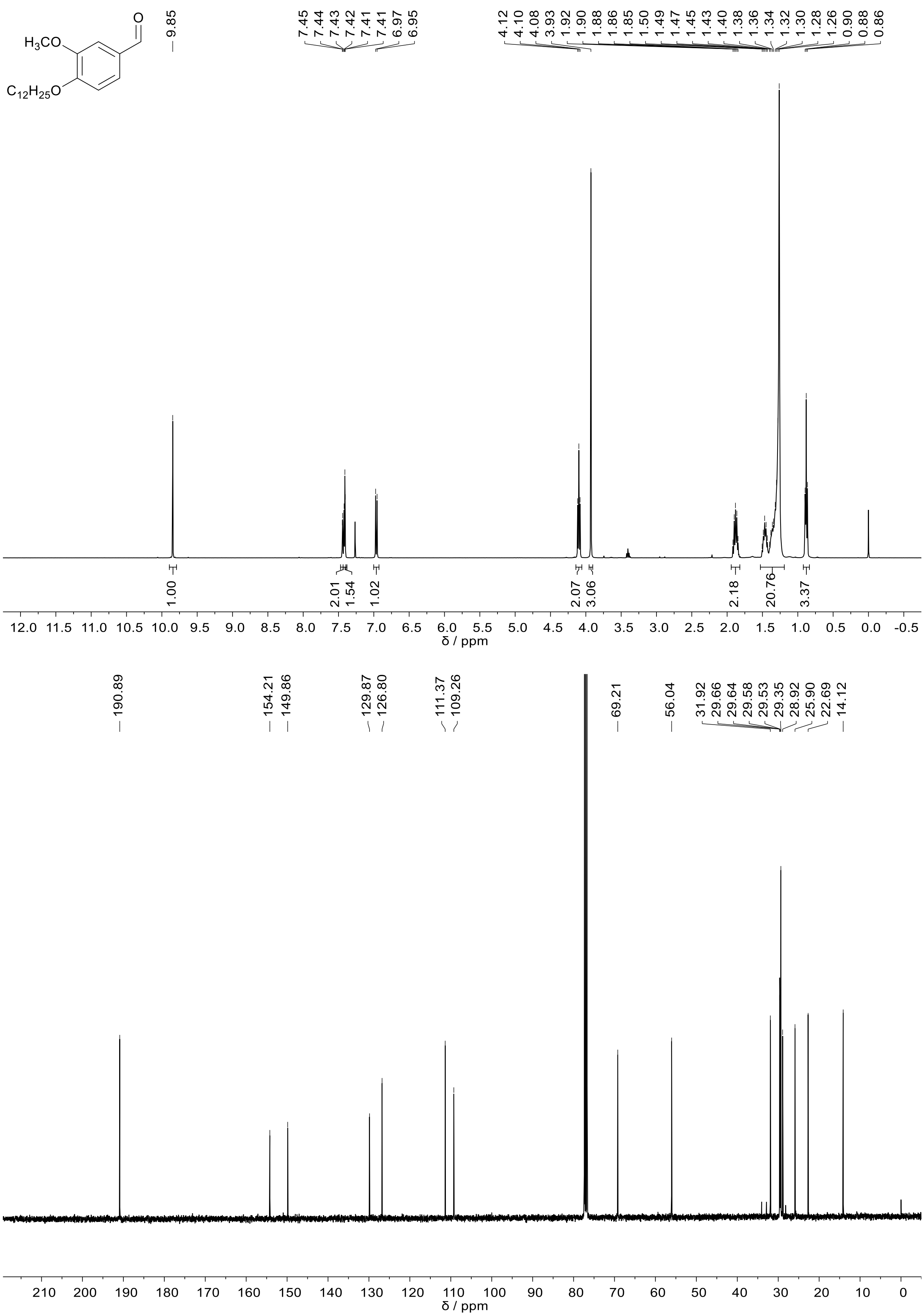

Figure S 27: ^{1}H (top, at 400 MHz) and ^{13}C NMR (bottom, at 101 MHz) of **6b**.

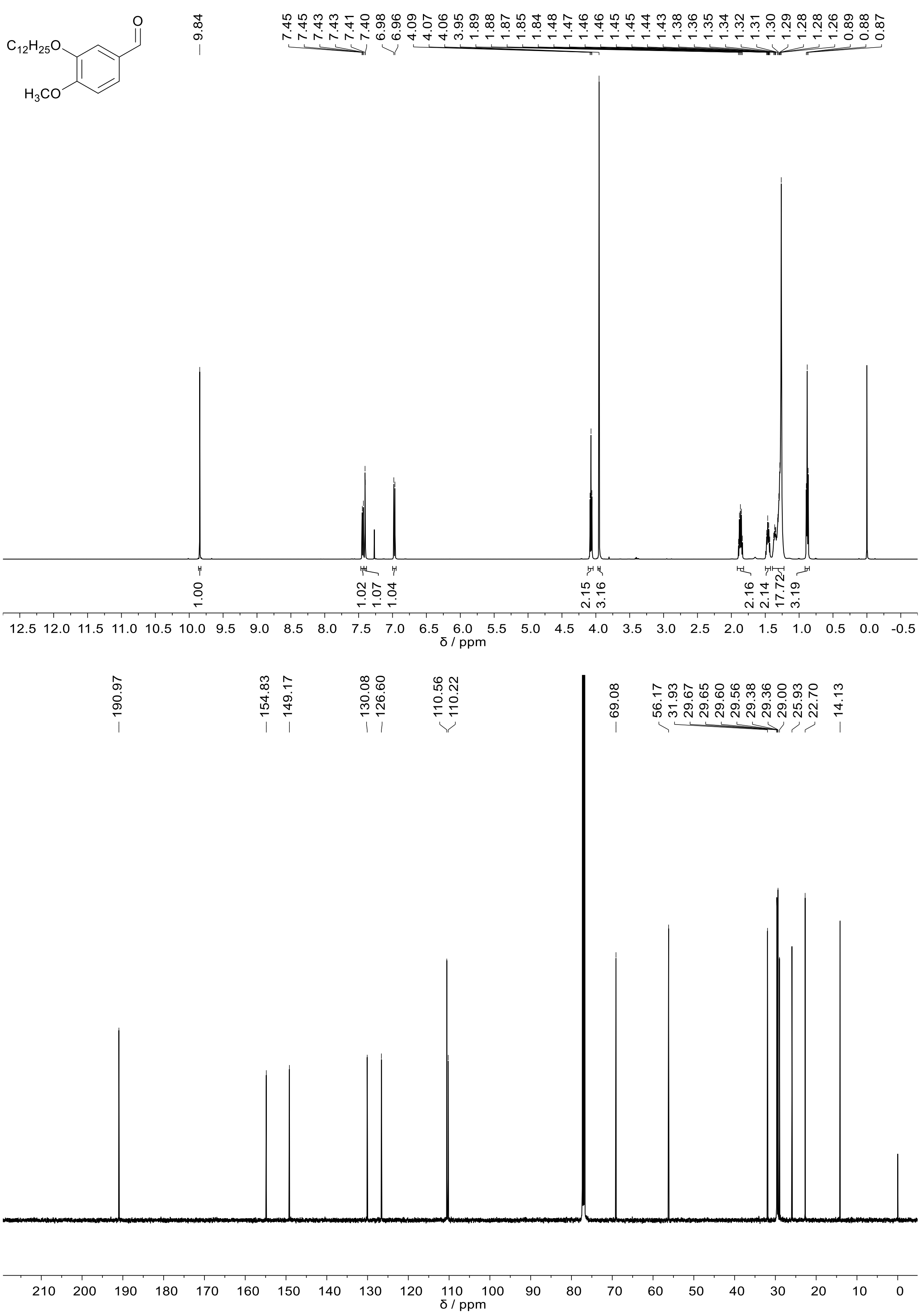

Figure S 28: ^{1}H (top, at 500 MHz) and ^{13}C NMR (bottom, at 126 MHz) of **6c**.

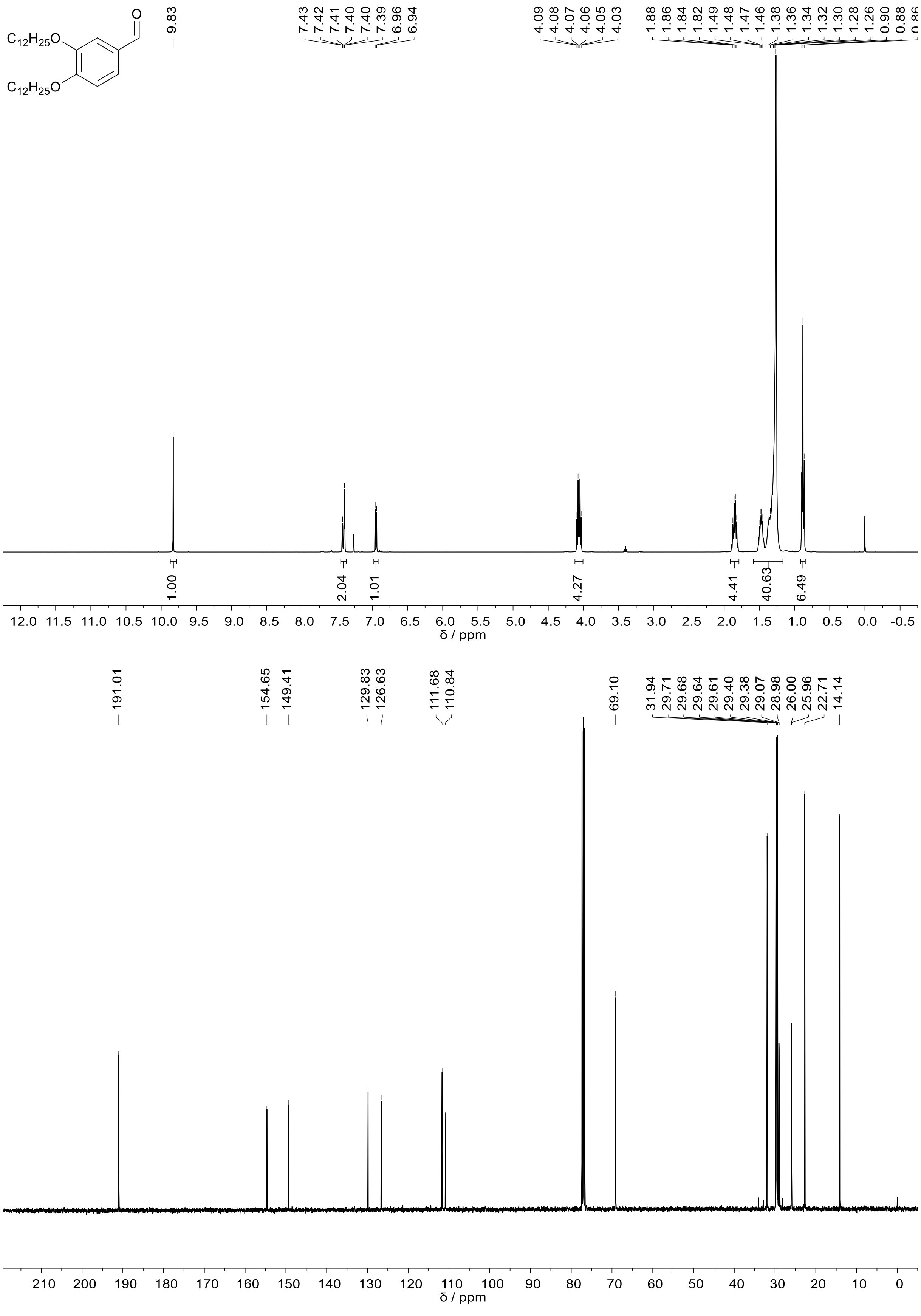

Figure S 29: ^{1}H (top, at 400 MHz) and ^{13}C NMR (bottom, at 101 MHz) of **6d**.

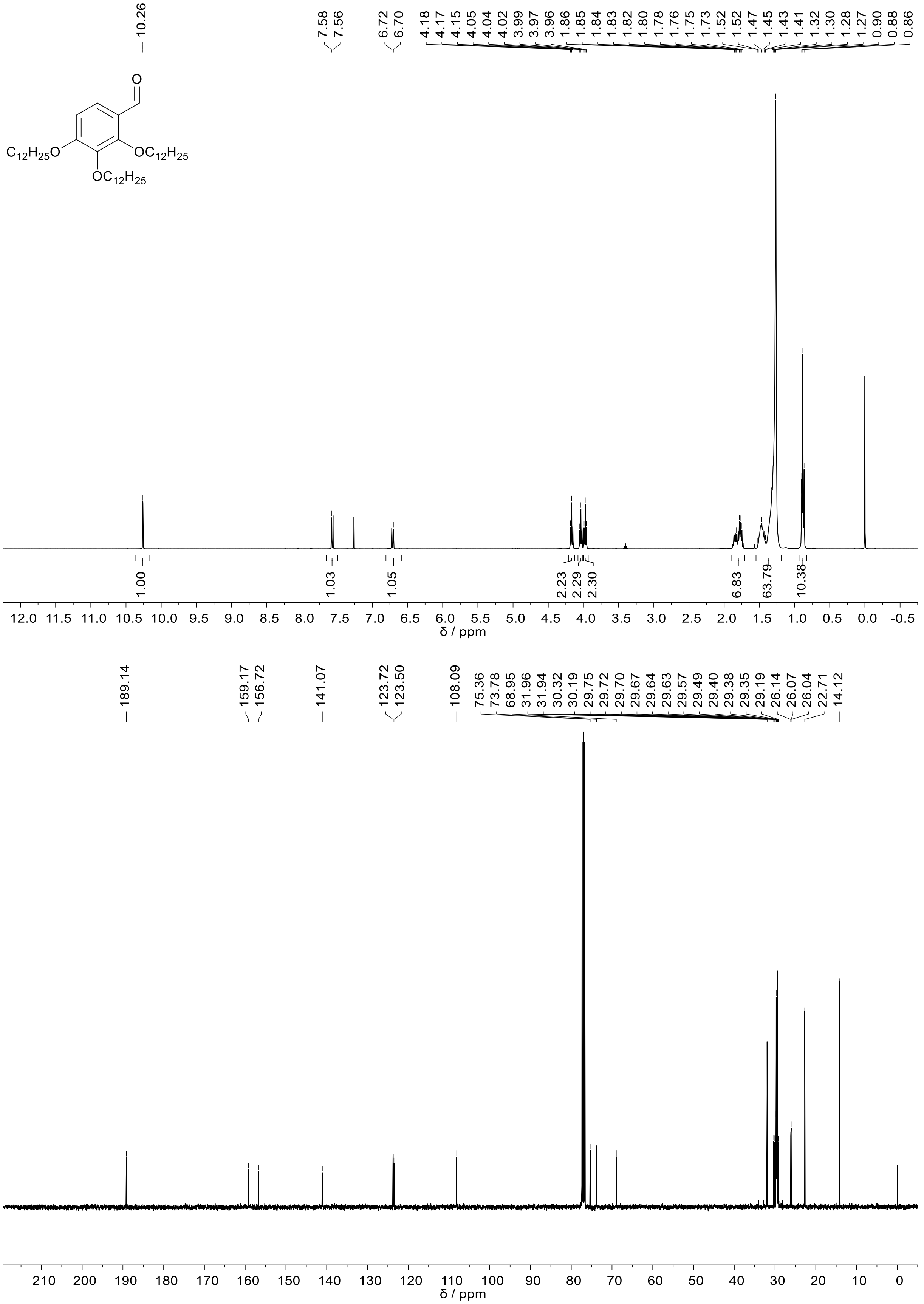

Figure S 30: ^{1}H (top, at 400 MHz) and ^{13}C NMR (bottom, at 101 MHz) of **6e**.

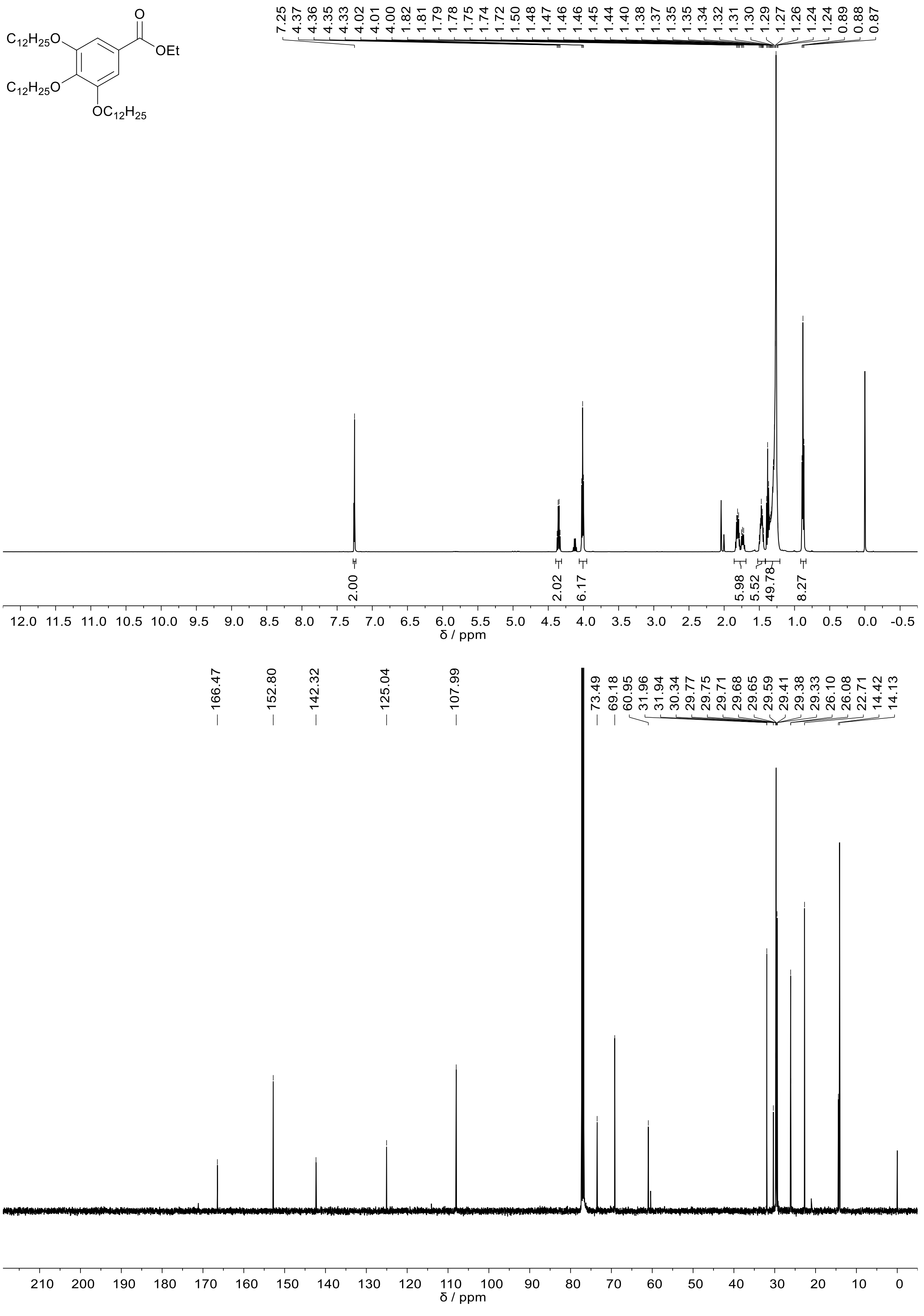

Figure S 31: ^{1}H (top, at 500 MHz) and ^{13}C NMR (bottom, at 126 MHz) of **2**.

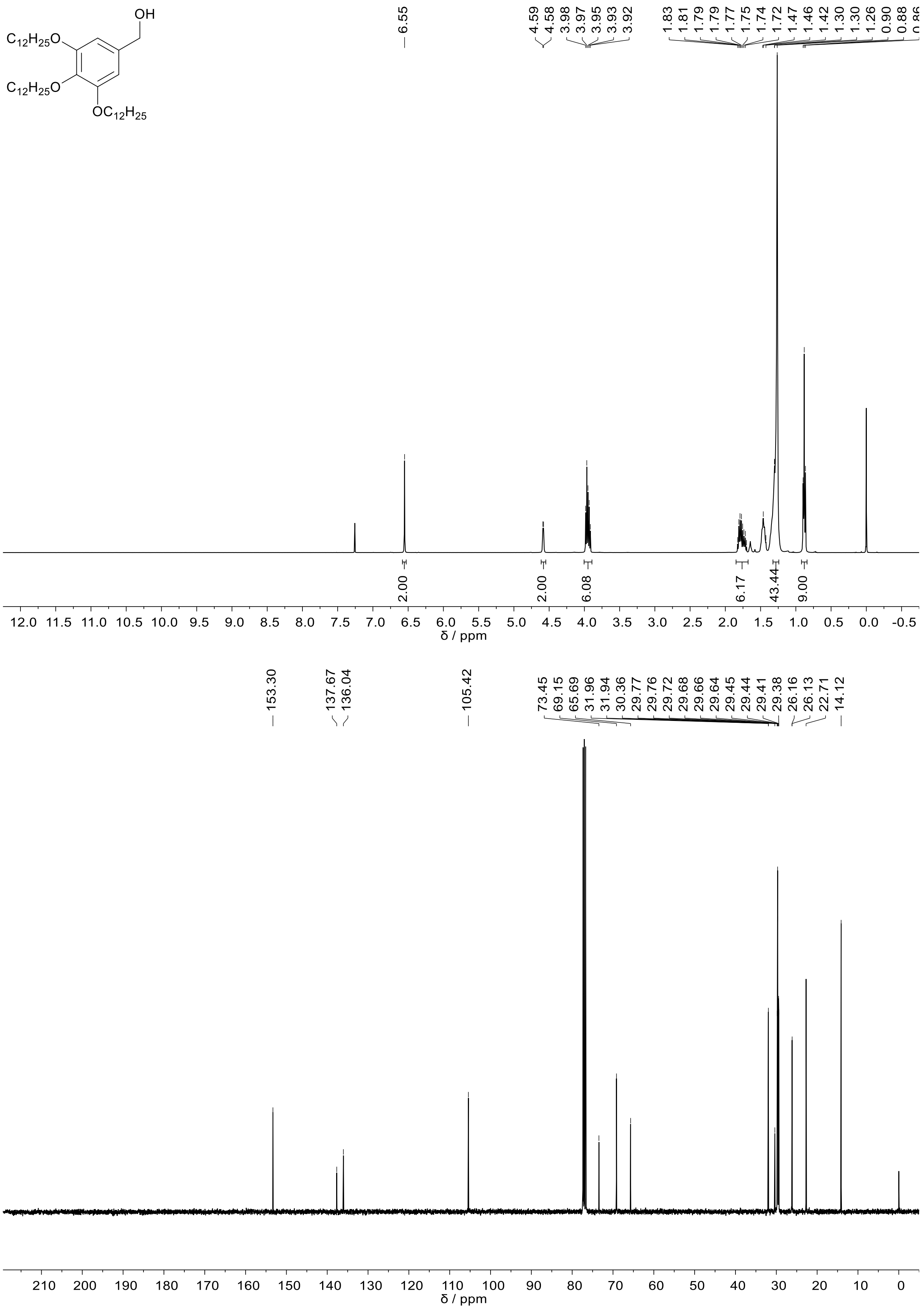

Figure S 32: ^{1}H (top, at 400 MHz) and ^{13}C NMR (bottom, at 101 MHz) of **3**.

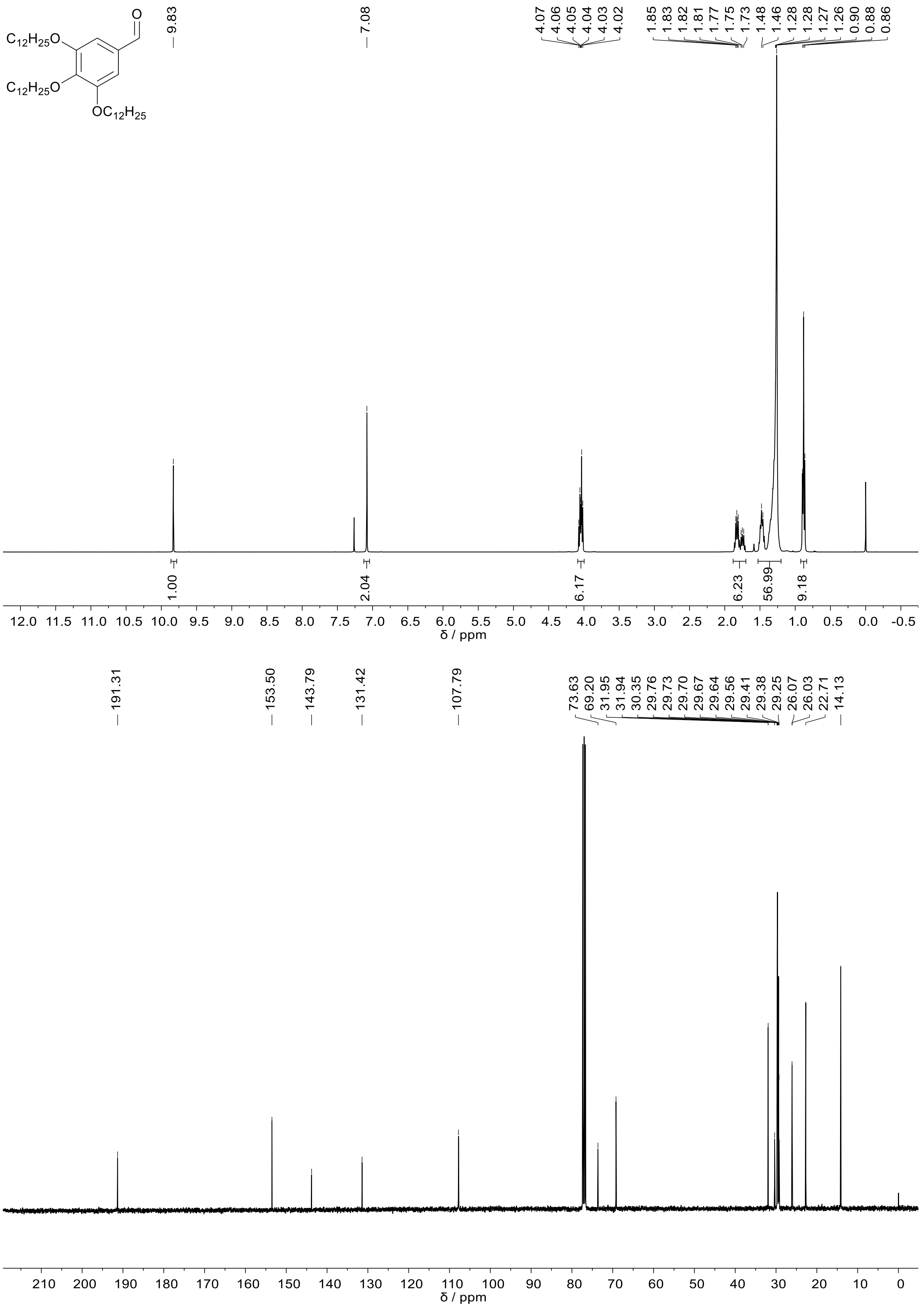

Figure S 33: ^{1}H (top, at 400 MHz) and ^{13}C NMR (bottom, at 101 MHz) of **6f**.

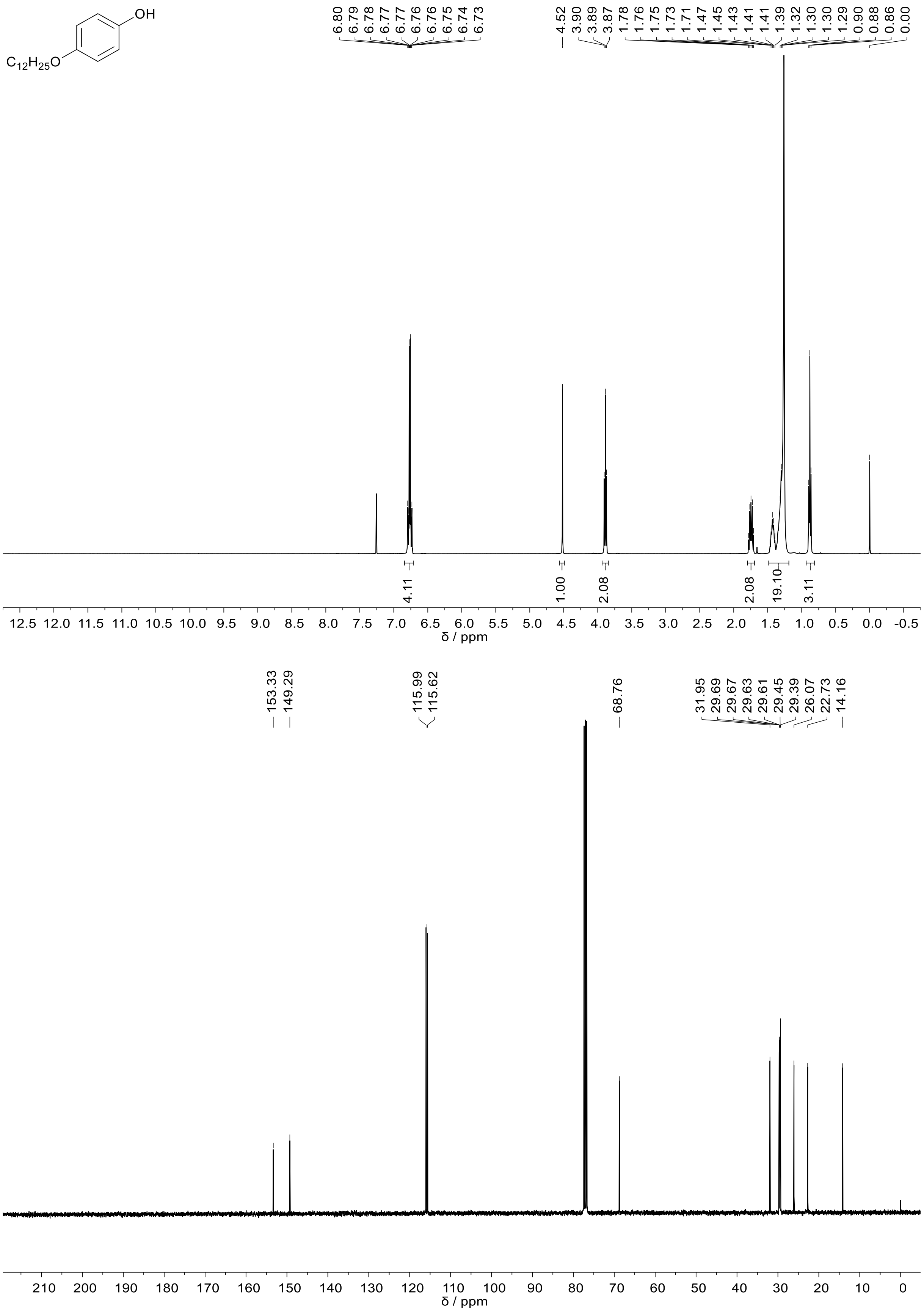

Figure S 34: ^{1}H (top, at 400 MHz) and ^{13}C NMR (bottom, at 101 MHz) of **7a**.

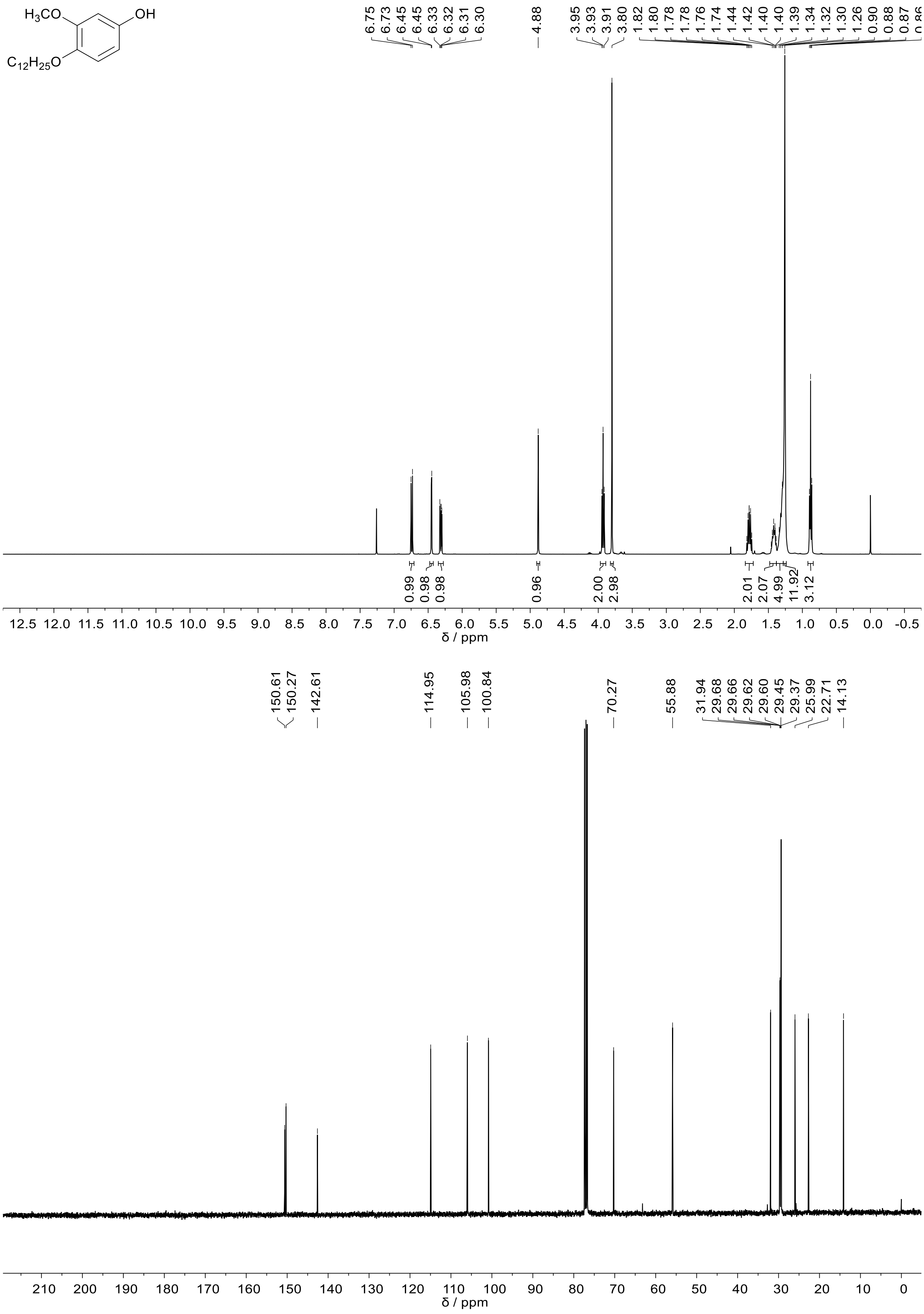

Figure S 35: ^{1}H (top, at 400 MHz) and ^{13}C NMR (bottom, at 101 MHz) of **7b**.

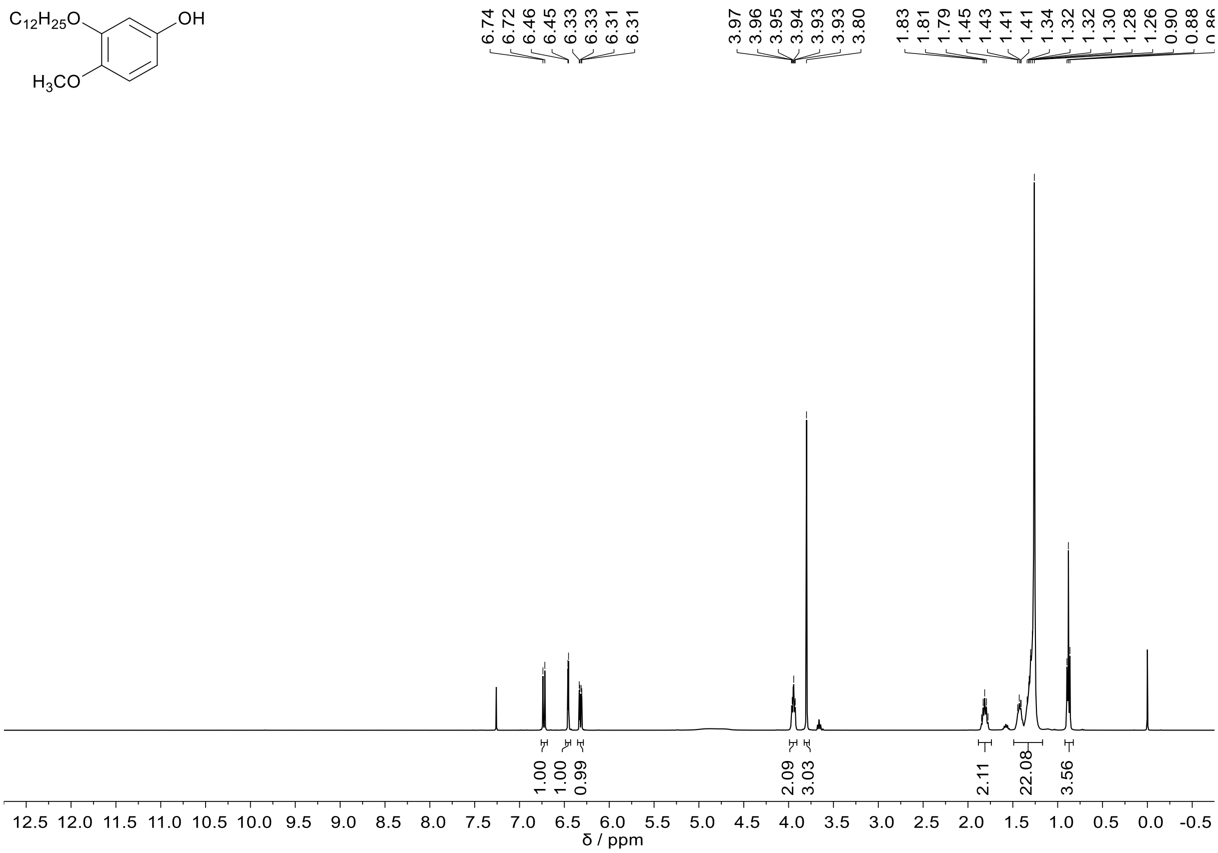

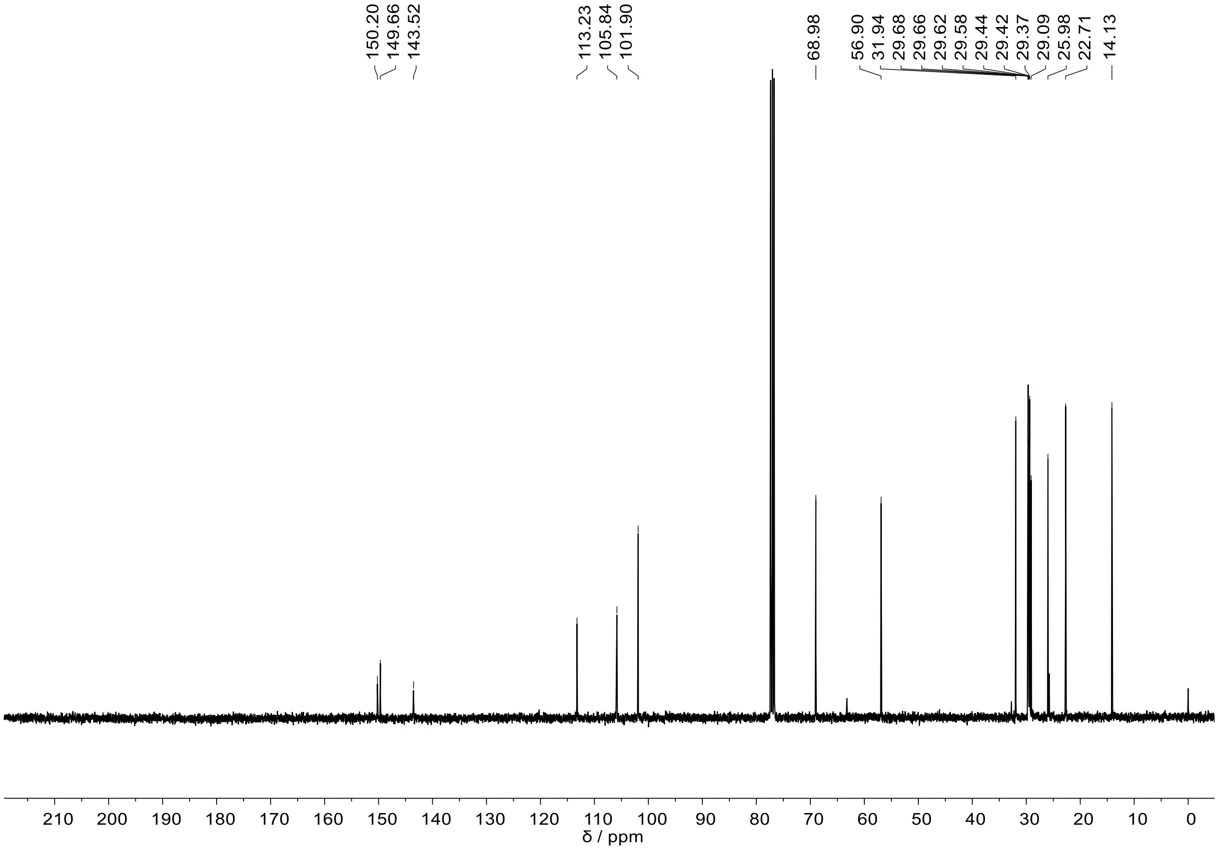

Figure S 36: ^{1}H (top, at 400 MHz) and ^{13}C NMR (bottom, at 101 MHz) of **7c**.

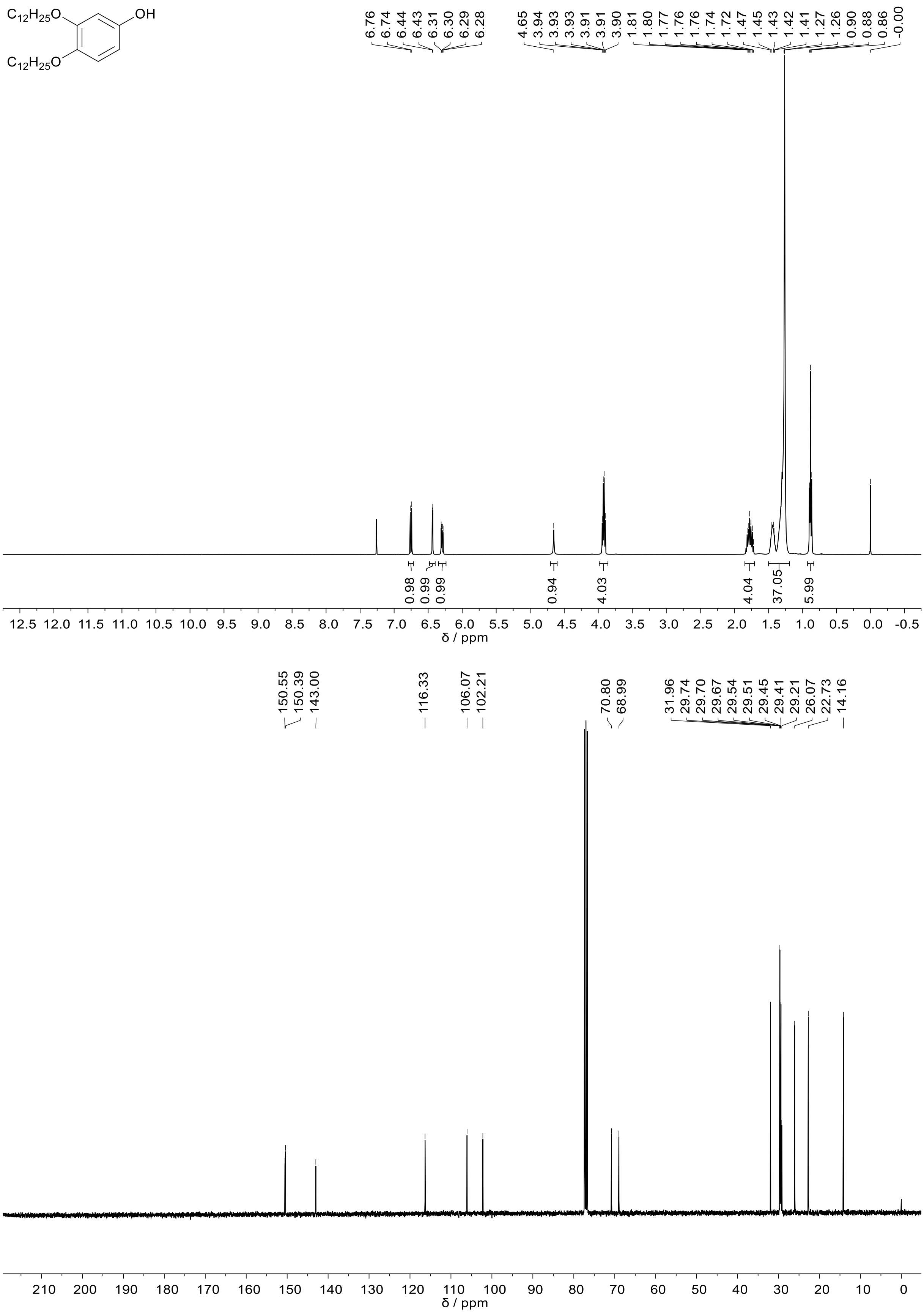

Figure S 37: ^{1}H (top, at 400 MHz) and ^{13}C NMR (bottom, at 101 MHz) of **7d**.

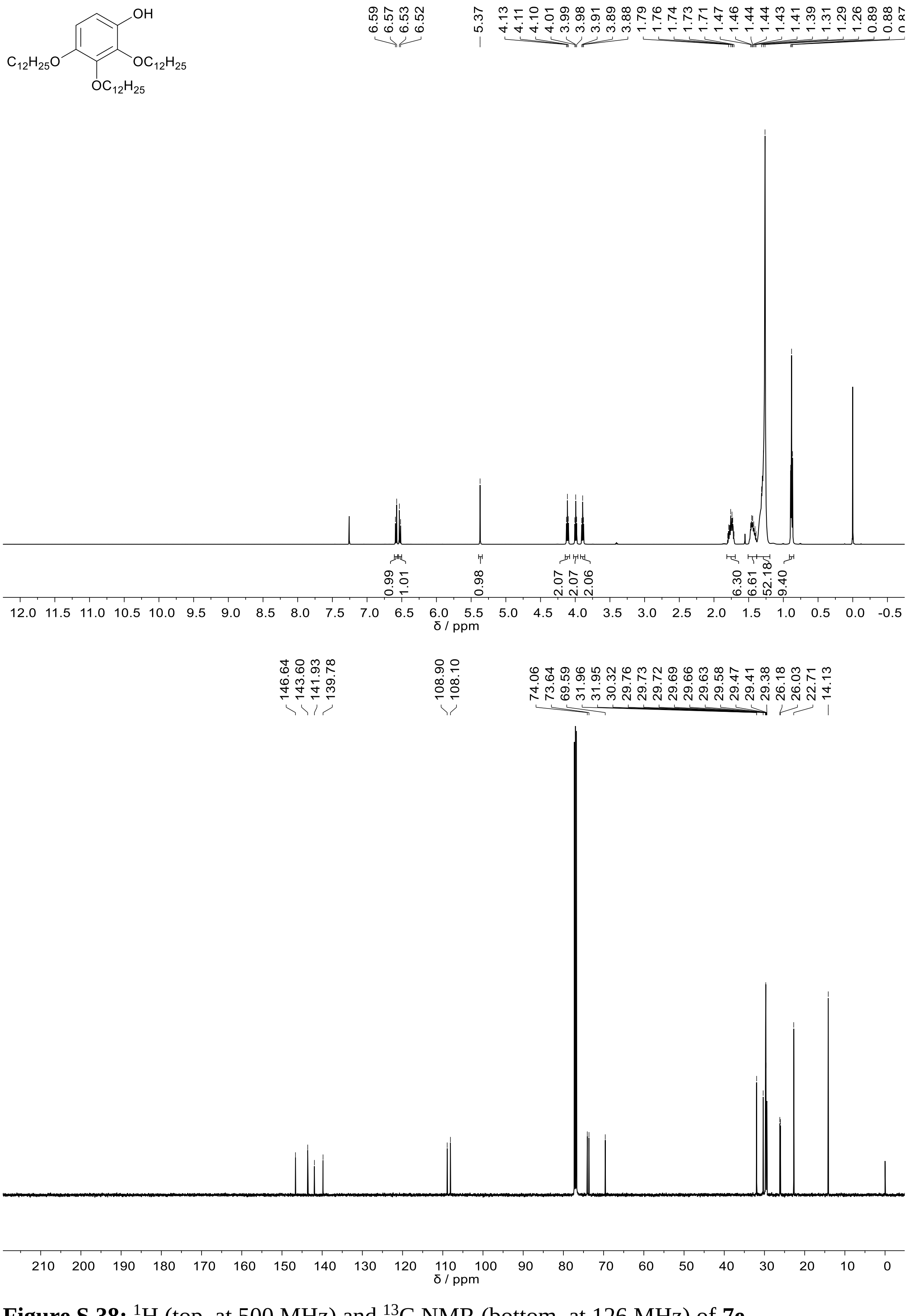

Figure S 38: ^{1}H (top, at 500 MHz) and ^{13}C NMR (bottom, at 126 MHz) of **7e**.

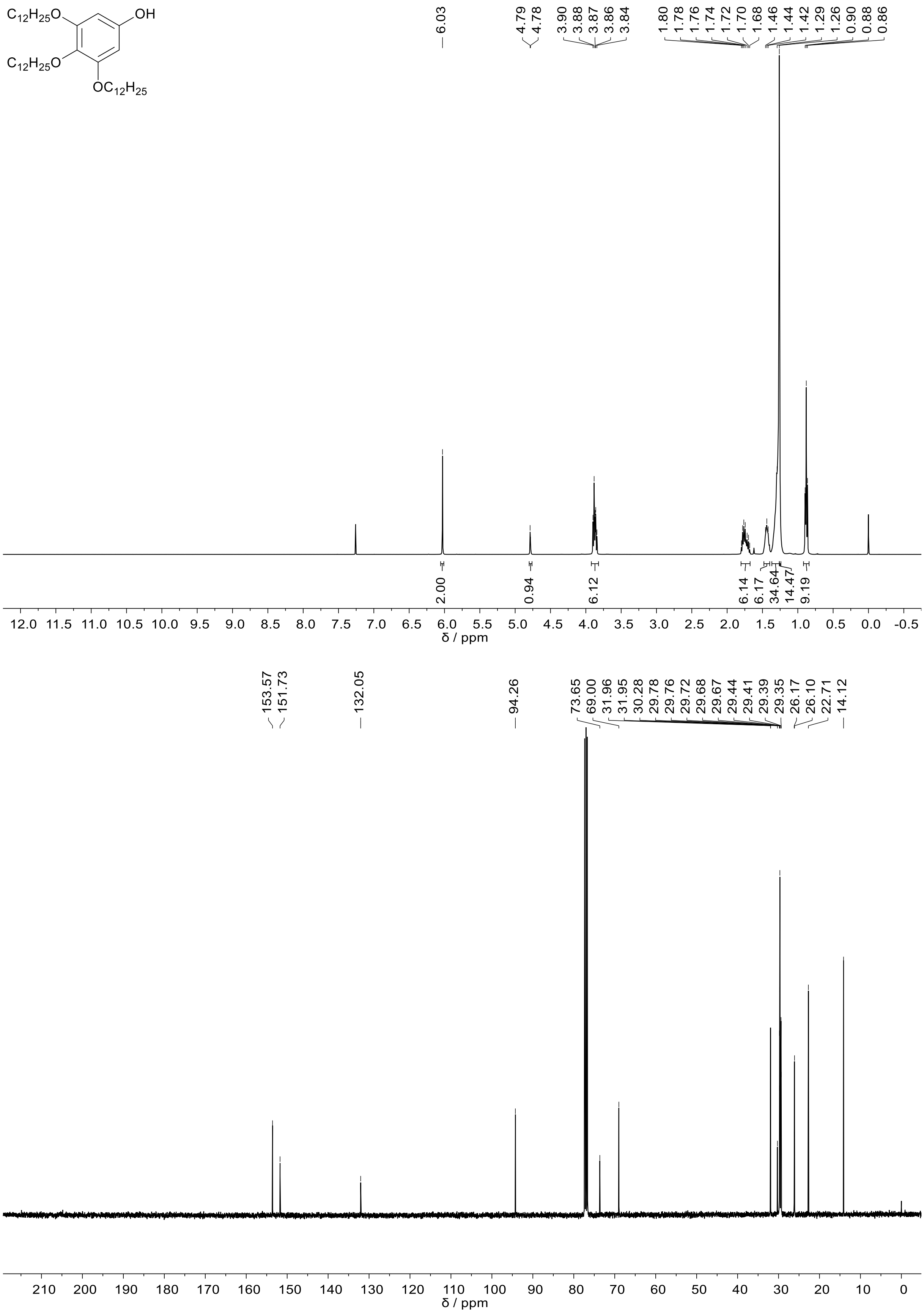

Figure S 39: ^{1}H (top, at 400 MHz) and ^{13}C NMR (bottom, at 101 MHz) of **7f**.

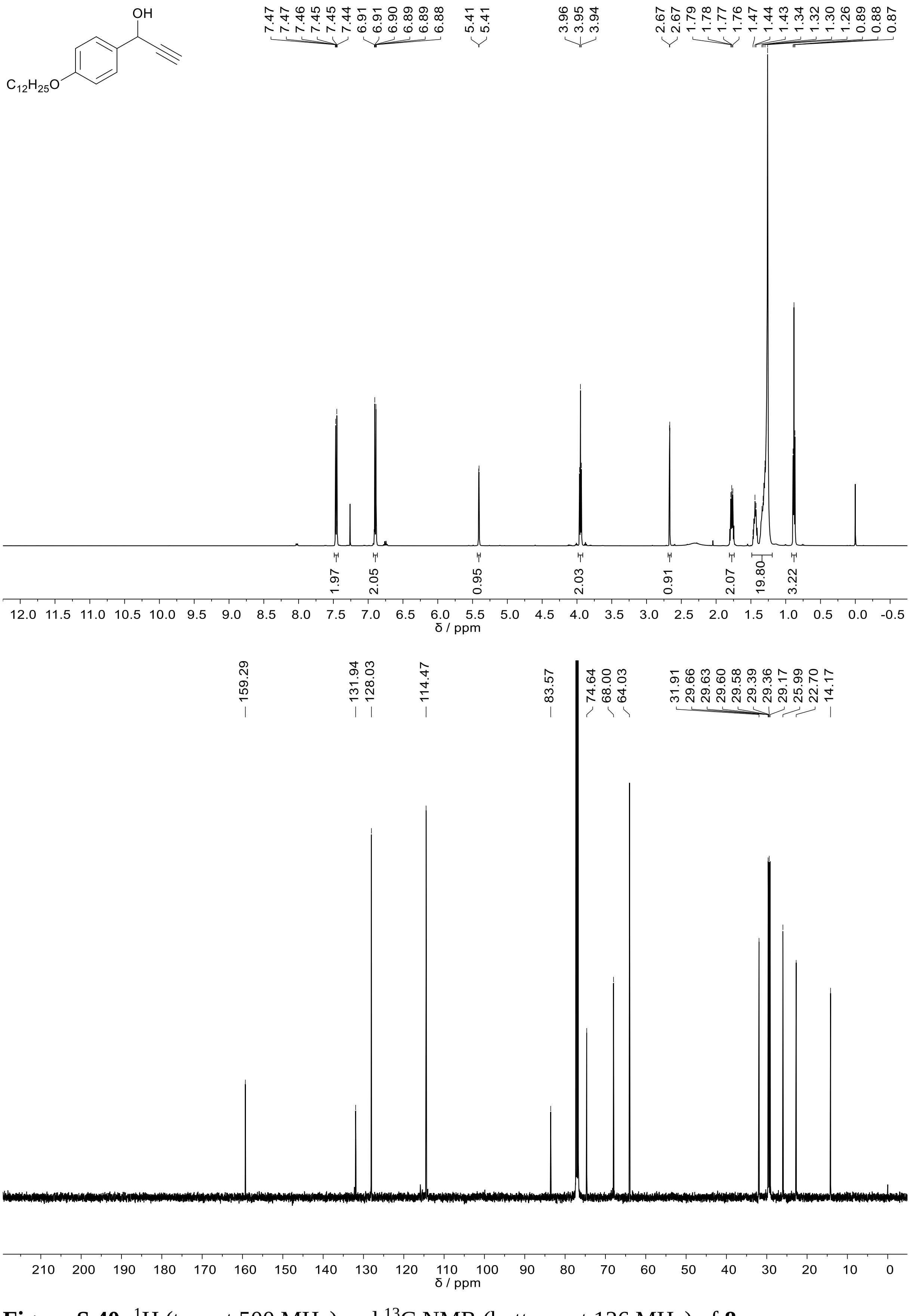

Figure S 40: ^{1}H (top, at 500 MHz) and ^{13}C NMR (bottom, at 126 MHz) of **8a**.

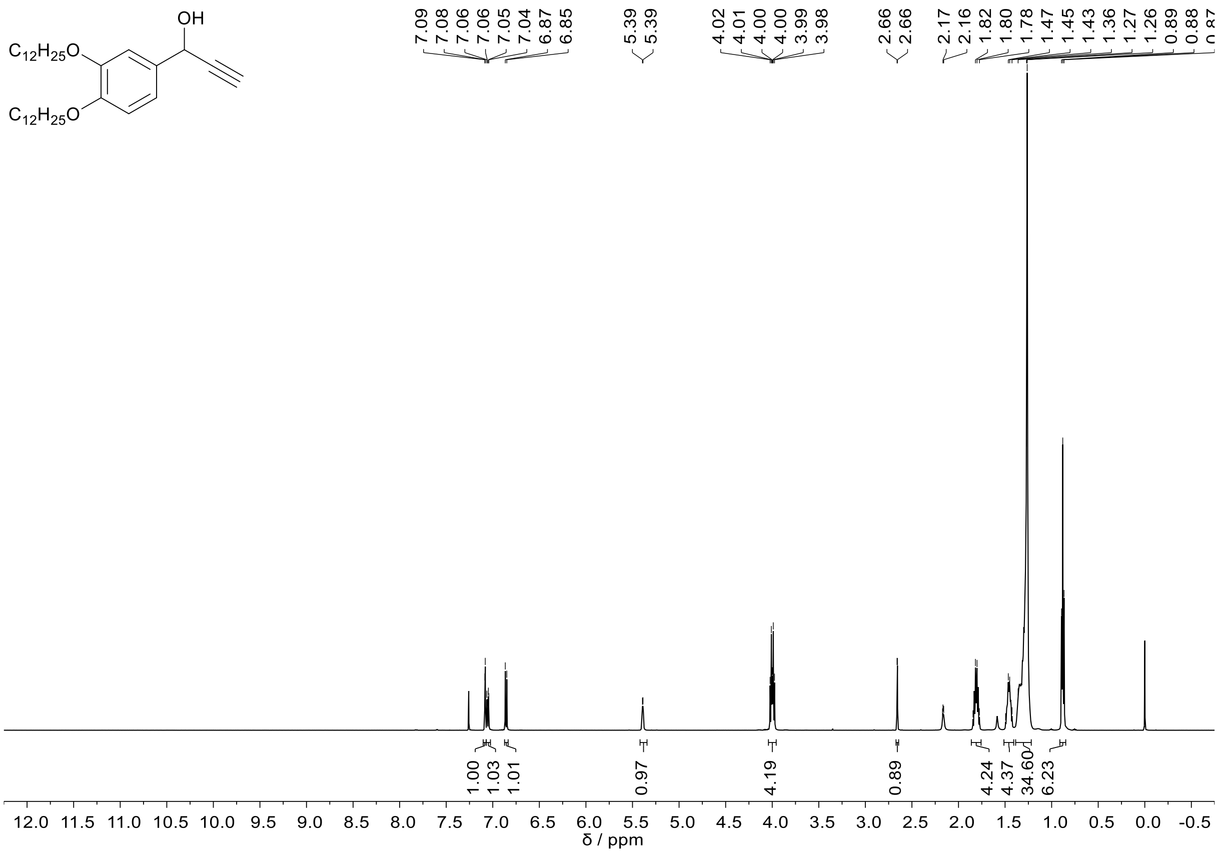

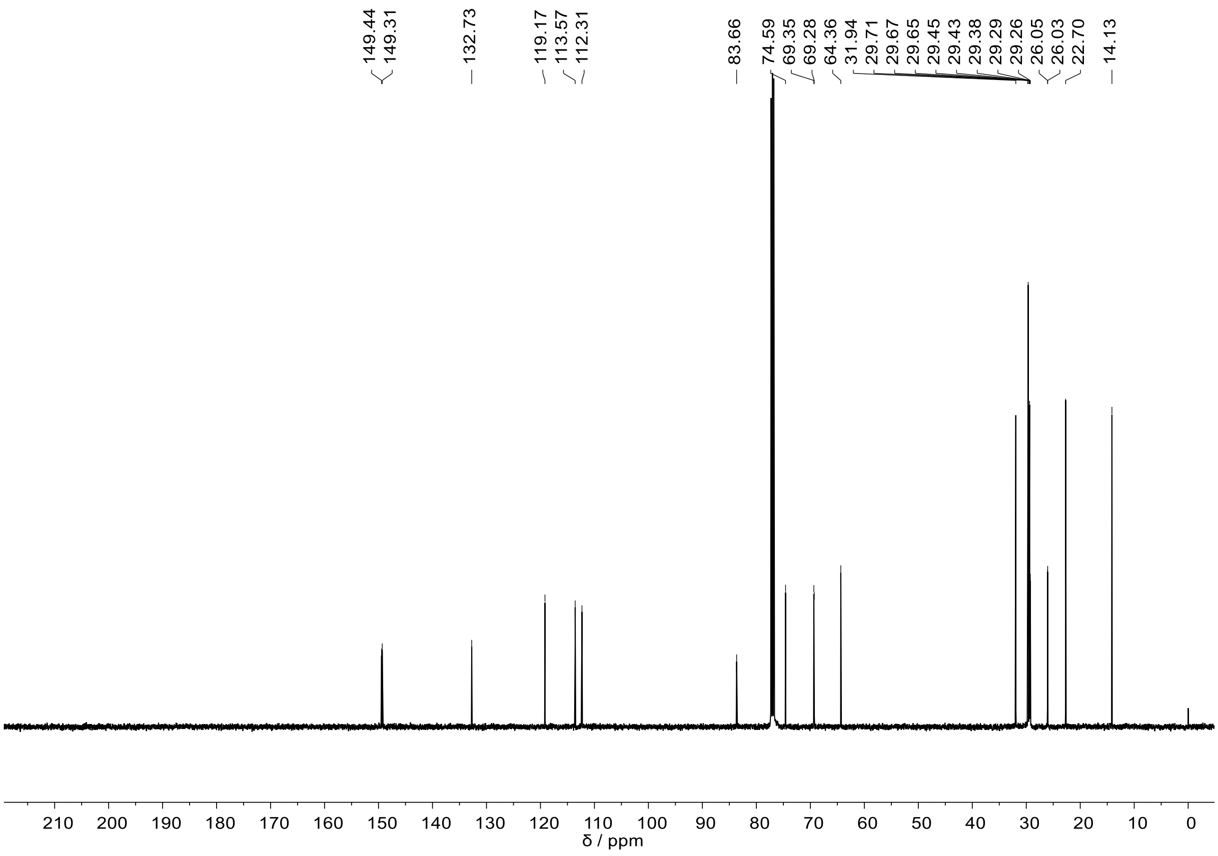

Figure S 41: ^{1}H (top, at 500 MHz) and ^{13}C NMR (bottom, at 126 MHz) of **8d**.

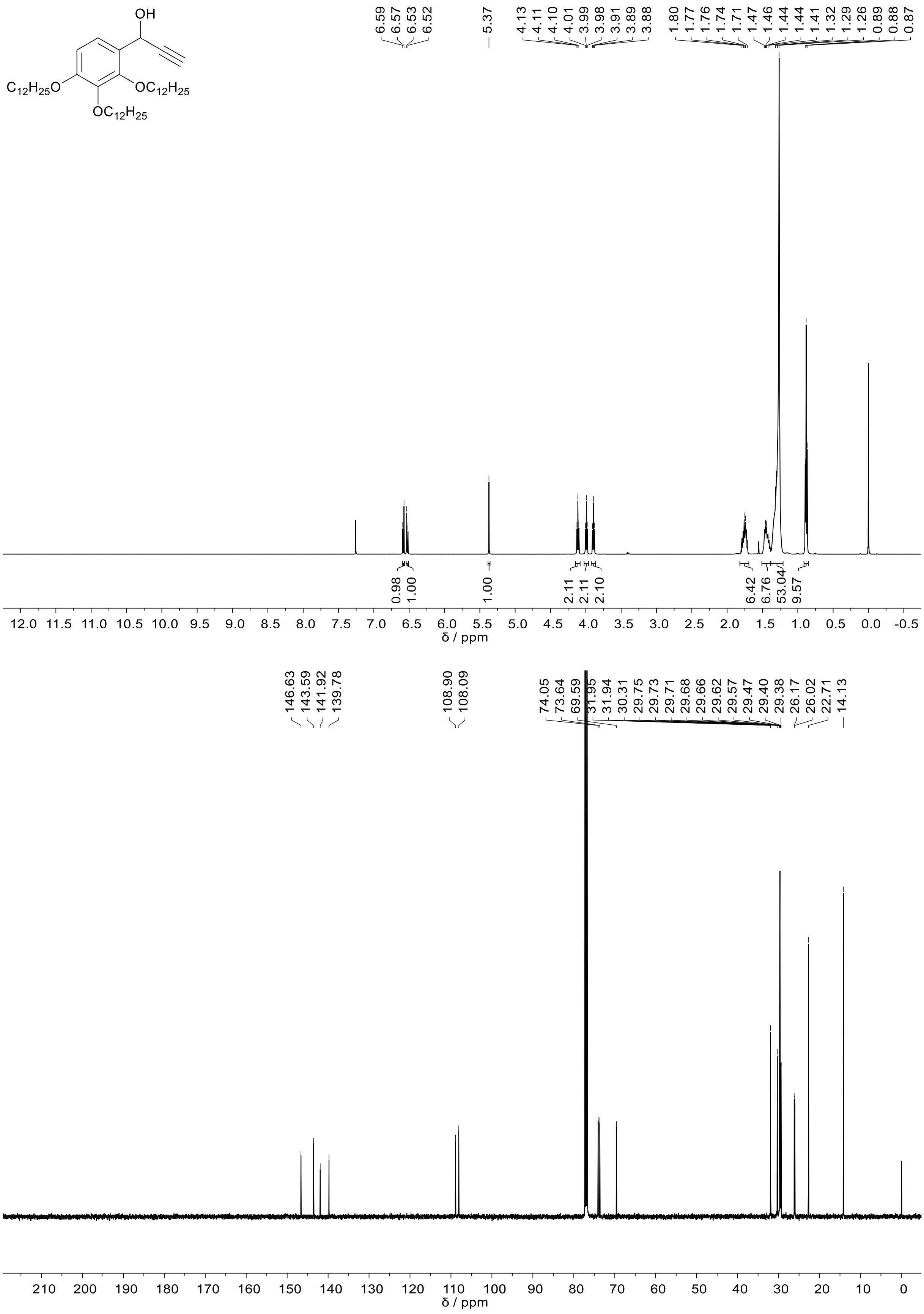

Figure S 42: ^{1}H (top, at 500 MHz) and ^{13}C NMR (bottom, at 126 MHz) of **8e**.

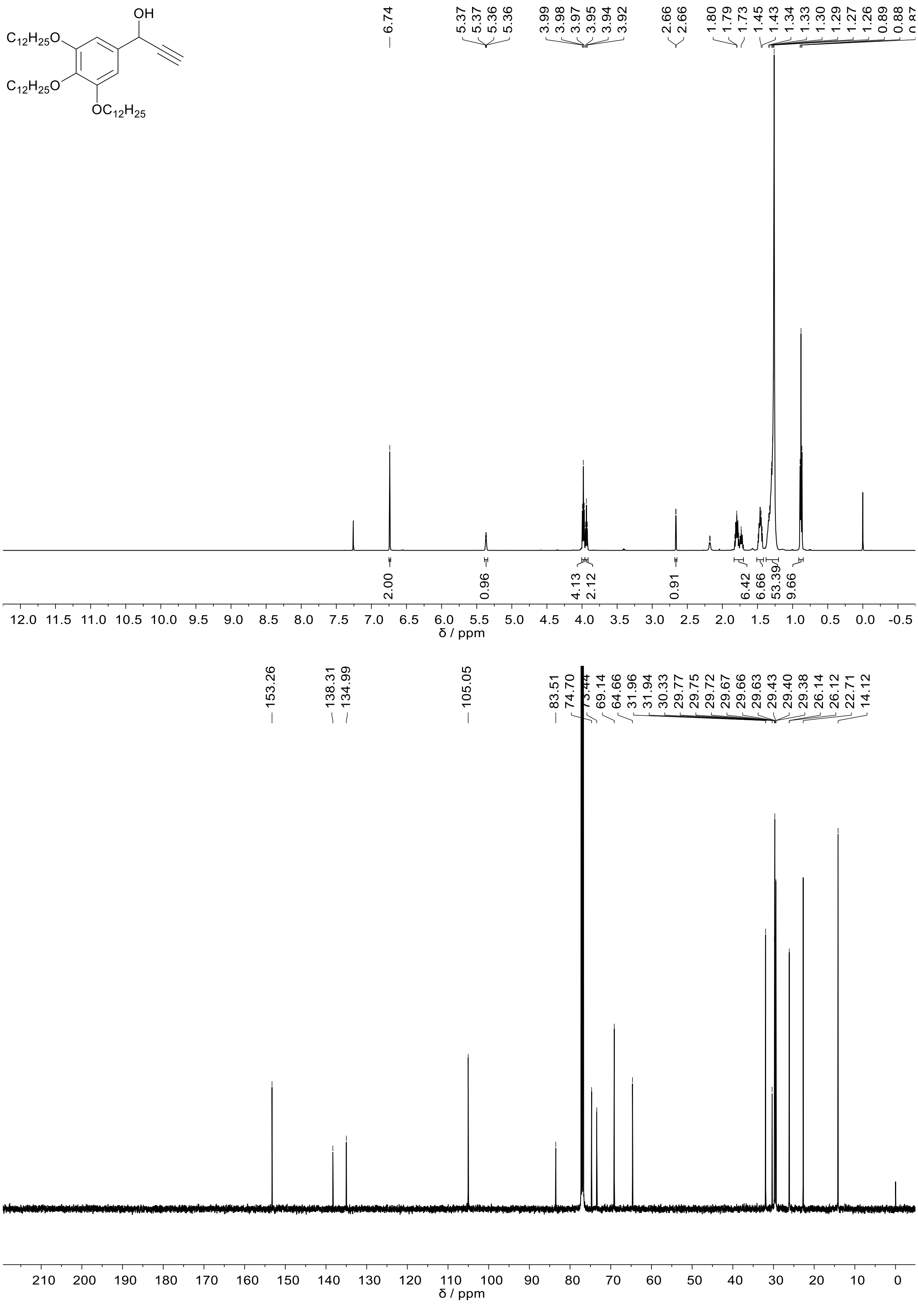

Figure S 43: ^{1}H (top, at 500 MHz) and ^{13}C NMR (bottom, at 126 MHz) of **8f**.

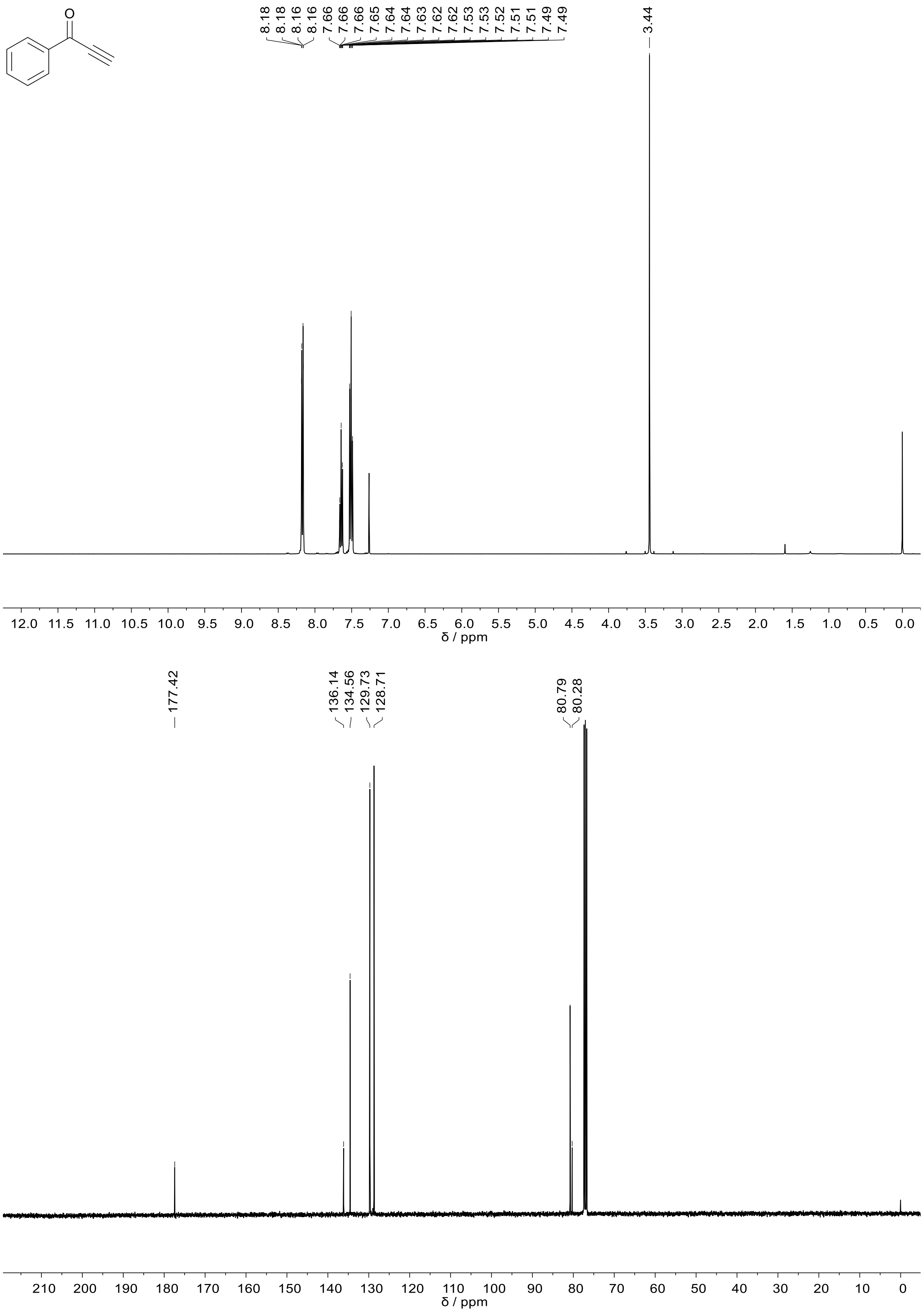

Figure S 44: ^{1}H (top, at 400 MHz) and ^{13}C NMR (bottom, at 101 MHz) of **XXx**.

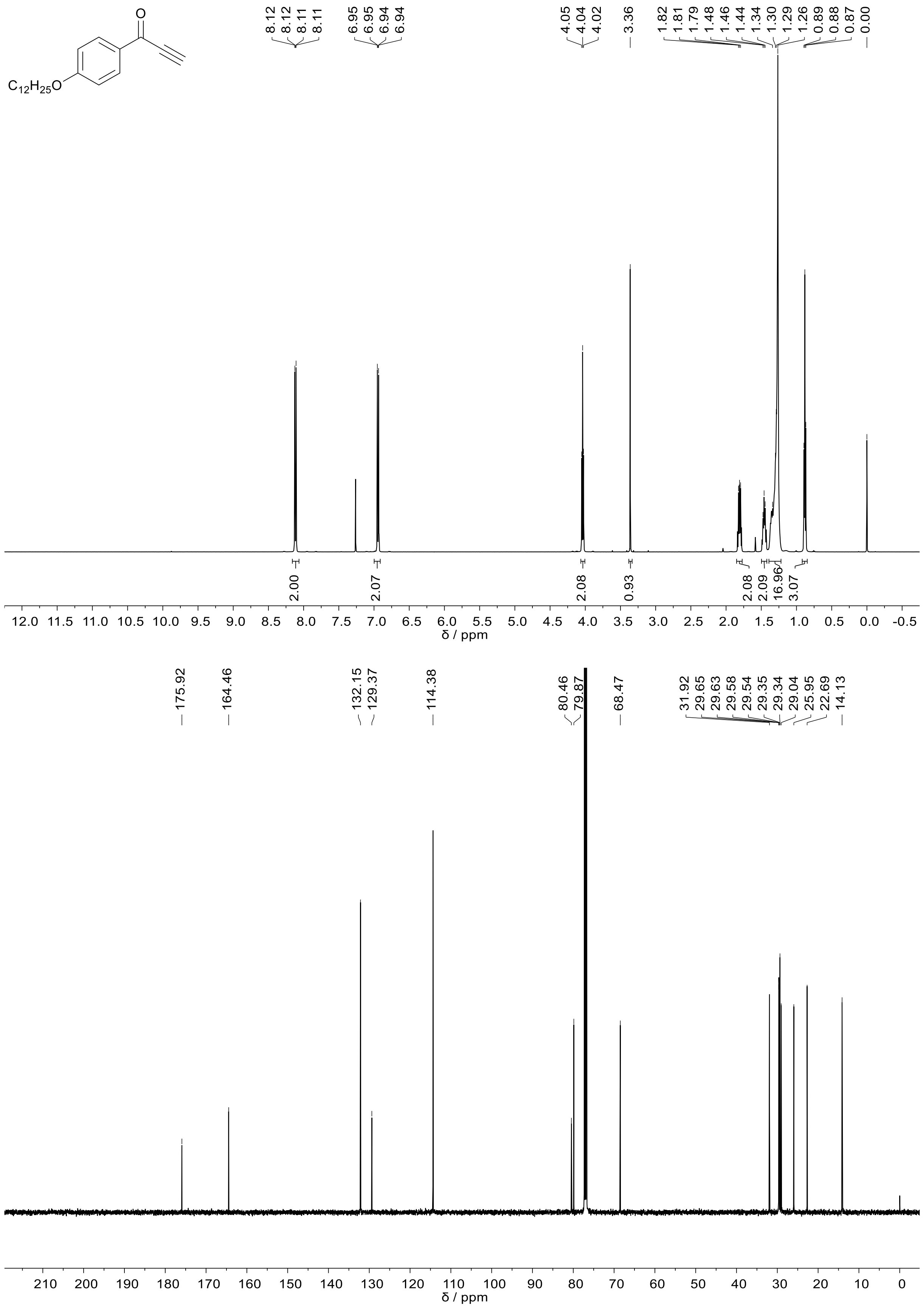

Figure S 45: ^{1}H (top, at 500 MHz) and ^{13}C NMR (bottom, at 126 MHz) of **9a**.

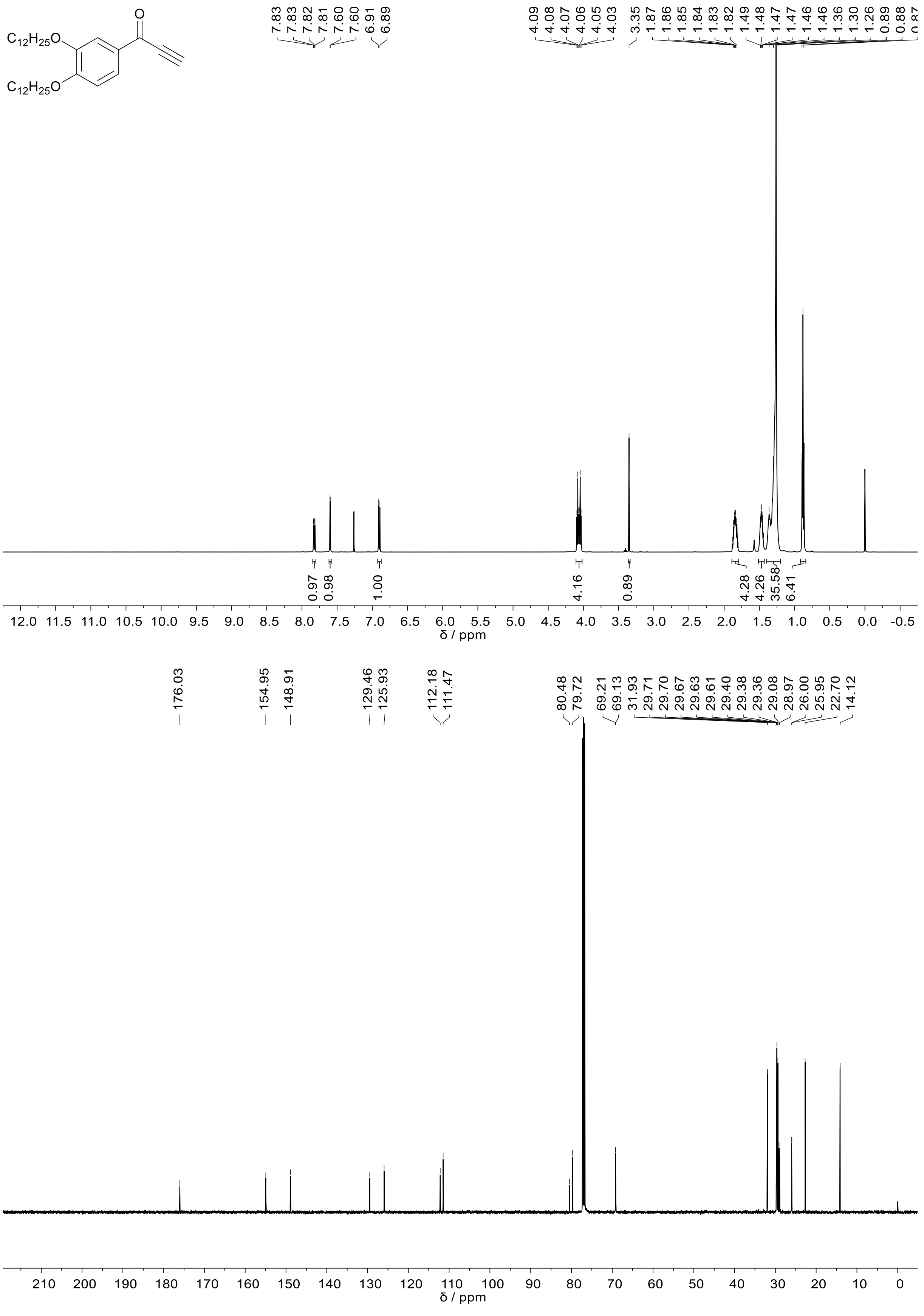

Figure S 46: ^{1}H (top, at 500 MHz) and ^{13}C NMR (bottom, at 126 MHz) of **9d**.

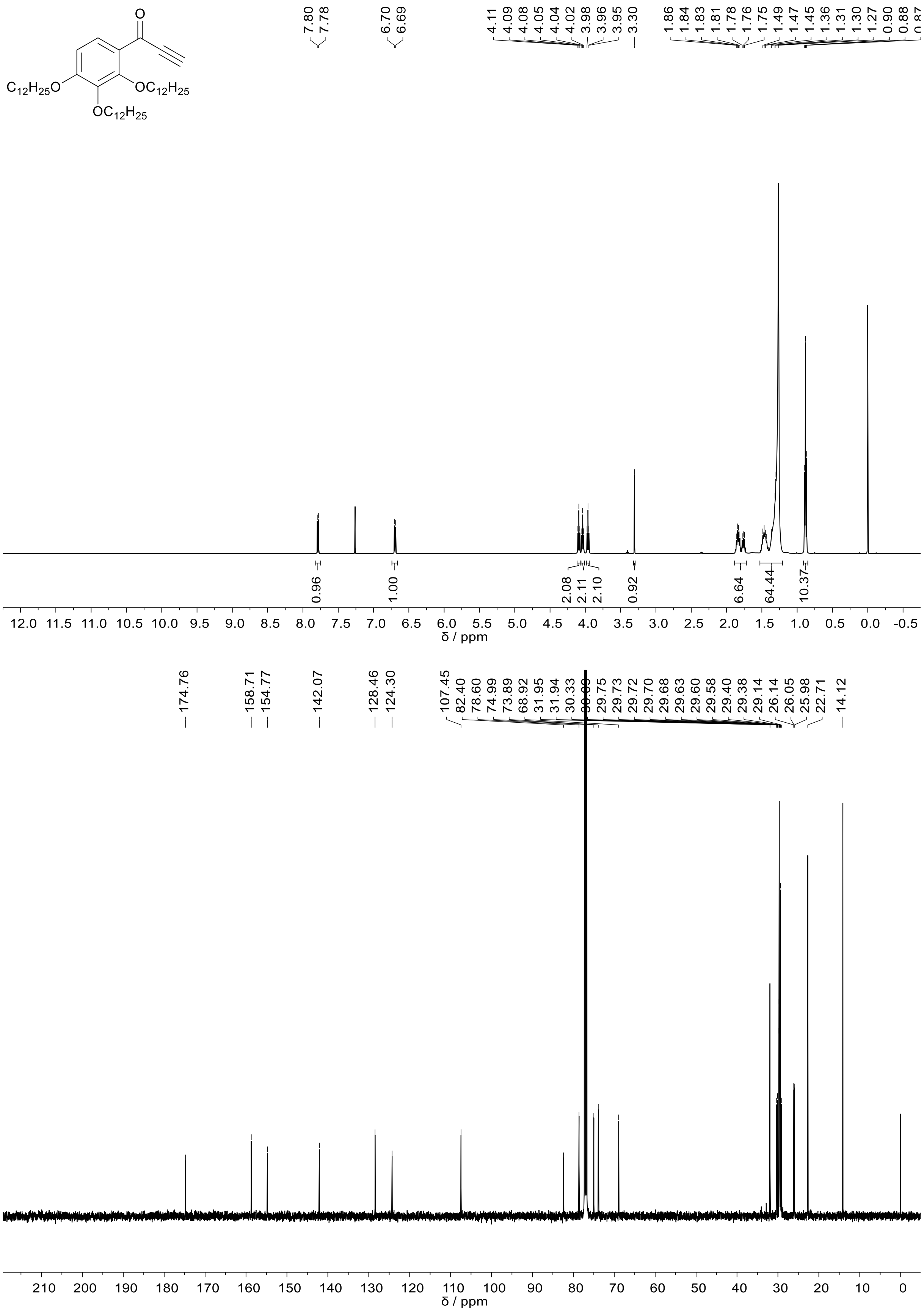

Figure S 47: ^{1}H (top, at 500 MHz) and ^{13}C NMR (bottom, at 126 MHz) of **9e**.

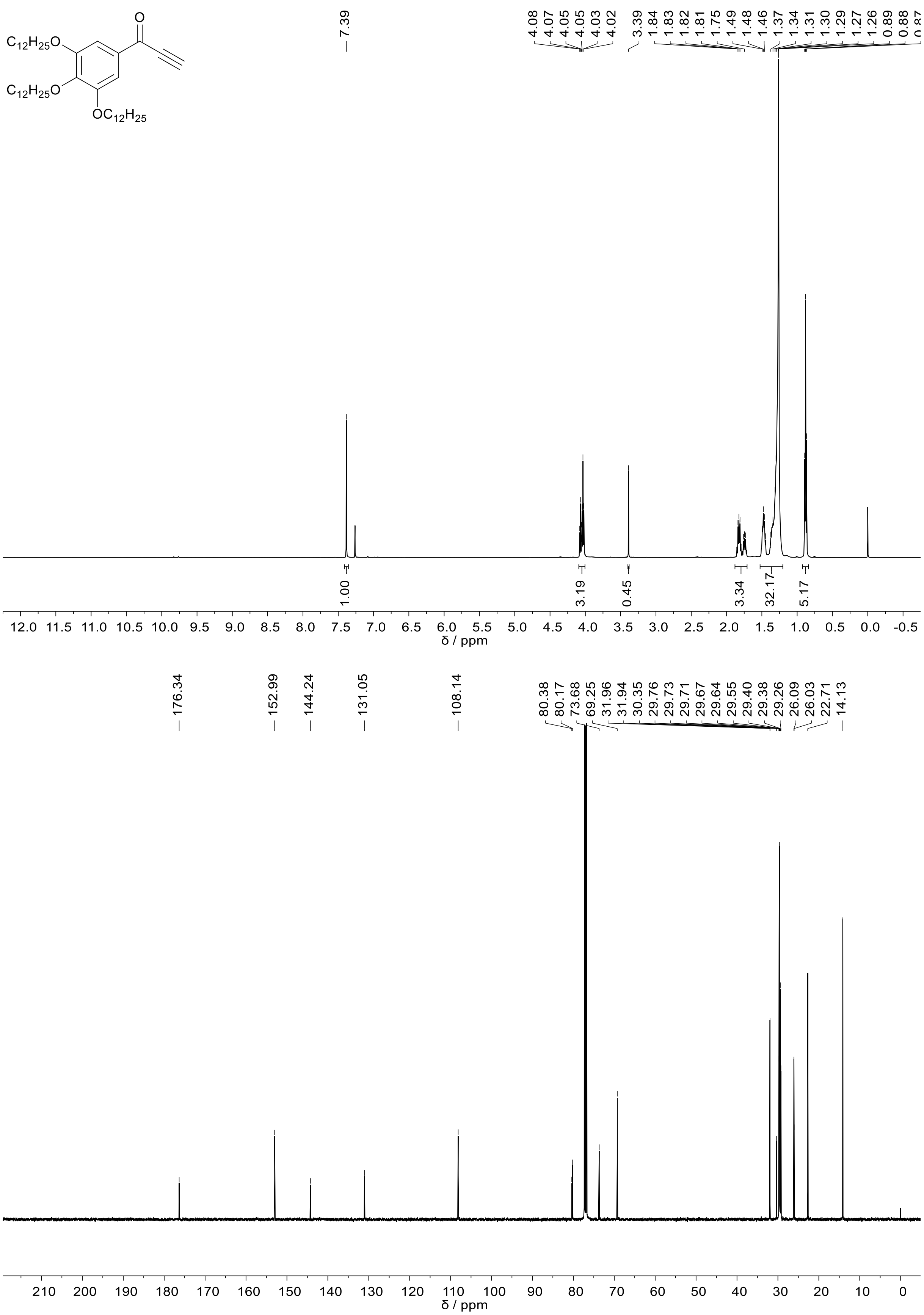

Figure S 48: ^{1}H (top, at 500 MHz) and ^{13}C NMR (bottom, at 126 MHz) of **9f**.

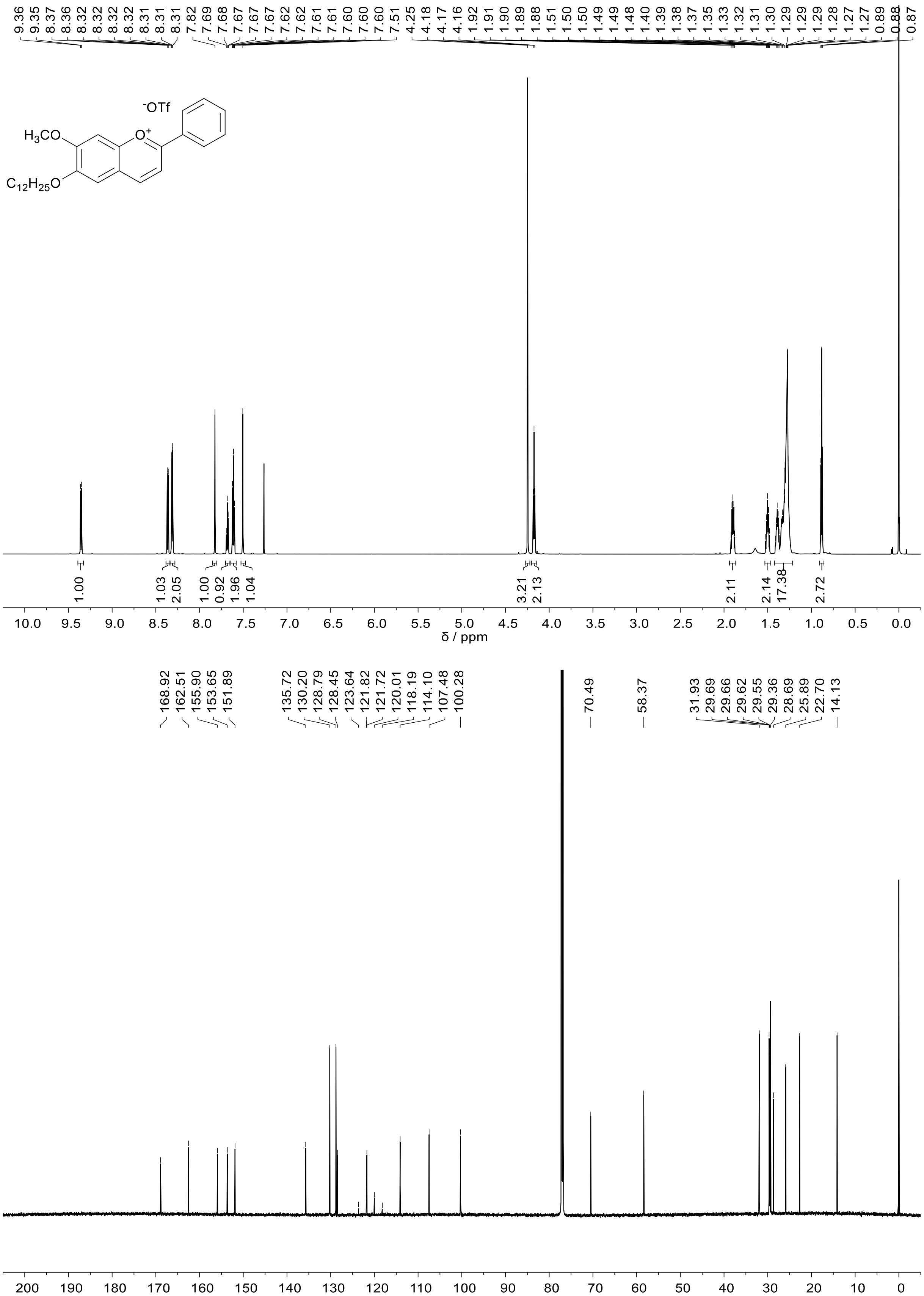

Figure S 49: ^{1}H (top, at 700 MHz) and ^{13}C NMR (bottom, at 176 MHz) of **V-Fla-0**.

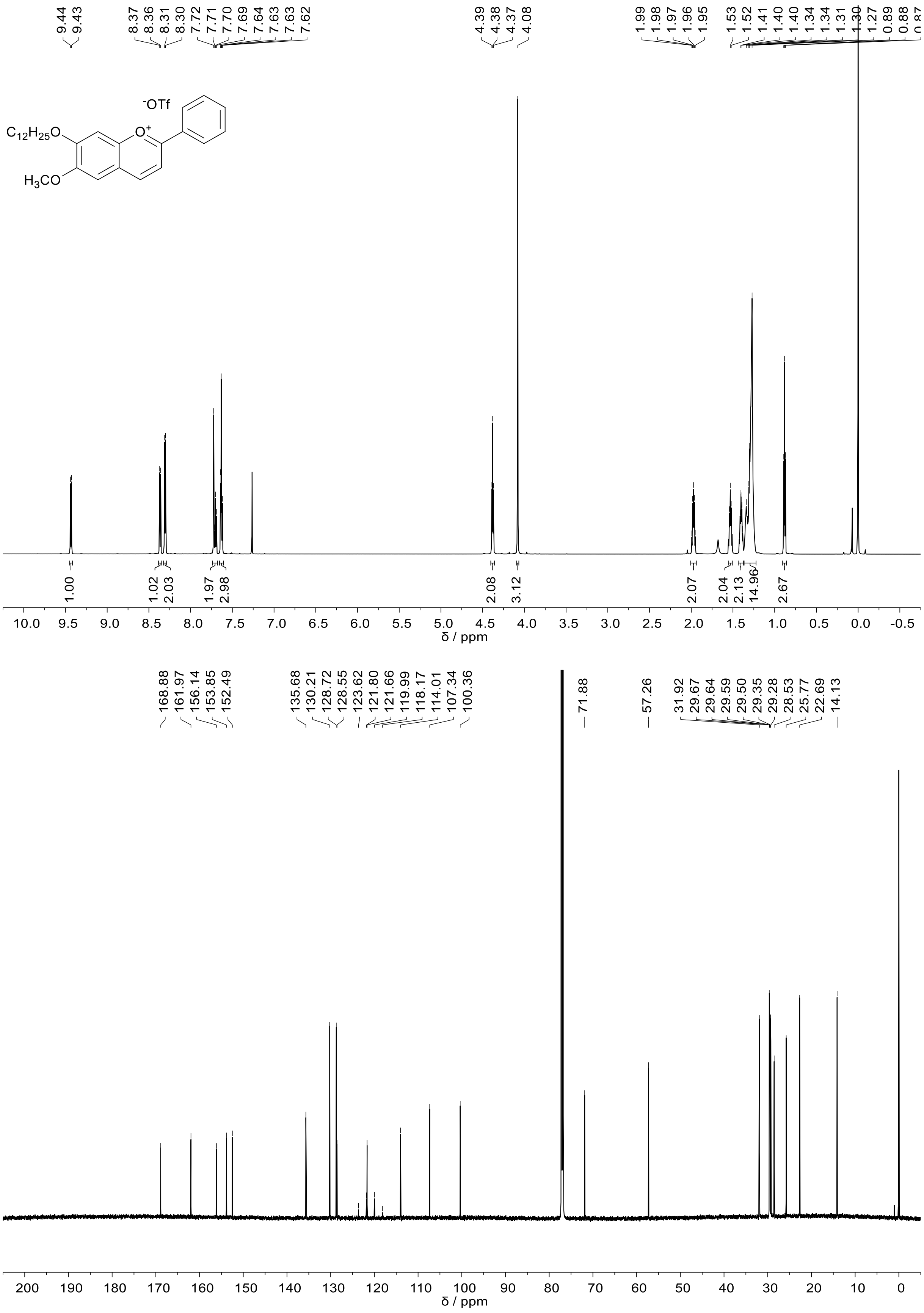

Figure S 50: ^{1}H (top, at 700 MHz) and ^{13}C NMR (bottom, at 176 MHz) of **iV-Fla-0**.

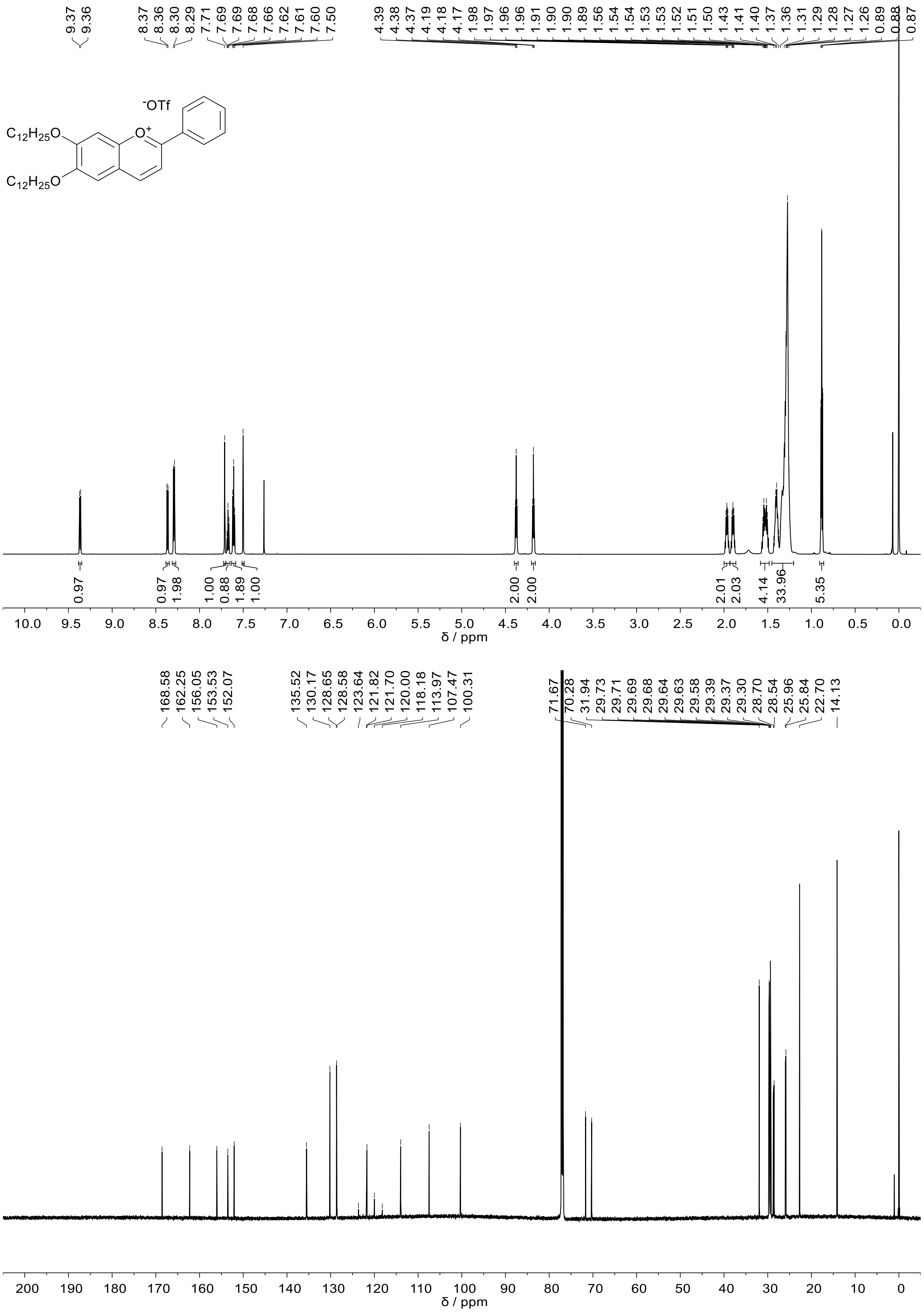

Figure S 51: ^{1}H (top, at 700 MHz) and ^{13}C NMR (bottom, at 176 MHz) of **2-Fla-0**.

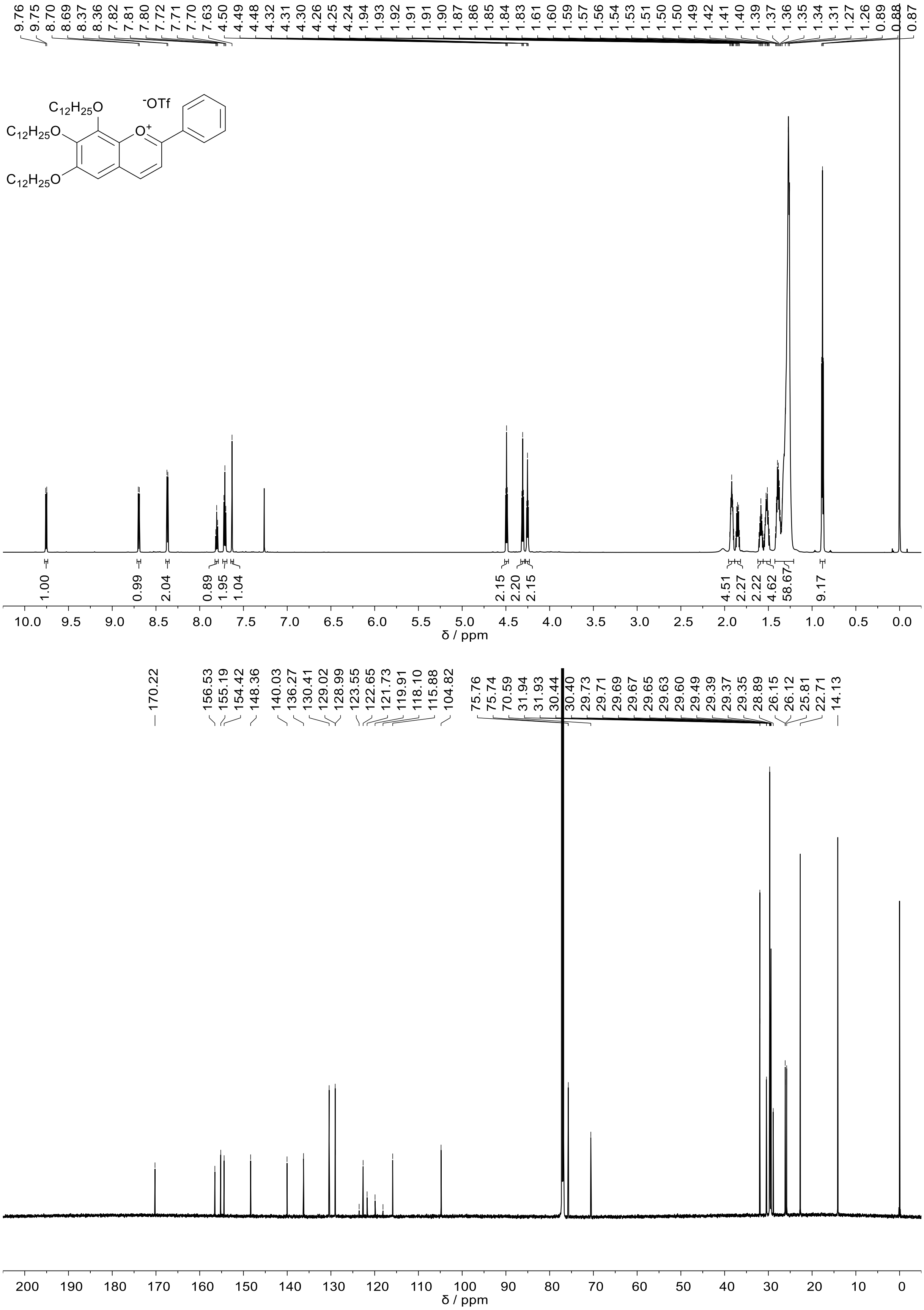

Figure S 52: ^{1}H (top, at 700 MHz) and ^{13}C NMR (bottom, at 176 MHz) of **3'-Fla-0**.

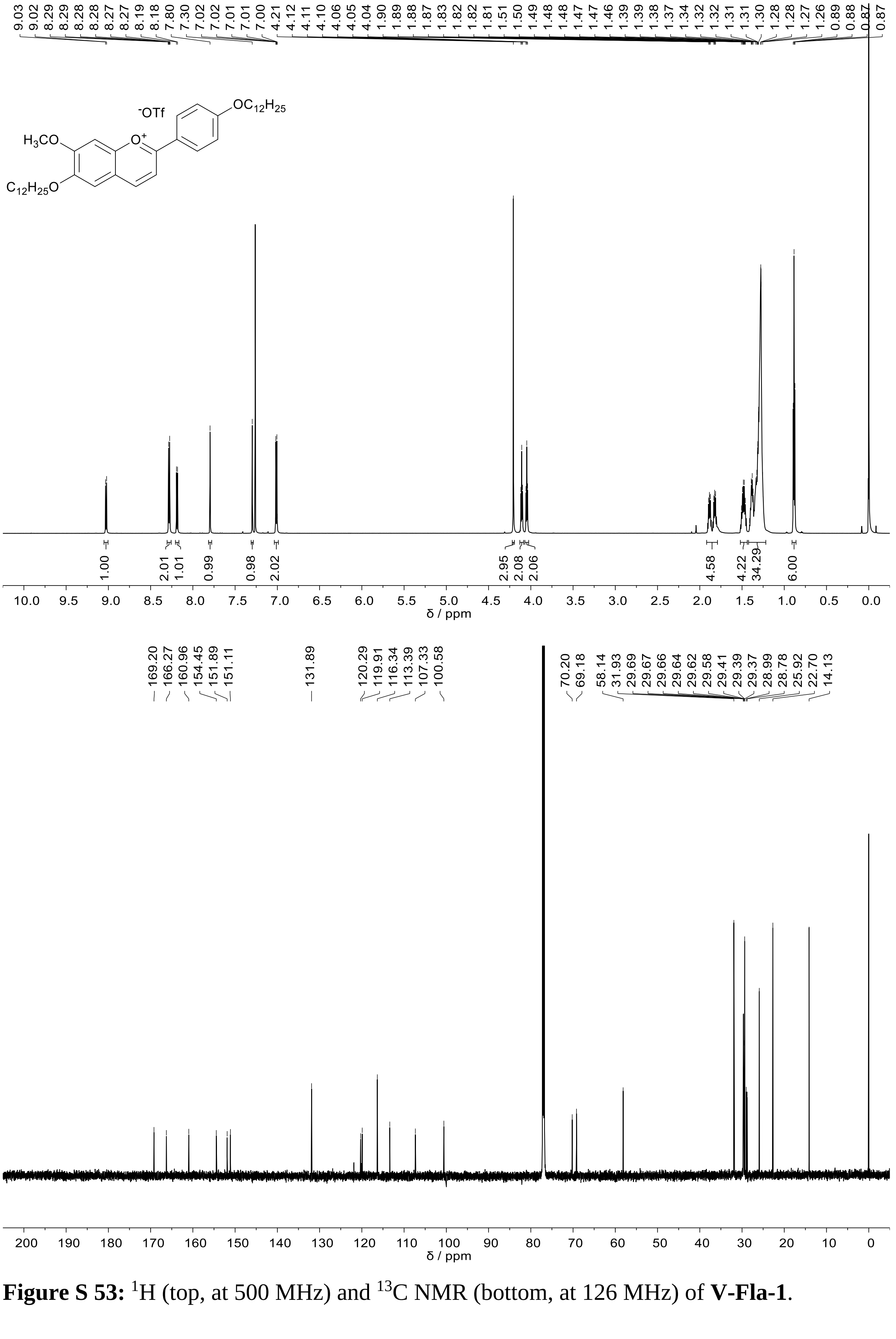

Figure S 53: ^{1}H (top, at 500 MHz) and ^{13}C NMR (bottom, at 126 MHz) of **V-Fla-1**.

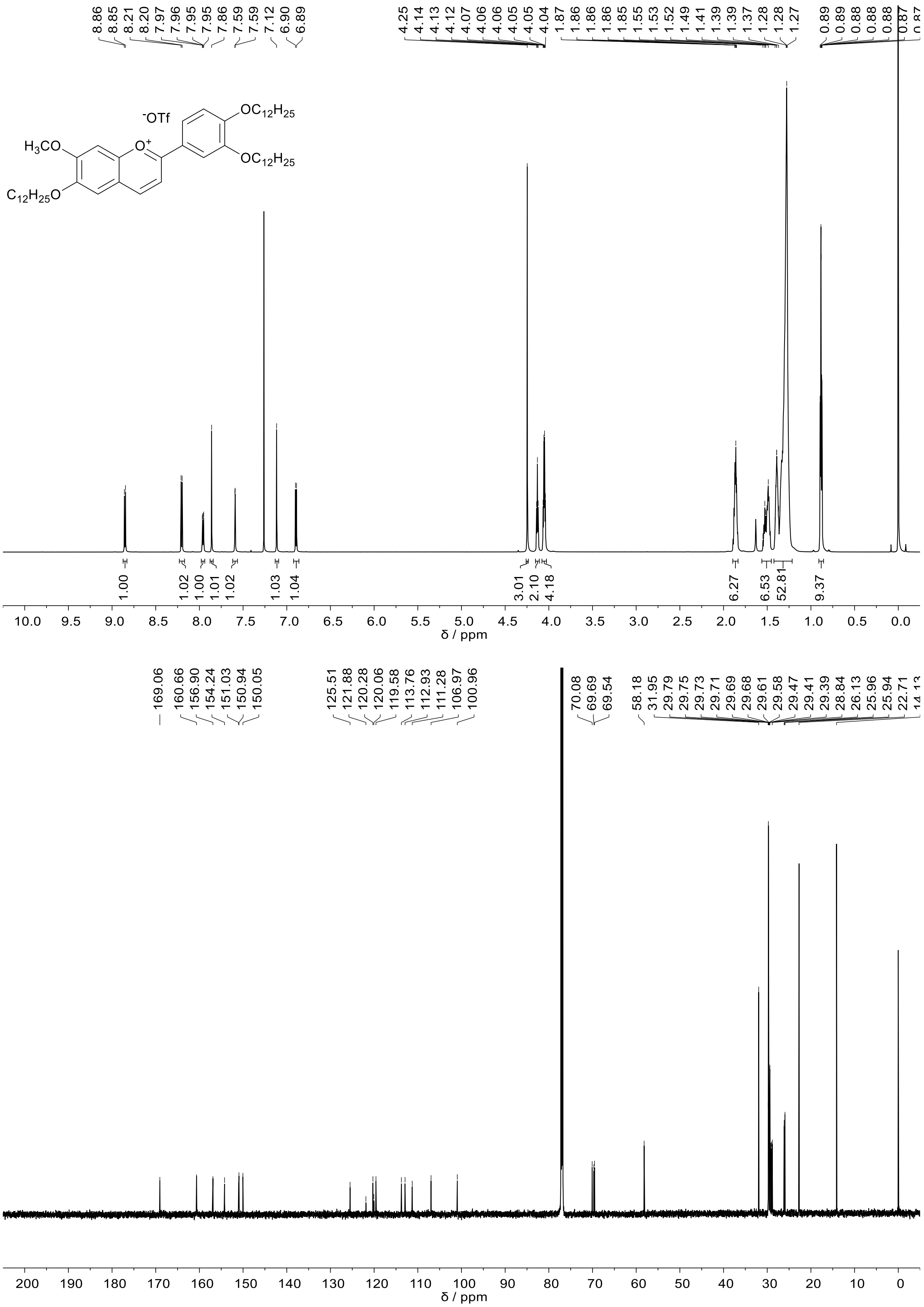

Figure S 54: ^{1}H (top, at 500 MHz) and ^{13}C NMR (bottom, at 126 MHz) of **V-Fla-2**.

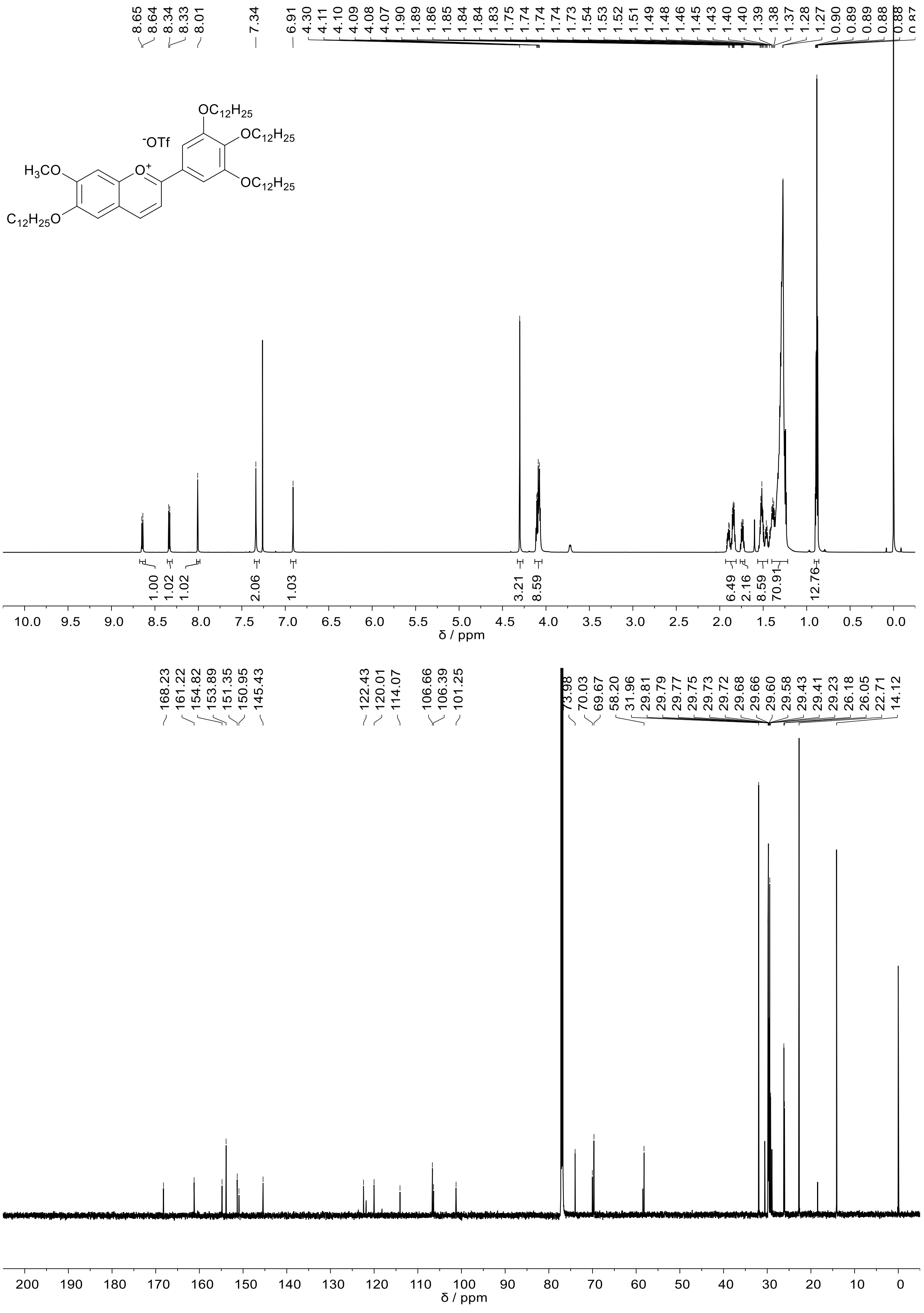

Figure S 55: ^{1}H (top, at 500 MHz) and ^{13}C NMR (bottom, at 126 MHz) of **V-Fla-3**.

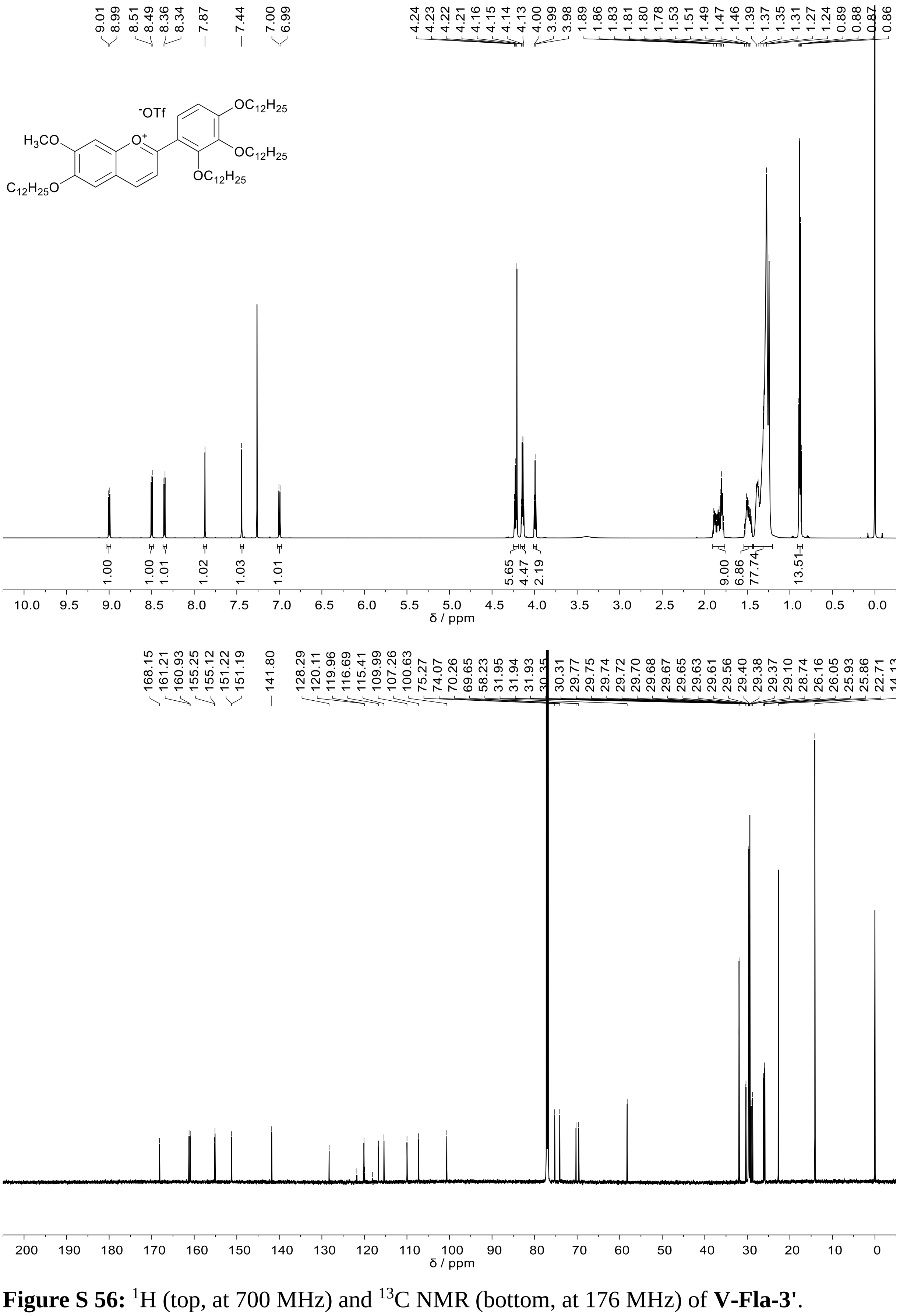

Figure S 56: ^{1}H (top, at 700 MHz) and ^{13}C NMR (bottom, at 176 MHz) of **V-Fla-3'**.

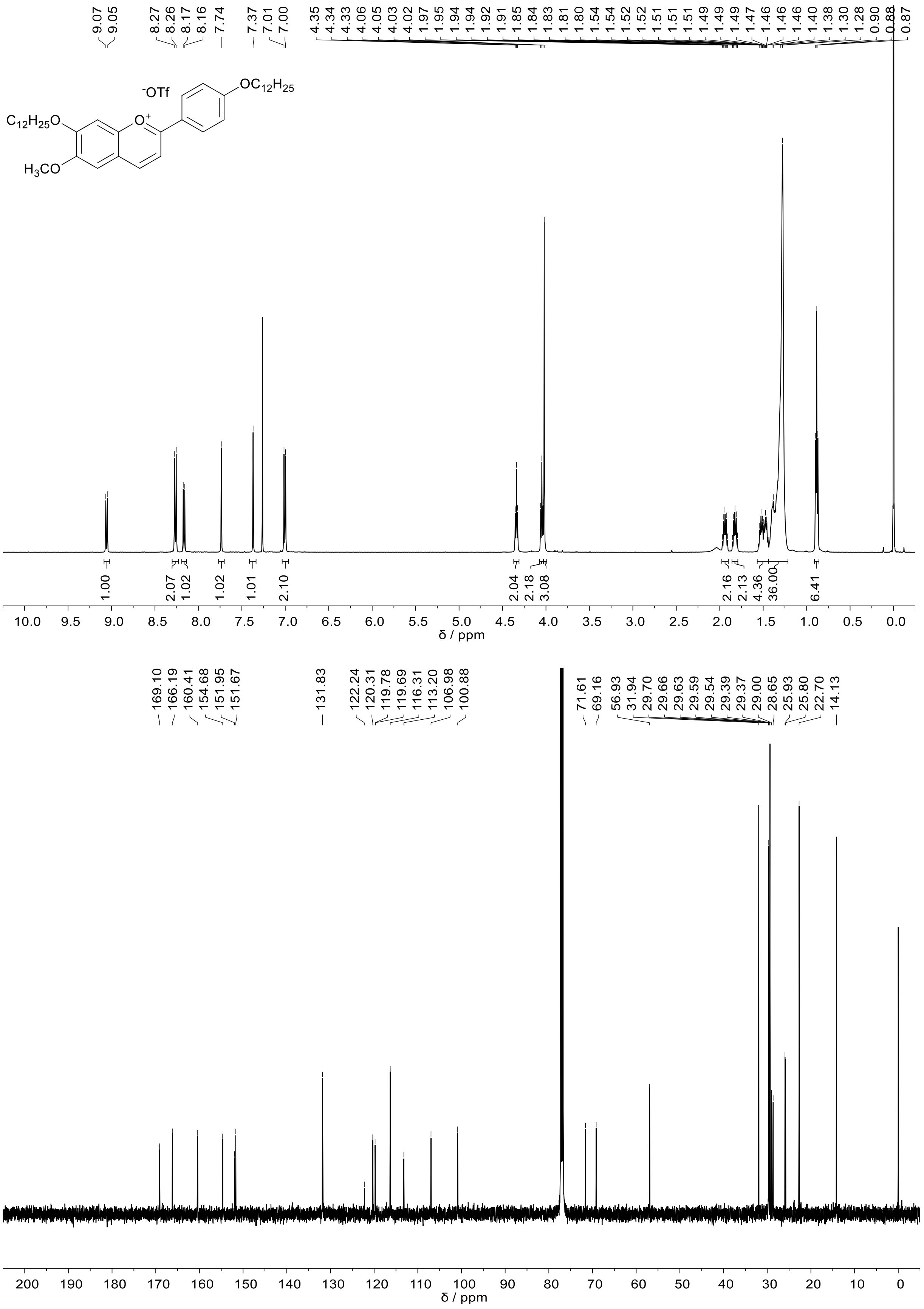

Figure S 57: ^{1}H (top, at 500 MHz) and ^{13}C NMR (bottom, at 126 MHz) of **iV-Fla-1**.

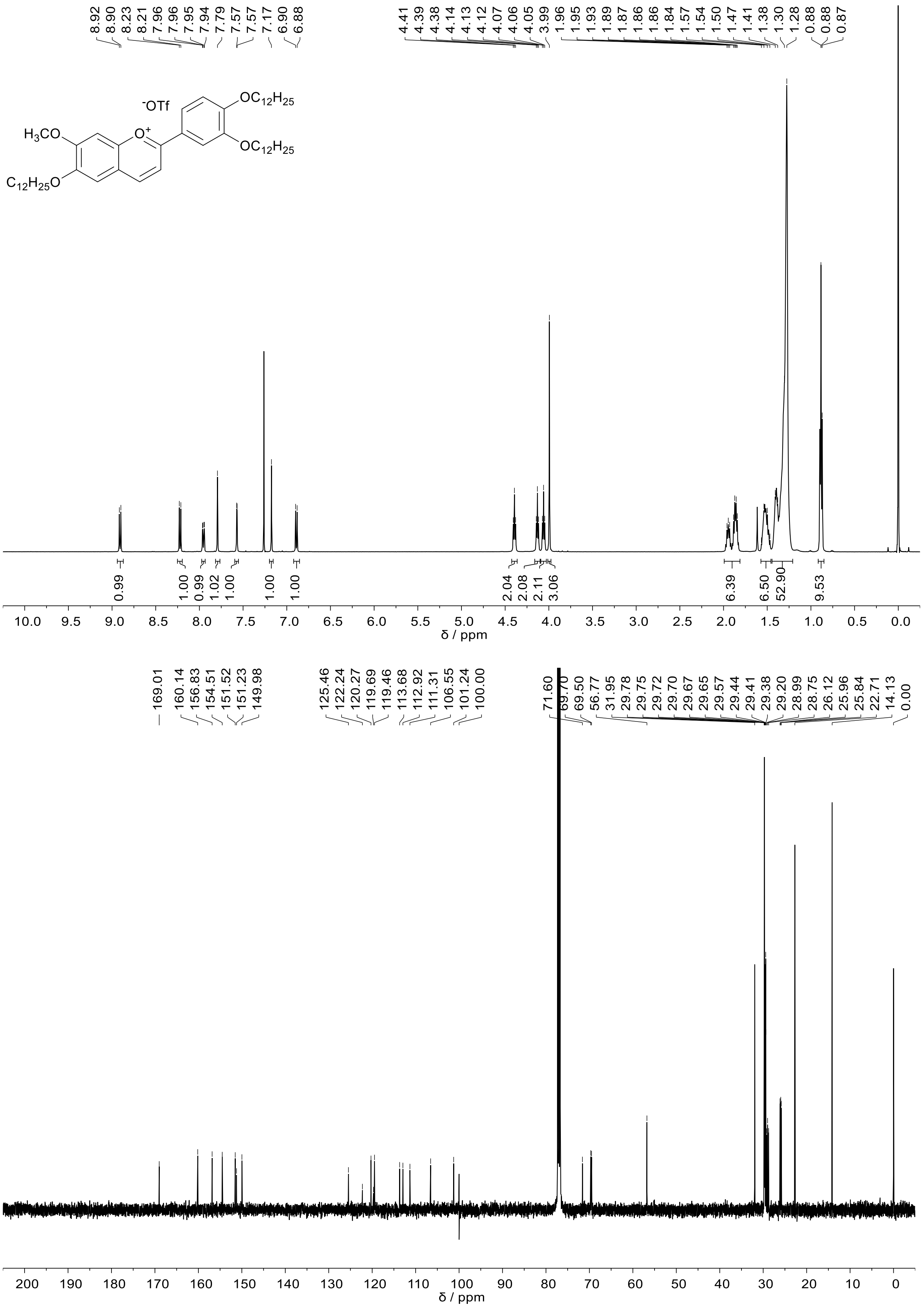

Figure S 58: ^{1}H (top, at 500 MHz) and ^{13}C NMR (bottom, at 126 MHz) of **iV-Fla-2**.

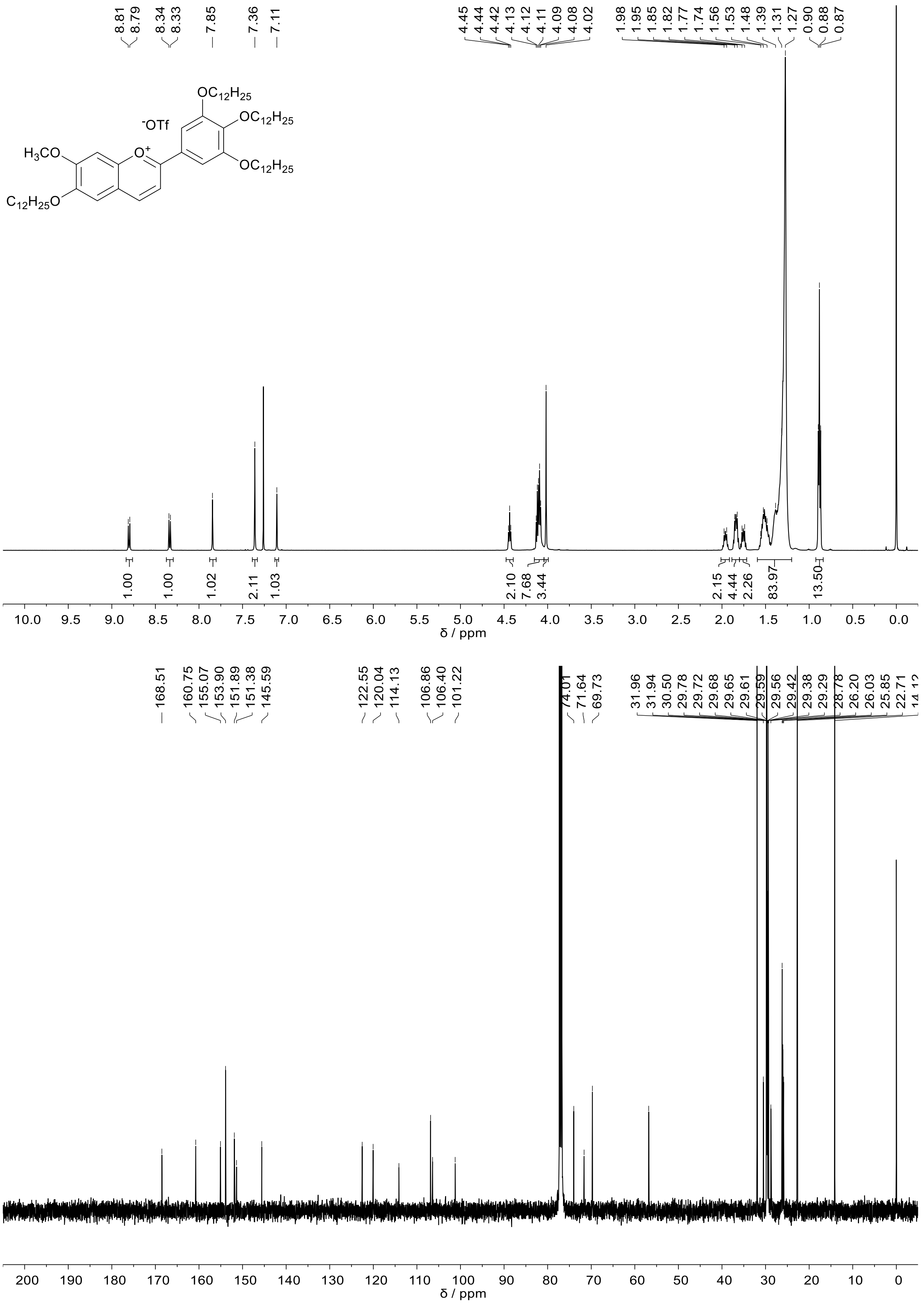

Figure S 59: ^{1}H (top, at 500 MHz) and ^{13}C NMR (bottom, at 126 MHz) of **iV-Fla-3**.

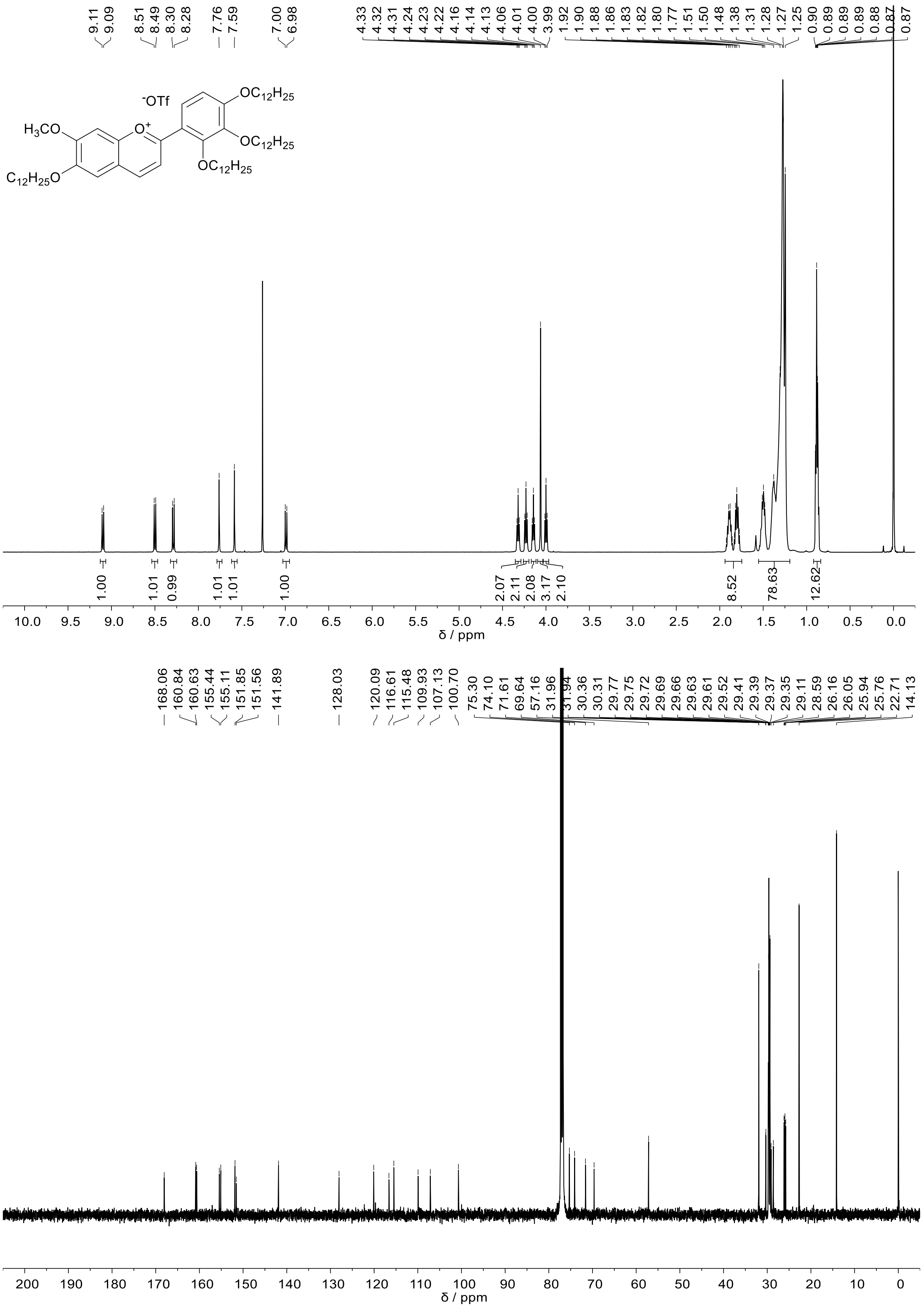

Figure S 60: ^{1}H (top, at 500 MHz) and ^{13}C NMR (bottom, at 126 MHz) of **iV-Fla-3'**.

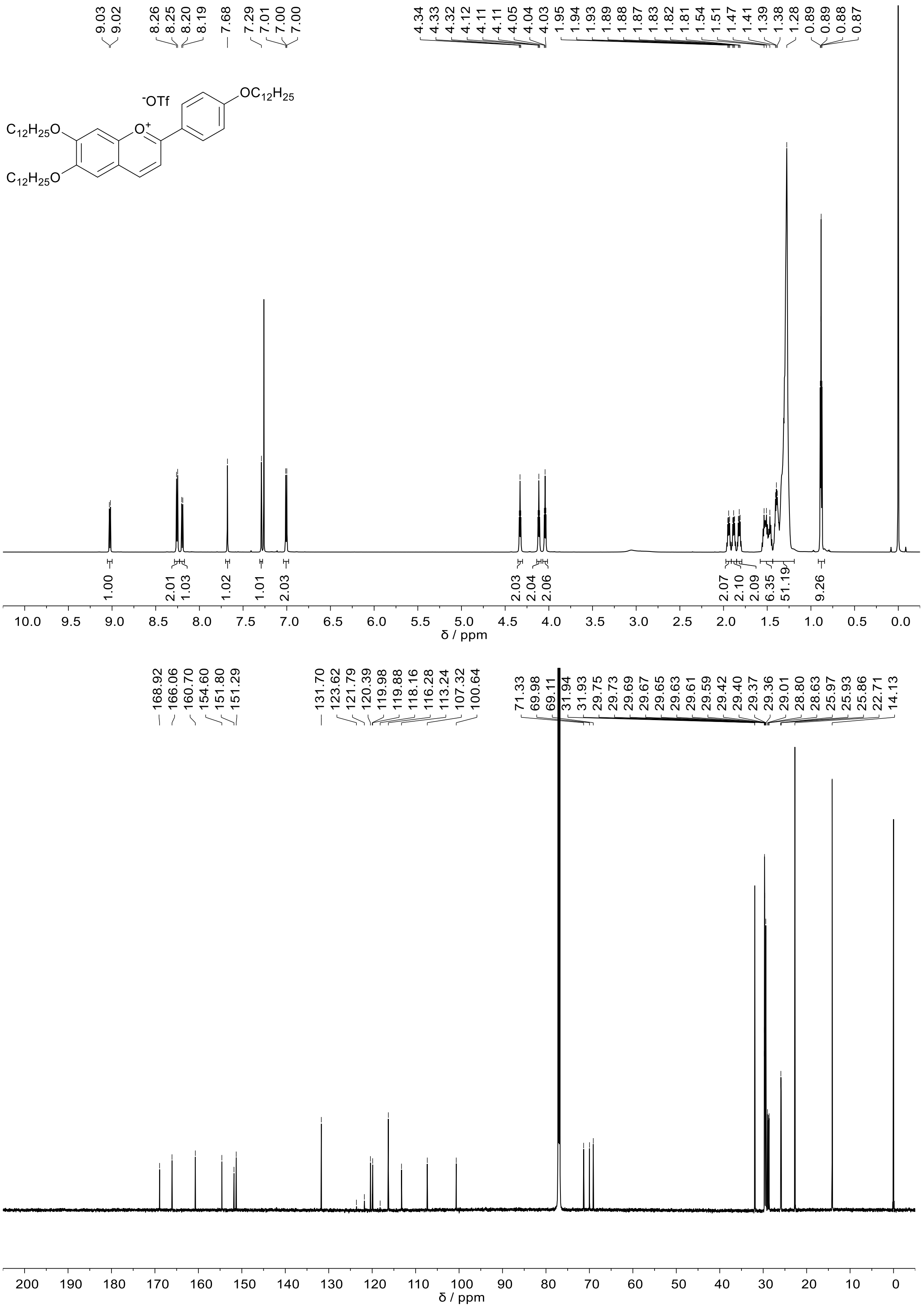

Figure S 61: ^{1}H (top, at 700 MHz) and ^{13}C NMR (bottom, at 176 MHz) of **2-Fla-1**.

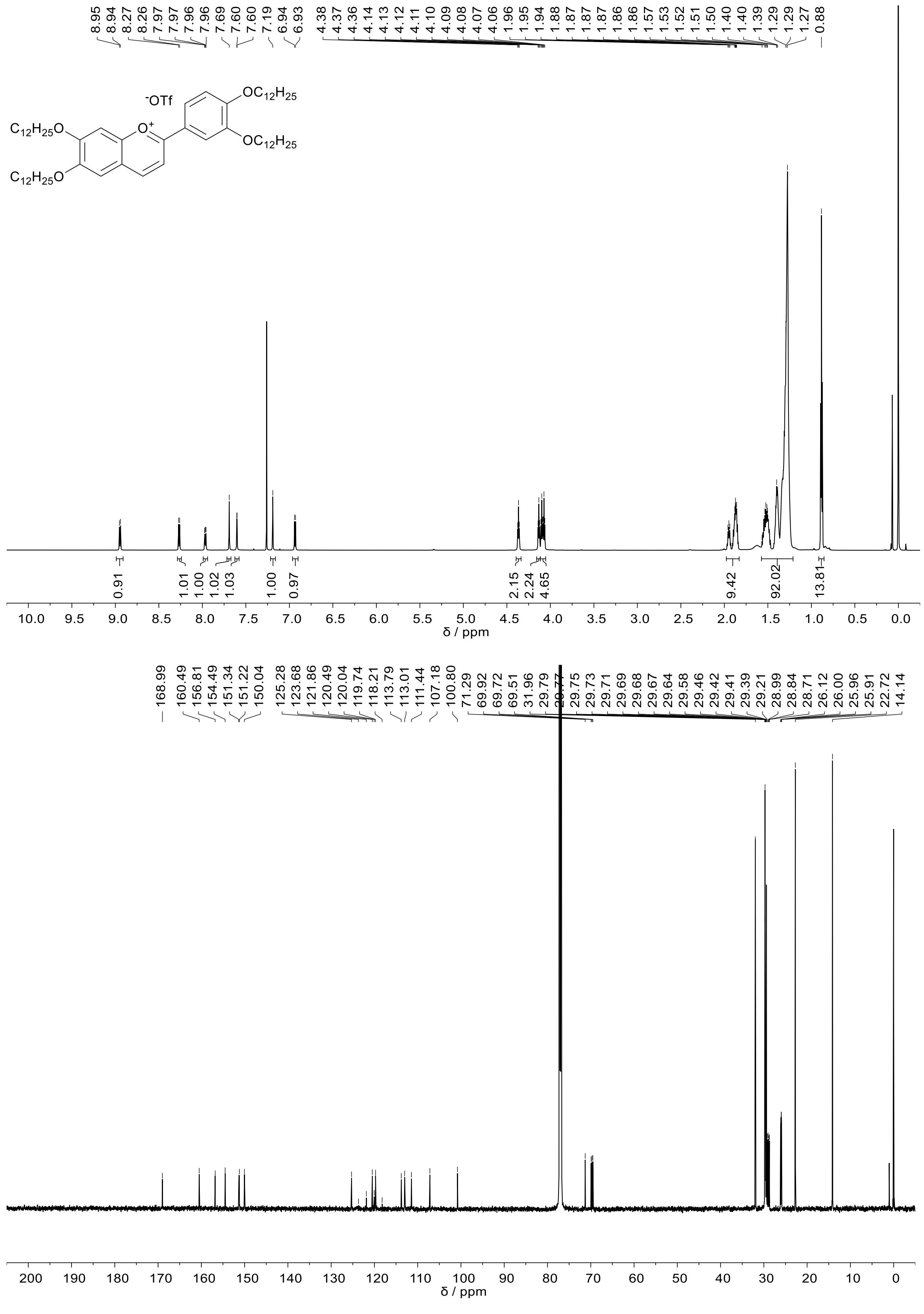

Figure S 62: ^{1}H (top, at 700 MHz) and ^{13}C NMR (bottom, at 176 MHz) of **2-Fla-2**.

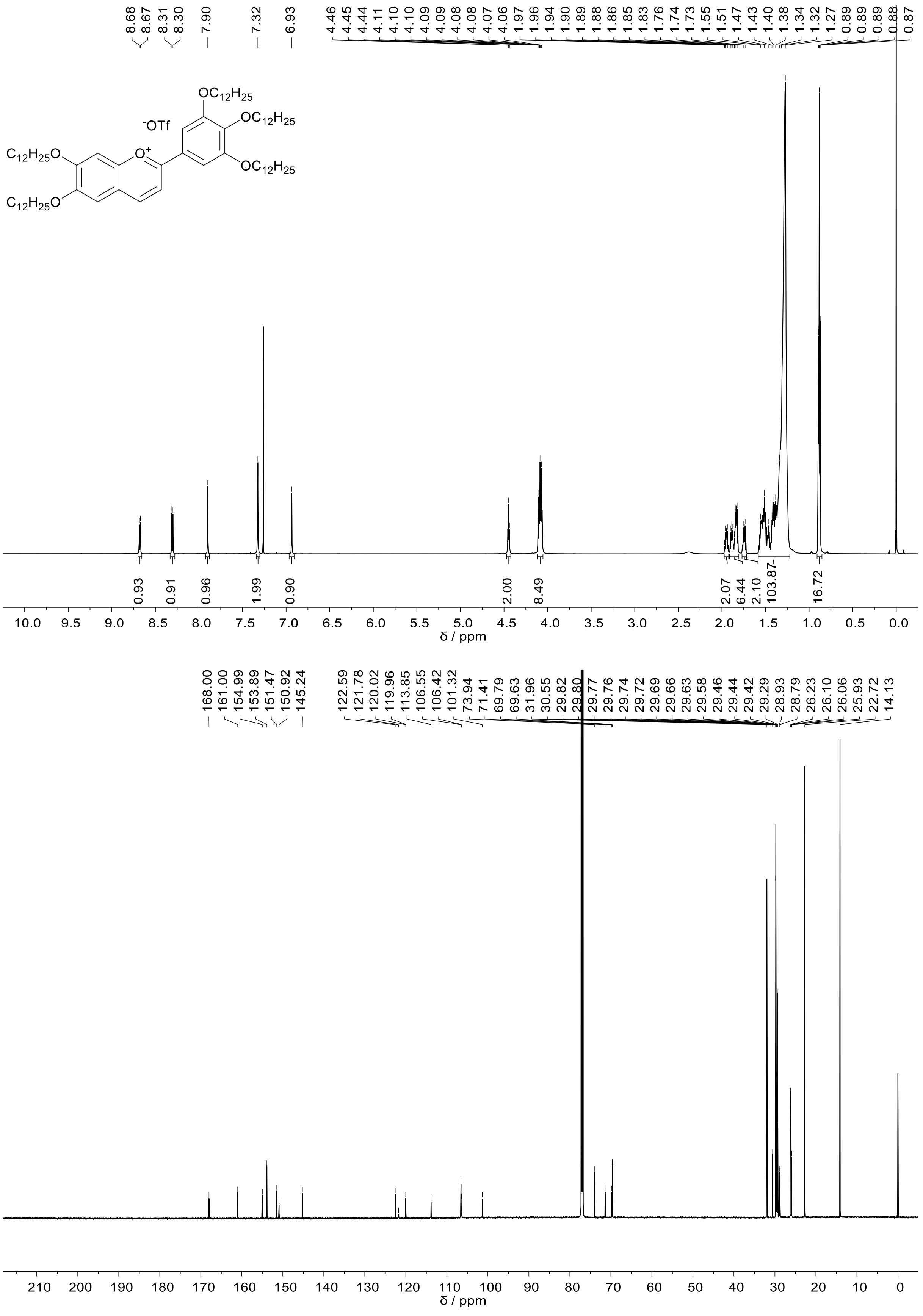

Figure S 63: ^{1}H (top, at 700 MHz) and ^{13}C NMR (bottom, at 176 MHz) of **2-Fla-3**.

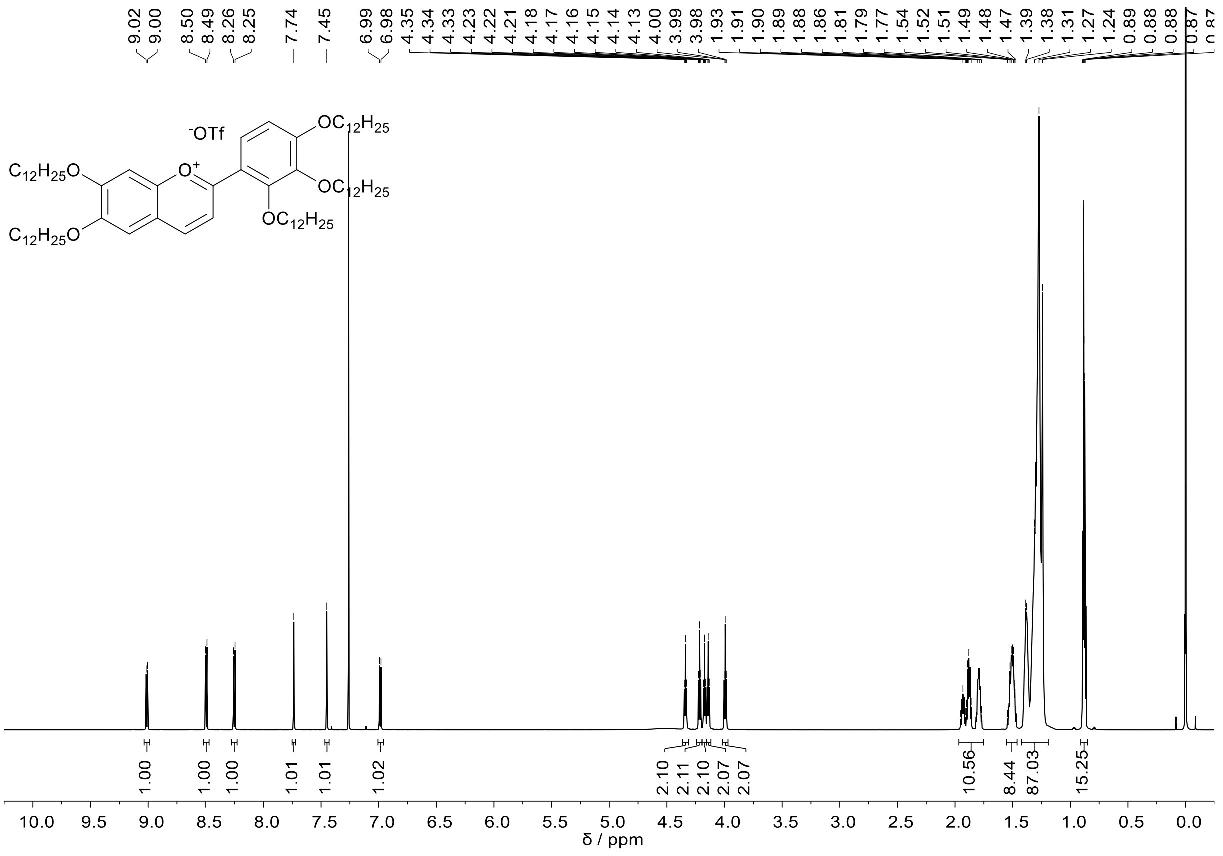

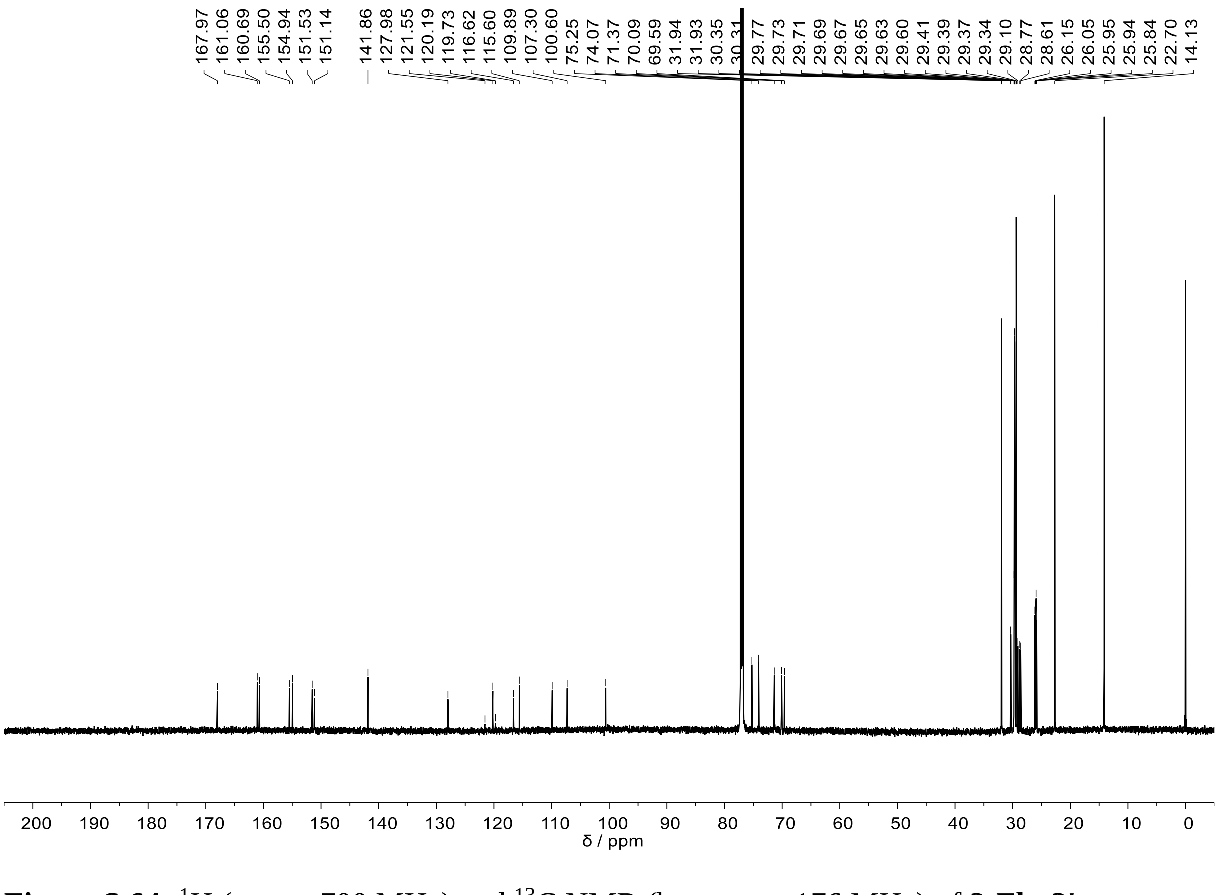

Figure S 64: ^{1}H (top, at 700 MHz) and ^{13}C NMR (bottom, at 176 MHz) of **2-Fla-3'**.

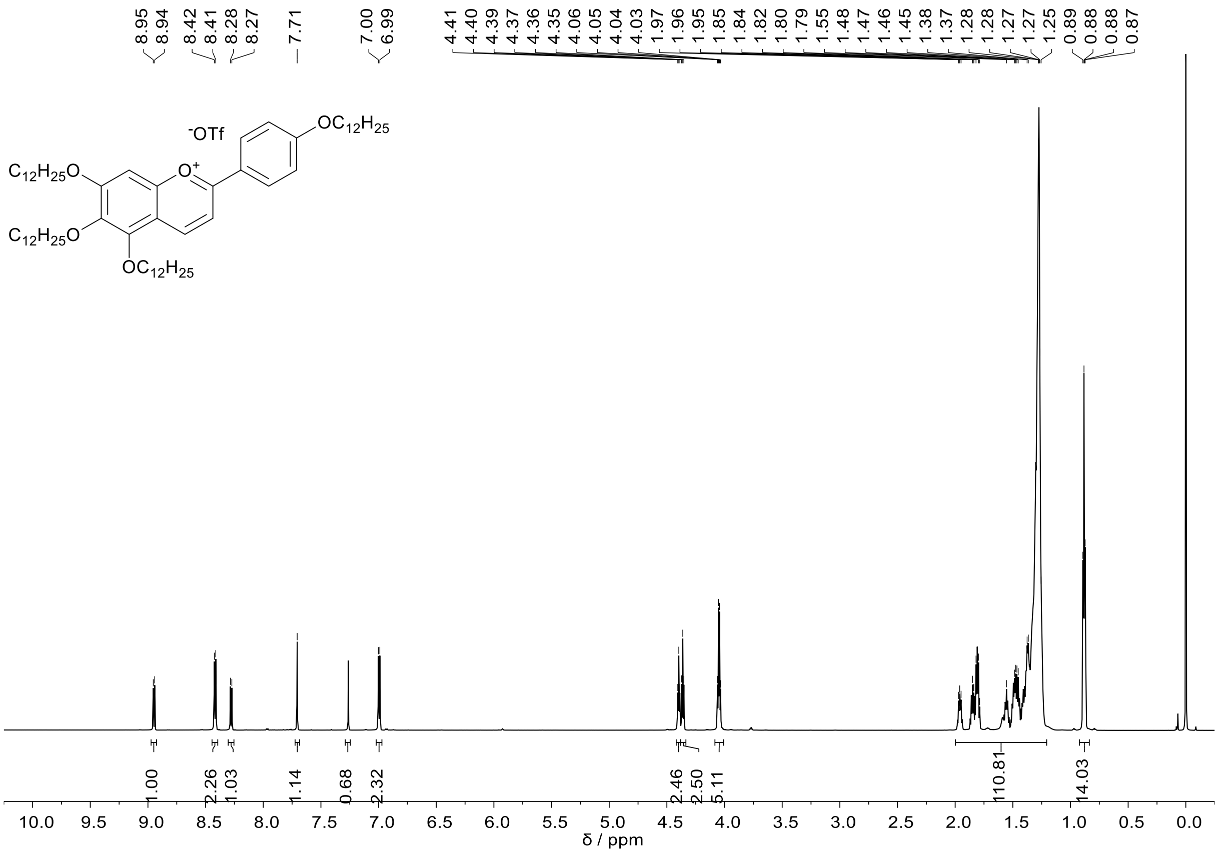

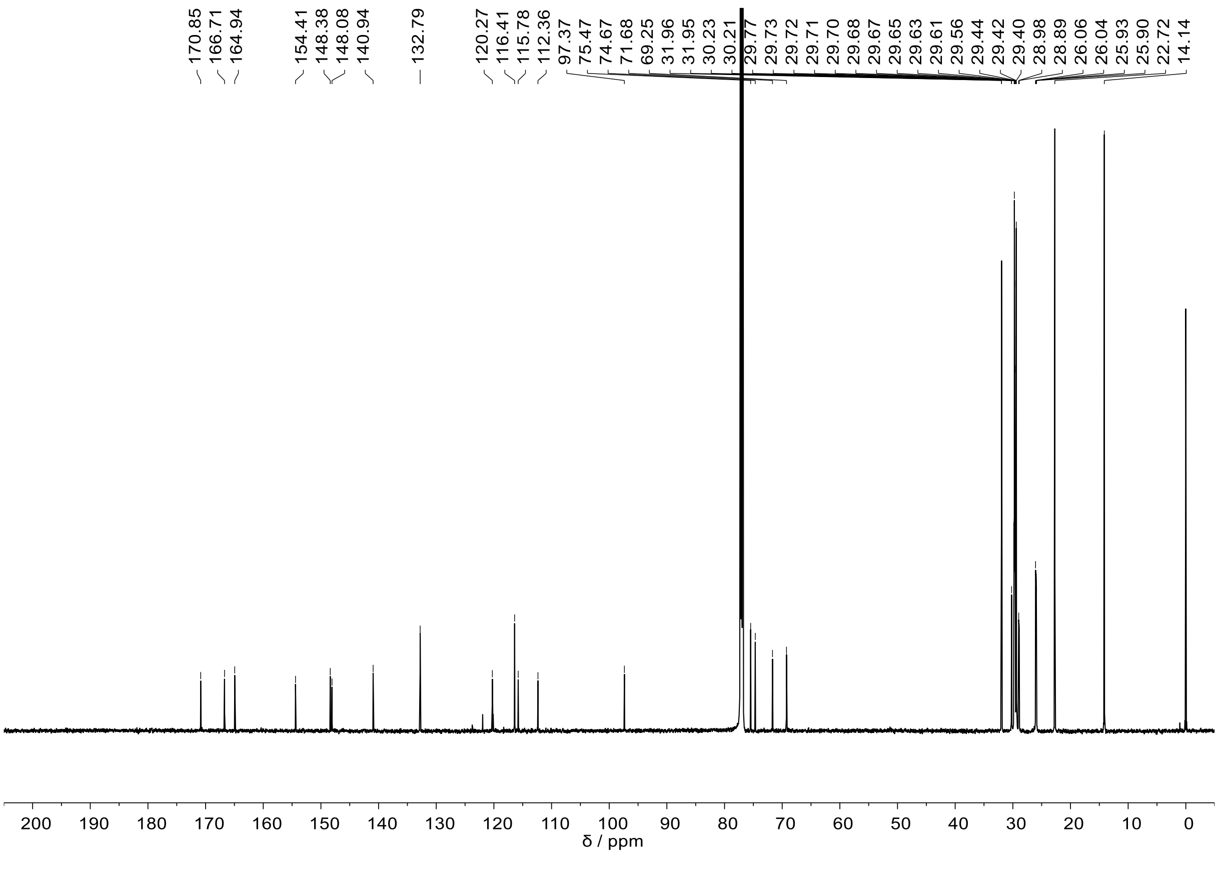

Figure S 65: ^{1}H (top, at 700 MHz) and ^{13}C NMR (bottom, at 176 MHz) of **3-Fla-1**.

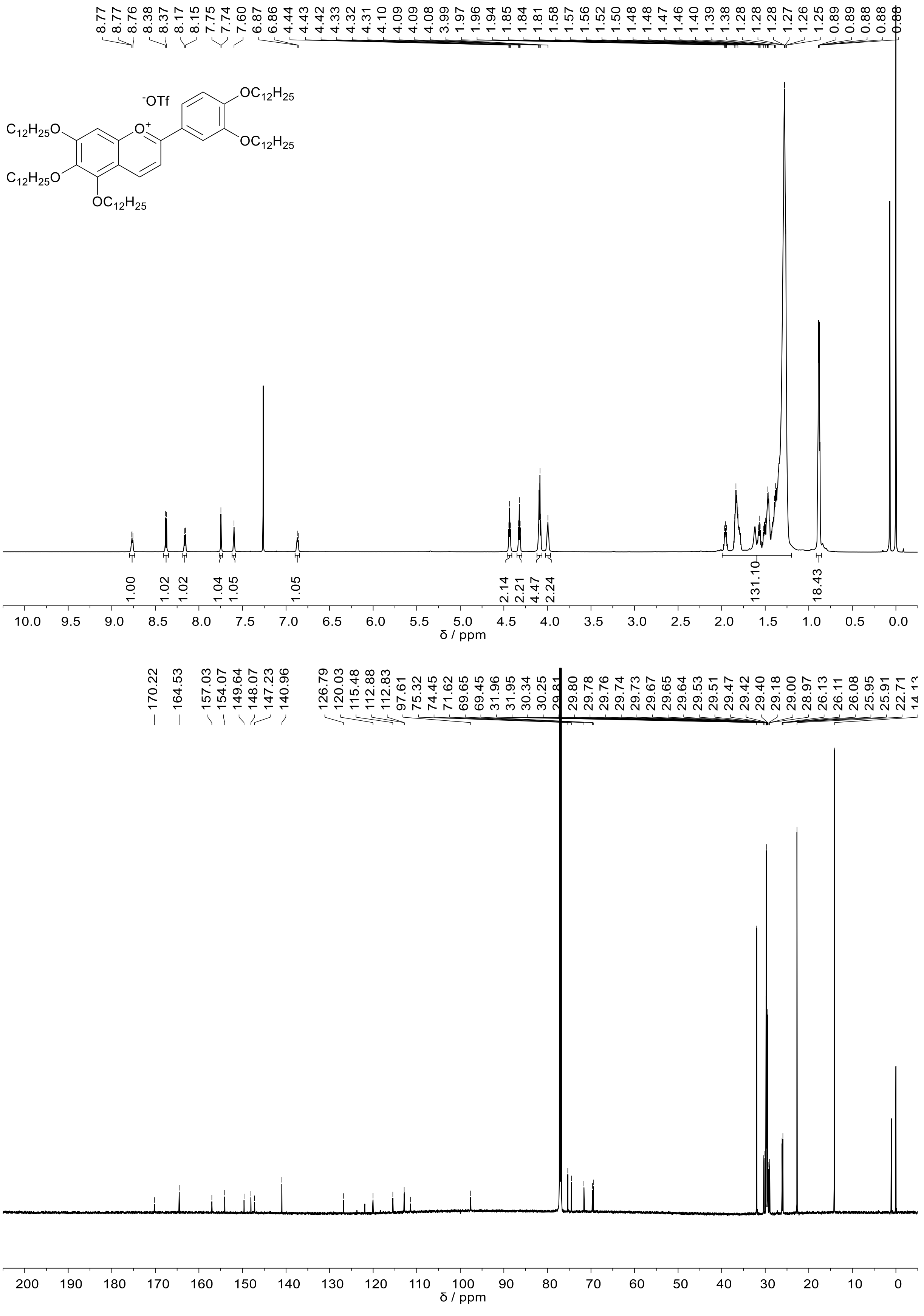

Figure S 66: ^{1}H (top, at 700 MHz) and ^{13}C NMR (bottom, at 176 MHz) of **3-Fla-2**.

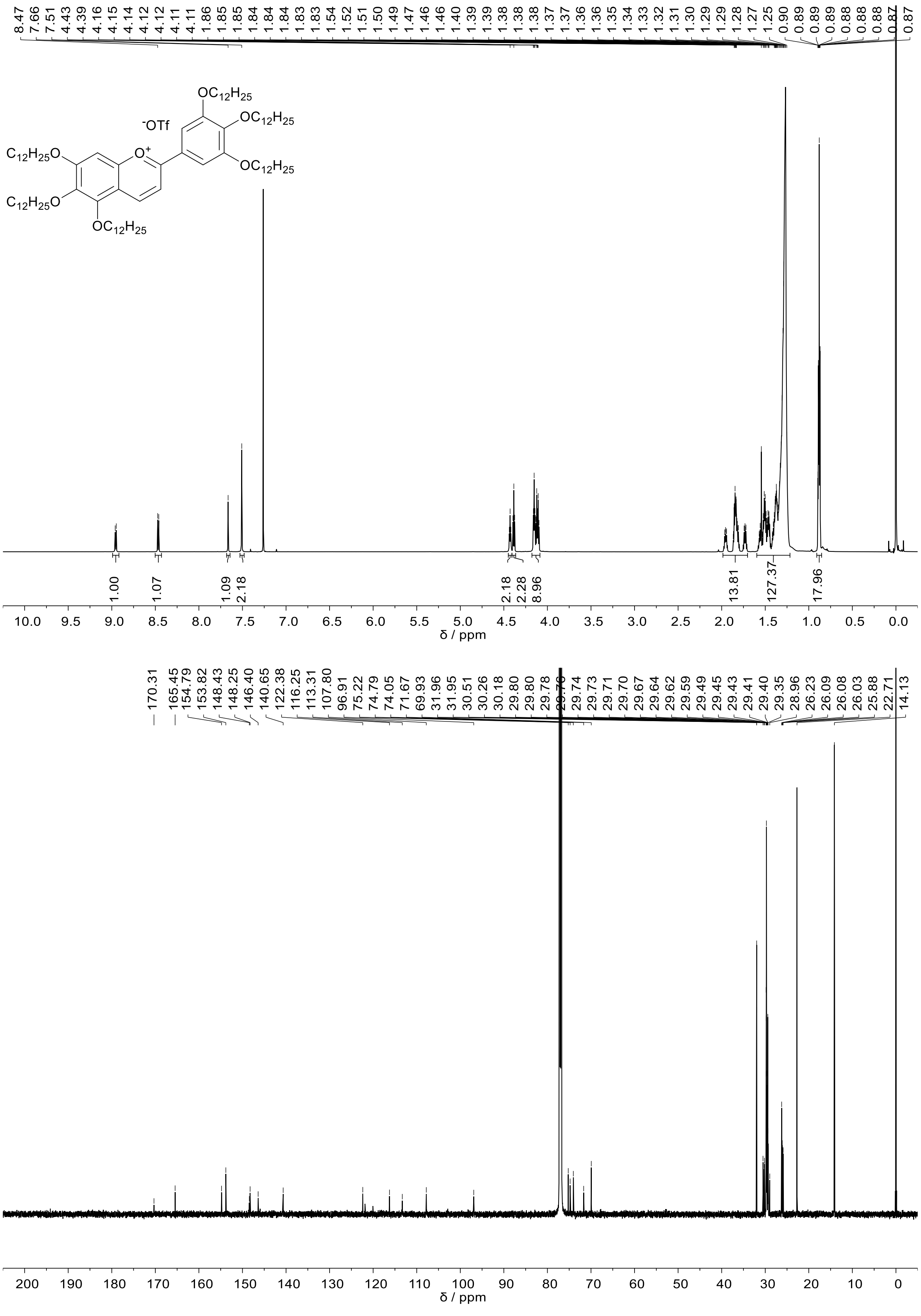

Figure S 67: ^{1}H (top, at 700 MHz) and ^{13}C NMR (bottom, at 176 MHz) of **3-Fla-3**.

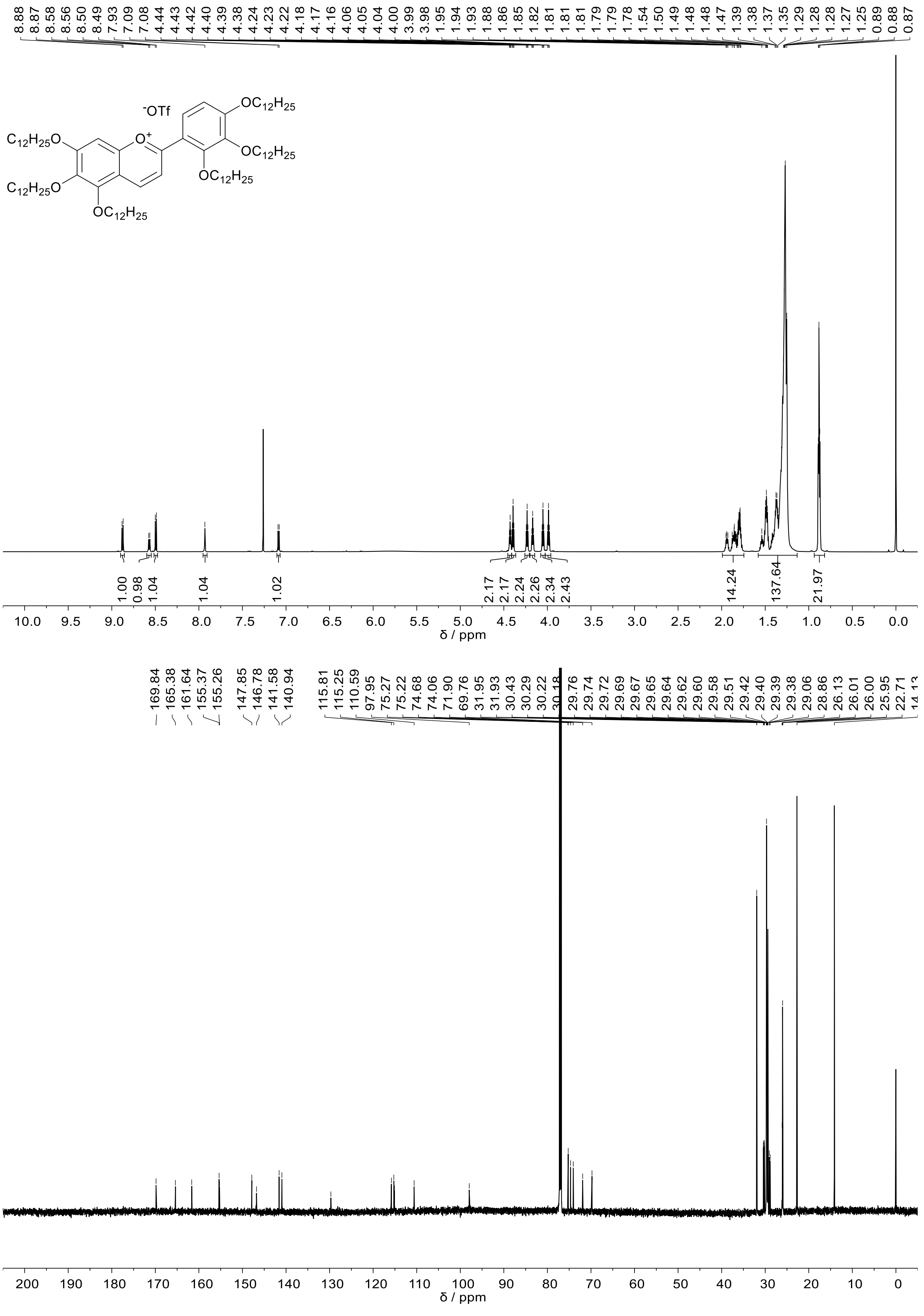

Figure S 68: ^{1}H (top, at 700 MHz) and ^{13}C NMR (bottom, at 176 MHz) of **3-Fla-3'**.

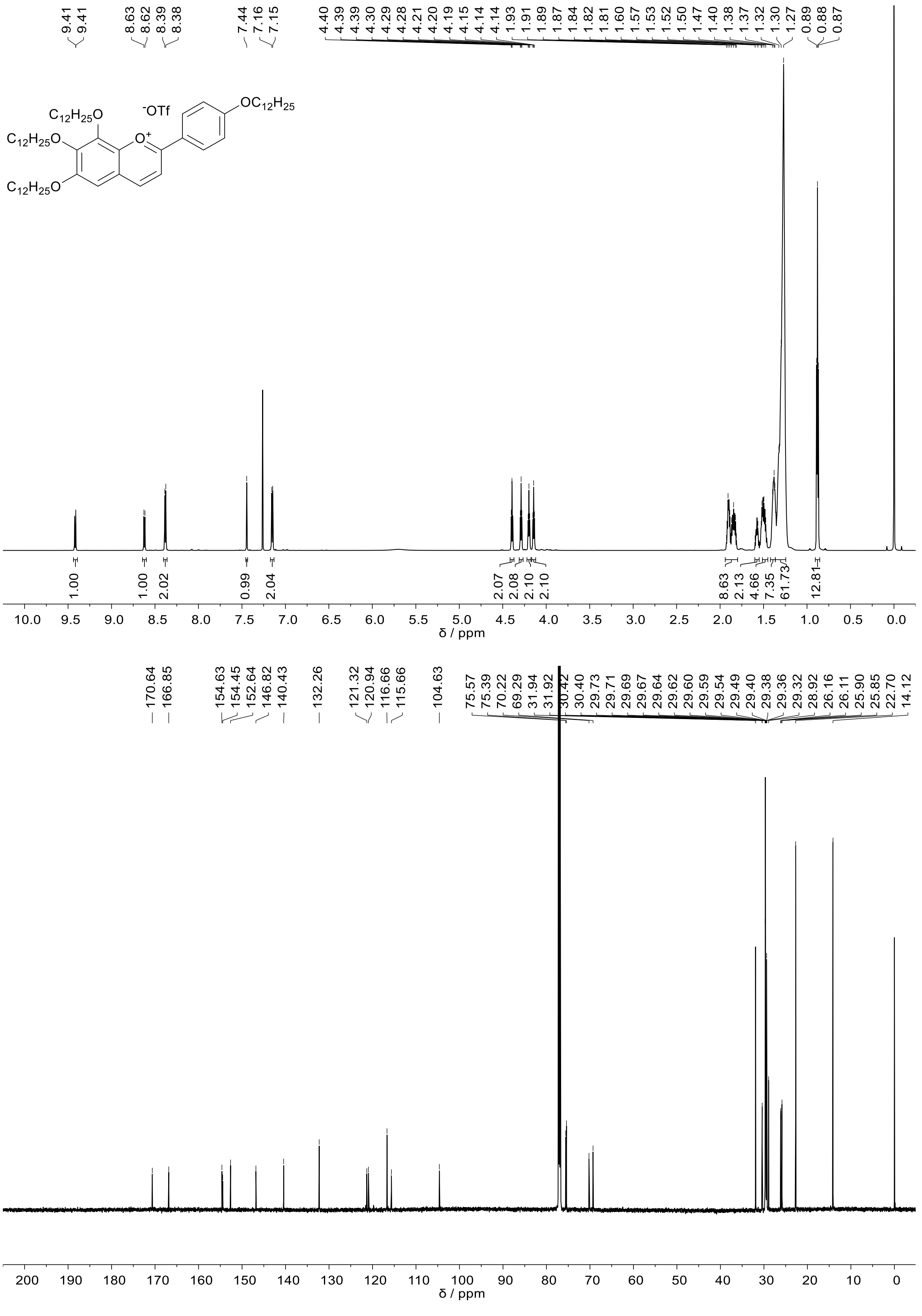

Figure S 69: ^{1}H (top, at 700 MHz) and ^{13}C NMR (bottom, at 176 MHz) of **3'-Fla-1**.

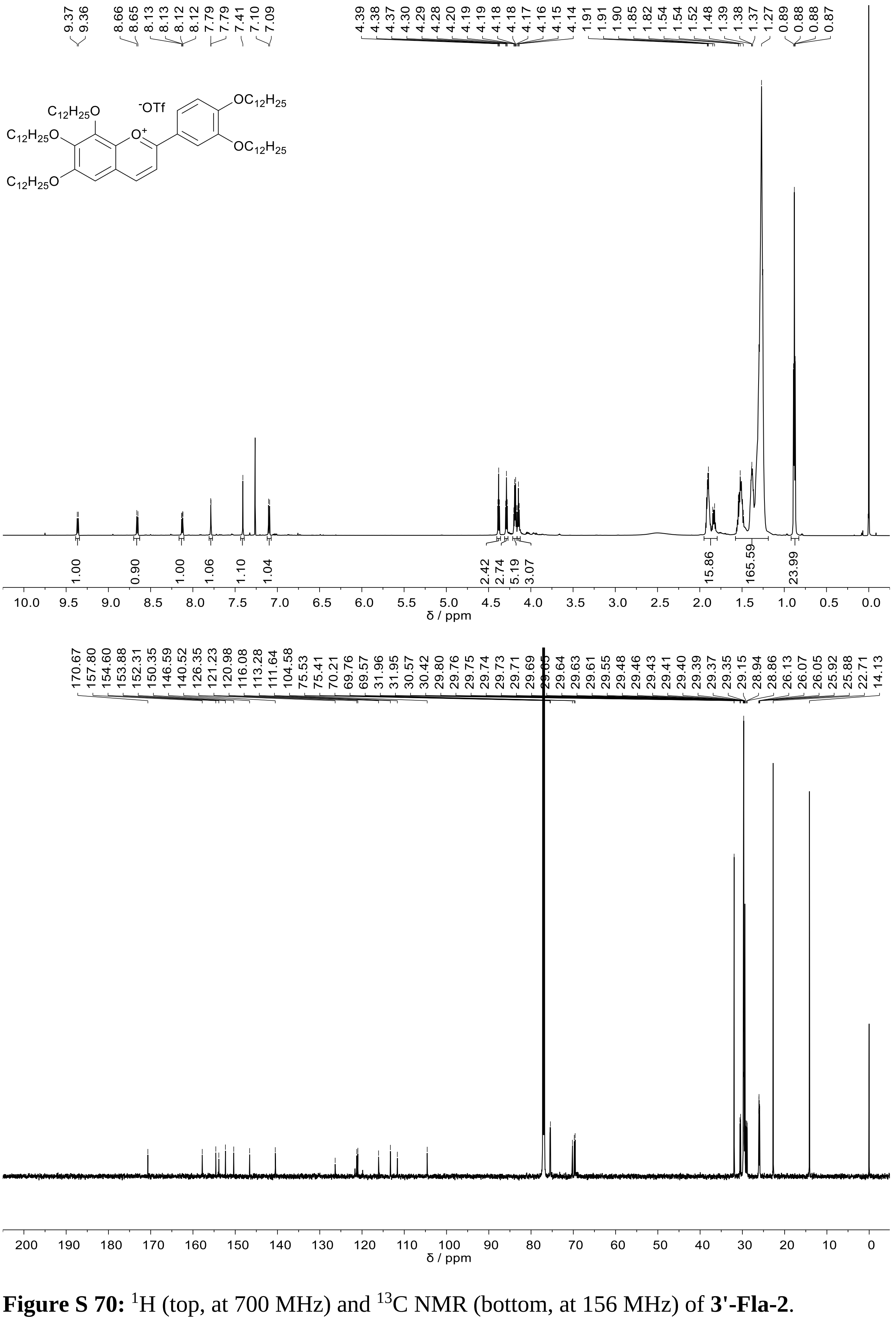

Figure S 70: ^{1}H (top, at 700 MHz) and ^{13}C NMR (bottom, at 156 MHz) of **3'-Fla-2**.

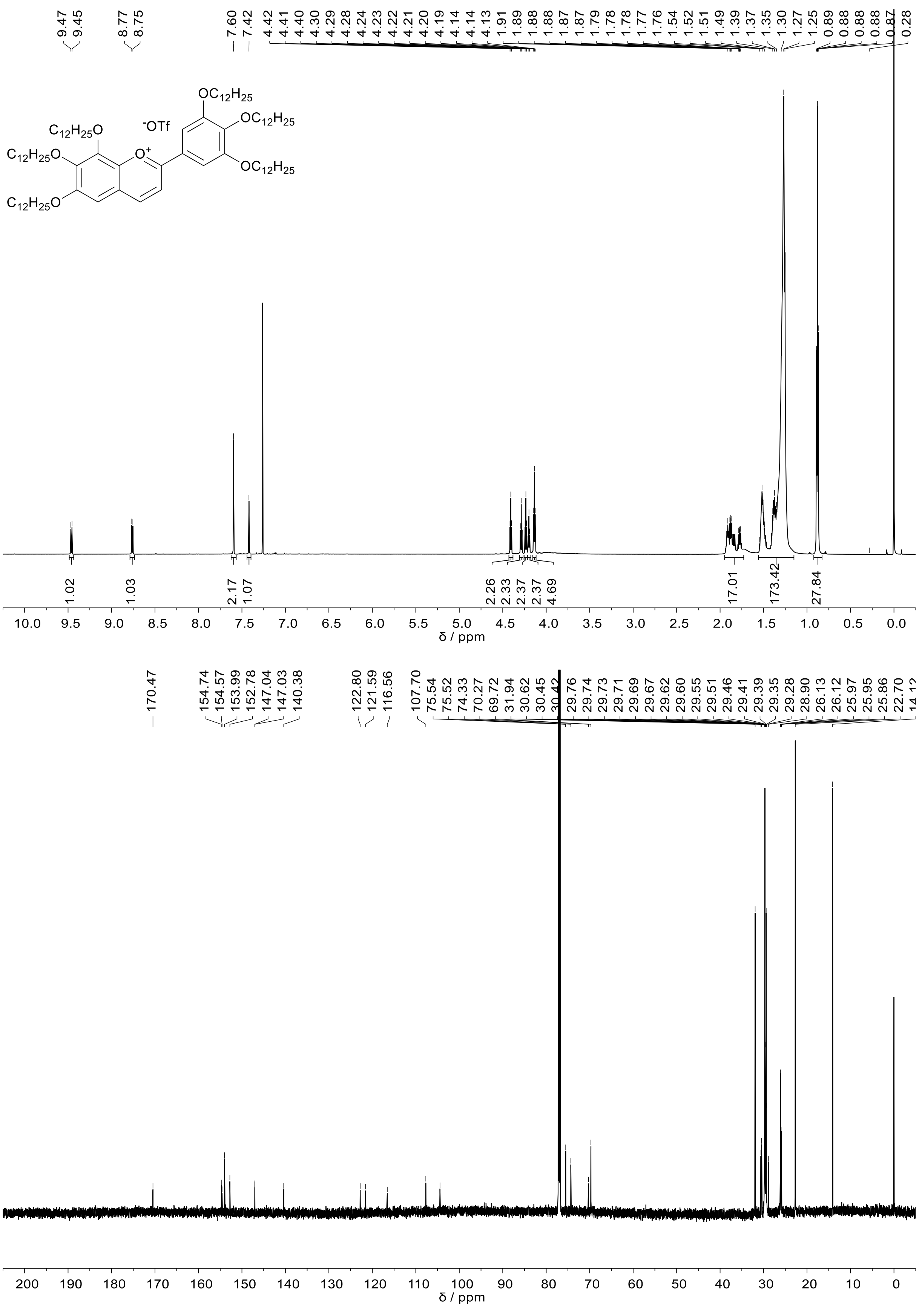

Figure S 71: ^{1}H (top, at 700 MHz) and ^{13}C NMR (bottom, at 176 MHz) of **3'-Fla-3**.

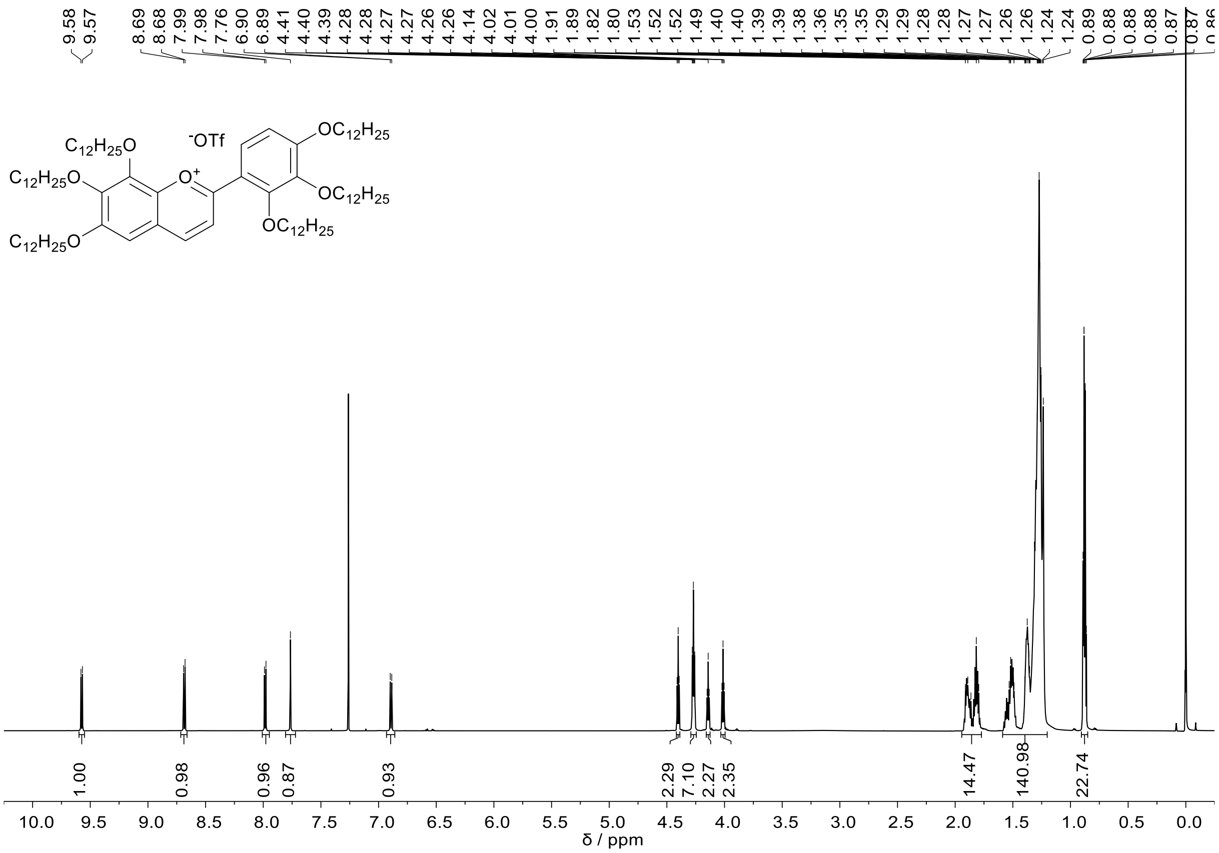

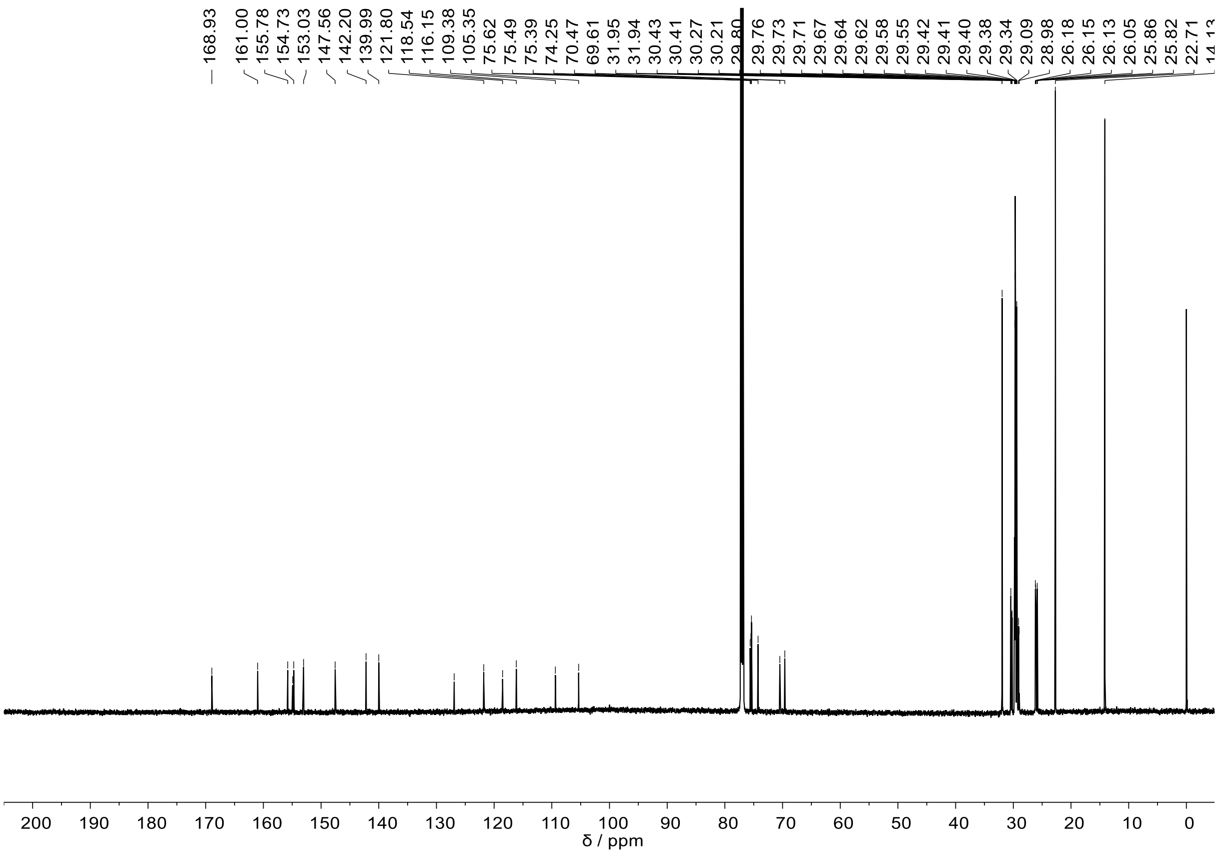

Figure S 72: ^{1}H (top, at 700 MHz) and ^{13}C NMR (bottom, at 176 MHz) of **3'-Fla-3'**.
